# Sequencing and Validation of the Genome of a *Campylobacter concisus* Reveals Intra-Species Diversity

**DOI:** 10.1371/journal.pone.0022170

**Published:** 2011-07-29

**Authors:** Nandan P. Deshpande, Nadeem O. Kaakoush, Hazel Mitchell, Karolina Janitz, Mark J. Raftery, Simone S. Li, Marc R. Wilkins

**Affiliations:** 1 Systems Biology Initiative, School of Biotechnology and Biomolecular Sciences, The University of New South Wales, Sydney, New South Wales, Australia; 2 School of Biotechnology and Biomolecular Sciences, The University of New South Wales, Sydney, New South Wales, Australia; 3 Ramaciotti Centre for Gene Function Analysis, The University of New South Wales, Sydney, New South Wales, Australia; 4 Biological Mass Spectrometry Facility, The University of New South Wales, Sydney, New South Wales, Australia; University of Vermont, United States of America

## Abstract

*Campylobacter concisus* is an emerging pathogen of the human gastrointestinal tract. Its role in different diseases remains a subject of debate; this may be due to strain to strain genetic variation. Here, we sequence and analyze the genome of a *C. concisus* from a biopsy of a child with Crohn's disease (UNSWCD); the second such genome for this species. A 1.8 Mb genome was assembled with paired-end reads from a next-generation sequencer. This genome is smaller than the 2.1 Mb *C. concisus* reference BAA-1457. While 1593 genes were conserved across UNSWCD and BAA-1457, 138 genes from UNSWCD and 281 from BAA-1457 were unique when compared against the other. To further validate the genome assembly and annotation, comprehensive shotgun proteomics was performed. This confirmed 78% of open reading frames in UNSWCD and, importantly, provided evidence of expression for 217 proteins previously defined as ‘hypothetical’ in *Campylobacter*. Substantial functional differences were observed between the UNSWCD and the reference strain. Enrichment analysis revealed differences in membrane proteins, response to stimulus, molecular transport and electron carriers. Synteny maps for the 281 genes not present in UNSWCD identified seven functionally associated gene clusters. These included one associated with the CRISPR family and another which encoded multiple restriction endonucleases; these genes are all involved in resistance to phage attack. Many of the observed differences are consistent with UNSWCD having adapted to greater surface interaction with host cells, as opposed to BAA-1457 which may prefer a free-living environment.

## Introduction

There is mounting evidence that members of the *Campylobacter* genus other than the well-established *Campylobacter jejuni* and *Campylobacter coli* play a role in intestinal disease. Indeed, they have been reported to account for a proportion of cases of acute gastroenteritis where no etiological agent is identified [1], [2]. *C. concisus* from this genus has received increasing attention over the last decade and has been described as an emergent pathogen of the human intestinal tract [3], [4], [5]. While historically, *C. concisus* has been associated with the human oral cavity and in a number of studies has been linked with periodontal lesions, including gingivitis and peridontitis [6], [7], [8], reports of the isolation of *C. concisus* as the sole pathogen isolated from fecal samples of diarrheic patients have been described [1], [2], [9], [10], [11], [12], [13]. Notably, studies that have used appropriate culture techniques for the highly fastidious *C. concisus* have reported it to contribute to a significant percentage (17–50%) of *Campylobacter* spp. cultured from fecal samples of patients with diarrhea [10], [12]. Furthermore a number of recent studies have reported both the detection and isolation of *C. concisus* from biopsy specimens and fecal samples of children with newly diagnosed Crohn's disease (CD) [14], [15]. While such studies would support the role of *C. concisus* as an intestinal pathogen, the isolation of *C. concisus* from healthy individuals, and the failure of some studies to show a significant difference in the prevalence of *C. concisus* in subjects with diarrhea and healthy controls [12], [13], [16], [17], has raised contention as to the role of *C. concisus* in intestinal disease. While these latter findings would argue, to some degree, against the role of *C. concisus* in gastroenteritis, the fact that great sequence diversity exists within *C. concisus* strains [12], [17] raises the possibility that differences may exist in the pathogenic potential of *C. concisus* strains [3].

Studies investigating the genetic make-up of *C. concisus* have shown that it is genetically and taxonomically diverse. For example, Vandamme *et al* using DNA-DNA hybridization reported a number of diarrheal isolates that fitted the phenotypic description of this species, to exhibit only 42 to 50% DNA-DNA hybridization values with the type and reference strains of oral origin [16]. This finding was supported by later studies using pulsed field gel electrophoresis (PFGE) that reported *C. concisus* to comprise at least two molecular groups (genomospecies), which were phenotypically indistinguishable, but genetically divergent [18], and by a protein profiling study by Aabenhus [11]. In a more recent study investigating the genotype of 62 *C. concisus* clinical isolates using amplified length fragment polymorphism analysis (ALFP), Aabenhus *et al* showed that *C. concisus* contained at least four distinct genomospecies, which led them to postulate that genomospecies may exhibit differences in virulence potential [19]. A recent publication, showing that only 1 of 6 *C. concisus* strains was able to colonize the intestinal tract of mice and induce weight loss, further supports this view [20]. To date there are limited studies investigating differences in the pathogenic potential of *C. concisus* strains. While Engberg *et al* have reported strain specific differences in the ability of *C. concisus* strains to induce cytolethal distending toxin-like effects on monkey kidney cells, no specific association with disease outcome was found [13]. The hemolytic phospholipase A_2_ activity and ability of *C. concisus* to adhere to and invade HEp-2 cells varies in strains from children with diarrhea, but this does not appear to be disease specific [21], [22].

A recent study by our group further substantiated the view that *C. concisus* strains are highly diverse [23]. We compared the attachment and invasive abilities of a range of *C. concisus* strains *in vitro*. This showed that a *C. concisus* strain isolated from a child with newly diagnosed CD (UNSWCD) had a significantly increased ability to attach to and invade human intestinal epithelial cell lines as compared with that of *C. concisus* strains isolated from two patients with gastroenteritis, respectively [23]. Interestingly, a *C. concisus* strain from healthy control attached but did not invade. Examination of the 16S rRNA gene, 23S rRNA gene and the internal transcribed spacer regions used to differentiate species within the *Campylobacter* genus, confirmed the UNSWCD strain to be a *C. concisus* [24].

Next-generation sequencing technologies have reduced the time and cost of whole-genome sequencing. They cost less than one-hundredth the amount of Sanger sequencing per base. Given that paired-end reads now exceed 100 bp, short-read sequencers have been used effectively for re-sequencing as well as the *de novo* assembly of many small prokaryotic genomes such as the bacterium *Pseudomonas syringae* [25], [26], [27]. At this time, the complete genome sequence of a single *C. concisus* strain, BAA-1457, isolated from a patient with gastroenteritis is available in the public domain as reference. In this work, we have produced a draft genome sequence of *C. concisus* UNSWCD. The genomic assembly was validated using a range of different methods including essential gene verification and shotgun proteomics. Comparative genomic analyses identified large differences between the UNSWCD strain and the reference strain BAA-1457. This study thus paves the way for further experimental investigation to determine the extent of heterogeneity within *C. concisus* species and analysis of the involvement of *C. concisus* in intestinal disease.

## Results and Discussion

### Genome assembly of *Campylobacter concisus* UNSWCD

The isolation of *C. concisus* from an intestinal biopsy of a child with CD allows for the investigation of heterogeneity within this bacterial species [14], [15]. The genome of *C. concisus* UNSWCD was first sequenced using 36 bp short reads produced by an Illumina/Solexa GII sequencer. A reference-sequence-based assembly was initially produced using the assembly tool, Bowtie. Bowtie aligned only 68.80% of the total Illumina reads to the reference BAA-1457 genome. Different strains of the same bacterial species have previously been shown to display high dissimilarities in the overall gene content of their genomes [28], the main reasons being attributed to horizontal gene transfer and gene loss. Pertinently a study by Matsheka *et al.* has suggested that the species of *C. concisus* as it is currently defined could in fact represent a taxonomic continuum comprised of several genomospecies [18], based on the high genetic diversity observed between strains of this species. For strains with genomes divergent from their closest references, reference-sequence guided assembly methods can provide limited genome definitions. Thus, our results from the above reference-based assembly method using Bowtie confirmed the requirement for *de novo* approaches to assemble the *C. concisus* UNSWCD genome.

**Figure 1:**
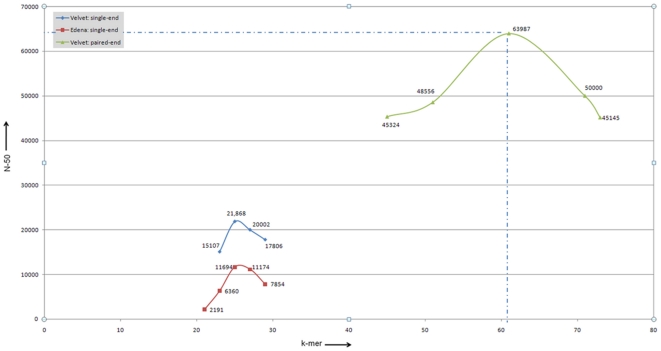
*De novo* assembly of *C. concisus* UNSWCD genome. k-mer values ranging from 19–33 were used for assembling the single-end read data for UNSWCD strain. The Velvet assembler generated a maximum N-50 value of 21,868 with a genome coverage of 1.68 Mb (352 contigs) where k = 25. The Edena assembler produced a bigger (1.79 Mb) but more fragmented assembly (459 contigs) with an overlap length of 25. Genome assembly was generated using a set of paired-end reads and Velvet assembler; this resulted in an improved base coverage (1.8 Mb) as well as a much improved N-50 value of 63,987 for a k-mer value of 61.

Given this, 36 bp reads were then used for *de novo* genome assembly. Using the Velvet assembly tool a range of k-mer values, which determine the amount of minimum read overlap, were used to reach the optimal genome assembly. For single-end reads a k-mer value of 25 was found to result in an optimized assembly with N50 of 21,868 bp, generating 352 contigs with the largest contig size being 102,216 and the genome size 1,684,142 bp (Figure 1 and Table S1). Contigs with a length smaller than 200 bp were removed prior to further analysis. The results from the Velvet assembly were then compared with assemblies generated using another *de novo* assembler, Edena [29]. When assembled using the overlap size parameter ranging from 21 to 29, Edena produced its best assembly of size 1,796,970 bases corresponding to an overlap size of 25. Interestingly, this assembly was larger in length than that from Velvet but was also more fragmented with 459 contigs and an N50 of 11,694.

To improve the length and quality of the *C. concisus* genome, it was then sequenced with the Illumina/Solexa GII sequencer using paired-end reads of 102 bp. A high level of coverage of the genome was obtained (2500×). Assembly was conducted by Velvet using a range of k-mers from 45–73. This generated a more compact assembly with an optimal k-mer value of 61 and a genome size of 1,805,982 bp (Figure 1). This assembly contained 123 contigs and it assisted in closing gaps between the previously assembled contig fragments. Figure S1 gives a brief snapshot of the two UNSWCD assemblies. In this figure, the single-end and paired-end assemblies have been visualized using the Integrative Genomic Viewer (IGV) viewer [30], with the *C. concisus* BAA-1457 genome used as a reference. Four contigs in Lane 1 (for single-end reads assembly) were merged to form a single contig in Lane 2 (for the paired-end reads assembly), which illustrates improvement in the quality of the UNSWCD genome using paired-end over single-end reads. In the larger genomic context 352 contigs produced by single-end reads were merged into 123 contigs in the paired-end assembly. The improvement in the assembly was reflected at the gene level as well, with more than 70% of the genes displaying fragmented homologs (across multiple contigs) in the single-end reads assembly being assembled as full length genes in the paired-end assembly.

After the assemblies were finalized, the UNSWCD genome was made up of 123 contigs and displayed a final genome size of 1,805,982 bp and an average GC content of 39.7%. The reference *C. concisus* genome from BAA-1457 strain (id : NC_009802 in NCBI), by comparison is made up of a single contig with a genome size of 2,052,007 bp and an average GC content of 39%.

### Gene definition and annotation of *Campylobacter concisus* UNSWCD

Gene definition and annotation was undertaken for individual assemblies using Rapid Annotation using Subsystem Technology (RAST), a service for annotating bacterial and archaeal genomes [31]. RAST includes a completely annotated genome for the reference species BAA-1457. RAST also includes the commonly used *ab initio* gene prediction program Glimmer [32]. The final annotated set from the assembled UNSWCD genome consisted of 1,763 genes. This Whole Genome Shotgun project has been deposited at DDBJ/EMBL/GenBank under the accession [GenBank: AENQ00000000]. The version described in this paper is the first version, [GenBank: AENQ01000000]. In comparison, BAA-1457 is comprised of 2010 genes, including 1929 protein coding genes (with RefSeq –YP identifiers), 81 recently annotated entries (without YP identifiers) and 59 structural RNAs.

### Differences between *Campylobacter concisus* UNSWCD and BAA-1457

In addition to using the comparative genomics modules in RAST, we also conducted customized homology searches using BLAST (blastp, tblastn) [33] to determine probable orthologs of genes conserved between the assembled *C. concisus* UNSWCD strain and the reference *C. concisus* BAA-1457. A total of 1593 of the 1,763 genes defined by RAST for the UNSWCD genome were found to possess orthologs in the BAA-1457 reference genome (Table S5) while 138 (7.8%) genes were specific to the UNSWCD strain. The presence of orthologs for remaining 32 genes could not be confirmed using comparative genomics techniques.

**Figure 2:**
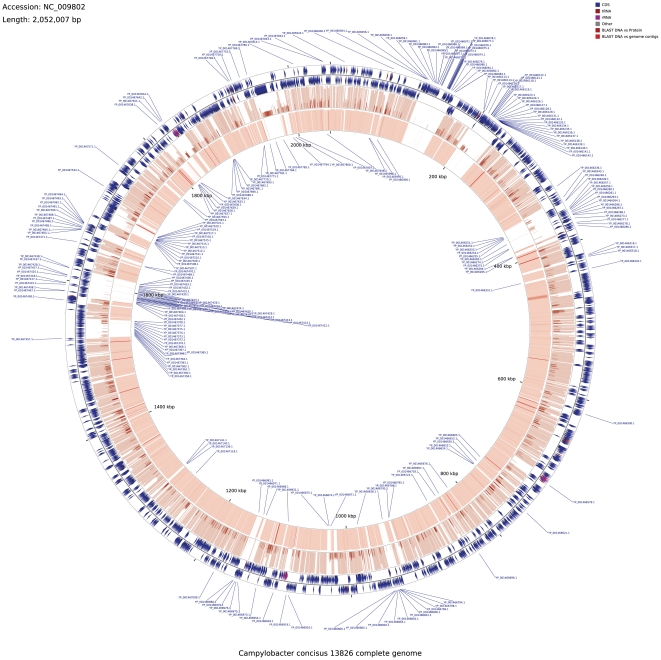
Differences in gene content across *C. concisus* BAA-1457 and UNSWCD genomes (visualized using CGView web server). 281 genes from positive and negative strands (outer ring I and II) of BAA-1457 were found to be absent in the UNSWCD genome and are labeled using gene symbols/CC_IDS. Ring III shows the homology of UNSWCD genome against BAA-1457. The innermost ring (IV) represents the proteomic identifications in the UNSWCD proteome from Orbitrap MS, used for validation of the UNSWCD genome.

Conversely, 1593 of the 2010 genes from the BAA-1457 were found to have orthologs in the assembled UNSWCD genome, while 281 (13.98%) were specific to BAA-1457 with no detectable homology in UNSWCD. A large number of genes from both UNSWCD (91/138) and BAA-1457 (130/281), specific to the respective strains could not be annotated using available information from public databases and hence were defined as hypothetical. Figure 2 shows a comparison of the UNSWCD genome against the reference BAA-1457 genome, drawn using the CGView web-server [34]. The outer two circles denote the genes from *C. concisus* BAA-1457 in the positive and negative orientations, respectively. 136 genes could not be verified to posses orthologs based solely on the bioinformatics study and these were grouped separately for further analysis. The 281 genes that were found to be absent in the UNSWCD assembly have been highlighted with their locus IDs or gene symbols. The third concentric circle (from outside) shows the genomic conservation of the UNSWCD strain with the reference BAA-1457. Apart from a few genes found deleted in isolation, most genes absent in UNSWCD as compared to BAA-1457 formed a series of groups. These were subjected to further syntenic association studies, below.

The sequence-based gene ontology annotation tool, Blast2GO [35] was used to categorize the genome-specific gene sets. Gene ontology definitions (including multiple GO definitions for any particular gene) obtained from Blast2GO were visualized as bar graphs on a normalized scale using the WEGO (Web Gene Ontology Annotation Plot) tool [36]. Around 46.26% of the genes (130/281) specific to BAA-1457 and 62.3% (86/138) specific to UNSWCD had no gene ontology definitions and are represented by unknown functions.

**Figure 3:**
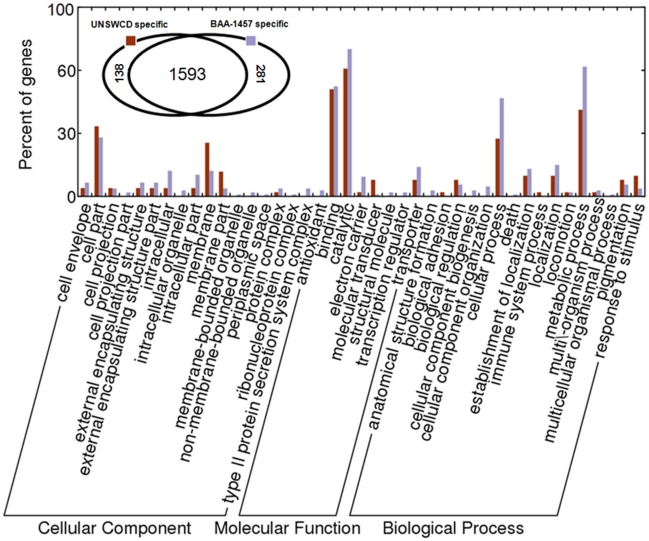
Gene ontology-based classification of genes specific to UNSWCD and BAA-1457 strains. Gene ontologies could be retrieved for 52 (out of 138) and 108 (out of 281) genes specific to *C. concisus* UNSWCD and BAA-1457 strains, respectively. Multiple GO categories (on the same hierarchical level-III) representing any particular gene are included in the above bar graph.

Gene ontologies such as membrane, membrane part, molecular transducer and response to stimulus in UNSWCD were found to be most over-represented when compared to its BAA-1457 specific counterparts (Figure 3). This greater number of unique membrane-related cellular components, and genes involved in the bacterium's response to stimuli may suggest that UNSWCD has adapted to greater surface interaction with its host, enabling a more efficient response to host-related stimuli. In contrast intracellular, electron carrier, cellular process, transporter, metabolic process were under-represented in UNSWCD in comparison with the BAA-1457 specific genes. This difference of intracellular components, transporter molecules and electron carriers in BAA-1457 suggests it may utilize additional energy sources to UNSWCD, perhaps due to its preference for a free-living as opposed to host cell-associated environment. Further investigation of the group comprising the non-GO categorized genes (a large group of unknown function) could prove important in elucidating the differences between the two *C. concisus* strains.

### Plasmids within Campylobacter concisus

**Figure 4:**
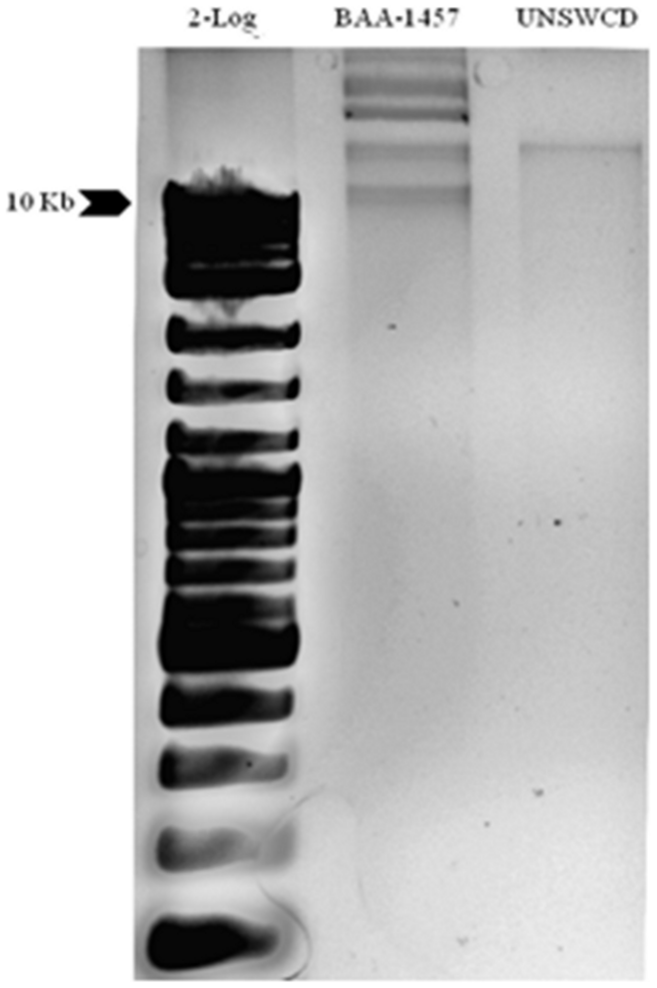
Purification of plasmids from *C. concisus* UNSWCD and BAA-1457. Purified plasmids were electrophoresed through a 1.5% agarose gel and visualized with Gel-Red staining. There were 5 bands present in BAA-1457 but only 1 band present in UNSWCD.

Plasmids can confer a variety of physiological advantages for bacterial strains such as antibiotic resistance or virulence. One essential aspect of bacterial diversity relates to the plasmids that each strain contains, and the proteins that they encode. To investigate the differences in plasmids between *C. concisus* UNSWCD and BAA-1457, plasmid DNA was purified from both strains. Five bands were observed for the BAA-1457 strain but only one band was observed in UNSWCD (Figure 4). Interestingly, only two plasmids were reported in the genomic sequence analysis of BAA-1457 (pCCON16 and pCCON31; accession numbers NC_009796 and NC_009795 respectively).

To identify the plasmid sequence within UNSWCD and any similarity to plasmids in BAA-1457, genes within the two reported BAA-1457 plasmids were matched against all UNSWCD contigs, using Blast. Only five genes from the plasmid pCCON16 and none of the genes from the plasmid pCCON31 were found to be conserved in the UNSWCD assembly. The five genes from the plasmid pCCON16 were present in different contigs of UNSWCD, and thus unlikely to be within a single plasmid of UNSWCD. This also indicates that the plasmid pCCON16 (or parts of it) were actually incorporated into the genome of UNSWCD, and that UNSWCD could have a unique plasmid. Subsequently, we sought to identify any origin of replication (*ori*) within UNSWCD contigs of approximate size 10–30 kb. *Ori* are generally made up of approximately four tandem repeats, flanked by an AT-rich region [37], [38]. In the BAA-1457 strain, the smaller reported plasmid (pCCON16∼16 kb) had an *ori* made up of 4.2 tandem repeats of approximately 43 nucleotides. However, no *ori* sequences were present in the 10–30 kb contigs of the UNSWCD genome.

### Genome validation I: Essential gene components in the assembled genome

The UNSWCD genome appeared to be smaller than the genome of *C. concisus* BAA-1457. To further understand the nature of this difference and to validate the sequenced genome we investigated whether the assembled UNSWCD genome contained a minimal set of essential genes required for bacterial survival. *Mycoplasma genitalium* is known to be the organism with the smallest genome that can be grown in pure culture [39]. *Helicobacter pylori* is a Gram-negative bacterium with the closest phylogeny to *C. concisus* in the group of bacterial genomes with a defined essential gene set as well as a similar genome size (1,566,651 bp). The database of essential genes (DEG) [40] was queried and essential genes from *M. genitalium* and *H. pylori* were downloaded. The UNSWCD and BAA-1457 genomes were found to contain an identical set of 364 and 166 essential genes from the set defined in *M. genitalium* and *H. pylori*, respectively. Conservation of all the essential genes validated the UNSWCD genome assembly, illustrating that the smaller genome size of UNSWCD is unlikely to be due to errors in assembly or annotation.

### Genome validation II: ‘Reverse annotation’ of *C. concisus* UNSWCD with proteomics

**Figure 5:**
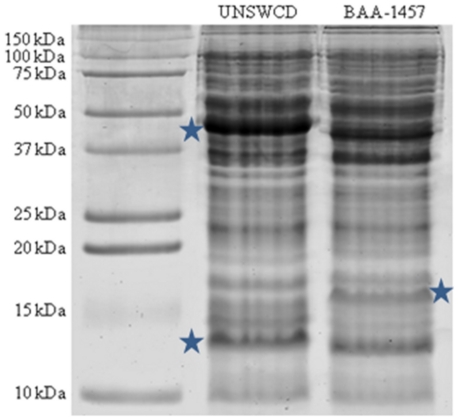
One-dimensional polyacrylamide gel electrophoresis of whole cell lysates of *C. concisus* UNSWCD and BAA-1457. Each gel lane for each strain was sectioned into 25 gel slices and processed for mass spectrometry analysis. Regions within the gel labeled with stars correspond to areas reflecting high diversity between the protein profiles of the two strains.

In order to comprehensively validate the assembly of the *C. concisus* UNSWCD genome, we undertook an in-depth analysis of the UNSWCD proteome. Whole cell lysates of UNSWCD were separated by 1-D SDS-PAGE (Figure 5). All protein bands were systematically cut from these gels, digested to peptides and analyzed by Orbitrap tandem mass spectrometry. Mascot searches were carried out against the proteins putatively encoded from the UNSWCD genome assembly. Proteins identified with a significance threshold of *P*<0.05 were filtered for downstream analysis (Figure S3 details protein identifications of genes specific to UNSWCD). The expression of 1,369 proteins (72.9% of the total ORFs) was validated using this approach. Importantly, 217 hypothetical proteins were unambiguously identified (Table S2). Whilst this does not provide clues to the function of these proteins, it unequivocally confirms their expression in the UNSWCD strain. The protein expression of 49 out of the 138 genes specific to UNSWCD was confirmed from this analysis (Table S3). In Figure 2 the proteomic identifications for UNSWCD have been mapped as the fourth concentric circle against the reference genome. As a further validation of the *C. concisus* UNSWCD genomic assembly, it was observed that none of the protein identifications matched to the genes encoding proteins specific to BAA-1457.

Proteomic analysis of BAA-1457 under similar experimental conditions was also carried out. This led to the identification of 1,321 proteins (65.7%) in BAA-1457 which included 220 hypothetical proteins. Furthermore, it was observed that none of the protein identifications in BAA-1457 matched to the genes encoding proteins specific to UNSWCD.

### Comparison of the proteomes of the *Campylobacter concisus* strains

**Figure 6:**
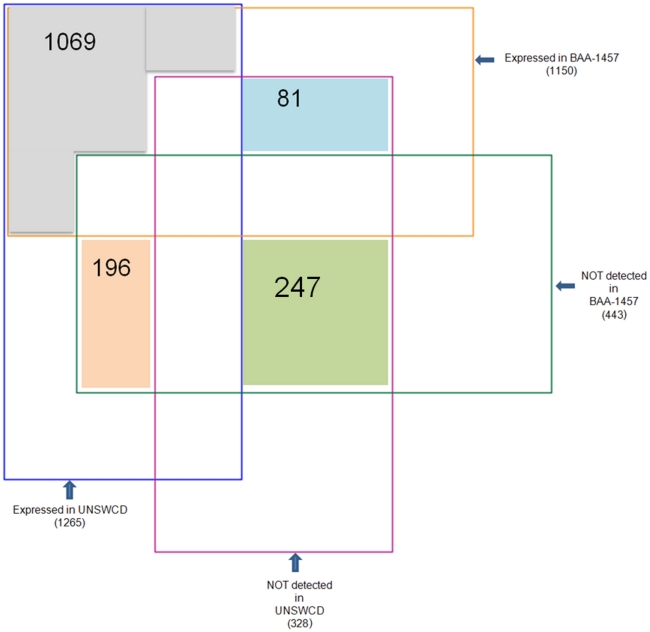
Proteomic comparison of the conserved genomes of *C. concisus* UNSWCD and BAA-1457. Orbitrap MS-MS analysis was used for high throughput proteomic profiling of the 1593 genes conserved across *C. concisus* strains UNSWCD and BAA-1457. 1069 genes were expressed in both strains while no peptides representing 247 genes were detected in either UNSWCD or BAA-1457. 196 genes with protein identifications only in UNSWCD and 81 genes with protein identifications only in BAA-1457 are of particular interest.

One-dimensional gel electrophoresis of cell lysates revealed many differences between the protein profiles of UNSWCD and BAA-1457 (Figure 5). Examples of these differences were proteins of approximately 45, 17 and 13 kDa (highlighted with stars). These differences provide evidence that the proteomes of these two strains vary even when grown under the same experimental conditions. This is to be expected, given the differences in their genomes. The comprehensive protein identifications from both UNSWCD and BAA-1457 strains, above, provided us with a platform for the comparison of proteomes of these *C. concisus* strains. A four-way Venn diagram was drawn to depict the similarities and differences in protein expression of the 1593 genes conserved in the *C. concisus* UNSWCD and BAA-1457 genomes (Figure 6). 1069 proteins were expressed by both UNSWCD and BAA-1457 strains while 247 proteins were marked by their absence in both. Proteins unique to each strain (81 genes expressed in BAA-1457 but not identified in UNSWCD; 196 expressed in UNSWCD but not identified in BAA-1457) were also evident. This illustrates that even under identical growth conditions; different strains of *C. concisus* express markedly different sets of proteins.

### Identifying novel genes from open reading frames using MS-MS data

The above MS-MS approach identified an extremely large proportion of proteins in the UNSWCD proteome. However, these identifications relied entirely on the accuracy of the gene prediction tools used in our analysis as proteomic data was matched only against predicted ORFs. To ensure that we had accurately identified the majority of genes in the UNSWCD genome, we applied a six-frame translational approach previously developed by Arthur *et al* [41] to identify novel open reading frames not picked out by traditional gene prediction programs or homology-based comparative analysis methods. Here, all contigs from the assembled UNSWCD genome were processed into 2000 bp hypothetical overlapping gene fragments. These fragments were then translated into 6 frames to yield 6 virtual proteins per fragment. These virtual proteins were then queried by the MS-MS data with the Mascot tool to identify further open reading frames. Two additional proteins were found using this process. A single peptide with a significant score of 67 was mapped to an ORF with a length of 112 aa. This protein sequence showed significant similarity to a ‘chain-length determinant protein’ in *Campylobacter hominis* (YP_001406017.1). A protein belonging to the pseudaminic acid synthesis pathway was also identified using this approach (253 aa). This protein was found in the UNSWCD strain but was absent in BAA-1457.

### Syntenic networks to analyze the association of reference genes absent in *C. concisus* UNSWCD

**Figure 7:**
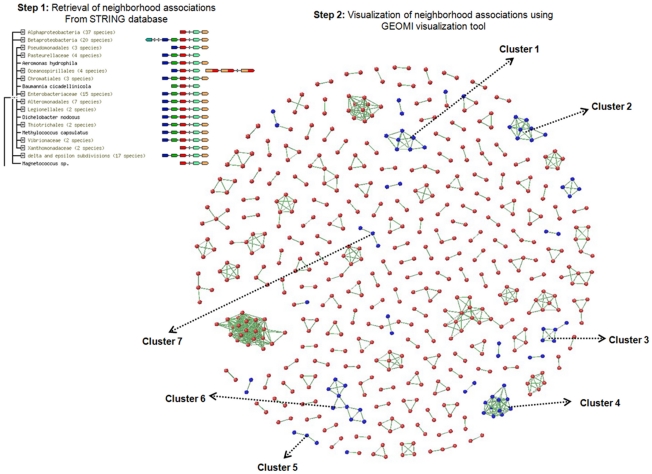
Synteny maps for genes from BAA-1457 found absent in UNSWCD genome. Clusters in the network include genes which were found associated with each other in the STRING database on the basis of syntenic conservation across multiple bacterial species. Blue nodes in the network represent genes from BAA-1457 with no conservation in the UNSWCD genome.

As a result of the above analyses, we predicted 1,763 ORFs in UNSWCD and confirmed 1,377 by proteomic analysis. An identical set of essential genes was also shown to be shared with the BAA-1457 strain. This suggested that our genome assembly and analysis was of very high quality. There were, however, numerous genes that were absent in UNSWCD as compared to the reference BAA-1457 strain. We investigated whether these were adjacent to each other in the genome of BAA-1457, and thus likely to have been lost *en bloc*, by developing ‘syntenic networks’. Figure 7 shows the syntenic network developed using the visualization platform GEOMI [42] for the *C. concisus* BAA-1457 specific gene set. Syntenic associations from the STRING database [43] were probed for the complete set of 2010 genes from *C. concisus* BAA-1457. A total of 827 associations were found to have strong syntenic partners based on a combined score of >900. Of these, 133 associations involving 58 of the 281 BAA-1457 genes absent in UNSWCD were analyzed further. Network analysis showed that 7 gene clusters were found to be comprised entirely of genes absent in UNSWCD (Figure 7). This suggests that the genes in each cluster were lost, together, from a specific region of the genome. Further investigation of these BAA-1457 specific gene clusters revealed that the syntenically related genes in at least of two of the seven clusters belonged to functionally related protein families.

A cluster of seven genes (Cluster 1) belonging to ‘Clustered Regularly Interspaced Short Palindromic Repeats (CRISPR) family’ were identified by the STRING database [43]. The CRISPR are a family of DNA direct repeats separated by regularly sized non-repetitive spacer sequences and appear to provide acquired resistance against bacteriophages [44]. Additionally, a cluster of three genes from *C. concisus* BAA-1457 (Cluster 5) showing restriction-related activity were found to be either completely or partially absent in UNSWCD. While the type I restriction modification DNA specificity domain-containing protein (CCC13826_1411) was found to be completely deleted, specific cleavage of the functional restriction domains was observed in the other two proteins, type I restriction enzyme EcoR124II R protein (CCC13826_1410) and type I restriction-modification system, M subunit (CCC13826_1412), with complete conservation of the remaining protein sequences (Figure S2). This would render these enzymes non-functional. The loss and/or loss of activity of the restriction related genes in the UNSWCD genome is of specific interest given that endonucleases are one line of defense against invading phage. Along with the loss of CRISPR genes, this suggests that the UNSWCD strain has lost some of its capacity to resist phage attack, possibly due to its invasive nature within the host [23].

**Figure 8:**
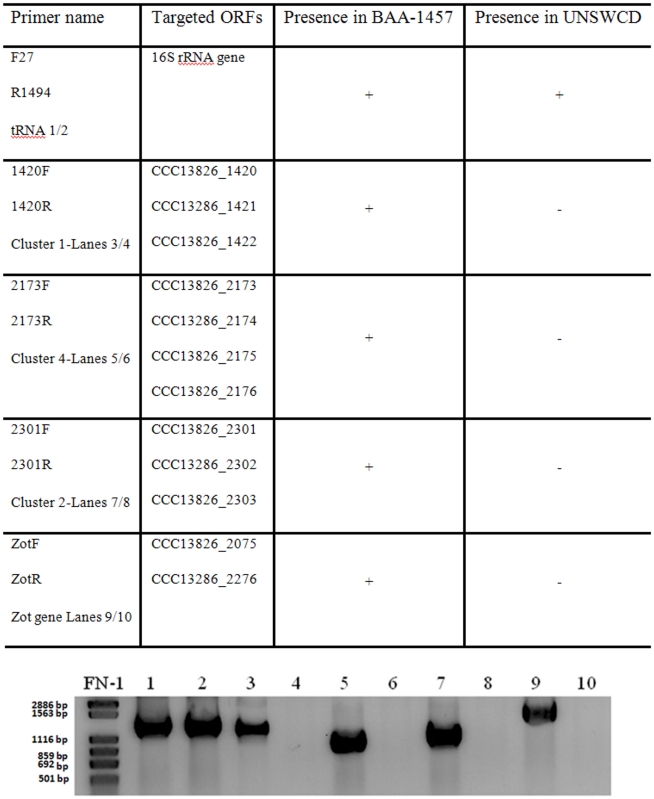
PCR confirmation of missing clusters. Lanes 1 and 2 correspond to 16s rRNA genes from *C.concisus* BAA-1457 and UNSWCD strains. Lanes 4,6,8,10 represent the putatively deleted clusters in UNSWCD (all genes in these clusters have been shown to be deleted in the assembled UNSWCD genomic sequence), while lanes 3,5,7,9 represent the corresponding gene clusters in BAA-1457.

As a further confirmation that gene clusters have been lost in UNSWCD, we chose several of these clusters (1, 2 and 4) and the *zot* genes (ccc13826_2075 and ccc13826_2276) to confirm their presence or absence with PCR (Figure 8). Amplification of the extracted DNA revealed that all three clusters and the two *zot* genes were present in BAA-1457 but were absent in UNSWCD, thus, confirming our genome assembly and synteny analyses. The 16S ribosomal RNA (rRNA) genes of both strains were amplified using the primers F27 and R1494 to ensure that lack of amplification within UNSWCD was not due to DNA degradation.

### Conclusions

This work involved the sequencing and validation of the genome of a *C. concisus* strain isolated from a child with CD. This provides the second *C. concisus* genome in addition to the reference BAA-1457. Sequence comparisons to identify orthologs, essential gene verification analysis, syntenic association maps and proteomic validations by Orbitrap tandem mass spectrometry revealed a highly accurate assembly but one with significant differences to *C. concisus* BAA-1457. A number of genes (138/1763 or 7.8%) from *C. concisus* UNSWCD were found to be unique when compared with *C. concisus* BAA-1457 genome. These were observed despite the UNSWCD genome being smaller than the reference genome. Conversely, 281/ 2010 (13.98%) of genes from *C. concisus* BAA-1457 were unique to this strain when compared with the assembled *C. concisus* UNSWCD genome. The differences between UNSWCD and the reference BAA-1457 are associated with specific functions, including the loss of mechanisms to resist phage attack, and differences in response to stimuli and molecular transporters. Together, these suggest that UNSWCD may have adapted to greater surface interaction with host cells as opposed to BAA-1457 which may prefer a free-living environment.

## Materials and Methods

The bacterial strain was isolated by our group in a previous study [15]. For this study, as no human or animal samples were used, no ethics approval was required. For the previous study by our group from which the strain was obtained, we have provided ethics approval in the published manuscript [15]. Full details of the ethics approval for that study are as follows:

This work was approved by the South Eastern Sydney Area Health Service and the Human Ethics Committee of the University of New South Wales (Human Ethics Research Committee no. 03/165), the ethics committee at Children's Hospital Westmead (Human Ethics Research Committee no. 2007/008), and the ethics committee at IKW (Human Ethics Research Committee no. 3725). For the previous study in which the strain was isolated from a child with Crohn's disease, informed consent was obtained from the parent/guardian and this is stated in the manuscript [15].

The work flow for the sequencing, assembly, annotation and validation of the UNSWCD genome is summarized in Figure S3.

### Sample preparation and genome sequencing

*Campylobacter concisus* strains UNSWCD and BAA-1457 were grown on Horse Blood agar (HBA) supplemented with 6% defibrinated horse blood (Oxoid; Heidelberg West, VIC, Australia). Cultures were incubated at 37°C under microaerobic conditions generated using *Campylobacter* Gas Generating Kits BR0056A (Oxoid). The purity of bacterial cultures was confirmed by motility and morphology observed under phase contrast microscopy. Bacterial DNA was extracted using the Puregene Core kit A (Qiagen; Hilden, Germany) according to the manufacturer's instructions.

The genomic DNA of *C. concisus* UNSWCD was sequenced using the Illumina Genome Analyser (GAIIx) following the standard Illumina protocol. The sample was prepared using the Illumina paired-end sample preparation kit. Briefly, 5 µg of DNA was fragmented by nebulization followed by end–repaired ligation of the adaptors. The size selection was performed using 2% agarose gels. This resulted in the recovery of 350 bp fragments. Ten cycles of PCR were used to enrich the adapter-modified DNA fragments. The library was finally purified using the QIAquick PCR purification kit, diluted with Elution Buffer (Qiagen) to a final concentration of 10 nM, and store at −20°C until use. The sample was run at two different concentrations 7 pM and 8 pM using two runs. The first run was of 36 bp and the second run of paired-end 102 bp chemistry, respectively. The first run (36 bp) was performed using the Genome analyzer sequencing control software (SCS) v2.4 and the second run (paired-end 102 bp) using the SCS v2.6.

Using the Illumina's quality filtering parameters 85% of the clusters with single read 36 bp sequencing and 64% clusters with PE 2×102 bp were selected for assembly. Using the above parameters 14 million reads in case of 36 bp sequencing and 56 million reads in case of 102 bp sequencing were generated, respectively.

### Genome assembly

A custom Perl script was developed to trim low quality bases near the end of reads corresponding to unreliable quality scores marked by ‘B’s by the Illumina analyzer. The trimmed reads were assembled by the *de nov*o assembly tool Velvet 1.0.09 [45]. Different values for k-mers, indicating the amount of minimum read overlap, were used to reach the optimal genome assembly size. The Velvet assembly tool is known for its speed, higher contig lengths and accuracy [45]. Velvet is based on a directed graph representation called de Bruijn graphs which uses non-redundant sets of k-mers or word length rather than sequence reads as its primary data structures. Apart from imparting speed to the assembly (∼20 min to assemble the *C. concisus* genome of size ∼1.7 Mb), the high redundancy in short reads are better supported using the graph approach. Velvet was used for assembly using k-mer values 23–29 for the 36 bp single-end read and from 41–73 for the 102 bp paired-end reads. The UNSWCD genome was also assembled using the single-end 36 bp reads with another fast assembly algorithm, Edena. Edena works on the classical graph approach and the tool is known for its efficiency in handling base errors and detecting potentially spurious reads [29].

### Gene prediction and identifying orthologs

The *C. concisus* BAA-1457 genome (NC_009802) and RefSeq fasta sequences for 2010 protein coding genes were downloaded from the NCBI website. The RAST web application server was used for gene predictions using the Glimmer program. Comparative genomics modules available in RAST were used for gene based comparisons between UNSWCD and BAA-1457 genomes. In addition, gene ontology predictions using Blast2GO, and functional domain analysis with both SMART and Pfam were used. Locally installed NCBI BLAST v 2.2.22 and NCBI BLAST web server programs were integrated with customized python scripts for detailed sequence alignment and analysis.

### Plasmid purification and bioinformatic analysis

*Campylobacter concisus* strains UNSWCD and BAA-1457 were grown in Brain Heart Infusion broth supplemented with 10% fetal bovine serum. Cultures were incubated at 37°C under microaerobic conditions generated using *Campylobacter* Gas Generating Kits BR0056A (Oxoid). Plasmid DNA was extracted and purified using the low copy number protocol from the HiYield Plasmid mini kit (Real Biotech Corporation; Banqiao City, Taipei County, Taiwan). Plasmid DNA was electrophoresed through 1.5% agarose gels at 100 V for 1.5 h. The web-based tool ‘Tandem Repeat Finder’ [46] was used to check the presence of origins of replication (*ori*) within contigs of approximately 10–30 kb size.

### Proteomic validation of the *C. concisus* UNSWCD genome

*C. concisus* UNSWCD and BAA-1457 were grown on HBA plates, and bacteria were washed three times in NaCl (150 mM). Following the final wash, packed cells were resuspended in 1 ml TSU buffer (50 mM Tris pH 8.0, 0.1% SDS, 2.5 M urea) and lysed by two freeze-thaw cycles in liquid nitrogen. Lysate proteins (40 µg) were separated and digested as previously described [47].

Digested peptides were separated by nano-LC using an Ultimate 3000 HPLC and autosampler system (Dionex; Amsterdam, Netherlands). Samples (1 µl) were concentrated and desalted onto a micro C18 pre-column (500 µm×2 mm, Michrom Bioresources; Auburn, CA, USA) with H_2_O∶CH_3_CN (98∶2, 0.05% trifluoroacetic acid) at 15 µl min^−1^. After a 4 min wash the pre-column was switched (Valco 10 port valve; Dionex) into line with a fritless nano column (75 µ×∼10 cm) containing C18 media (5 µ, 200 Å Magic; Michrom) manufactured according to Gatlin [48]. Peptides were eluted using a linear gradient of H_2_O∶CH_3_CN (98∶2, 0.1% formic acid) to H_2_O∶CH_3_CN (64∶36, 0.1% formic acid) at 250 nl min^−1^ over 30 min. High voltage (2000 V) was applied to low volume tee (Upchurch Scientific) and the column tip positioned ∼0.5 cm from the heated capillary (T = 280°C) of an Orbitrap Velos (Thermo Electron; Bremen, Germany) mass spectrometer. Positive ions were generated by electrospray and the Orbitrap operated in data dependent acquisition mode (DDA).

A survey scan m/z 350–1750 was acquired in the Orbitrap (Resolution = 30,000 at m/z 400, with an accumulation target value of 1,000,000 ions) with lockmass enabled. Up to the 10 most abundant ions (>5,000 counts) with charge states >+2 were sequentially isolated and fragmented within the linear ion trap using collisionally induced dissociation with an activation q = 0.25 and activation time of 30 ms at a target value of 30,000 ions. M/z ratios selected for MS/MS were dynamically excluded for 30 s.

Peak lists were generated using Mascot Daemon/extract_msn (Matrix Science, Thermo; London, England) using the default parameters, and submitted to the database search program Mascot (version 2.1, Matrix Science). Search parameters were: Precursor tolerance 4 ppm and product ion tolerances ±0.4 Da; Oxidation (M) and Carbamidomethyl (C) specified as variable modifications, enzyme specificity was trypsin, 1 missed cleavage was possible and the *C. concisus* BAA-1457 or UNSWCD complete proteome sequences searched.

In an attempt to identify novel genes, the assembled genome was theoretically cleaved into equal sized overlapping sequence fragments of 2000 bp. The dataset containing these DNA fragments was formatted and uploaded as a target database in the Mascot search engine. Peptide masses were searched against 6-frame translations of these fragments to allow an unbiased identification of ORFs. Peptide masses were also matched against open reading frames for the UNSWCD and BAA-1457 strains.

### Syntenic associations using STRING database and GEOMI visualization

The STRING database contains predicted functional relationships between proteins based on various criteria including conserved neighborhood association across multiple species [43]. Datasets for such synteny based associations were downloaded for *C. concisus* and were parsed in formats compatible for further processing. Very high scores (>0.9 of a possible maximum of 1.0) representing good syntenic association were considered as cutoffs. The 3-D network visualization platform GEOMI was then used to develop synteny networks for proteins in *C. concisus* [42]. A custom plug-in was developed for GEOMI to color-code functional associations for proteins deleted in the assembled UNSWCD genome.

### Validations of syntenic associations

Bacterial DNA was extracted using the Puregene Core kit A (Qiagen) according to the manufacturer's instructions. The presence of DNA was confirmed by amplifying the 16S rRNA gene sequence using the universal primer pair F27 and R1494, which amplifies a region of approximately 1460 bp [49]. The confirmation of the presence/absence of gene clusters that were shown to be present in *C. concisus* BAA-1457 but absent in UNSWCD by next-generation sequencing was performed using PCR. Primer pairs were designed to amplify regions (978, 1123, 1330 and 1782 bp) within several ORF clusters found in BAA-1457 (Table S4). The thermal cycling conditions for all reactions were: 94°C for 4 min, 30 cycles of 94°C for 20 s, 57°C for 20 s, and 72°C for 90 s, followed by 72°C for 5 min. PCR products were electrophoresed through 1.5% agarose gels at 100 V for 20 min. The products were then purified using the QIAquick® PCR Purification Kit (Qiagen) according to manufacturer's instructions. Sequencing of the positive PCR products was undertaken using the BigDye™ terminator chemistry (Applied Biosystems; Foster City, USA).

## Supporting Information

Figure S1 **Comparative view of the contigs assembled using single-end read data (lane 1) and paired-end read data (lane 2) for the UNSWCD sample, mapped against the** ***C. concisus*** **reference genome.** Lane 3 shows the genes in reference strain. Contigs produced from the paired-end assembly (lane 2) show higher coverage and merger of contig fragments when compared to lane 1.
(TIFF)

Figure S2 **A cluster of genes involved in restriction are completely/partially deleted in UNSWCD genome.** While proteins encoded by genes CCC13826_1410 and CCC13826_1412 have missing functional domains (related to restriction activity), the gene CCC13826_1411 is completely absent in the UNSWCD genome.
(TIFF)

Figure S3 **Sequencing, assembly and analysis of** ***C. concisus*** **UNSWCD strain.**
(TIFF)

Table S1 Different parameters such as overlap lengths, size of the contigs, N50 values were considered for the *de novo* assembly algorithms velvet and Edena.
(DOC)

Table S2 Proteins representing 217 of the possible 494 genes encoding hypothetical proteins in *C. concisus* UNSWCD draft genome have been identified using Orbitrap MS analysis.
(DOC)

Table S3 49 proteins encoded from the 138 genes specific to *C. concisus* UNSWCD (absent in the BAA-1457 reference genome) were identified using Orbitrap MS analysis of the UNSWCD strain. The complete list of identifications of UNSWCD proteins contained 1369 proteins.
(DOC)

Table S4 Primer sequences used for PCR confirmation of missing clusters.
(DOC)

Table S5 Ortholog IDs across UNSWCD and BAA-1457.
(DOC)
