# Supplementary figures and images for: Sequencing and Validation of the Genome of a Campylobacter concisus Reveals Intra-Species Diversity
Source: PLoS One. 2011 Jul 29;6(7):e22170. doi: 10.1371/journal.pone.0022170 (PMC3146479; doi:10.1371/journal.pone.0022170)

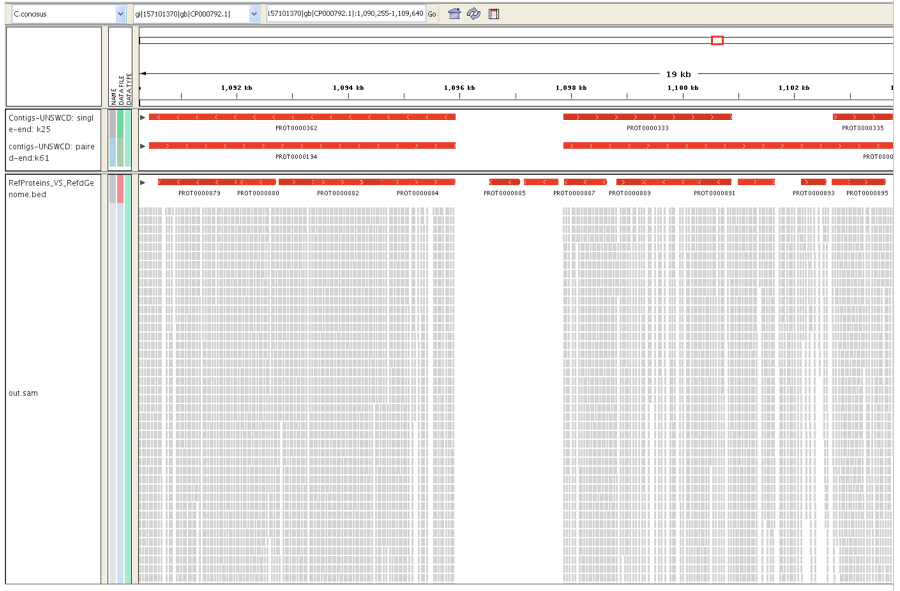

Supplement: Figure S1 — Comparative view of the contigs assembled using single-end read data (lane 1) and paired-end read data (lane 2) for the UNSWCD sample, mapped against the C. concisus reference genome. Lane 3 shows the genes in reference strain. Contigs produced from the paired-end assembly (lane 2) show higher coverage and merger of contig fragments when compared to lane 1. (TIFF) [file pone.0022170.s001.tiff]

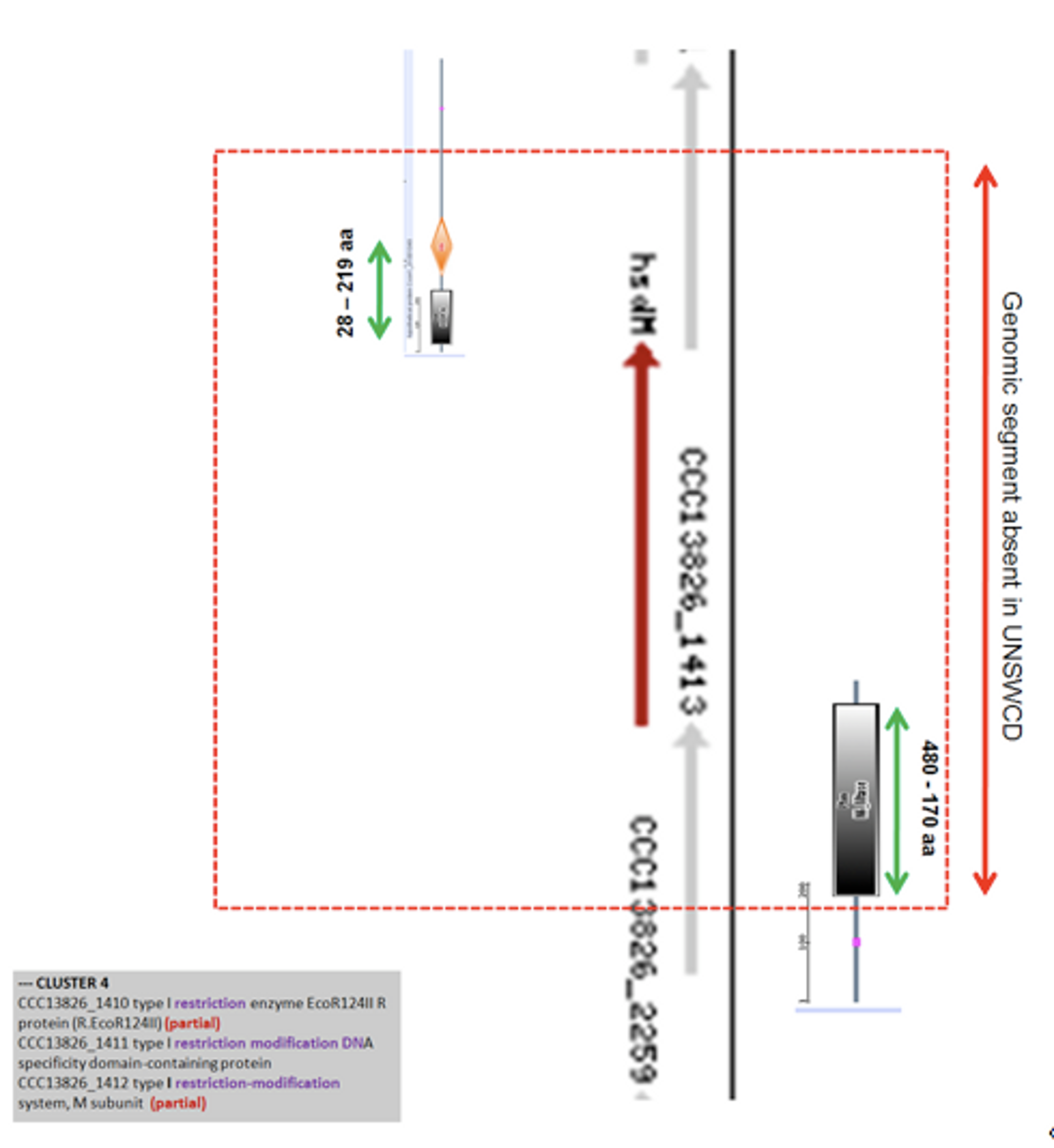

Supplement: Figure S2 — A cluster of genes involved in restriction are completely/partially deleted in UNSWCD genome. While proteins encoded by genes CCC13826_1410 and CCC13826_1412 have missing functional domains (related to restriction activity), the gene CCC13826_1411 is completely absent in the UNSWCD genome. (TIFF) [file pone.0022170.s002.tiff]

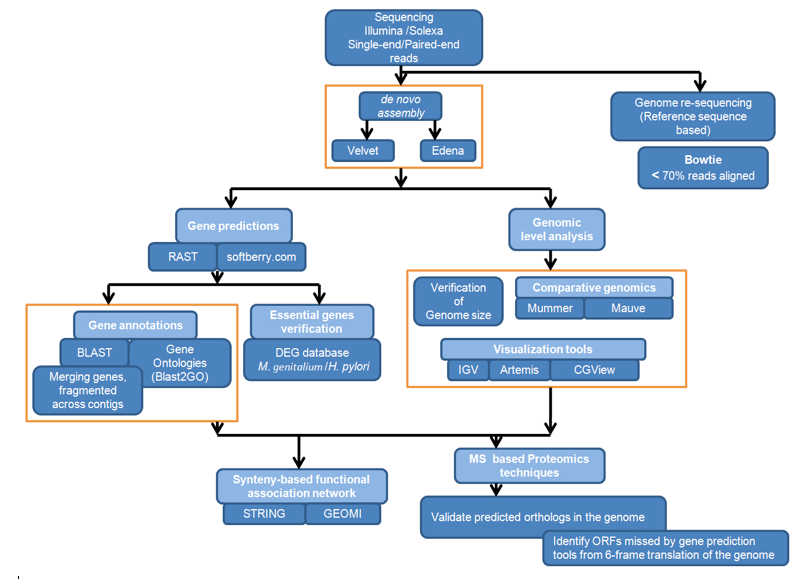

Supplement: Figure S3 — Sequencing, assembly and analysis of C. concisus UNSWCD strain. (TIFF) [file pone.0022170.s003.tiff]
