# Supplementary material for: Sequencing and Validation of the Genome of a Campylobacter concisus Reveals Intra-Species Diversity
Source: PLoS One. 2011 Jul 29;6(7):e22170. doi: 10.1371/journal.pone.0022170 (PMC3146479; doi:10.1371/journal.pone.0022170)
Supplement: Table S1 — Different parameters such as overlap lengths, size of the contigs, N50 values were considered for the de novo assembly algorithms velvet and Edena. (DOC) [file pone.0022170.s004.doc]

| **Single end data** | | | | | | | | | | | | | |
| --- | --- | --- | --- | --- | --- | --- | --- | --- | --- | --- | --- | --- | --- |
| **Velvet assembly** | | | | | | | **Edena assembly** | | | | | | |
| **k-mer** | **Nodes (Contigs)** | | **N50** | **Max length** | | **Assembled genome size** | **k-mer** | | **Nodes (Contigs)**  **>100 bp** | **N50** | | **Max length** | **Assembled genome size** |
|  |  | |  |  | |  | 21 | | 1444 | 2,191 | | 11711 | 1,776,886 |
| 23 | 457 | | 15,107 | 87,675 | | 1,671,843 | 23 | | 621 | 6,360 | | 27462 | 1,795,822 |
| **25** | **352** | | **21,868** | **102,216** | | **1,684,142** | **25** | | **459** | **11,694** | | **53090** | **1,796,970** |
| 27 | 352 | | 20,002 | 74,095 | | 1,681,690 | 27 | | 516 | 11,174 | | 34983 | 1,796,229 |
| 29 | 318 | | 17,806 | 37,344 | | 1,666,745 | 29 | | 636 | 7,854 | | 36898 | 1,788,951 |
| **Paired-end data :** Velvet assembly | | | | | | | | | | | | | |
| **k-mer** | | **Nodes (Contigs)** | | | **N50** | | | **Max length** | | | **Assembled genome size** | | |
| 45 | | 235 | | | 45,324 | | | 110,841 | | | 1,804,789 | | |
| 51 | | 174 | | | 48,556 | | | 177,467 | | | 1,805,532 | | |
| **61** | | **123** | | | **63,987** | | | **129,427** | | | **1,805,982** | | |
| 71 | | 135 | | | 50,000 | | | 134,870 | | | 1,802,913 | | |
| 73 | | 172 | | | 45,145 | | | 124,532 | | | 1,800,012 | | |
