# Supplementary material for: Sequencing and Validation of the Genome of a Campylobacter concisus Reveals Intra-Species Diversity
Source: PLoS One. 2011 Jul 29;6(7):e22170. doi: 10.1371/journal.pone.0022170 (PMC3146479; doi:10.1371/journal.pone.0022170)
Supplement: Table S2 — Proteins representing 217 of the possible 494 genes encoding hypothetical proteins in C. concisus UNSWCD draft genome have been identified using Orbitrap MS analysis. (DOC) [file pone.0022170.s005.doc]

| **Protein ID** | **Description** | **Peptides** | **Score** | **Mapping** | **Combined score** | |
| --- | --- | --- | --- | --- | --- | --- |
|  |  |  |  |  |  | |
| NODE_34_fig|6666666.462.peg.1036 | hypothetical protein | K.SQALNVIK.Y | 29 | 37-44 | 101 | |
|  |  | K.LSVKDSQK.A | 35 | 75-82 |  | |
|  |  | K.SELMSLGLNK.S | 35 | 27-36 |  | |
|  |  | K.SELMSLGLNK.S | 30 | 27-36 |  | |
|  |  | K.VQGIGFNDMQK.V | 26 | 60-70 |  | |
|  |  |  |  |  |  | |
|  |  |  |  |  |  | |
| NODE_18_fig|6666666.462.peg.496 | hypothetical protein | K.VIAGIQK.D | 23 | 356-362 | 2134 | |
|  |  | K.ILSESMK.N | 21 | 476-482 |  | |
|  |  | K.VILTDVR.K | 29 | 644-650 |  | |
|  |  | K.HTMPILK.A | 31 | 275-281 |  | |
|  |  | K.LSDKILDIGK.T | 47 | 180-189 |  | |
|  |  | K.LSDKILDIGK.T | 26 | 180-189 |  | |
|  |  | K.NSKNQDVTAK.I | 41 | 243-252 |  | |
|  |  | K.NKEIYTEPR.V | 38 | 138-146 |  | |
|  |  | K.EGGYAYIMKK.D | 31 | 192-201 |  | |
|  |  | R.TTVADKYDPR.T | 21 | 117-126 |  | |
|  |  | K.NIDKVIAGIQK.D | 20 | 352-362 |  | |
|  |  | R.SLKPIGVIQEK.L | 25 | 305-315 |  | |
|  |  | R.SLKPIGVIQEK.L | 51 | 305-315 |  | |
|  |  | K.LSENPNNPSLIK.L | 52 | 386-397 |  | |
|  |  | K.FITYESSTAPSK.L | 65 | 322-333 |  | |
|  |  | K.IEIDENIEADLK.A | 54 | 432-443 |  | |
|  |  | K.NLGNVINILNAYSK.N | 54 | 412-425 |  | |
|  |  | K.NLGNVINILNAYSK.N | 23 | 412-425 |  | |
|  |  | K.ILSESMKNLTSGANK.Q | 39 | 476-490 |  | |
|  |  | K.VVELVSQKEDQILK.D | 68 | 36-49 |  | |
|  |  | K.YANKEFDENGLISYK.N | 64 | 228-242 |  | |
|  |  | R.VTSTNDLVITFTAPVIK.N | 80 | 147-163 |  | |
|  |  | K.DNTMLEELNIAANNMIR.G | 51 | 363-379 |  | |
|  |  | K.DNTMLEELNIAANNMIR.G | 59 | 363-379 |  | |
|  |  | R.DIADQTNLLALNAAIEAAR.A | 101 | 535-553 |  | |
|  |  | R.DIADQTNLLALNAAIEAAR.A | 41 | 535-553 |  | |
|  |  | K.ENISVVNTTNDVTSEIDGMAK.V | 114 | 623-643 |  | |
|  |  | K.ENISVVNTTNDVTSEIDGMAK.V | 36 | 623-643 |  | |
|  |  |  |  |  |  | |
|  |  |  |  |  |  | |
| NODE_6_fig|6666666.462.peg.1599 | hypothetical protein | K.LAGDEKALR.L | 28 | 44-52 | 152 | |
|  |  | R.LYLDELYAK.E | 36 | 31-39 |  | |
|  |  | R.LYLDELYAKESK.K | 49 | 31-42 |  | |
|  |  | R.QIGQEAMIMQNNDEK.F | 64 | 65-79 |  | |
|  |  |  |  |  |  | |
|  |  |  |  |  |  | |
| NODE_6_fig|6666666.462.peg.1597 | hypothetical protein | R.TSEEVAK.I | 20 | 30-36 | 107 | |
|  |  | R.VIEHESAR.T | 47 | 22-29 |  | |
|  |  | R.SVVLNAKDYAR.I | 31 | 167-177 |  | |
|  |  | R.NEEIAFNAGLLDR.S | 48 | 154-166 |  | |
|  |  |  |  |  |  | |
|  |  |  |  |  |  | |
| NODE_6_fig|6666666.462.peg.1590 | hypothetical protein | R.LYAVANDGAK.I | 21 | 292-301 | 21 | |
|  |  |  |  |  |  | |
|  |  |  |  |  |  | |
| NODE_6_fig|6666666.462.peg.1591 | hypothetical protein | K.DASAFMQAQK.S | 34 | 44-53 | 34 | |
|  |  |  |  |  |  | |
|  |  |  |  |  |  | |
| NODE_26_fig|6666666.462.peg.748 | hypothetical protein | K.TIETEKTK.Q | 23 | 194-201 | 78 | |
|  |  | K.GSLTEALSSLVIK.G | 69 | 79-91 |  | |
|  |  |  |  |  |  | |
|  |  |  |  |  |  | |
| NODE_9_fig|6666666.462.peg.1855 | hypothetical protein | R.LDEMLTYQK.L | 43 | 211-219 | 64 | |
|  |  | R.LDEMLTYQK.L | 34 | 211-219 |  | |
|  |  |  |  |  |  | |
|  |  |  |  |  |  | |
| NODE_22_fig|6666666.462.peg.651 | hypothetical protein | K.IINFVSVK.E | 22 | 89-96 | 250 | |
|  |  | K.IINFVSVKE.- | 45 | 89-97 |  | |
|  |  | -.MVGFYVNVK.L | 34 | 1-9 |  | |
|  |  | K.AGCEAKFEEILK.E | 53 | 12-23 |  | |
|  |  | K.FEEILKEIVPASR.K | 52 | 18-30 |  | |
|  |  | K.DKGCISYECGVVVGAK.S | 78 | 32-47 |  | |
|  |  |  |  |  |  | |
|  |  |  |  |  |  | |
| NODE_19_fig|6666666.462.peg.539 | hypothetical protein | R.NVNEDFLLR.N | 24 | 82-90 | 200 | |
|  |  | R.VGSDEFIILSK.M | 71 | 424-434 |  | |
|  |  | K.ISMNIDYIQSTSEK.F | 74 | 612-625 |  | |
|  |  |  |  |  |  | |
|  |  |  |  |  |  | |
| NODE_19_fig|6666666.462.peg.538 | hypothetical protein | K.SNEEIKAEI.- | 52 | 54-62 | 81 | |
|  |  | K.DLVVCNVCGLK.S | 23 | 5-15 |  | |
|  |  |  |  |  |  | |
|  |  |  |  |  |  | |
| NODE_19_fig|6666666.462.peg.533 | hypothetical protein | K.FTSNTLGFGK.K | 48 | 120-129 | 74 | |
|  |  | K.IEGVQFQGVMR.E | 39 | 76-86 |  | |
|  |  |  |  |  |  | |
|  |  |  |  |  |  | |
| NODE_19_fig|6666666.462.peg.532 | hypothetical protein | M.AREAGVVK.S | 31 | 2-9 | 1644 | |
|  |  | R.AGDTVEIK.Y | 54 | 1551-1558 | | |
|  |  | K.VTLTDAFDK.S | 24 | 1495-1503 | | |
|  |  | R.QESVSNYKK.T | 26 | 578-586 | |  |
|  |  | R.TSPVEITLPK.D | 22 | 470-479 | |  |
|  |  | K.AVAVDQNGNVR.E | 52 | 15-25 | |  |
|  |  | K.VNFIEDDASR.D | 49 | 1243-1252 | | |
|  |  | K.GYSSVDQVEGK.T | 44 | 1741-1751 | | |
|  |  | K.GAVDEHGNSTTK.V | 45 | 1710-1721 | | |
|  |  | K.DIKTLEESGEK.T | 30 | 1527-1537 | | |
|  |  | K.DGNLKETTIAVK.V | 23 | 879-890 | |  |
|  |  | K.TLEESGEKTTTAK.F | 67 | 1530-1542 | | |
|  |  | R.FVEDGDGNVSLTR.A | 77 | 861-873 | |  |
|  |  | K.ATGKNTETYTVVK.D | 42 | 1162-1174 | | |
|  |  | K.NLKNGEDTIITAK.V | 47 | 1075-1087 | | |
|  |  | K.NLKNGEDTIITAK.V | 58 | 1075-1087 | | |
|  |  | R.VEHGNDTNINTSK.V | 45 | 232-244 | |  |
|  |  | K.TYDGVEIDLGDQK.V | 58 | 587-599 | |  |
|  |  | K.IKGAVDEHGNSTTK.V | 42 | 1708-1721 | | |
|  |  | R.SSAGYDLKVEATLK.T | 65 | 1595-1608 | | |
|  |  | K.DVTDPAHPFTYPK.Q | 33 | 1459-1471 | | |
|  |  | K.FINDINGNHTLSR.V | 50 | 219-231 | |  |
|  |  | K.AVAVDQNGNVRELK.V | 43 | 15-28 | |  |
|  |  | K.EDGTSLDVVNGNAVK.Y | 67 | 1056-1070 | | |
|  |  | K.SAESAPDSVTPQTIK.M | 61 | 547-561 | |  |
|  |  | K.AIFDITDDLNTVLK.I | 39 | 1694-1707 | | |
|  |  | R.EQAISDGDLNSTTVTIK.L | 105 | 740-756 | |  |
|  |  | K.LTGITNNDNTSDHVTIK.N | 64 | 793-809 | |  |
|  |  | K.SDVATDNVTPDKNPTIR.F | 21 | 429-445 | |  |
|  |  | K.DGSGNISLKDAANNTITLSGNK.L | 51 | 1175-1196 | | |
|  |  | K.DGSGNISLKDAANNTITLSGNK.L | 34 | 1175-1196 | | |
|  |  | K.TINVTAVTTDANGNGKAEATNK.N | 51 | 954-975 | |  |
|  |  | K.TYDGVEIDLGDQKVNHTTAK.I | 51 | 587-606 | |  |
|  |  | K.LEETAAGGNAAAAGGGDGVSLSDAR.F | 110 | 97-121 | |  |
|  |  | K.ITNAAGYVSPTSPEAMSATSPEAK.A | 33 | 1354-1377 | | |
|  |  | K.ITNAAGYVSPTSPEAMSATSPEAK.A | 85 | 1354-1377 | | |
|  |  | K.ITNAAGYVSPTSPEAMSATSPEAK.A | 71 | 1354-1377 | | |
|  |  | K.ITNAAGYVSPTSPEAMSATSPEAK.A | 59 | 1354-1377 | | |
|  |  | K.VEASVTNFQGNTSGSSEDHVTPTK.S | 39 | 302-325 | |  |
|  |  | K.VTGAAGDVSETKSDIILDTGNGSGTR.F | 41 | 1088-1113 | | |
|  |  |  |  |  | |  |
|  |  |  |  |  | |  |
| NODE_2_fig|6666666.462.peg.899 | hypothetical protein | -.MIQVYK.L | 23 | 1-6 | | 116 |
|  |  | K.FKIGNLSK.Y | 26 | 66-73 | |  |
|  |  | R.HMDDNEKLPR.E | 53 | 11-20 | |  |
|  |  | K.ILELSDEEFDEMIK.N | 53 | 45-58 | |  |
|  |  |  |  |  | |  |
|  |  |  |  |  | |  |
| NODE_34_fig|6666666.462.peg.1040 | hypothetical protein | R.YSHAVHGK.K | 26 | 216-223 | | 290 |
|  |  | K.NSPVGLALVSK.I | 32 | 103-113 | |  |
|  |  | K.SILNDIIATR.G | 62 | 192-201 | |  |
|  |  | K.SDIYSDHFALK.F | 63 | 266-276 | |  |
|  |  | K.LEGGYLHTPHGK.I | 23 | 416-427 | |  |
|  |  | K.NSIGVGDELDILIR.R | 70 | 352-365 | |  |
|  |  | K.IKPSGSEIFEVPNVK.T | 41 | 114-128 | |  |
|  |  | K.IKPSGSEIFEVPNVK.T | 29 | 114-128 | |  |
|  |  |  |  |  | |  |
|  |  |  |  |  | |  |
| NODE_54_fig|6666666.462.peg.1391 | hypothetical protein | K.ANELYAK.K | 42 | 25-31 | | 255 |
|  |  | K.LGEMTAYGLIK.D | 26 | 92-102 | |  |
|  |  | K.NFNEAYLAFNK.A | 61 | 33-43 | |  |
|  |  | K.MCDENEGMACEK.L | 64 | 80-91 | |  |
|  |  | K.MCDENEGMACEK.L | 55 | 80-91 | |  |
|  |  | K.ACDNGYKPACDFVTK.- | 23 | 122-136 | |  |
|  |  | K.ACDNGYKPACDFVTK.- | 23 | 122-136 | |  |
|  |  | K.ACTMNAIMLFNGDGVAK.D | 53 | 52-68 | |  |
|  |  |  |  |  | |  |
|  |  |  |  |  | |  |
| NODE_34_fig|6666666.462.peg.1048 | hypothetical protein | K.FDEMMR.Q | 28 | 173-178 | | 116 |
|  |  | R.EHLYQDK.D | 27 | 190-196 | |  |
|  |  | R.QVQYLDAYSAR.E | 52 | 179-189 | |  |
|  |  | R.GFELNYEDGEYIVR.V | 49 | 64-77 | |  |
|  |  |  |  |  | |  |
|  |  |  |  |  | |  |
| NODE_18_fig|6666666.462.peg.480 | hypothetical protein | K.SVVEQIYSGEAAK.F | 59 | 84-96 | | 59 |
|  |  |  |  |  | |  |
|  |  |  |  |  | |  |
| NODE_27_fig|6666666.462.peg.840 | hypothetical protein | K.SFKVPPK.I | 23 | 58-64 | | 223 |
|  |  | K.LLAEFYDLK.S | 49 | 169-177 | |  |
|  |  | R.YEAITNDIFAK.D | 57 | 149-159 | |  |
|  |  | K.TNCEVISLATNK.K | 76 | 212-223 | |  |
|  |  | K.TNCEVISLATNKK.L | 21 | 212-224 | |  |
|  |  | K.TNCEVISLATNKK.L | 61 | 212-224 | |  |
|  |  |  |  |  | |  |
|  |  |  |  |  | |  |
| NODE_14_fig|6666666.462.peg.248 | hypothetical protein | K.NALVISK.F | 30 | 345-351 | | 506 |
|  |  | K.LKNPLAK.E | 22 | 372-378 | |  |
|  |  | K.ISFYSSLK.D | 26 | 189-196 | |  |
|  |  | K.TILTLNKDK.T | 27 | 75-83 | |  |
|  |  | K.SKEEKPLLR.D | 23 | 33-41 | |  |
|  |  | K.KENSYIVTSK.N | 65 | 295-304 | |  |
|  |  | K.NSLLQLDDKK.N | 27 | 129-138 | |  |
|  |  | K.LTNESANADVMK.F | 56 | 270-281 | |  |
|  |  | K.LTNESANADVMK.F | 86 | 270-281 | |  |
|  |  | K.TANDIFIIANDK.I | 52 | 230-241 | |  |
|  |  | K.NENLKPQNQEQTQK.S | 58 | 19-32 | |  |
|  |  | K.NENLKPQNQEQTQK.S | 25 | 19-32 | |  |
|  |  | R.VGVLAQIYNYEPVNR.A | 42 | 142-156 | |  |
|  |  | K.TANDIFIIANDKICK.N | 25 | 230-244 | |  |
|  |  |  |  |  | |  |
|  |  |  |  |  | |  |
| NODE_72_fig|6666666.462.peg.1637 | hypothetical protein | K.NAISAKFENSR.I | 34 | 36-46 | | 34 |
|  |  |  |  |  | |  |
|  |  |  |  |  | |  |
| NODE_72_fig|6666666.462.peg.1636 | hypothetical protein | K.TKSELVNLK.L | 24 | 19-27 | | 24 |
|  |  |  |  |  | |  |
|  |  |  |  |  | |  |
| NODE_72_fig|6666666.462.peg.1631 | hypothetical protein | R.AQYENEFNITGSK.T | 49 | 137-149 | | 49 |
|  |  |  |  |  | |  |
|  |  |  |  |  | |  |
| NODE_72_fig|6666666.462.peg.1632 | hypothetical protein | K.FNELFK.T | 31 | 199-204 | | 206 |
|  |  | K.LMENFTTLR.T | 51 | 221-229 | |  |
|  |  | K.TYTTDQIAAR.Y | 39 | 205-214 | |  |
|  |  | R.SDGEQATMDNPK.F | 56 | 187-198 | |  |
|  |  | R.TSDLRSDGEQATMDNPK.F | 41 | 182-198 | |  |
|  |  | R.TSDLRSDGEQATMDNPK.F | 46 | 182-198 | |  |
|  |  | R.TSDLRSDGEQATMDNPK.F | 21 | 182-198 | |  |
|  |  |  |  |  | |  |
|  |  |  |  |  | |  |
| NODE_19_fig|6666666.462.peg.521 | hypothetical protein | K.IISMLNIPK.N | 50 | 67-75 | | 145 |
|  |  | R.FSQNGIGVKK.D | 30 | 39-48 | |  |
|  |  | K.VGILEDGVYR.F | 53 | 29-38 | |  |
|  |  | K.VISVQNADNSR.K | 49 | 54-64 | |  |
|  |  |  |  |  | |  |
|  |  |  |  |  | |  |
| NODE_51_fig|6666666.462.peg.1333 | hypothetical protein | K.NSTITISSR.L | 46 | 322-330 | | 347 |
|  |  | R.LDESSYLVLTK.E | 56 | 89-99 | |  |
|  |  | K.IKDNSVTTLTTR.L | 63 | 77-88 | |  |
|  |  | K.NNPQNFNPNNLK.T | 56 | 35-46 | |  |
|  |  | R.ENTNGAVASLVLNLKG.- | 63 | 390-405 | |  |
|  |  | K.SLPDEFLPLGNSINR.L | 42 | 163-177 | |  |
|  |  |  |  |  | |  |
|  |  |  |  |  | |  |
| NODE_51_fig|6666666.462.peg.1337 | conserved hypothetical protein-putative ATP-bindingprotein | K.TPMLTLLK.Q | 47 | 339-346 | | 478 |
|  |  | K.ENLSSQGVDK.S | 24 | 459-468 | |  |
|  |  | K.SLTPNLLASAK.V | 22 | 492-502 | |  |
|  |  | K.INALIASPSFK.G | 32 | 246-256 | |  |
|  |  | K.ASLENLISNLAK.R | 34 | 405-416 | |  |
|  |  | K.EQLLISTIFESK.F | 50 | 190-201 | |  |
|  |  | K.NINEDGLNVISTR.W | 68 | 423-435 | |  |
|  |  | K.ENLSSQGVDKSEMK.F | 51 | 459-472 | |  |
|  |  | K.IGVFDVDTFYPSSER.L | 42 | 225-239 | |  |
|  |  | R.LQDKCNEILEEQTAK.L | 68 | 607-621 | |  |
|  |  | R.VDASENEPSGASFAAVPNFITSAK.N | 48 | 542-565 | |  |
|  |  |  |  |  | |  |
|  |  |  |  |  | |  |
| NODE_3_fig|6666666.462.peg.1152 | hypothetical protein | K.QNNINSVLYSNASGK.L | 80 | 115-129 | | 80 |
|  |  |  |  |  | |  |
|  |  |  |  |  | |  |
| NODE_23_fig|6666666.462.peg.699 | hypothetical protein | R.FIEDILSK.E | 24 | 291-298 | | 41 |
|  |  | R.FIEDILSKEGSK.Y | 30 | 291-302 | |  |
|  |  |  |  |  | |  |
|  |  |  |  |  | |  |
| NODE_23_fig|6666666.462.peg.698 | hypothetical protein | R.IIDLCQYASEPLLSR.L | 56 | 107-121 | | 64 |
|  |  |  |  |  | |  |
|  |  |  |  |  | |  |
| NODE_23_fig|6666666.462.peg.697 | hypothetical protein | K.ILEIIIPTNAGK.Y | 27 | 134-145 | | 140 |
|  |  | K.VELENDHAAQDAFGVANGVK.M | 43 | 25-44 | |  |
|  |  | K.VELENDHAAQDAFGVANGVK.M | 95 | 25-44 | |  |
|  |  |  |  |  | |  |
|  |  |  |  |  | |  |
| NODE_23_fig|6666666.462.peg.696 | hypothetical protein | K.AAQLYKK.V | 24 | 326-332 | | 130 |
|  |  | K.DLDKAAQLYQK.A | 39 | 178-188 | |  |
|  |  | K.DPNKAAQFYQK.A | 28 | 142-152 | |  |
|  |  | K.AKKPTSLTELEK.C | 28 | 105-116 | |  |
|  |  | K.DMAACNNLGNAYSK.G | 63 | 122-135 | |  |
|  |  |  |  |  | |  |
|  |  |  |  |  | |  |
| NODE_22_fig|6666666.462.peg.631 | hypothetical protein | R.SLIEKNK.H | 22 | 53-59 | | 151 |
|  |  | K.NKHNIEK.N | 21 | 58-64 | |  |
|  |  | K.MIEQNSDGIK.E | 21 | 33-42 | |  |
|  |  | K.MIEQNSDGIK.E | 44 | 33-42 | |  |
|  |  | K.NTKDITTNVEK.I | 22 | 65-75 | |  |
|  |  | K.MIEQNSDGIKENR.D | 42 | 33-45 | |  |
|  |  | K.DITTNVEKIDIDLED.- | 58 | 68-82 | |  |
|  |  |  |  |  | |  |
|  |  |  |  |  | |  |
| NODE_22_fig|6666666.462.peg.634 | hypothetical protein | R.NFSEYQISR.A | 22 | 10-18 | | 22 |
|  |  |  |  |  | |  |
|  |  |  |  |  | |  |
| NODE_7_fig|6666666.462.peg.1688 | hypothetical protein | R.VLNIATFLK.R | 21 | 105-113 | | 21 |
|  |  |  |  |  | |  |
|  |  |  |  |  | |  |
| NODE_45_fig|6666666.462.peg.1287 | hypothetical protein | K.GTVALLGAK.N | 61 | 214-222 | | 585 |
|  |  | K.FNDLVAGK.S | 32 | 3-10 | |  |
|  |  | K.DGEILNLK.A | 48 | 189-196 | |  |
|  |  | K.AKDFSVSAK.F | 50 | 197-205 | |  |
|  |  | K.NFDGYAFK.Q | 28 | 223-230 | |  |
|  |  | K.NVVLAGSEVR.E | 54 | 134-143 | |  |
|  |  | -.MKFNDLVAGK.S | 21 | 1-10 | |  |
|  |  | -.MKFNDLVAGK.S | 40 | 1-10 | |  |
|  |  | -.MKFNDLVAGK.S | 53 | 1-10 | |  |
|  |  | K.SLSIANLLSLSDK.N | 63 | 11-23 | |  |
|  |  | K.YISCFEDNELGSK.N | 67 | 36-48 | |  |
|  |  | K.NASDEIVGGVQFGIVAK.A | 83 | 170-186 | |  |
|  |  | K.SLSIANLLSLSDKNLAK.K | 64 | 11-27 | |  |
|  |  |  |  |  | |  |
|  |  |  |  |  | |  |
| NODE_7_fig|6666666.462.peg.1668 | hypothetical protein | K.AYNVNFNV.- | 33 | 72-79 | | 75 |
|  |  | K.NGTDVFDFSK.V | 38 | 33-42 | |  |
|  |  | K.EADLKEIYAK.F | 31 | 45-54 | |  |
|  |  |  |  |  | |  |
|  |  |  |  |  | |  |
| NODE_23_fig|6666666.462.peg.680 | hypothetical protein | R.FESEAK.A | 24 | 45-50 | | 320 |
|  |  | K.ANEWLEK.N | 37 | 51-57 | |  |
|  |  | K.IDSVQDGIR.I | 45 | 21-29 | |  |
|  |  | K.SQRFESEAK.A | 31 | 42-50 | |  |
|  |  | K.SQRFESEAK.A | 35 | 42-50 | |  |
|  |  | R.FYEGANQVAK.S | 24 | 32-41 | |  |
|  |  | K.LNDRTYLNK.D | 27 | 5-13 | |  |
|  |  | R.IKIDSVQDGIR.I | 46 | 19-29 | |  |
|  |  | R.IRFYEGANQVAK.S | 60 | 30-41 | |  |
|  |  | K.ANEWLEKNFLNK.- | 24 | 51-62 | |  |
|  |  | R.FESEAKANEWLEK.N | 50 | 45-57 | |  |
|  |  | R.FESEAKANEWLEK.N | 22 | 45-57 | |  |
|  |  |  |  |  | |  |
|  |  |  |  |  | |  |
| NODE_22_fig|6666666.462.peg.606 | hypothetical protein | K.NSSDKVLISK.E | 38 | 177-186 | | 50 |
|  |  | K.NSDKNGNEITPENVK.N | 25 | 129-143 | |  |
|  |  |  |  |  | |  |
|  |  |  |  |  | |  |
| NODE_27_fig|6666666.462.peg.780 | hypothetical protein | K.FNDANQTLR.Q | 39 | 124-132 | | 88 |
|  |  | K.LINEIIDVTTHR.K | 62 | 68-79 | |  |
|  |  |  |  |  | |  |
|  |  |  |  |  | |  |
| NODE_12_fig|6666666.462.peg.152 | hypothetical protein | K.EVVEDR.A | 26 | 91-96 | | 607 |
|  |  | R.EGGLKSR.S | 30 | 143-149 | |  |
|  |  | R.DIDEIVR.R | 29 | 39-45 | |  |
|  |  | -.MKTAQQR.K | 26 | 1-7 | |  |
|  |  | R.SMDEILSK.L | 54 | 154-161 | |  |
|  |  | R.DIDEIVRR.A | 22 | 39-46 | |  |
|  |  | K.EGVAVLESLPLR.Y | 52 | 18-29 | |  |
|  |  | K.AAGLDEEVVMER.R | 59 | 182-193 | |  |
|  |  | K.SFLGDAYEGLSVK.E | 80 | 76-88 | |  |
|  |  | K.KEGVAVLESLPLR.Y | 78 | 17-29 | |  |
|  |  | K.KEGVAVLESLPLR.Y | 27 | 17-29 | |  |
|  |  | K.AAGLDEEVVMERR.A | 42 | 182-194 | |  |
|  |  | K.SFLGDAYEGLSVKEK.E | 96 | 76-90 | |  |
|  |  |  |  |  | |  |
|  |  |  |  |  | |  |
| NODE_7_fig|6666666.462.peg.1696 | hypothetical protein | K.TNLASEAFTMLSK.V | 76 | 253-265 | | 76 |
|  |  |  |  |  | |  |
|  |  |  |  |  | |  |
| NODE_7_fig|6666666.462.peg.1695 | hypothetical protein | R.IGYKIDAQEL.- | 26 | 213-222 | | 96 |
|  |  | R.NKTNNNFIISK.R | 51 | 200-210 | |  |
|  |  | K.YYGTDDKNIVK.I | 45 | 119-129 | |  |
|  |  |  |  |  | |  |
|  |  |  |  |  | |  |
| NODE_7_fig|6666666.462.peg.1698 | hypothetical protein | K.NGEYYPPEIYK.N | 33 | 86-96 | | 33 |
|  |  |  |  |  | |  |
|  |  |  |  |  | |  |
| NODE_12_fig|6666666.462.peg.112 | hypothetical protein | K.TQEQVFELPISK.M | 57 | 29-40 | | 91 |
|  |  |  |  |  | |  |
|  |  |  |  |  | |  |
| NODE_45_fig|6666666.462.peg.1272 | hypothetical protein | K.EKEQMLIER.K | 33 | 96-104 | | 201 |
|  |  | K.LAVNEAAEILGITK.E | 43 | 4-17 | |  |
|  |  | K.LAVNEAAEILGITK.E | 101 | 4-17 | |  |
|  |  |  |  |  | |  |
|  |  |  |  |  | |  |
| NODE_45_fig|6666666.462.peg.1279 | hypothetical protein | K.GAANSDEEHLEK.I | 36 | 99-110 | | 36 |
|  |  |  |  |  | |  |
|  |  |  |  |  | |  |
| NODE_22_fig|6666666.462.peg.612 | hypothetical protein | K.IAELGGNVLNVSA.- | 47 | 183-195 | | 101 |
|  |  | -.MQISQIVSSYNTSSVK.E | 67 | 1-16 | |  |
|  |  |  |  |  | |  |
|  |  |  |  |  | |  |
| NODE_22_fig|6666666.462.peg.611 | hypothetical protein | K.SSIKGFIK.D | 22 | 42-49 | | 56 |
|  |  | K.TNIEVPKEEK.I | 35 | 133-142 | |  |
|  |  | R.KPQNEVVVKR.K | 25 | 119-128 | |  |
|  |  |  |  |  | |  |
|  |  |  |  |  | |  |
| NODE_27_fig|6666666.462.peg.793 | hypothetical protein | K.NHLLPK.E | 28 | 57-62 | | 39 |
|  |  | K.EVASDLHK.M | 24 | 63-70 | |  |
|  |  |  |  |  | |  |
|  |  |  |  |  | |  |
| NODE_27_fig|6666666.462.peg.795 | hypothetical protein | K.SLEINSF.- | 33 | 266-272 | | 106 |
|  |  | K.IYKDQR.C | 20 | 47-52 | |  |
|  |  | K.SSFYVANLNNEIVK.V | 61 | 58-71 | |  |
|  |  |  |  |  | |  |
|  |  |  |  |  | |  |
| NODE_27_fig|6666666.462.peg.798 | hypothetical protein | K.DFLNVIK.K | 24 | 400-406 | | 872 |
|  |  | R.GGEFSKER.M | 26 | 186-193 | |  |
|  |  | K.NMTYAAIR.G | 55 | 178-185 | |  |
|  |  | K.NMTYAAIR.G | 32 | 178-185 | |  |
|  |  | R.LNSQLLNLK.N | 42 | 251-259 | |  |
|  |  | -.MDKLEQNLR.D | 37 | 1-9 | |  |
|  |  | -.MDKLEQNLR.D | 20 | 1-9 | |  |
|  |  | -.MDKLEQNLR.D | 42 | 1-9 | |  |
|  |  | K.NVDEEFIALLK.E | 38 | 260-270 | |  |
|  |  | R.ISDQAMEQISSR.L | 67 | 302-313 | |  |
|  |  | R.DYKDSEGLLTSIK.A | 53 | 10-22 | |  |
|  |  | K.SAMEGINQALVSGEEK.G | 83 | 68-83 | |  |
|  |  | K.SAMEGINQALVSGEEK.G | 75 | 68-83 | |  |
|  |  | K.NIISAAGNLANEGHIFAK.E | 80 | 200-217 | |  |
|  |  | K.SEIESVINSELDTNLAK.F | 85 | 282-298 | |  |
|  |  | K.FAPDELRLNSQLLNLK.N | 39 | 244-259 | |  |
|  |  | R.ISDQAMEQISSRLEELK.S | 27 | 302-318 | |  |
|  |  |  |  |  | |  |
|  |  |  |  |  | |  |
| NODE_2_fig|6666666.462.peg.920 | hypothetical protein | R.RVDAVVR.D | 36 | 173-179 | | 729 |
|  |  | K.IAASLDEK.D | 35 | 198-205 | |  |
|  |  | K.ERPNAPVK.I | 37 | 190-197 | |  |
|  |  | K.SMELLISR.R | 32 | 165-172 | |  |
|  |  | K.SMELLISR.R | 38 | 165-172 | |  |
|  |  | K.EGKLEAISK.S | 34 | 233-241 | |  |
|  |  | K.SYFGKDVSK.- | 22 | 242-250 | |  |
|  |  | K.DADSATSNWAK.V | 47 | 137-147 | |  |
|  |  | K.KDADSATSNWAK.V | 47 | 136-147 | |  |
|  |  | K.NELVGYDVDIAR.A | 52 | 47-58 | |  |
|  |  | K.YGAELVVTDSYAK.S | 64 | 152-164 | |  |
|  |  | K.ADVVFNQVSITDER.K | 73 | 87-100 | |  |
|  |  | K.IAASLDEKDYTAAAVK.K | 64 | 198-213 | |  |
|  |  | K.IAASLDEKDYTAAAVK.K | 40 | 198-213 | |  |
|  |  | K.ADVVFNQVSITDERK.K | 52 | 87-101 | |  |
|  |  | K.VAVKYGAELVVTDSYAK.S | 21 | 148-164 | |  |
|  |  |  |  |  | |  |
|  |  |  |  |  | |  |
| NODE_2_fig|6666666.462.peg.925 | hypothetical protein | K.AVLHDLGAEK.I | 31 | 16-25 | | 61 |
|  |  | M.SVLVIGADEITPIK.A | 43 | 2-15 | |  |
|  |  |  |  |  | |  |
|  |  |  |  |  | |  |
| NODE_10_fig|6666666.462.peg.10 | hypothetical protein | R.SGSGFGKGK.M | 29 | 117-125 | | 1243 |
|  |  | K.LTSTIPVLR.L | 43 | 74-82 | |  |
|  |  | R.SGELRSGSGFGK.G | 37 | 112-123 | |  |
|  |  | R.SGELRSGSGFGK.G | 25 | 112-123 | |  |
|  |  | R.GAMMDNMMQER.A | 54 | 97-107 | |  |
|  |  | R.GAMMDNMMQER.A | 62 | 97-107 | |  |
|  |  | R.GAMMDNMMQER.A | 71 | 97-107 | |  |
|  |  | R.GAMMDNMMQER.A | 66 | 97-107 | |  |
|  |  | R.GAMMDNMMQER.A | 51 | 97-107 | |  |
|  |  | R.GAMMDNMMQER.A | 55 | 97-107 | |  |
|  |  | R.GAMMDNMMQER.A | 46 | 97-107 | |  |
|  |  | R.GAMMDNMMQER.A | 35 | 97-107 | |  |
|  |  | R.GAMMDNMMQER.A | 50 | 97-107 | |  |
|  |  | R.GAMMDNMMQER.A | 38 | 97-107 | |  |
|  |  | R.GAMMDNMMQER.A | 75 | 97-107 | |  |
|  |  | R.GAMMDNMMQER.A | 32 | 97-107 | |  |
|  |  | R.NYKLTSTIPVLR.L | 26 | 71-82 | |  |
|  |  | R.NYKLTSTIPVLR.L | 38 | 71-82 | |  |
|  |  | K.ETQTTLVLIENR.S | 57 | 170-181 | |  |
|  |  | K.ETQTTLVLIENR.S | 38 | 170-181 | |  |
|  |  | R.LLAQQTGCFVIVER.G | 73 | 83-96 | |  |
|  |  |  |  |  | |  |
|  |  |  |  |  | |  |
| NODE_51_fig|6666666.462.peg.1360 | hypothetical protein | K.ITQQLAK.S | 30 | 144-150 | | 660 |
|  |  | -.MVNAVESK.I | 25 | 1-8 | |  |
|  |  | K.KADFIFSK.Q | 26 | 179-186 | |  |
|  |  | R.NQMTMVADIAK.A | 59 | 79-89 | |  |
|  |  | K.QIEYETTSDDLAK.I | 67 | 187-199 | |  |
|  |  | K.SAYQDLTQGTSIIK.T | 68 | 216-229 | |  |
|  |  | K.WLPVAGAAAMAAWSK.I | 40 | 156-170 | |  |
|  |  | K.ISDGVAQMQLAADDLK.S | 29 | 200-215 | |  |
|  |  | K.ISDGVAQMQLAADDLK.S | 68 | 200-215 | |  |
|  |  | K.ITEGINSALTSVINSR.E | 94 | 9-24 | |  |
|  |  | K.ISDGVAQMQLAADDLK.S | 61 | 200-215 | |  |
|  |  |  |  |  | |  |
|  |  |  |  |  | |  |
| NODE_21_fig|6666666.462.peg.590 | hypothetical protein | K.IFIAGDR.A | 36 | 177-183 | | 951 |
|  |  | K.HLNPNIK.T | 25 | 471-477 | |  |
|  |  | K.EHALPSGK.F | 22 | 339-346 | |  |
|  |  | K.TQLLFLPK.F | 36 | 305-312 | |  |
|  |  | R.LLGLSSDFKK.A | 33 | 363-372 | |  |
|  |  | K.MKDDFGLDGVK.F | 40 | 396-406 | |  |
|  |  | K.MKDDFGLDGVK.F | 30 | 396-406 | |  |
|  |  | K.AILNESGALALSK.F | 55 | 105-117 | |  |
|  |  | R.ASAILPYSLFIK.R | 27 | 56-67 | |  |
|  |  | K.NVTLSGDLFYNK.V | 47 | 451-462 | |  |
|  |  | K.FDANFLMPVNLK.Q | 52 | 161-172 | |  |
|  |  | K.VSGSVEELSALADR.A | 119 | 42-55 | |  |
|  |  | K.TNFDPGFGVEIKL.- | 47 | 478-490 | |  |
|  |  | K.NLLLMSSDFSGQK.G | 79 | 376-388 | |  |
|  |  | K.NPAFLAPFLCSLAK.E | 29 | 12-25 | |  |
|  |  | K.EGFSFLSVSVNEQK.N | 38 | 235-248 | |  |
|  |  | K.FGTLSPSQAAAFLANGK.A | 58 | 88-104 | |  |
|  |  | R.SEVDVADELDVAGEIK.K | 82 | 69-84 | |  |
|  |  | R.AQIALASFEKPLLALK.T | 32 | 184-199 | |  |
|  |  | R.SEVDVADELDVAGEIKK.V | 104 | 69-85 | |  |
|  |  |  |  |  | |  |
|  |  |  |  |  | |  |
| NODE_16_fig|6666666.462.peg.376 | hypothetical protein | R.SGDTAMIR.G | 23 | 132-139 | | 79 |
|  |  | K.NLEVIENNGVVEPETK.I | 69 | 16-31 | |  |
|  |  |  |  |  | |  |
|  |  |  |  |  | |  |
| NODE_16_fig|6666666.462.peg.377 | hypothetical protein | K.LGLSDVR.Y | 26 | 64-70 | | 162 |
|  |  | R.YATNLSVSSEER.S | 67 | 71-82 | |  |
|  |  | K.EFSLDGMPEIQK.F | 47 | 125-136 | |  |
|  |  | K.ALNEPENIEQNVLNAPLMPK.L | 42 | 173-192 | |  |
|  |  |  |  |  | |  |
|  |  |  |  |  | |  |
| NODE_28_fig|6666666.462.peg.869 | hypothetical protein | K.LIEILPK.K | 22 | 16-22 | | 43 |
|  |  | K.FKDQLDAANK.L | 34 | 6-15 | |  |
|  |  |  |  |  | |  |
|  |  |  |  |  | |  |
| NODE_28_fig|6666666.462.peg.867 | hypothetical protein | K.LQNEEISLAPFAK.R | 35 | 9-21 | | 35 |
|  |  |  |  |  | |  |
|  |  |  |  |  | |  |
| NODE_28_fig|6666666.462.peg.863 | hypothetical protein | K.IADESFHLVK.G | 39 | 174-183 | | 77 |
|  |  | K.IADESFHLVK.G | 20 | 174-183 | |  |
|  |  | K.NENGSMSLLSFDK.D | 43 | 136-148 | |  |
|  |  |  |  |  | |  |
|  |  |  |  |  | |  |
| NODE_45_fig|6666666.462.peg.1240 | hypothetical protein | R.GEIYKQNSSINVLE.- | 30 | 135-148 | | 30 |
|  |  |  |  |  | |  |
|  |  |  |  |  | |  |
| NODE_75_fig|6666666.462.peg.1661 | hypothetical protein | K.NIDTHIEK.Q | 26 | 336-343 | | 283 |
|  |  | K.DKEGSEIENNTNLMK.Y | 80 | 130-144 | |  |
|  |  | R.IAQVKPSSIILGTSRPK.H | 30 | 58-74 | |  |
|  |  | K.TILGIGESTVLDNGNAIPLAK.V | 85 | 158-178 | |  |
|  |  |  |  |  | |  |
|  |  |  |  |  | |  |
| NODE_2_fig|6666666.462.peg.918 | hypothetical protein | K.EGVLSEISLK.Y | 27 | 242-251 | | 338 |
|  |  | K.DNSDIKSFADLK.G | 31 | 131-142 | |  |
|  |  | K.IIEAGDEPMYTAAIVK.K | 72 | 207-222 | |  |
|  |  |  |  |  | |  |
|  |  |  |  |  | |  |
| NODE_59_fig|6666666.462.peg.1412 | hypothetical protein | R.SLIVVPNK.E | 27 | 115-122 | | 140 |
|  |  | R.SVFADCQSFK.V | 45 | 180-189 | |  |
|  |  | R.VSDIDTVFWK.L | 51 | 210-219 | |  |
|  |  | K.TGEVEYYQLPFYTR.V | 37 | 196-209 | |  |
|  |  |  |  |  | |  |
|  |  |  |  |  | |  |
| NODE_59_fig|6666666.462.peg.1410 | hypothetical protein | K.NLDVIK.R | 21 | 25-30 | | 238 |
|  |  | K.NSSFASR.V | 34 | 8-14 | |  |
|  |  | K.NLDVIKR.N | 23 | 25-31 | |  |
|  |  | R.VDKFWCELGK.N | 37 | 15-24 | |  |
|  |  | R.NLEDKNYETASK.M | 66 | 32-43 | |  |
|  |  | K.SVDMCLYNETLSK.L | 68 | 136-148 | |  |
|  |  |  |  |  | |  |
|  |  |  |  |  | |  |
| NODE_12_fig|6666666.462.peg.51 | hypothetical protein | K.IDGINLAK.G | 45 | 277-284 | | 548 |
|  |  | K.SSFLGKER.I | 43 | 377-384 | |  |
|  |  | K.LNDDLTPAK.T | 48 | 321-329 | |  |
|  |  | K.TLNEIYADTK.N | 47 | 179-188 | |  |
|  |  | R.VQRDENEFAK.I | 25 | 264-273 | |  |
|  |  | R.ISEEGYEISVK.N | 67 | 385-395 | |  |
|  |  | K.IWKIDGINLAK.G | 29 | 274-284 | |  |
|  |  | K.ISEENSQSYIK.W | 73 | 68-78 | |  |
|  |  | R.ISEEGYEISVKNNSSK.S | 75 | 385-400 | |  |
|  |  | R.FIEQALIGENKSNDATK.R | 21 | 99-115 | |  |
|  |  | R.FIEQALIGENKSNDATK.R | 57 | 99-115 | |  |
|  |  | K.SVDVTLVERVPVSADEAVK.V | 38 | 401-419 | |  |
|  |  |  |  |  | |  |
|  |  |  |  |  | |  |
| NODE_45_fig|6666666.462.peg.1257 | hypothetical protein | K.APLSMVYECDR.S | 37 | 9-19 | | 44 |
|  |  |  |  |  | |  |
|  |  |  |  |  | |  |
| NODE_8_fig|6666666.462.peg.1779 | hypothetical protein | K.ENLLDPNSNDELK.I | 37 | 71-83 | | 172 |
|  |  | K.GLVGQNNDDSLENK.A | 82 | 142-155 | |  |
|  |  |  |  |  | |  |
|  |  |  |  |  | |  |
| NODE_18_fig|6666666.462.peg.418 | hypothetical protein | R.ADEALISQIVAISK.A | 57 | 8-21 | | 89 |
|  |  | K.LIIDDGYADELNAR.A | 45 | 31-44 | |  |
|  |  |  |  |  | |  |
|  |  |  |  |  | |  |
| NODE_75_fig|6666666.462.peg.1657 | hypothetical protein | -.MQINPNFINLNK.S | 39 | 1-12 | | 46 |
|  |  |  |  |  | |  |
|  |  |  |  |  | |  |
| NODE_23_fig|6666666.462.peg.676 | hypothetical protein | K.EASEINEICTK.F | 40 | 61-71 | | 93 |
|  |  | R.VIFYNSQICSK.S | 45 | 84-94 | |  |
|  |  |  |  |  | |  |
|  |  |  |  |  | |  |
| NODE_35_fig|6666666.462.peg.1084 | hypothetical protein | M.SYDLQEIILER.T | 52 | 2-12 | | 52 |
|  |  |  |  |  | |  |
|  |  |  |  |  | |  |
| NODE_35_fig|6666666.462.peg.1088 | hypothetical protein | K.VSLFQNTMK.I | 41 | 151-159 | | 90 |
|  |  |  |  |  | |  |
|  |  |  |  |  | |  |
| NODE_40_fig|6666666.462.peg.1160 | hypothetical protein | -.MMSNEEIAQK.F | 57 | 1-10 | | 210 |
|  |  | -.MMSNEEIAQK.F | 47 | 1-10 | |  |
|  |  | R.ALDQILEFKES.- | 42 | 130-140 | |  |
|  |  | K.ICPCAISDSMR.A | 25 | 119-129 | |  |
|  |  | K.FAEQNCAQMILAR.Y | 55 | 11-23 | |  |
|  |  | R.YAQHLGANEAQLMK.L | 37 | 24-37 | |  |
|  |  |  |  |  | |  |
|  |  |  |  |  | |  |
| NODE_41_fig|6666666.462.peg.1191 | hypothetical protein | R.AFEDTKR.A | 34 | 48-54 | | 128 |
|  |  | K.ESGTTTLASVLDDEVK.S | 64 | 66-81 | |  |
|  |  | K.ESGTTTLASVLDDEVKSK.L | 55 | 66-83 | |  |
|  |  |  |  |  | |  |
|  |  |  |  |  | |  |
| NODE_87_fig|6666666.462.peg.1768 | hypothetical protein | K.ANFIELNAK.M | 49 | 61-69 | | 147 |
|  |  | R.SLQDFYVLR.A | 45 | 128-136 | |  |
|  |  | K.DKLPANLSAYK.A | 20 | 50-60 | |  |
|  |  | K.YENELEKIEK.L | 38 | 28-37 | |  |
|  |  |  |  |  | |  |
|  |  |  |  |  | |  |
| NODE_51_fig|6666666.462.peg.1343 | hypothetical protein | K.IANKEINFELVR.N | 42 | 8-19 | | 42 |
|  |  |  |  |  | |  |
|  |  |  |  |  | |  |
| NODE_38_fig|6666666.462.peg.1121 | hypothetical protein | K.AQECVFK.A | 26 | 69-75 | | 139 |
|  |  | R.FEDVKAELDSQK.- | 62 | 95-106 | |  |
|  |  |  |  |  | |  |
|  |  |  |  |  | |  |
| NODE_38_fig|6666666.462.peg.1120 | hypothetical protein | -.MQENSLFTLSNTR.L | 86 | 1-13 | | 167 |
|  |  | -.MQENSLFTLSNTR.L | 35 | 1-13 | |  |
|  |  | -.MQENSLFTLSNTR.L | 71 | 1-13 | |  |
|  |  |  |  |  | |  |
|  |  |  |  |  | |  |
| NODE_45_fig|6666666.462.peg.1236 | hypothetical protein | K.ILEEAKEFTQK.S | 51 | 188-198 | | 68 |
|  |  | K.GVINVIEADHASINLNK.T | 21 | 82-98 | |  |
|  |  |  |  |  | |  |
|  |  |  |  |  | |  |
| NODE_34_fig|6666666.462.peg.966 | hypothetical protein | K.IYETEISK.K | 21 | 3-10 | | 56 |
|  |  | R.QIPEGLKAQTR.Q | 30 | 40-50 | |  |
|  |  | -.MKIYETEISK.K | 32 | 1-10 | |  |
|  |  |  |  |  | |  |
|  |  |  |  |  | |  |
| NODE_45_fig|6666666.462.peg.1233 | hypothetical protein | R.FDELTNLAK.N | 37 | 141-149 | | 63 |
|  |  | -.MHSIGIDIGSTSAK.V | 39 | 1-14 | |  |
|  |  |  |  |  | |  |
|  |  |  |  |  | |  |
| NODE_34_fig|6666666.462.peg.1043 | hypothetical protein | -.MKLDDIAR.M | 22 | 1-8 | | 48 |
|  |  | K.VEASVPSVSSAQSEPEPK.V | 40 | 57-74 | |  |
|  |  |  |  |  | |  |
|  |  |  |  |  | |  |
| NODE_12_fig|6666666.462.peg.42 | hypothetical protein | K.LGLIVSNFNIK.T | 45 | 513-523 | | 191 |
|  |  | K.LATNDINNITTK.S | 33 | 368-379 | |  |
|  |  | K.IAPNLASELFDLAK.S | 62 | 86-99 | |  |
|  |  | K.NQPAGAPSEQTVSNALDNVGK.L | 90 | 36-56 | |  |
|  |  |  |  |  | |  |
|  |  |  |  |  | |  |
| NODE_8_fig|6666666.462.peg.1832 | hypothetical protein | R.SFKDESELQNDALR.A | 58 | 187-200 | | 58 |
|  |  |  |  |  | |  |
|  |  |  |  |  | |  |
| NODE_27_fig|6666666.462.peg.842 | hypothetical protein | R.ALNLLDSSGSLSGDVSK.I | 92 | 350-366 | | 92 |
|  |  |  |  |  | |  |
|  |  |  |  |  | |  |
| NODE_34_fig|6666666.462.peg.950 | FIG008220: hypothetical protein | R.KEVYAK.K | 22 | 158-163 | | 724 |
|  |  | K.TFSAVVK.G | 31 | 210-216 | |  |
|  |  | K.LVAAMNQK.I | 35 | 167-174 | |  |
|  |  | K.ILAFYEK.I | 25 | 175-181 | |  |
|  |  | R.ILYKQEQ.- | 25 | 229-235 | |  |
|  |  | K.TELEGNLEK.I | 31 | 133-141 | |  |
|  |  | K.LIDNKNAQK.D | 24 | 117-125 | |  |
|  |  | R.LDEEVNDLK.S | 49 | 49-57 | |  |
|  |  | R.IQEVEKAYK.S | 34 | 25-33 | |  |
|  |  | K.NAQKDELNAK.K | 49 | 122-131 | |  |
|  |  | K.KTELEGNLEK.I | 34 | 132-141 | |  |
|  |  | K.WAHNTAVVPVK.K | 51 | 185-195 | |  |
|  |  | K.IKSEVSSELEK.I | 29 | 142-152 | |  |
|  |  | K.IKSEVSSELEK.I | 44 | 142-152 | |  |
|  |  | K.TELEGNLEKIK.S | 29 | 133-143 | |  |
|  |  | K.SGTNAHIAEFSAK.I | 63 | 61-73 | |  |
|  |  | K.WAHNTAVVPVKK.Q | 34 | 185-196 | |  |
|  |  | K.EQLEAANEEIAR.L | 54 | 102-113 | |  |
|  |  | K.GEDIVTCPHCGR.I | 42 | 217-228 | |  |
|  |  | R.LDEEVNDLKSQK.S | 84 | 49-60 | |  |
|  |  | R.LDEEVNDLKSQK.S | 40 | 49-60 | |  |
|  |  |  |  |  | |  |
|  |  |  |  |  | |  |
| NODE_34_fig|6666666.462.peg.953 | hypothetical protein | R.SHQKGDENLK.I | 27 | 72-81 | | 82 |
|  |  | R.YFESSELER.A | 33 | 109-117 | |  |
|  |  | K.YALFSFANEPQNVAK.F | 48 | 87-101 | |  |
|  |  |  |  |  | |  |
|  |  |  |  |  | |  |
| NODE_34_fig|6666666.462.peg.954 | hypothetical protein | K.TIGTILNLHEK.V | 23 | 74-84 | | 23 |
|  |  |  |  |  | |  |
|  |  |  |  |  | |  |
| NODE_34_fig|6666666.462.peg.957 | hypothetical protein | K.ADEPQANSSK.H | 39 | 49-58 | | 39 |
|  |  |  |  |  | |  |
|  |  |  |  |  | |  |
| NODE_15_fig|6666666.462.peg.361 | hypothetical protein | R.ELINAFVEK.S | 33 | 43-51 | | 58 |
|  |  | K.DIEEILKPENK.- | 39 | 68-78 | |  |
|  |  |  |  |  | |  |
|  |  |  |  |  | |  |
| NODE_15_fig|6666666.462.peg.362 | hypothetical protein | K.VISVNIADSGTK.N | 63 | 81-92 | | 138 |
|  |  | K.LDGQTANFTYK.S | 28 | 107-117 | |  |
|  |  | K.SGISLKYNDSVLK.G | 47 | 50-62 | |  |
|  |  | R.SDAIVANAKTQIAAIK.S | 38 | 34-49 | |  |
|  |  |  |  |  | |  |
|  |  |  |  |  | |  |
| NODE_13_fig|6666666.462.peg.161 | hypothetical protein | R.NAEIFTGER.A | 33 | 5-13 | | 233 |
|  |  | K.INGGYLEPLAR.A | 66 | 62-72 | |  |
|  |  | K.GINFTNCVFENGESPLK.H | 102 | 20-36 | |  |
|  |  |  |  |  | |  |
|  |  |  |  |  | |  |
| NODE_20_fig|6666666.462.peg.560 | hypothetical protein | K.ILIVAENNK.I | 24 | 65-73 | | 57 |
|  |  | K.ILIVAENNKIR.Y | 34 | 65-75 | |  |
|  |  | K.FVSEPSEMIYK.S | 26 | 82-92 | |  |
|  |  |  |  |  | |  |
|  |  |  |  |  | |  |
| NODE_23_fig|6666666.462.peg.667 | hypothetical protein | R.GASWSKYM.- | 22 | 173-180 | | 178 |
|  |  | R.MFPNQDMPK.E | 23 | 148-156 | |  |
|  |  | -.MQSIDTSLIK.I | 44 | 1-10 | |  |
|  |  | -.MQSIDTSLIK.I | 44 | 1-10 | |  |
|  |  | K.IITTHYYIK.R | 29 | 11-19 | |  |
|  |  | K.INEPLTYSVMK.E | 51 | 41-51 | |  |
|  |  | R.EEGFINFTAYETK.T | 36 | 96-108 | |  |
|  |  |  |  |  | |  |
|  |  |  |  |  | |  |
| NODE_23_fig|6666666.462.peg.660 | hypothetical protein | K.IGLVFNQR.W | 41 | 139-146 | | 113 |
|  |  | K.FISQNEAIFEEGESK.I | 85 | 196-210 | |  |
|  |  |  |  |  | |  |
|  |  |  |  |  | |  |
| NODE_41_fig|6666666.462.peg.1189 | hypothetical protein | R.VSLNLGLINR.K | 52 | 198-207 | | 86 |
|  |  | R.IGQSKELETIVK.N | 47 | 240-251 | |  |
|  |  |  |  |  | |  |
|  |  |  |  |  | |  |
| NODE_51_fig|6666666.462.peg.1354 | FIG099352: hypothetical protein | K.IDVVLLK.S | 27 | 62-68 | | 121 |
|  |  | R.EIDVVKTELK.R | 25 | 326-335 | |  |
|  |  | R.ANFEMTTPTGAAILK.A | 44 | 191-205 | |  |
|  |  | R.AMICDVDESQNLVQK.M | 63 | 240-254 | |  |
|  |  |  |  |  | |  |
|  |  |  |  |  | |  |
| NODE_51_fig|6666666.462.peg.1357 | hypothetical protein | K.AAVEIKK.Y | 30 | 357-363 | | 30 |
|  |  |  |  |  | |  |
|  |  |  |  |  | |  |
| NODE_38_fig|6666666.462.peg.1113 | hypothetical protein | R.NLKPNTK.F | 24 | 43-49 | | 206 |
|  |  | K.AGAIITGSK.G | 40 | 65-73 | |  |
|  |  | K.FKEIISK.Y | 23 | 212-218 | |  |
|  |  | K.AIAIIPDVTK.A | 34 | 55-64 | |  |
|  |  | K.NAINILNAFGTR.N | 53 | 31-42 | |  |
|  |  | K.NSEAYASLFNVK.D | 70 | 117-128 | |  |
|  |  | K.INAQDTNDYYDR.M | 40 | 180-191 | |  |
|  |  |  |  |  | |  |
|  |  |  |  |  | |  |
| NODE_38_fig|6666666.462.peg.1118 | hypothetical protein | K.NFNKEEEDE.- | 30 | 208-216 | | 102 |
|  |  | -.MLNELLNASYTSEK.N | 51 | 1-14 | |  |
|  |  |  |  |  | |  |
|  |  |  |  |  | |  |
| NODE_38_fig|6666666.462.peg.1119 | hypothetical protein | K.DGLDKGK.E | 27 | 44-50 | | 219 |
|  |  | K.DAKDFMVK.E | 39 | 66-73 | |  |
|  |  | K.DFMVKEEK.K | 26 | 69-76 | |  |
|  |  | -.MNNLQNQTK.K | 43 | 1-9 | |  |
|  |  | -.MNNLQNQTK.K | 44 | 1-9 | |  |
|  |  |  |  |  | |  |
|  |  |  |  |  | |  |
| NODE_7_fig|6666666.462.peg.1704 | hypothetical protein | K.ESILELSR.I | 24 | 45-52 | | 249 |
|  |  | K.IIDVPAKLD.- | 47 | 126-134 | |  |
|  |  | K.SAAGFMHLDPILK.L | 24 | 59-71 | |  |
|  |  | K.LTTLAEEVTQEAR.S | 77 | 72-84 | |  |
|  |  |  |  |  | |  |
|  |  |  |  |  | |  |
| NODE_7_fig|6666666.462.peg.1702 | hypothetical protein | R.NISQNYLESEELF.- | 42 | 283-295 | | 42 |
|  |  |  |  |  | |  |
|  |  |  |  |  | |  |
| NODE_15_fig|6666666.462.peg.285 | hypothetical protein | R.SPQLLAK.D | 45 | 166-172 | | 245 |
|  |  | K.NQFIINK.Q | 21 | 37-43 | |  |
|  |  | R.VEKNQFIINK.Q | 30 | 34-43 | |  |
|  |  | R.FNEILVYDPK.Q | 55 | 119-128 | |  |
|  |  | K.QSAIFDDLQDSDLTLLSSK.K | 79 | 44-62 | |  |
|  |  |  |  |  | |  |
|  |  |  |  |  | |  |
| NODE_16_fig|6666666.462.peg.389 | hypothetical protein | K.IDQYFDSK.K | 32 | 9-16 | | 32 |
|  |  |  |  |  | |  |
|  |  |  |  |  | |  |
| NODE_16_fig|6666666.462.peg.388 | hypothetical protein | K.VEAFNFLK.T | 22 | 30-37 | | 22 |
|  |  |  |  |  | |  |
|  |  |  |  |  | |  |
| NODE_16_fig|6666666.462.peg.382 | hypothetical protein | K.AVIAYIK.L | 24 | 210-216 | | 2850 |
|  |  | K.AYFLQR.K | 32 | 426-431 | |  |
|  |  | K.LAERTQK.S | 34 | 564-570 | |  |
|  |  | K.GLSVDEQIK.S | 29 | 125-133 | |  |
|  |  | R.SLFEFNLK.D | 23 | 300-307 | |  |
|  |  | R.LTLINEINK.T | 52 | 436-444 | |  |
|  |  | R.GFAVVADEVR.K | 49 | 553-562 | |  |
|  |  | R.INFEEATKK.D | 39 | 329-337 | |  |
|  |  | R.INFEEATKK.D | 30 | 329-337 | |  |
|  |  | K.MMSGYAFGVK.I | 40 | 282-291 | |  |
|  |  | K.FSILSGNLQK.R | 46 | 238-247 | |  |
|  |  | K.RINFEEATK.K | 22 | 328-336 | |  |
|  |  | R.EIYEFLDTK.T | 37 | 249-257 | |  |
|  |  | K.TQNFQDTGKK.F | 28 | 228-237 | |  |
|  |  | K.TQNFQDTGKK.F | 29 | 228-237 | |  |
|  |  | R.GFAVVADEVRK.L | 57 | 553-563 | |  |
|  |  | R.GFAVVADEVRK.L | 31 | 553-563 | |  |
|  |  | K.EISENFKDVK.T | 20 | 466-475 | |  |
|  |  | R.SLFEFNLKDK.N | 36 | 300-309 | |  |
|  |  | K.NANAISSQIESK.T | 60 | 620-631 | |  |
|  |  | K.FSILSGNLQKR.E | 34 | 238-248 | |  |
|  |  | K.REIYEFLDTK.T | 21 | 248-257 | |  |
|  |  | K.IAIYENILEKL.- | 63 | 645-655 | |  |
|  |  | K.SSVPIISGVSGTLK.E | 53 | 452-465 | |  |
|  |  | R.SSFISNTILDLVK.N | 71 | 607-619 | |  |
|  |  | K.YLDDRIVSNSEK.I | 60 | 491-502 | |  |
|  |  | K.YLDDRIVSNSEK.I | 24 | 491-502 | |  |
|  |  | K.GLSVDEQIKSIER.E | 51 | 125-137 | |  |
|  |  | R.VENINENTKDTSK.D | 47 | 586-598 | |  |
|  |  | R.AGEAGRGFAVVADEVR.K | 43 | 547-562 | |  |
|  |  | R.AGEAGRGFAVVADEVR.K | 41 | 547-562 | |  |
|  |  | K.SVVQRVENINENTK.D | 65 | 581-594 | |  |
|  |  | K.TNLSNVNDKFGDFSK.Y | 30 | 476-490 | |  |
|  |  | K.TNLSNVNDKFGDFSK.Y | 81 | 476-490 | |  |
|  |  | R.VFCDIDNAEELIVVK.R | 81 | 313-327 | |  |
|  |  | K.QEGEFNDNYVNSFTK.H | 59 | 405-419 | |  |
|  |  | R.LTLINEINKTMLTQIK.S | 61 | 436-451 | |  |
|  |  | R.LTLINEINKTMLTQIK.S | 42 | 436-451 | |  |
|  |  | K.VVIGYAMPNVNLANAASSIK.S | 31 | 41-60 | |  |
|  |  | K.VVIGYAMPNVNLANAASSIK.S | 110 | 41-60 | |  |
|  |  | K.VVIGYAMPNVNLANAASSIK.S | 75 | 41-60 | |  |
|  |  | K.LDFSGTYIYNNSQIVQSK.A | 82 | 192-209 | |  |
|  |  |  |  |  | |  |
|  |  |  |  |  | |  |
| NODE_45_fig|6666666.462.peg.1234 | hypothetical protein | K.ISPVHKDAK.R | 24 | 235-243 | | 474 |
|  |  | K.KNLAQNVEAVK.N | 55 | 21-31 | |  |
|  |  | K.FGVSVSDEEIR.N | 55 | 158-168 | |  |
|  |  | K.IAAIFCTYTPR.E | 46 | 37-47 | |  |
|  |  | K.EQISDLEIIVEK.L | 52 | 215-226 | |  |
|  |  | R.IIITGCPSGGVYDK.I | 44 | 245-258 | |  |
|  |  | R.NSDIVIGETTCDGK.K | 66 | 100-113 | |  |
|  |  | R.YMAIPCSIMSPNK.E | 20 | 300-312 | |  |
|  |  | K.FGVSVSDEEIRNSIR.L | 28 | 158-172 | |  |
|  |  | K.NFSNLIDENDDPINAIAK.R | 77 | 281-298 | |  |
|  |  |  |  |  | |  |
|  |  |  |  |  | |  |
| NODE_45_fig|6666666.462.peg.1230 | hypothetical protein | R.LENLVQNSTQR.G | 59 | 57-67 | | 75 |
|  |  |  |  |  | |  |
|  |  |  |  |  | |  |
| NODE_6_fig|6666666.462.peg.1572 | hypothetical protein | K.AIPAEIIALYS.- | 31 | 120-130 | | 31 |
|  |  |  |  |  | |  |
|  |  |  |  |  | |  |
| NODE_6_fig|6666666.462.peg.1575 | hypothetical protein | K.VLDSIFK.- | 21 | 243-249 | | 350 |
|  |  | K.WAQDLSK.S | 33 | 89-95 | |  |
|  |  | K.NMSEADVKK.L | 29 | 118-126 | |  |
|  |  | R.SGFSLDSGKK.V | 55 | 233-242 | |  |
|  |  | K.SLEMASNLAK.K | 48 | 73-82 | |  |
|  |  | K.EGFINNATAK.I | 42 | 58-67 | |  |
|  |  | K.SLEMASNLAK.K | 23 | 73-82 | |  |
|  |  | K.AAEIFSESIK.N | 38 | 108-117 | |  |
|  |  | K.IATHASSGDYK.S | 22 | 31-41 | |  |
|  |  | K.SINNAATTAVPK.A | 51 | 96-107 | |  |
|  |  | K.SLEMASNLAKK.V | 39 | 73-83 | |  |
|  |  | K.SLEMASNLAKK.V | 26 | 73-83 | |  |
|  |  | K.VGGEKWAQDLSK.S | 44 | 84-95 | |  |
|  |  | K.TLDGLFNVMSEK.E | 39 | 216-227 | |  |
|  |  |  |  |  | |  |
|  |  |  |  |  | |  |
| NODE_23_fig|6666666.462.peg.722 | hypothetical protein | R.QNMEGGNFTFGAR.S | 24 | 131-143 | | 84 |
|  |  | K.IDTQAKDDGDIAVGVSK.F | 56 | 32-48 | |  |
|  |  | K.IDTQAKDDGDIAVGVSK.F | 30 | 32-48 | |  |
|  |  |  |  |  | |  |
|  |  |  |  |  | |  |
| NODE_23_fig|6666666.462.peg.721 | hypothetical protein | K.SAYELLLK.I | 37 | 59-66 | | 56 |
|  |  |  |  |  | |  |
|  |  |  |  |  | |  |
| NODE_14_fig|6666666.462.peg.232 | hypothetical protein | K.VENLETKK.E | 21 | 174-181 | | 36 |
|  |  | R.ISMLNEFR.N | 28 | 123-130 | |  |
|  |  |  |  |  | |  |
|  |  |  |  |  | |  |
| NODE_5_fig|6666666.462.peg.1474 | hypothetical protein | K.GALGLDAPK.L | 45 | 66-74 | | 79 |
|  |  | K.IIDKQTK.G | 24 | 59-65 | |  |
|  |  | K.LKEILLNEQC.- | 36 | 75-84 | |  |
|  |  |  |  |  | |  |
|  |  |  |  |  | |  |
| NODE_15_fig|6666666.462.peg.294 | hypothetical protein | R.QISNYAFSVDIAGGK.L | 46 | 124-138 | | 46 |
|  |  |  |  |  | |  |
|  |  |  |  |  | |  |
| NODE_12_fig|6666666.462.peg.62 | hypothetical protein | K.TQEILSK.A | 24 | 348-354 | | 557 |
|  |  | R.DALLATIK.A | 32 | 132-139 | |  |
|  |  | K.AANLKDAR.V | 24 | 37-44 | |  |
|  |  | R.TKTQEILSK.A | 29 | 346-354 | |  |
|  |  | K.LAALAYEPKK.T | 25 | 396-405 | |  |
|  |  | K.EQLNDFLFNR.S | 49 | 295-304 | |  |
|  |  | K.AYPQSLNLNEVK.A | 64 | 355-366 | |  |
|  |  | R.FDAHIEQSYNTR.I | 37 | 280-291 | |  |
|  |  | K.GANLSEDELKELAFK.L | 83 | 462-476 | |  |
|  |  | K.VVGIDISSHQVEEGNK.I | 66 | 68-83 | |  |
|  |  | K.FSLQNQSDVVYKDSMK.L | 54 | 197-212 | |  |
|  |  | R.VLELGSSYGGNILPFAASHK.E | 38 | 45-64 | |  |
|  |  |  |  |  | |  |
|  |  |  |  |  | |  |
| NODE_12_fig|6666666.462.peg.66 | hypothetical protein | K.LFDVSLSR.L | 47 | 327-334 | | 146 |
|  |  | K.NSQILDFLK.D | 42 | 11-19 | |  |
|  |  | K.ALNLEYENVK.S | 36 | 289-298 | |  |
|  |  | R.EISPNFTANLK.S | 35 | 178-188 | |  |
|  |  |  |  |  | |  |
|  |  |  |  |  | |  |
| NODE_16_fig|6666666.462.peg.394 | hypothetical protein | R.LATPIDKLVK.S | 26 | 68-77 | | 26 |
|  |  |  |  |  | |  |
|  |  |  |  |  | |  |
| NODE_34_fig|6666666.462.peg.973 | hypothetical protein | K.GLSVYHR.T | 20 | 56-62 | | 219 |
|  |  | -.MELKLAR.A | 31 | 1-7 | |  |
|  |  | R.AELDAKPK.T | 34 | 8-15 | |  |
|  |  | K.IEAAVEKEGQK.I | 58 | 22-32 | |  |
|  |  | K.TISLEKIEAAVEK.E | 40 | 16-28 | |  |
|  |  | K.TISLEKIEAAVEK.E | 35 | 16-28 | |  |
|  |  | K.QLIALVEHFEEK.G | 48 | 44-55 | |  |
|  |  |  |  |  | |  |
|  |  |  |  |  | |  |
| NODE_34_fig|6666666.462.peg.976 | hypothetical protein | K.ALDLDAYNK.M | 49 | 52-60 | | 49 |
|  |  |  |  |  | |  |
|  |  |  |  |  | |  |
| NODE_34_fig|6666666.462.peg.977 | hypothetical protein | K.SVAIFGR.D | 24 | 254-260 | | 120 |
|  |  | K.SLDDFLSDDKPK.E | 52 | 223-234 | |  |
|  |  | K.FTDISIENIDEK.V | 70 | 43-54 | |  |
|  |  |  |  |  | |  |
|  |  |  |  |  | |  |
| NODE_15_fig|6666666.462.peg.345 | hypothetical protein | R.FTVDITKPNK.E | 45 | 396-405 | | 219 |
|  |  | K.FSPNGEFLSGK.Q | 57 | 421-431 | |  |
|  |  | K.ELNLSNIDPNVAK.Y | 34 | 63-75 | |  |
|  |  | R.DPGTYLYTPIPGIR.N | 33 | 584-597 | |  |
|  |  | K.KPIASAVELSELLYQK.S | 77 | 502-517 | |  |
|  |  |  |  |  | |  |
|  |  |  |  |  | |  |
| NODE_15_fig|6666666.462.peg.343 | hypothetical protein | R.IFQNINIK.V | 28 | 321-328 | | 46 |
|  |  | K.INIFNSDINNNGAMSR.E | 32 | 266-281 | |  |
|  |  |  |  |  | |  |
|  |  |  |  |  | |  |
| NODE_6_fig|6666666.462.peg.1617 | hypothetical protein | R.VQEVLAK.A | 31 | 86-92 | | 31 |
|  |  |  |  |  | |  |
|  |  |  |  |  | |  |
| NODE_18_fig|6666666.462.peg.423 | hypothetical protein | R.VVSHDEAR.G | 44 | 97-104 | | 44 |
|  |  |  |  |  | |  |
|  |  |  |  |  | |  |
| NODE_18_fig|6666666.462.peg.420 | hypothetical protein | K.SFAAEIK.K | 30 | 55-61 | | 150 |
|  |  | K.SIEGGFLTK.I | 25 | 38-46 | |  |
|  |  | K.LEILNEANAPAPK.L | 56 | 63-75 | |  |
|  |  | K.KLEILNEANAPAPK.L | 69 | 62-75 | |  |
|  |  |  |  |  | |  |
|  |  |  |  |  | |  |
| NODE_18_fig|6666666.462.peg.421 | hypothetical protein | K.SDSIPEAK.K | 22 | 319-326 | | 73 |
|  |  | R.NLGLALLDSK.V | 48 | 130-139 | |  |
|  |  | K.LLNFFIDPR.S | 28 | 99-107 | |  |
|  |  |  |  |  | |  |
|  |  |  |  |  | |  |
| NODE_12_fig|6666666.462.peg.85 | hypothetical protein | R.LSNLFNK.T | 24 | 95-101 | | 24 |
|  |  |  |  |  | |  |
|  |  |  |  |  | |  |
| NODE_8_fig|6666666.462.peg.1789 | hypothetical protein | K.ESGILPADFR.L | 33 | 139-148 | | 87 |
|  |  | K.SPYYFIDAR.E | 29 | 273-281 | |  |
|  |  | R.LNASITMVPTK.N | 50 | 149-159 | |  |
|  |  |  |  |  | |  |
|  |  |  |  |  | |  |
| NODE_27_fig|6666666.462.peg.860 | hypothetical protein | K.QVDALSK.E | 36 | 125-131 | | 258 |
|  |  | K.LAQALILR.A | 50 | 3-10 | |  |
|  |  | K.VDLYSNSEIK.I | 35 | 103-112 | |  |
|  |  | K.ILSTVDVAALQK.Q | 42 | 113-124 | |  |
|  |  | K.FDGMSLTEMIAK.K | 38 | 70-81 | |  |
|  |  | K.FDGMSLTEMIAK.K | 39 | 70-81 | |  |
|  |  | K.TQENESPSEDPK.L | 52 | 30-41 | |  |
|  |  | K.LQEANWSVDLVE.- | 33 | 140-151 | |  |
|  |  |  |  |  | |  |
|  |  |  |  |  | |  |
| NODE_6_fig|6666666.462.peg.1545 | hypothetical protein | K.YYEVLVR.I | 34 | 571-577 | | 512 |
|  |  | K.ASFALDFAK.S | 37 | 159-167 | |  |
|  |  | K.NLNDDFTR.K | 25 | 72-79 | |  |
|  |  | K.KLPTDENAR.V | 27 | 717-725 | |  |
|  |  | K.VIALAFDLVK.K | 49 | 608-617 | |  |
|  |  | K.TQLYLQITK.K | 36 | 598-606 | |  |
|  |  | K.FTLENNILR.L | 39 | 23-31 | |  |
|  |  | R.VLVETIVSFAR.R | 52 | 726-736 | |  |
|  |  | K.LNSLIQIFQTEK.S | 68 | 54-65 | |  |
|  |  | K.SNIHPESFYAEIR.N | 25 | 286-298 | |  |
|  |  | K.ITLGVSLSNDTPQTNR.L | 50 | 487-502 | |  |
|  |  | K.SLNTIAEESSNMELGK.N | 80 | 168-183 | |  |
|  |  | K.STAIINPATLNQTEEQIK.K | 62 | 123-140 | |  |
|  |  | K.STAIINPATLNQTEEQIKK.Y | 20 | 123-141 | |  |
|  |  | K.STAIINPATLNQTEEQIKK.Y | 55 | 123-141 | |  |
|  |  |  |  |  | |  |
|  |  |  |  |  | |  |
| NODE_6_fig|6666666.462.peg.1543 | hypothetical protein | K.LLNNQFK.Y | 22 | 72-78 | | 199 |
|  |  | K.IADMTAAGKK.C | 38 | 15-24 | |  |
|  |  | K.GKPAVIKVEK.G | 48 | 42-51 | |  |
|  |  | K.GANQNDSLCLK.I | 44 | 52-62 | |  |
|  |  | K.FTLVDFVTDTGDTK.G | 75 | 28-41 | |  |
|  |  | K.ESETGEYPVSGLSVAF.- | 37 | 88-103 | |  |
|  |  |  |  |  | |  |
|  |  |  |  |  | |  |
| NODE_80_fig|6666666.462.peg.1765 | hypothetical protein | K.INDGASYVK.E | 54 | 39-47 | | 89 |
|  |  | K.EKINDGASYVK.E | 48 | 37-47 | |  |
|  |  |  |  |  | |  |
|  |  |  |  |  | |  |
| NODE_14_fig|6666666.462.peg.225 | hypothetical protein | K.IMQECQEQK.G | 34 | 23-31 | | 34 |
|  |  |  |  |  | |  |
|  |  |  |  |  | |  |
| NODE_14_fig|6666666.462.peg.227 | hypothetical protein | R.GYALIATQSDQIAPAIK.Q | 85 | 36-52 | | 94 |
|  |  |  |  |  | |  |
|  |  |  |  |  | |  |
| NODE_14_fig|6666666.462.peg.229 | hypothetical protein | K.GIFVSLPDEQAPSNVSK.K | 56 | 21-37 | | 56 |
|  |  |  |  |  | |  |
|  |  |  |  |  | |  |
| NODE_40_fig|6666666.462.peg.1155 | hypothetical protein | R.ALNDLTGEVR.Q | 45 | 18-27 | | 45 |
|  |  |  |  |  | |  |
|  |  |  |  |  | |  |
| NODE_40_fig|6666666.462.peg.1157 | hypothetical protein | R.LDEISAYKDK.N | 30 | 83-92 | | 535 |
|  |  | K.GADLAAIQADNK.K | 61 | 36-47 | |  |
|  |  | K.LAQILSDKGYK.V | 53 | 208-218 | |  |
|  |  | K.LAQILSDKGYK.V | 26 | 208-218 | |  |
|  |  | K.FGSINAEEFKK.I | 63 | 135-145 | |  |
|  |  | K.TIVTHCYSGNR.S | 42 | 194-204 | |  |
|  |  | K.IKGADLAAIQADNK.K | 24 | 34-47 | |  |
|  |  | K.IANDPNVLIIDVR.E | 24 | 146-158 | |  |
|  |  | K.IANDPNVLIIDVR.E | 45 | 146-158 | |  |
|  |  | K.HAISIPLEEIEAR.L | 28 | 70-82 | |  |
|  |  | K.HAISIPLEEIEAR.L | 44 | 70-82 | |  |
|  |  | K.GAISIPDGEPVDNYK.D | 56 | 170-184 | |  |
|  |  | K.IANDPNVLIIDVREK.K | 42 | 146-160 | |  |
|  |  | K.LSDAEGVKEFSYDLVK.F | 70 | 119-134 | |  |
|  |  | K.LSDAEGVKEFSYDLVK.F | 37 | 119-134 | |  |
|  |  |  |  |  | |  |
|  |  |  |  |  | |  |
| NODE_35_fig|6666666.462.peg.1075 | hypothetical protein | K.SELATLLK.N | 26 | 481-488 | | 435 |
|  |  | K.AQILIGIR.I | 31 | 147-154 | |  |
|  |  | K.IDIGVNSTITK.V | 60 | 105-115 | |  |
|  |  | K.ANDVNIGGQTHQK.A | 62 | 362-374 | |  |
|  |  | K.QYSVPVEFVDFR.L | 29 | 32-43 | |  |
|  |  | K.MPESAEATQDQLSK.I | 84 | 462-475 | |  |
|  |  | K.NVINENKDSIYTIK.A | 64 | 495-508 | |  |
|  |  | R.FVIVDPPQNVTYVSK.Q | 61 | 586-600 | |  |
|  |  | K.EYQGLVNEYNTLLR.I | 44 | 531-544 | |  |
|  |  | K.TTGSIQAGTDTNVTLVVR.E | 70 | 311-328 | |  |
|  |  |  |  |  | |  |
|  |  |  |  |  | |  |
| NODE_35_fig|6666666.462.peg.1072 | hypothetical protein | R.NFSSVLSR.V | 35 | 18-25 | | 35 |
|  |  |  |  |  | |  |
|  |  |  |  |  | |  |
| NODE_19_fig|6666666.462.peg.540 | hypothetical protein | K.NKETSELVK.A | 29 | 148-156 | | 37 |
|  |  | K.EMIFEVDEK.N | 21 | 95-103 | |  |
|  |  |  |  |  | |  |
|  |  |  |  |  | |  |
| NODE_7_fig|6666666.462.peg.1724 | hypothetical protein | K.MICIQTK.R | 22 | 95-101 | | 93 |
|  |  | -.MKNYQVAK.I | 26 | 1-8 | |  |
|  |  | -.MKNYQVAK.I | 40 | 1-8 | |  |
|  |  | K.MICIQTKR.G | 30 | 95-102 | |  |
|  |  |  |  |  | |  |
|  |  |  |  |  | |  |
| NODE_7_fig|6666666.462.peg.1722 | hypothetical protein | K.AAGLVNVVAGVGGVSLAK.E | 77 | 65-82 | | 99 |
|  |  | K.AAGLVNVVAGVGGVSLAK.E | 36 | 65-82 | |  |
|  |  |  |  |  | |  |
|  |  |  |  |  | |  |
| NODE_7_fig|6666666.462.peg.1720 | hypothetical protein | K.SDDEKFK.G | 20 | 13-19 | | 20 |
|  |  |  |  |  | |  |
|  |  |  |  |  | |  |
| NODE_8_fig|6666666.462.peg.1808 | hypothetical protein | K.ESLTQLFR.D | 24 | 65-72 | | 62 |
|  |  | K.LKNENALDSATK.E | 50 | 53-64 | |  |
|  |  |  |  |  | |  |
|  |  |  |  |  | |  |
| NODE_34_fig|6666666.462.peg.987 | hypothetical protein | R.YGNIDGLR.S | 58 | 101-108 | | 150 |
|  |  | K.DGNVLAMQQTGVCFR.D | 67 | 65-79 | |  |
|  |  |  |  |  | |  |
|  |  |  |  |  | |  |
| NODE_34_fig|6666666.462.peg.985 | hypothetical protein | R.ILNNTDGLLPK.S | 68 | 25-35 | | 294 |
|  |  | K.INETTYYIMGK.I | 66 | 93-103 | |  |
|  |  | K.INETTYYIMGK.I | 53 | 93-103 | |  |
|  |  | R.RILNNTDGLLPK.S | 23 | 24-35 | |  |
|  |  | R.RILNNTDGLLPK.S | 21 | 24-35 | |  |
|  |  | K.KINETTYYIMGK.I | 44 | 92-103 | |  |
|  |  |  |  |  | |  |
|  |  |  |  |  | |  |
| NODE_34_fig|6666666.462.peg.983 | hypothetical protein | K.NAIDLLTPK.C | 39 | 50-58 | | 431 |
|  |  | K.QDDSWQQPK.D | 44 | 28-36 | |  |
|  |  | K.VVQNKEQISK.N | 26 | 40-49 | |  |
|  |  | K.VVQNKEQISK.N | 61 | 40-49 | |  |
|  |  | K.EYDNAYANYKK.A | 28 | 79-89 | |  |
|  |  | K.QDDSWQQPKDEK.V | 36 | 28-39 | |  |
|  |  | K.VQLACSNLGTLYENGLGVK.K | 97 | 95-113 | |  |
|  |  |  |  |  | |  |
|  |  |  |  |  | |  |
| NODE_15_fig|6666666.462.peg.336 | hypothetical protein | R.SMQTPR.L | 23 | 184-189 | | 1290 |
|  |  | K.NNKIAEK.Q | 23 | 169-175 | |  |
|  |  | K.ISVVKDSK.K | 33 | 105-112 | |  |
|  |  | K.VINGYMELK.K | 31 | 145-153 | |  |
|  |  | K.QQQIEYLR.S | 36 | 176-183 | |  |
|  |  | K.FFDIEVIAK.T | 47 | 114-122 | |  |
|  |  | K.VINGYMELKK.I | 23 | 145-154 | |  |
|  |  | K.GVEGLDYQVQK.I | 47 | 94-104 | |  |
|  |  | K.TNDIALKQINK.M | 23 | 123-133 | |  |
|  |  | K.TNDIALKQINK.M | 48 | 123-133 | |  |
|  |  | K.MVEDLANEHQK.V | 42 | 134-144 | |  |
|  |  | K.MVEDLANEHQK.V | 23 | 134-144 | |  |
|  |  | K.MVEDLANEHQK.V | 35 | 134-144 | |  |
|  |  | K.ELLNLQTEELNK.L | 68 | 240-251 | |  |
|  |  | K.QTTNLEQYATLAK.D | 75 | 193-205 | |  |
|  |  | K.IAEKQQQIEYLR.S | 44 | 172-183 | |  |
|  |  | R.DISTNDKLLFLEK.E | 35 | 227-239 | |  |
|  |  | R.DISTNDKLLFLEK.E | 47 | 227-239 | |  |
|  |  | K.IQLANIDSQINFLK.N | 81 | 155-168 | |  |
|  |  | R.LDKQTTNLEQYATLAK.D | 53 | 190-205 | |  |
|  |  | R.LDKQTTNLEQYATLAK.D | 84 | 190-205 | |  |
|  |  |  |  |  | |  |
|  |  |  |  |  | |  |
| NODE_18_fig|6666666.462.peg.457 | hypothetical protein | K.GLVEIGK.F | 22 | 17-23 | | 115 |
|  |  | K.DIIVGIGK.G | 26 | 9-16 | |  |
|  |  | R.QLDDDELKK.I | 44 | 41-49 | |  |
|  |  | K.YGSGIDKDIAK.T | 27 | 53-63 | |  |
|  |  | K.YGSGIDKDIAK.T | 47 | 53-63 | |  |
|  |  |  |  |  | |  |
|  |  |  |  |  | |  |
| NODE_8_fig|6666666.462.peg.1790 | hypothetical protein | K.AGFRNEPNIK.N | 20 | 66-75 | | 217 |
|  |  | K.TSDPIFFTLGEANK.S | 72 | 27-40 | |  |
|  |  | K.NSATGQDVNAEIISEFAK.A | 83 | 48-65 | |  |
|  |  |  |  |  | |  |
|  |  |  |  |  | |  |
| NODE_23_fig|6666666.462.peg.708 | hypothetical protein | K.NQAIEQTSNQK.N | 65 | 33-43 | | 133 |
|  |  | R.SIAGVMANKVLPVK.T | 48 | 143-156 | |  |
|  |  |  |  |  | |  |
|  |  |  |  |  | |  |
| NODE_23_fig|6666666.462.peg.701 | hypothetical protein | K.LLNVSSQTGAIR.V | 52 | 99-110 | | 52 |
|  |  |  |  |  | |  |
|  |  |  |  |  | |  |
| NODE_23_fig|6666666.462.peg.703 | hypothetical protein | K.SMNVLIIK.R | 45 | 226-233 | | 207 |
|  |  | K.SLNLFIDEK.R | 40 | 404-412 | |  |
|  |  | K.SLKDENIFVLSK.F | 32 | 329-340 | |  |
|  |  | R.SLDDSLLLDPELIYK.S | 50 | 314-328 | |  |
|  |  |  |  |  | |  |
|  |  |  |  |  | |  |
| NODE_23_fig|6666666.462.peg.674 | hypothetical protein | K.GISELANLK.V | 38 | 110-118 | | 427 |
|  |  | M.AIKEDLTEIK.K | 32 | 2-11 | |  |
|  |  | K.IALDSQLQTLVK.N | 73 | 167-178 | |  |
|  |  | K.LILNPNDASALNVLK.E | 56 | 73-87 | |  |
|  |  | K.LILNPNDASALNVLK.E | 43 | 73-87 | |  |
|  |  | K.EIDAQEQFLESMIK.G | 62 | 13-26 | |  |
|  |  | K.EIDAQEQFLESMIK.G | 51 | 13-26 | |  |
|  |  | K.VLNDNRVEEANLAYSK.L | 36 | 57-72 | |  |
|  |  | K.VLNDNRVEEANLAYSK.L | 38 | 57-72 | |  |
|  |  |  |  |  | |  |
|  |  |  |  |  | |  |
| NODE_36_fig|6666666.462.peg.1089 | hypothetical protein | K.STLDELSR.F | 49 | 99-106 | | 123 |
|  |  | -.MNDISILSTNVNSYNK.Q | 87 | 1-16 | |  |
|  |  |  |  |  | |  |
|  |  |  |  |  | |  |
| NODE_5_fig|6666666.462.peg.1456 | FIG000859: hypothetical protein | K.MSKVFPK.L | 21 | 19-25 | | 539 |
|  |  | R.ANGKDSADIK.T | 33 | 71-80 | |  |
|  |  | R.AAIAAAKAENMPK.D | 52 | 49-61 | |  |
|  |  | R.AAIAAAKAENMPK.D | 46 | 49-61 | |  |
|  |  | K.DSADIKTIFYDGK.A | 48 | 75-87 | |  |
|  |  | K.DGGCDPDMNPKLR.A | 26 | 36-48 | |  |
|  |  | K.AENMPKDNIDAAIK.R | 32 | 56-69 | |  |
|  |  | K.AENMPKDNIDAAIK.R | 45 | 56-69 | |  |
|  |  | K.AENMPKDNIDAAIK.R | 30 | 56-69 | |  |
|  |  | K.AITVAAKDGGCDPDMNPK.L | 57 | 29-46 | |  |
|  |  | K.AAHGVQIIVECATDNPTR.T | 85 | 88-105 | |  |
|  |  | K.AAHGVQIIVECATDNPTR.T | 47 | 88-105 | |  |
|  |  | R.LLDKLEDDDDVQAVYTNIE.- | 70 | 217-235 | |  |
|  |  |  |  |  | |  |
|  |  |  |  |  | |  |
| NODE_7_fig|6666666.462.peg.1758 | hypothetical protein | K.ALGDSGSYDLGK.K | 53 | 146-157 | | 53 |
|  |  |  |  |  | |  |
|  |  |  |  |  | |  |
| NODE_65_fig|6666666.462.peg.1515 | hypothetical protein | K.YIATLPK.L | 21 | 703-709 | | 421 |
|  |  | K.GGTSDLADPK.L | 41 | 533-542 | |  |
|  |  | R.QILAIPDER.V | 31 | 434-442 | |  |
|  |  | K.DSILFAAISK.G | 56 | 523-532 | |  |
|  |  | K.KAFVANVEAK.F | 32 | 371-380 | |  |
|  |  | R.VPIGDMNVIK.S | 24 | 183-192 | |  |
|  |  | K.DIFAGSIYQK.R | 35 | 172-181 | |  |
|  |  | K.VLNDKIVSYEK.S | 54 | 567-577 | |  |
|  |  | K.ALTNDDIKEAAR.L | 27 | 885-896 | |  |
|  |  | K.LGEAENFKDDGVR.S | 57 | 710-722 | |  |
|  |  | K.LLNEITLEDVNAR.F | 76 | 419-431 | |  |
|  |  | K.LGSFAVALTNESGVGK.F | 73 | 543-558 | |  |
|  |  | K.LPNGSQVIFKPLATK.K | 27 | 507-521 | |  |
|  |  | K.LKDEAKPYDTNLASQSLNK.S | 36 | 461-479 | |  |
|  |  |  |  |  | |  |
|  |  |  |  |  | |  |
| NODE_12_fig|6666666.462.peg.125 | hypothetical protein | K.IYSQNAFK.F | 27 | 328-335 | | 170 |
|  |  | K.GIFDFASAAK.E | 32 | 198-207 | |  |
|  |  | K.FVDENFSHK.A | 33 | 336-344 | |  |
|  |  | K.SVPALCEVIR.S | 29 | 67-76 | |  |
|  |  | K.DATIVFSGNISDGTKK.Q | 64 | 214-229 | |  |
|  |  | K.VPVVVYDNAPMNVLVK.D | 51 | 281-296 | |  |
|  |  |  |  |  | |  |
|  |  |  |  |  | |  |
| NODE_12_fig|6666666.462.peg.127 | hypothetical protein | R.NPAEDQNNTQR.H | 47 | 129-139 | | 47 |
|  |  |  |  |  | |  |
|  |  |  |  |  | |  |
| NODE_12_fig|6666666.462.peg.128 | hypothetical protein | K.IIDAVDAK.N | 30 | 32-39 | | 39 |
|  |  | K.AMIDCLLK.S | 22 | 49-56 | |  |
|  |  |  |  |  | |  |
|  |  |  |  |  | |  |
| NODE_3_fig|6666666.462.peg.1129 | hypothetical protein | K.VSVNELR.N | 24 | 51-57 | | 31 |
|  |  | R.SGKVSVNELR.N | 20 | 48-57 | |  |
|  |  |  |  |  | |  |
|  |  |  |  |  | |  |
| NODE_54_fig|6666666.462.peg.1372 | hypothetical protein | K.MAEDLGVPMR.T | 25 | 20-29 | | 25 |
|  |  |  |  |  | |  |
|  |  |  |  |  | |  |
| NODE_34_fig|6666666.462.peg.993 | hypothetical protein | R.ELIEKILA.- | 24 | 124-131 | | 32 |
|  |  |  |  |  | |  |
|  |  |  |  |  | |  |
| NODE_7_fig|6666666.462.peg.1732 | hypothetical protein | K.IQKGDQSIIK.N | (28) | 137-146 | | 150 |
|  |  | K.IQKGDQSIIK.N | 48 | 137-146 | |  |
|  |  | K.NKSTIATITDGK.G | 21 | 57-68 | |  |
|  |  | K.NISNNQTKFELVR.T | 44 | 147-159 | |  |
|  |  | K.GDQSIIKNISNNQTK.F | 62 | 140-154 | |  |
|  |  |  |  |  | |  |
|  |  |  |  |  | |  |
| NODE_42_fig|6666666.462.peg.1216 | hypothetical protein | K.KAEIVSQK.N | 35 | 390-397 | | 180 |
|  |  | K.NNLNLVIER.N | 54 | 398-406 | |  |
|  |  | K.LQEEITNLR.I | 41 | 362-370 | |  |
|  |  | K.ELVDRIETDISTLNK.K | 65 | 240-254 | |  |
|  |  |  |  |  | |  |
|  |  |  |  |  | |  |
| NODE_42_fig|6666666.462.peg.1217 | hypothetical protein | K.RSPLVVAK.E | 23 | 16-23 | | 23 |
|  |  |  |  |  | |  |
|  |  |  |  |  | |  |
| NODE_18_fig|6666666.462.peg.440 | hypothetical protein | K.ISEEVMSKR.E | 42 | 120-128 | | 42 |
|  |  |  |  |  | |  |
|  |  |  |  |  | |  |
| NODE_18_fig|6666666.462.peg.444 | hypothetical protein | K.QSMDQLASLPK.E | 46 | 58-68 | | 501 |
|  |  | K.QSMDQLASLPK.E | 34 | 58-68 | |  |
|  |  | K.EQQDATCKQAK.E | 33 | 69-79 | |  |
|  |  | K.KQSMDQLASLPK.E | 65 | 57-68 | |  |
|  |  | K.KQSMDQLASLPK.E | 25 | 57-68 | |  |
|  |  | K.EMFAQVMDQMK.K | 58 | 80-90 | |  |
|  |  | K.KQSMDQLASLPK.E | 37 | 57-68 | |  |
|  |  | K.EMFAQVMDQMK.K | 50 | 80-90 | |  |
|  |  | K.EMFAQVMDQMK.K | 47 | 80-90 | |  |
|  |  | K.EMFAQVMDQMKK.Q | 49 | 80-91 | |  |
|  |  | K.EMFAQVMDQMKK.Q | 29 | 80-91 | |  |
|  |  | K.EMFAQVMDQMKK.Q | 36 | 80-91 | |  |
|  |  | K.MVEAYKQAGQEQQVK.M | 67 | 36-50 | |  |
|  |  | K.MVEAYKQAGQEQQVK.M | 30 | 36-50 | |  |
|  |  | K.QSMDQLASLPKEQQDATCK.Q | 46 | 58-76 | |  |
|  |  | K.QSMDQLASLPKEQQDATCK.Q | 27 | 58-76 | |  |
|  |  |  |  |  | |  |
|  |  |  |  |  | |  |
| NODE_15_fig|6666666.462.peg.259 | hypothetical protein | -.MIDIFEGSAR.D | 49 | 1-10 | | 49 |
|  |  |  |  |  | |  |
|  |  |  |  |  | |  |
| NODE_18_fig|6666666.462.peg.448 | hypothetical protein | K.SDAKFEK.I | 27 | 140-146 | | 262 |
|  |  | K.FASASAEILASKK.A | 49 | 93-105 | |  |
|  |  | K.FASASAEILASKK.A | 32 | 93-105 | |  |
|  |  | K.SEPGTQAIFFASAK.S | 52 | 50-63 | |  |
|  |  | K.VSLYELLSTPNNK.S | 62 | 22-34 | |  |
|  |  | K.SVDDGAYAQFAFSQK.E | 81 | 155-169 | |  |
|  |  |  |  |  | |  |
|  |  |  |  |  | |  |
| NODE_15_fig|6666666.462.peg.252 | hypothetical protein | R.LILASDNGLLK.F | 42 | 139-149 | | 163 |
|  |  | K.LGQSSEFSGIFR.V | 48 | 64-75 | |  |
|  |  | R.SLAQTSIIEAMIK.G | 62 | 102-114 | |  |
|  |  |  |  |  | |  |
|  |  |  |  |  | |  |
| NODE_34_fig|6666666.462.peg.1007 | hypothetical protein | R.FGEKLR.S | 37 | 457-462 | | 672 |
|  |  | R.KVDFVGR.Y | 25 | 430-436 | |  |
|  |  | M.AAVTVSQIVK.E | 53 | 2-11 | |  |
|  |  | K.NGTAQVVDDK.K | 54 | 90-99 | |  |
|  |  | K.EALSEIKDR.H | 40 | 12-20 | |  |
|  |  | K.YGFTTEESK.K | 28 | 40-48 | |  |
|  |  | K.VQNIKNDLK.N | 26 | 289-297 | |  |
|  |  | K.EGGRNQVMPK.I | 33 | 510-519 | |  |
|  |  | K.ELGEELSTIR.S | 58 | 214-223 | |  |
|  |  | R.IKVTISSGVATR.S | 55 | 477-488 | |  |
|  |  | K.ALAEQGMQLLAR.R | 71 | 125-136 | |  |
|  |  | R.IEEDRTEIIEK.V | 39 | 250-260 | |  |
|  |  | R.ISDIAASSQSNSVK.V | 79 | 275-288 | |  |
|  |  | R.EIQCIEEAYKR.Y | 28 | 376-386 | |  |
|  |  | K.VGSLNNILQNIGER.I | 76 | 261-274 | |  |
|  |  |  |  |  | |  |
|  |  |  |  |  | |  |
| NODE_10_fig|6666666.462.peg.9 | hypothetical protein | R.SIDSFDKK.I | 26 | 45-52 | | 53 |
|  |  | R.LCGLINQR.E | 39 | 27-34 | |  |
|  |  |  |  |  | |  |
|  |  |  |  |  | |  |
| NODE_10_fig|6666666.462.peg.2 | hypothetical protein | K.AKDLVR.V | 28 | 52-57 | | 1116 |
|  |  | R.AGSLNLEK.L | 34 | 342-349 | |  |
|  |  | K.TAFKITR.I | 46 | 241-247 | |  |
|  |  | K.ASSADFIAR.G | 52 | 424-432 | |  |
|  |  | K.SGINMSAVR.D | 61 | 154-162 | |  |
|  |  | K.ACDKIYAK.I | 36 | 40-47 | |  |
|  |  | R.IIKETAAASR.M | 40 | 167-176 | |  |
|  |  | R.IIKETAAASR.M | 56 | 167-176 | |  |
|  |  | R.IGQLVGQMASER.L | 28 | 248-259 | |  |
|  |  | K.INKASSADFIAR.G | 41 | 421-432 | |  |
|  |  | R.GGQIPAPIHSFKN.- | 44 | 433-445 | |  |
|  |  | R.EGDMIEFGGLLGR.A | 52 | 403-415 | |  |
|  |  | R.GESFDVVAETVKK.T | 60 | 228-240 | |  |
|  |  | R.EGDMIEFGGLLGR.A | 36 | 403-415 | |  |
|  |  | K.VRGESFDVVAETVK.K | 60 | 226-239 | |  |
|  |  | K.VRGESFDVVAETVK.K | 24 | 226-239 | |  |
|  |  | R.VSVTPISIIGAATDAK.D | 80 | 75-90 | |  |
|  |  | R.VSVTPISIIGAATDAK.D | 46 | 75-90 | |  |
|  |  | R.VGNEISAELGIPIVNK.R | 76 | 58-73 | |  |
|  |  | R.VGNEISAELGIPIVNKR.V | 74 | 58-74 | |  |
|  |  | R.VGNEISAELGIPIVNKR.V | 26 | 58-74 | |  |
|  |  | K.FGIVDLSLAPTPAVGDSVAR.V | 26 | 264-283 | |  |
|  |  | K.FGIVDLSLAPTPAVGDSVAR.V | 59 | 264-283 | |  |
|  |  | K.GYQKGDEILINSIPQALAQTAK.V | 48 | 121-142 | |  |
|  |  | K.GYQKGDEILINSIPQALAQTAK.V | 57 | 121-142 | |  |
|  |  |  |  |  | |  |
|  |  |  |  |  | |  |
| NODE_10_fig|6666666.462.peg.3 | hypothetical protein | K.ALEWTK.K | 32 | 159-164 | | 163 |
|  |  | K.GIEKLEAR.N | 51 | 190-197 | |  |
|  |  | K.KQQEIADK.A | 57 | 151-158 | |  |
|  |  | K.FTDVVAAIGAFMK.L | 50 | 123-135 | |  |
|  |  | K.QQEIADKALEWTK.K | 24 | 152-164 | |  |
|  |  |  |  |  | |  |
|  |  |  |  |  | |  |
| NODE_10_fig|6666666.462.peg.7 | hypothetical protein | K.INLLSFAK.I | 26 | 11-18 | | 60 |
|  |  | K.NALSGYCEVNDAK.M | 46 | 122-134 | |  |
|  |  |  |  |  | |  |
|  |  |  |  |  | |  |
| NODE_23_fig|6666666.462.peg.714 | hypothetical protein | K.ISGKDMSK.F | 34 | 51-58 | | 34 |
|  |  |  |  |  | |  |
|  |  |  |  |  | |  |
| NODE_23_fig|6666666.462.peg.712 | hypothetical protein | K.SVLDIIDSK.T | 28 | 1470-1478 | | 314 |
|  |  | K.IAGKDGDIVK.L | 21 | 1505-1514 | | |
|  |  | K.ISILNPLTNK.Q | 42 | 780-789 | |  |
|  |  | K.SFEILQLGSK.S | 48 | 1836-1845 | | |
|  |  | K.STQAVSIGLDAK.S | 61 | 1846-1857 | | |
|  |  | K.NGFLGGAQLNSLPK.T | 67 | 227-240 | |  |
|  |  | K.IITSIGVDIPDVAK.N | 47 | 247-260 | |  |
|  |  | K.SSQNDSDYKIIGSK.D | 38 | 1319-1332 | | |
|  |  | R.NLSYSGSFNVAQNTGSK.N | 67 | 1065-1081 | | |
|  |  |  |  |  | |  |
|  |  |  |  |  | |  |
| NODE_23_fig|6666666.462.peg.710 | hypothetical protein | K.KVNGCIK.K | 21 | 413-419 | | 21 |
|  |  |  |  |  | |  |
|  |  |  |  |  | |  |
| NODE_9_fig|6666666.462.peg.1866 | hypothetical protein | R.SLYPAFDSVETNEQK.Q | 37 | 81-95 | | 37 |
|  |  |  |  |  | |  |
|  |  |  |  |  | |  |
| NODE_36_fig|6666666.462.peg.1097 | hypothetical protein | K.LKDEIYAQILK.Y | 53 | 53-63 | | 53 |
|  |  |  |  |  | |  |
|  |  |  |  |  | |  |
| NODE_7_fig|6666666.462.peg.1741 | hypothetical protein | R.TSDEYLTAVTLSLR.F | 83 | 159-172 | | 83 |
|  |  |  |  |  | |  |
|  |  |  |  |  | |  |
| NODE_7_fig|6666666.462.peg.1743 | hypothetical protein | R.LGEGVVELR.A | 62 | 421-429 | | 149 |
|  |  | K.IVMDSDSLLR.V | 28 | 679-688 | |  |
|  |  | K.SSELTIGKDAK.L | 30 | 612-622 | |  |
|  |  | K.ALQNSNELMNSR.L | 67 | 904-915 | |  |
|  |  |  |  |  | |  |
|  |  |  |  |  | |  |
| NODE_7_fig|6666666.462.peg.1746 | hypothetical protein | K.LNDDAKVVLEGK.I | 52 | 37-48 | | 52 |
|  |  |  |  |  | |  |
|  |  |  |  |  | |  |
| NODE_12_fig|6666666.462.peg.135 | hypothetical protein | K.QVVEASK.W | 33 | 43-49 | | 101 |
|  |  | R.LLSLANR.A | 21 | 95-101 | |  |
|  |  | R.LTSVSAKL.- | 41 | 228-235 | |  |
|  |  | K.GLLLGSEIK.F | 29 | 194-202 | |  |
|  |  |  |  |  | |  |
|  |  |  |  |  | |  |
| NODE_15_fig|6666666.462.peg.319 | hypothetical protein | K.FELIGVR.A | 23 | 64-70 | | 100 |
|  |  | K.VYHDGKELEGANASK.M | 55 | 186-200 | |  |
|  |  |  |  |  | |  |
|  |  |  |  |  | |  |
| NODE_40_fig|6666666.462.peg.1169 | hypothetical protein | R.HAVVFK.D | 29 | 61-66 | | 782 |
|  |  | R.FGGNEATAK.S | 45 | 146-154 | |  |
|  |  | K.YFTENTR.H | 34 | 54-60 | |  |
|  |  | K.IEVTWNGAPK.S | 44 | 117-126 | |  |
|  |  | K.NGPETHVEGDK.I | 61 | 104-114 | |  |
|  |  | K.IGAKPGNNMTLK.N | 49 | 92-103 | |  |
|  |  | K.IGAKPGNNMTLK.N | 27 | 92-103 | |  |
|  |  | K.VNGKYFTENTR.H | 29 | 50-60 | |  |
|  |  | K.VNGKYFTENTR.H | 30 | 50-60 | |  |
|  |  | K.NGPETHVEGDKIK.I | 33 | 104-116 | |  |
|  |  | K.NGPETHVEGDKIK.I | 28 | 104-116 | |  |
|  |  | K.SYDINEVITDSNGK.Q | 87 | 127-140 | |  |
|  |  | K.DVLPPDGTFVAISFR.V | 27 | 195-209 | |  |
|  |  | K.SYDINEVITDSNGKQIDMR.F | 93 | 127-145 | |  |
|  |  | K.SYDINEVITDSNGKQIDMR.F | 35 | 127-145 | |  |
|  |  | K.SYDINEVITDSNGKQIDMR.F | 35 | 127-145 | |  |
|  |  | K.SYDINEVITDSNGKQIDMR.F | 45 | 127-145 | |  |
|  |  |  |  |  | |  |
|  |  |  |  |  | |  |
| NODE_15_fig|6666666.462.peg.261 | hypothetical protein | K.VEAVYLDQPYSK.A | 33 | 52-63 | | 33 |
|  |  |  |  |  | |  |
|  |  |  |  |  | |  |
| NODE_34_fig|6666666.462.peg.1018 | hypothetical protein | K.SASVFLQNPK.N | 44 | 132-141 | | 108 |
|  |  | K.KSASVFLQNPK.N | 51 | 131-141 | |  |
|  |  |  |  |  | |  |
|  |  |  |  |  | |  |
| NODE_18_fig|6666666.462.peg.478 | hypothetical protein | R.ANLASSFSLKEQK.G | 51 | 43-55 | | 51 |
|  |  |  |  |  | |  |
|  |  |  |  |  | |  |
| NODE_18_fig|6666666.462.peg.474 | hypothetical protein | K.SEPIEVLTPNK.Y | 27 | 37-47 | | 27 |
|  |  |  |  |  | |  |
|  |  |  |  |  | |  |
| NODE_2_fig|6666666.462.peg.909 | hypothetical protein | -.MKISENLSNLK.N | 50 | 1-11 | | 113 |
|  |  | -.MKISENLSNLK.N | 25 | 1-11 | |  |
|  |  | K.NDLDSSATGSFLQNLEK.A | 64 | 20-36 | |  |
|  |  |  |  |  | |  |
|  |  |  |  |  | |  |
| NODE_40_fig|6666666.462.peg.1163 | hypothetical protein | R.ASNYALVK.L | 35 | 45-52 | | 334 |
|  |  | K.SIDYIVLVK.R | 38 | 28-36 | |  |
|  |  | R.SDFNSDKTR.L | 49 | 155-163 | |  |
|  |  | K.SIDYIVLVKR.F | 28 | 28-37 | |  |
|  |  | R.TDEEILYNFSK.N | 61 | 192-202 | |  |
|  |  | R.DIPLCIPENLTNIER.Q | 48 | 108-122 | |  |
|  |  | R.SLSISDSENPDKIYCK.G | 60 | 128-143 | |  |
|  |  |  |  |  | |  |
|  |  |  |  |  | |  |
| NODE_9_fig|6666666.462.peg.1874 | hypothetical protein | M.AQTIQEIIDK.A | 36 | 2-11 | | 138 |
|  |  | K.VSDFFGFVVSK.I | 33 | 202-212 | |  |
|  |  | K.FSTIYNEQNGEVK.I | 47 | 99-111 | |  |
|  |  | K.KFSTIYNEQNGEVK.I | 61 | 98-111 | |  |
|  |  |  |  |  | |  |
|  |  |  |  |  | |  |
| NODE_51_fig|6666666.462.peg.1349 | hypothetical protein | R.NLADEIIR.S | 34 | 308-315 | | 46 |
|  |  | R.LFQNEIPKPK.L | 25 | 253-262 | |  |
|  |  |  |  |  | |  |
|  |  |  |  |  | |  |
| NODE_15_fig|6666666.462.peg.304 | hypothetical protein | K.NGITEISFDKICK.N | 47 | 36-48 | | 47 |
|  |  |  |  |  | |  |
|  |  |  |  |  | |  |
| NODE_15_fig|6666666.462.peg.279 | hypothetical protein | R.SPSCGSGQIYDGSFSK.K | 49 | 101-116 | | 49 |
|  |  |  |  |  | |  |
|  |  |  |  |  | |  |
| NODE_18_fig|6666666.462.peg.468 | hypothetical protein | R.LSDYVK.A | 21 | 193-198 | | 169 |
|  |  | R.LLSADEKSK.F | 34 | 430-438 | |  |
|  |  | K.EQILDELR.S | 32 | 91-98 | |  |
|  |  | K.GAGTIHTMQGK.E | 31 | 1019-1029 | | |
|  |  | R.LDVRDELNQR.A | 21 | 274-283 | |  |
|  |  | K.SNFVFNAINGVK.I | 39 | 464-475 | |  |
|  |  | R.ADKAQNIGTYIK.G | 41 | 904-915 | |  |
|  |  | K.SEIAGLESRAAELNTK.I | 28 | 673-688 | |  |
|  |  |  |  |  | |  |
|  |  |  |  |  | |  |
| NODE_26_fig|6666666.462.peg.753 | hypothetical protein | K.VLSSFVGLK.R | 23 | 166-174 | | 23 |
|  |  |  |  |  | |  |
|  |  |  |  |  | |  |
| NODE_6_fig|6666666.462.peg.1581 | hypothetical protein | K.GVVSEIK.D | 24 | 82-88 | | 1124 |
|  |  | K.ISAIITR.E | 40 | 105-111 | |  |
|  |  | K.IAGGEVLK.A | 54 | 29-36 | |  |
|  |  | K.ASSVIVSK.D | 51 | 61-68 | |  |
|  |  | K.QAIFLFK.A | 46 | 54-60 | |  |
|  |  | K.LSATNQIK.G | 31 | 74-81 | |  |
|  |  | K.ATQIIVGVK.- | 52 | 131-139 | |  |
|  |  | K.ATITVDSEK.A | 46 | 37-45 | |  |
|  |  | R.ESVQNLALK.V | 40 | 112-120 | |  |
|  |  | R.TGAVNSLIAGK.I | 80 | 18-28 | |  |
|  |  | K.VGDKVTAIIK.A | 52 | 121-130 | |  |
|  |  | R.NQLNVEISEVR.T | 61 | 7-17 | |  |
|  |  | K.ASSVIVSKDDSIK.L | 34 | 61-73 | |  |
|  |  | R.ESVQNLALKVGDK.V | 56 | 112-124 | |  |
|  |  | K.DDSIKLSATNQIK.G | 66 | 69-81 | |  |
|  |  | K.DDSIKLSATNQIK.G | 53 | 69-81 | |  |
|  |  | K.ATITVDSEKALDLK.V | 24 | 37-50 | |  |
|  |  | K.DGAVNAEVIIDANGSK.I | 81 | 89-104 | |  |
|  |  | K.IAGGEVLKATITVDSEK.A | 52 | 29-45 | |  |
|  |  | K.ISAIITRESVQNLALK.V | 40 | 105-120 | |  |
|  |  | R.TGAVNSLIAGKIAGGEVLK.A | 62 | 18-36 | |  |
|  |  |  |  |  | |  |
|  |  |  |  |  | |  |
| NODE_6_fig|6666666.462.peg.1588 | hypothetical protein | K.ALATPFPQIINK.- | 31 | 170-181 | | 231 |
|  |  | K.QGGNLVLFTIQR.R | 62 | 133-144 | |  |
|  |  | K.KALATPFPQIINK.- | 38 | 169-181 | |  |
|  |  | K.KALATPFPQIINK.- | 49 | 169-181 | |  |
|  |  | R.FATNFTIESQDFSK.K | 70 | 155-168 | |  |
|  |  |  |  |  | |  |
|  |  |  |  |  | |  |
| NODE_9_fig|6666666.462.peg.1848 | hypothetical protein | K.IGLNITK.N | 49 | 150-156 | | 56 |
|  |  | K.AFGTNFAR.F | 20 | 122-129 | |  |
|  |  |  |  |  | |  |
|  |  |  |  |  | |  |
| NODE_9_fig|6666666.462.peg.1841 | hypothetical protein | R.AYAFNLGEGGSLNTK.A | 85 | 200-214 | | 113 |
|  |  | R.VFAYENSPDLAPDTAVR.S | 41 | 108-124 | |  |
|  |  |  |  |  | |  |
|  |  |  |  |  | |  |
| NODE_9_fig|6666666.462.peg.1843 | hypothetical protein | R.EQSELVAR.A | 30 | 42-49 | | 30 |
|  |  |  |  |  | |  |
|  |  |  |  |  | |  |
| NODE_9_fig|6666666.462.peg.1842 | hypothetical protein | R.TVNELETQTEIALEQISK.R | 58 | 42-59 | | 58 |
|  |  |  |  |  | |  |
|  |  |  |  |  | |  |
| NODE_22_fig|6666666.462.peg.647 | hypothetical protein | K.FIIADITLAK.D | 22 | 68-77 | | 55 |
|  |  | K.FEIDAAILPNAIKE.- | 35 | 108-121 | |  |
|  |  | K.SEDEISSGRQNLFK.F | 24 | 19-32 | |  |
|  |  |  |  |  | |  |
|  |  |  |  |  | |  |
| NODE_19_fig|6666666.462.peg.502 | hypothetical protein | R.QTSADALKPVLLSEAR.N | 23 | 133-148 | | 23 |
|  |  |  |  |  | |  |
|  |  |  |  |  | |  |
| NODE_19_fig|6666666.462.peg.506 | hypothetical protein | K.NDFLIK.S | 25 | 90-95 | | 413 |
|  |  | K.CGTLVIR.L | 37 | 116-122 | |  |
|  |  | K.VANYLGVVR.A | 58 | 67-75 | |  |
|  |  | K.SSLNYGGEELAK.I | 43 | 29-40 | |  |
|  |  | K.FLEQNESAFLK.M | 43 | 136-146 | |  |
|  |  | K.SVNDLTQSFDASLER.V | 93 | 96-110 | |  |
|  |  | K.SVNDLTQSFDASLER.V | 22 | 96-110 | |  |
|  |  |  |  |  | |  |
|  |  |  |  |  | |  |
| NODE_19_fig|6666666.462.peg.507 | hypothetical protein | K.LVTHLK.G | 23 | 494-499 | | 243 |
|  |  | K.GYIVGTAK.I | 26 | 500-507 | |  |
|  |  | R.DLMLLAAK.D | 28 | 164-171 | |  |
|  |  | K.AYESVANK.Q | 48 | 309-316 | |  |
|  |  | R.QSLIVHAK.A | 25 | 301-308 | |  |
|  |  | K.NTLEEIAAK.S | 35 | 461-469 | |  |
|  |  | K.SYDELTYK.S | 38 | 10-17 | |  |
|  |  | K.DNADIIFANK.F | 42 | 430-439 | |  |
|  |  | K.IASMLYLLEEI.- | 22 | 508-518 | |  |
|  |  | K.LGANYEDKIPVK.L | 29 | 198-209 | |  |
|  |  | K.LGANYEDKIPVK.L | 40 | 198-209 | |  |
|  |  |  |  |  | |  |
|  |  |  |  |  | |  |
| NODE_2_fig|6666666.462.peg.880 | hypothetical protein | R.AINEEASPVFDQSR.C | 61 | 316-329 | | 61 |
|  |  |  |  |  | |  |
|  |  |  |  |  | |  |
| NODE_12_fig|6666666.462.peg.157 | hypothetical protein | K.LFTPLK.I | 24 | 5-10 | | 388 |
|  |  | K.LARELEK.N | 26 | 233-239 | |  |
|  |  | R.LLLEILR.E | 22 | 195-201 | |  |
|  |  | K.AGANIAVGVR.I | 49 | 205-214 | |  |
|  |  | K.FSDEYATPK.E | 52 | 123-131 | |  |
|  |  | K.FTPLYQAGYAK.A | 43 | 262-272 | |  |
|  |  | K.NVVNIPVFAVGLITK.A | 48 | 276-290 | |  |
|  |  | R.AIGGVGMIIVEATAVEAR.G | 80 | 49-66 | |  |
|  |  | R.AIGGVGMIIVEATAVEAR.G | 32 | 49-66 | |  |
|  |  | K.ASECEALLLGDVCDGVALGR.E | 62 | 291-310 | |  |
|  |  |  |  |  | |  |
