# Supplementary material for: Sequencing and Validation of the Genome of a Campylobacter concisus Reveals Intra-Species Diversity
Source: PLoS One. 2011 Jul 29;6(7):e22170. doi: 10.1371/journal.pone.0022170 (PMC3146479; doi:10.1371/journal.pone.0022170)
Supplement: Table S3 — 49 proteins encoded from the 138 genes specific to C. concisus UNSWCD (absent in the BAA-1457 reference genome) were identified using Orbitrap MS analysis of the UNSWCD strain. The complete list of identifications of UNSWCD proteins contained 1369 proteins. (DOC) [file pone.0022170.s006.doc]

| **Protein ID** | **Description** | **Peptides** | **Score** | **Mapping** | **Combined score** |
| --- | --- | --- | --- | --- | --- |
|  |  |  |  |  |  |
| NODE_12_fig|6666666.462.peg.127 | hypothetical protein | R.NPAEDQNNTQR.H | 47 | 129-139 | 47 |
|  |  |  |  |  |  |
|  |  |  |  |  |  |
|  |  |  |  |  |  |
|  |  | Top Hit: Campylobacter jejuni 14/23 (61%) | | |  |
|  |  |  |  |  |  |
| NODE_12_fig|6666666.462.peg.154 | nucleotidyltransferase family protein | K.LGLFGSYAK.G | 40 | 26-34 | 77 |
|  |  | K.SEILNFLSSQK.E | 50 | 5-15 |  |
|  |  |  |  |  |  |
|  |  |  |  |  |  |
|  |  |  |  |  |  |
|  |  | Top Hit: Campylobacter gracilis 69/106 (66%) | | |  |
|  |  |  |  |  |  |
| NODE_15_fig|6666666.462.peg.252 | hypothetical protein | R.LILASDNGLLK.F | 42 | 139-149 | 163 |
|  |  | K.LGQSSEFSGIFR.V | 48 | 64-75 |  |
|  |  | R.SLAQTSIIEAMIK.G | 62 | 102-114 |  |
|  |  |  |  |  |  |
|  |  |  |  |  |  |
|  |  |  |  |  |  |
|  |  | Top Hit: Campylobacter showae 204/224 (92%) | | | |
|  |  |  |  |  |  |
| NODE_15_fig|6666666.462.peg.349 | putative capK protein | R.NLFISK.S | 23 | 91-96 | 1394 |
|  |  | K.NVIDFK.N | 21 | 63-68 |  |
|  |  | R.SDDILIK.K | 33 | 335-341 |  |
|  |  | K.LYIDVIK.K | 21 | 187-193 |  |
|  |  | K.QFQIIQK.N | 25 | 364-370 |  |
|  |  | R.SDDILIKK.D | 50 | 335-342 |  |
|  |  | K.LYIDVIKK.S | 40 | 187-194 |  |
|  |  | K.EENQIMQTEK.L | 47 | 34-43 |  |
|  |  | K.MFLIEDLLYK.R | 38 | 123-132 |  |
|  |  | K.STFSIPISSISGR.S | 45 | 322-334 |  |
|  |  | R.SYLPGPNDKLYK.H | 23 | 154-165 |  |
|  |  | K.TKEENQIMQTEK.L | 65 | 32-43 |  |
|  |  | K.TKEENQIMQTEK.L | 35 | 32-43 |  |
|  |  | K.ILLYAVNNIEFYK.K | 54 | 47-59 |  |
|  |  | K.TSGSTGEPSEFLTSFK.M | 78 | 107-122 |  |
|  |  | R.LGNVDVLVTVVEQIKR.N | 49 | 400-415 |  |
|  |  | R.LGNVDVLVTVVEQIKR.N | 30 | 400-415 |  |
|  |  | K.NFTQDEIYKLPIIDK.A | 62 | 69-83 |  |
|  |  | R.YKTGDIAIENTDVDYK.C | 72 | 302-317 |  |
|  |  | K.IDQIDVISTSSETMLSSYR.K | 84 | 221-239 |  |
|  |  |  |  |  |  |
|  |  |  |  |  |  |
|  |  |  |  |  |  |
|  |  | Top Hit: Campylobacter gracilis 199/410 (49%) | | |  |
|  |  |  |  |  |  |
| NODE_16_fig|6666666.462.peg.382 | hypothetical protein | K.AVIAYIK.L | 24 | 210-216 | 2850 |
|  |  | K.AYFLQR.K | 32 | 426-431 |  |
|  |  | K.LAERTQK.S | 34 | 564-570 |  |
|  |  | K.GLSVDEQIK.S | 29 | 125-133 |  |
|  |  | R.SLFEFNLK.D | 23 | 300-307 |  |
|  |  | R.LTLINEINK.T | 52 | 436-444 |  |
|  |  | R.GFAVVADEVR.K | 49 | 553-562 |  |
|  |  | R.INFEEATKK.D | 39 | 329-337 |  |
|  |  | R.INFEEATKK.D | 30 | 329-337 |  |
|  |  | K.MMSGYAFGVK.I | 40 | 282-291 |  |
|  |  | K.FSILSGNLQK.R | 46 | 238-247 |  |
|  |  | K.RINFEEATK.K | 22 | 328-336 |  |
|  |  | R.EIYEFLDTK.T | 37 | 249-257 |  |
|  |  | K.TQNFQDTGKK.F | 28 | 228-237 |  |
|  |  | K.TQNFQDTGKK.F | 29 | 228-237 |  |
|  |  | R.GFAVVADEVRK.L | 57 | 553-563 |  |
|  |  | R.GFAVVADEVRK.L | 31 | 553-563 |  |
|  |  | K.EISENFKDVK.T | 20 | 466-475 |  |
|  |  | R.SLFEFNLKDK.N | 36 | 300-309 |  |
|  |  | K.NANAISSQIESK.T | 60 | 620-631 |  |
|  |  | K.FSILSGNLQKR.E | 34 | 238-248 |  |
|  |  | K.REIYEFLDTK.T | 21 | 248-257 |  |
|  |  | K.IAIYENILEKL.- | 63 | 645-655 |  |
|  |  | K.SSVPIISGVSGTLK.E | 53 | 452-465 |  |
|  |  | R.SSFISNTILDLVK.N | 71 | 607-619 |  |
|  |  | K.YLDDRIVSNSEK.I | 60 | 491-502 |  |
|  |  | K.YLDDRIVSNSEK.I | 24 | 491-502 |  |
|  |  | K.GLSVDEQIKSIER.E | 51 | 125-137 |  |
|  |  | R.VENINENTKDTSK.D | 47 | 586-598 |  |
|  |  | R.AGEAGRGFAVVADEVR.K | 43 | 547-562 |  |
|  |  | R.AGEAGRGFAVVADEVR.K | 41 | 547-562 |  |
|  |  | K.SVVQRVENINENTK.D | 65 | 581-594 |  |
|  |  | K.TNLSNVNDKFGDFSK.Y | 30 | 476-490 |  |
|  |  | K.TNLSNVNDKFGDFSK.Y | 81 | 476-490 |  |
|  |  | R.VFCDIDNAEELIVVK.R | 81 | 313-327 |  |
|  |  | K.QEGEFNDNYVNSFTK.H | 59 | 405-419 |  |
|  |  | R.LTLINEINKTMLTQIK.S | 61 | 436-451 |  |
|  |  | R.LTLINEINKTMLTQIK.S | 42 | 436-451 |  |
|  |  | K.VVIGYAMPNVNLANAASSIK.S | 31 | 41-60 |  |
|  |  | K.VVIGYAMPNVNLANAASSIK.S | 110 | 41-60 |  |
|  |  | K.VVIGYAMPNVNLANAASSIK.S | 75 | 41-60 |  |
|  |  | K.LDFSGTYIYNNSQIVQSK.A | 82 | 192-209 |  |
|  |  |  |  |  |  |
|  |  |  |  |  |  |
|  |  |  |  |  |  |
|  |  | Top Hit: Campylobacter jejuni 217/526 (42%) | | |  |
|  |  |  |  |  |  |
| NODE_18_fig|6666666.462.peg.418 | hypothetical protein | R.ADEALISQIVAISK.A | 57 | 8-21 | 89 |
|  |  | K.LIIDDGYADELNAR.A | 45 | 31-44 |  |
|  |  |  |  |  |  |
|  |  |  |  |  |  |
|  |  |  |  |  |  |
|  |  | Top Hit: Campylobacter gracilis 49/82 (60%) | | |  |
|  |  |  |  |  |  |
| NODE_18_fig|6666666.462.peg.457 | hypothetical protein | K.GLVEIGK.F | 22 | 17-23 | 115 |
|  |  | K.DIIVGIGK.G | 26 | 9-16 |  |
|  |  | R.QLDDDELKK.I | 44 | 41-49 |  |
|  |  | K.YGSGIDKDIAK.T | 27 | 53-63 |  |
|  |  | K.YGSGIDKDIAK.T | 47 | 53-63 |  |
|  |  |  |  |  |  |
|  |  |  |  |  |  |
|  |  |  |  |  |  |
|  |  | Top Hit: Campylobacter curvus 21/53 (40%) | | |  |
|  |  |  |  |  |  |
| NODE_40_fig|6666666.462.peg.1159 | Sodium/solute symporter | K.GNIPAELAK.T | 23 | 286-294 | 23 |
|  |  |  |  |  |  |
|  |  |  |  |  |  |
|  |  |  |  |  |  |
|  |  | Top Hit: Campylobacter rectus 19/45 (43%) | | |  |
|  |  |  |  |  |  |
| NODE_40_fig|6666666.462.peg.1160 | hypothetical protein | -.MMSNEEIAQK.F | 57 | 1-10 | 210 |
|  |  | -.MMSNEEIAQK.F | 47 | 1-10 |  |
|  |  | R.ALDQILEFKES.- | 42 | 130-140 |  |
|  |  | K.ICPCAISDSMR.A | 25 | 119-129 |  |
|  |  | K.FAEQNCAQMILAR.Y | 55 | 11-23 |  |
|  |  | R.YAQHLGANEAQLMK.L | 37 | 24-37 |  |
|  |  |  |  |  |  |
|  |  |  |  |  |  |
|  |  |  |  |  |  |
|  |  | Top Hit: Campylobacter gracilis 15/39 (39%) | | |  |
|  |  |  |  |  |  |
| NODE_42_fig|6666666.462.peg.1217 | hypothetical protein | K.RSPLVVAK.E | 23 | 16-23 | 23 |
|  |  |  |  |  |  |
|  |  |  |  |  |  |
|  |  |  |  |  |  |
|  |  | Top Hit: Campylobacter coli 12/25 (48%) | | |  |
|  |  |  |  |  |  |
| NODE_15_fig|6666666.462.peg.264 | Na | K.VKESEQEDVK.A | 34 | 389-398 | 34 |
|  |  |  |  |  |  |
|  |  |  |  |  |  |
|  |  |  |  |  |  |
|  |  | Top Hit: Campylobacter curvus 388/477 (82%) | | |  |
|  |  |  |  |  |  |
| NODE_15_fig|6666666.462.peg.341 | Glycosyltransferase | R.FDIIVK.A | 36 | 209-214 | 363 |
|  |  | K.FVQSWIK.S | 33 | 162-168 |  |
|  |  | K.ESYYVSVGR.L | 37 | 194-202 |  |
|  |  | K.LIIIGDGSQR.K | 37 | 224-233 |  |
|  |  | K.ILVVDWLDK.Y | 27 | 4-12 |  |
|  |  | K.KPDFIISNSK.F | 33 | 152-161 |  |
|  |  | K.LIISSSHAISK.Y | 46 | 89-99 |  |
|  |  | K.KESYYVSVGR.L | 26 | 193-202 |  |
|  |  | K.KLIIIGDGSQR.K | 49 | 223-233 |  |
|  |  | K.LIIIGDGSQRK.Y | 39 | 224-234 |  |
|  |  | K.INHTLLNVLGR.R | 35 | 55-65 |  |
|  |  | K.FDIEASKKPDFIISNSK.F | 23 | 145-161 |  |
|  |  |  |  |  |  |
|  |  |  |  |  |  |
|  |  |  |  |  |  |
|  |  | Top Hit: Campylobacter gracilis 169/380 (45%) | | |  |
|  |  |  |  |  |  |
| NODE_15_fig|6666666.462.peg.342 | Glycosyl transferase2C group 1 | K.NLEILVK.A | 23 | 197-203 | 951 |
|  |  | -.MLFVNAR.F | 37 | 1-7 |  |
|  |  | -.MLFVNAR.F | 22 | 1-7 |  |
|  |  | K.AINNINNK.N | 28 | 204-211 |  |
|  |  | R.TKGFENIK.R | 22 | 330-337 |  |
|  |  | K.IINLINKL.- | 45 | 348-355 |  |
|  |  | R.KNLEILVK.A | 31 | 196-203 |  |
|  |  | K.QEILSYYK.N | 30 | 142-149 |  |
|  |  | R.FSWEESAQK.I | 52 | 339-347 |  |
|  |  | R.YILYVGSVSK.R | 39 | 185-194 |  |
|  |  | R.SSDIANNEYVK.F | 39 | 230-240 |  |
|  |  | R.FLTQDITGVQR.F | 51 | 8-18 |  |
|  |  | K.ILLYIVGGCDQK.I | 75 | 215-226 |  |
|  |  | K.ILKDDVQFIAPK.N | 30 | 30-41 |  |
|  |  | K.ILKDDVQFIAPK.N | 69 | 30-41 |  |
|  |  | K.IELVLDDKNLQNELR.T | 36 | 315-329 |  |
|  |  | R.NIDVVYNSYIAPNNNK.I | 81 | 157-172 |  |
|  |  |  |  |  |  |
|  |  |  |  |  |  |
|  |  |  |  |  |  |
|  |  | Top Hit: Campylobacter rectus 117/389 (31%) | | |  |
|  |  |  |  |  |  |
| NODE_23_fig|6666666.462.peg.694 | endonuclease2C probable | R.QILCLLLSR.I | 23 | 207-215 | 23 |
|  |  |  |  |  |  |
|  |  |  |  |  |  |
|  |  |  |  |  |  |
|  |  | Top Hit: Campylobacter rectus 390/580 (68%) | | |  |
|  |  |  |  |  |  |
| NODE_12_fig|6666666.462.peg.131 | sugar transferase | K.IYGDKK.L | 22 | 120-125 | 112 |
|  |  | K.NNSYVK.A | 29 | 272-277 |  |
|  |  | K.VLHNAANK.A | 24 | 156-163 |  |
|  |  | R.NDGIILANGK.Y | 34 | 73-82 |  |
|  |  | K.IFNTINGFLSNK.K | 41 | 324-335 |  |
|  |  | K.SVYMPAISSRPNLK.N | 28 | 258-271 |  |
|  |  |  |  |  |  |
|  |  |  |  |  |  |
|  |  |  |  |  |  |
|  |  | Top Hit: Campylobacter curvus 148/339 (44%) | | |  |
|  |  |  |  |  |  |
| NODE_12_fig|6666666.462.peg.153 | 2-isopropylmalate synthase | K.IGSILNK.C | 38 | 17-23 | 38 |
|  |  |  |  |  |  |
|  |  |  |  |  |  |
|  |  |  |  |  |  |
|  |  | Top Hit: Campylobacter gracilis 86/115 (75%) | | |  |
|  |  |  |  |  |  |
| NODE_15_fig|6666666.462.peg.336 | hypothetical protein | R.SMQTPR.L | 23 | 184-189 | 1290 |
|  |  | K.NNKIAEK.Q | 23 | 169-175 |  |
|  |  | K.ISVVKDSK.K | 33 | 105-112 |  |
|  |  | K.VINGYMELK.K | 31 | 145-153 |  |
|  |  | K.QQQIEYLR.S | 36 | 176-183 |  |
|  |  | K.FFDIEVIAK.T | 47 | 114-122 |  |
|  |  | K.VINGYMELKK.I | 23 | 145-154 |  |
|  |  | K.GVEGLDYQVQK.I | 47 | 94-104 |  |
|  |  | K.TNDIALKQINK.M | 23 | 123-133 |  |
|  |  | K.TNDIALKQINK.M | 48 | 123-133 |  |
|  |  | K.MVEDLANEHQK.V | 42 | 134-144 |  |
|  |  | K.MVEDLANEHQK.V | 23 | 134-144 |  |
|  |  | K.MVEDLANEHQK.V | 35 | 134-144 |  |
|  |  | K.ELLNLQTEELNK.L | 68 | 240-251 |  |
|  |  | K.QTTNLEQYATLAK.D | 75 | 193-205 |  |
|  |  | K.IAEKQQQIEYLR.S | 44 | 172-183 |  |
|  |  | R.DISTNDKLLFLEK.E | 35 | 227-239 |  |
|  |  | R.DISTNDKLLFLEK.E | 47 | 227-239 |  |
|  |  | K.IQLANIDSQINFLK.N | 81 | 155-168 |  |
|  |  | R.LDKQTTNLEQYATLAK.D | 53 | 190-205 |  |
|  |  | R.LDKQTTNLEQYATLAK.D | 84 | 190-205 |  |
|  |  |  |  |  |  |
|  |  |  |  |  |  |
|  |  |  |  |  |  |
|  |  | Top Hit: Campylobacter hominis 120/311 (39%) | | | |
|  |  |  |  |  |  |
| NODE_15_fig|6666666.462.peg.337 | Undecaprenyl-phosphate galactosephosphotransferase | R.SDVDFETR.A | 50 | 390-397 | 191 |
|  |  | K.ANPDEEEYYK.V | 22 | 304-313 |  |
|  |  | R.LNVQNLNELIER.N | 62 | 173-184 |  |
|  |  | K.TSLDELPQILNVLK.G | 54 | 334-347 |  |
|  |  |  |  |  |  |
|  |  |  |  |  |  |
|  |  |  |  |  |  |
|  |  | Top Hit: Campylobacter curvus 231/376 (62%) | | |  |
|  |  |  |  |  |  |
| NODE_15_fig|6666666.462.peg.343 | hypothetical protein | R.IFQNINIK.V | 28 | 321-328 | 46 |
|  |  | K.INIFNSDINNNGAMSR.E | 32 | 266-281 |  |
|  |  |  |  |  |  |
|  |  |  |  |  |  |
|  |  |  |  |  |  |
|  |  | Top Hit: Campylobacter gracilis 29/86 (34%) | | |  |
|  |  |  |  |  |  |
| NODE_15_fig|6666666.462.peg.344 | Glycosyltransferase | K.EFLQDIESY.- | 31 | 370-378 | 84 |
|  |  | R.NVYILGAMSYSR.V | 45 | 268-279 |  |
|  |  | K.TLPQELKDIPEPR.I | 34 | 210-222 |  |
|  |  |  |  |  |  |
|  |  |  |  |  |  |
|  |  |  |  |  |  |
|  |  | Top Hit: Campylobacter gracilis 293/373 (79%) | | |  |
|  |  |  |  |  |  |
| NODE_15_fig|6666666.462.peg.345 | hypothetical protein | R.FTVDITKPNK.E | 45 | 396-405 | 219 |
|  |  | K.FSPNGEFLSGK.Q | 57 | 421-431 |  |
|  |  | K.ELNLSNIDPNVAK.Y | 34 | 63-75 |  |
|  |  | R.DPGTYLYTPIPGIR.N | 33 | 584-597 |  |
|  |  | K.KPIASAVELSELLYQK.S | 77 | 502-517 |  |
|  |  |  |  |  |  |
|  |  |  |  |  |  |
|  |  |  |  |  |  |
|  |  | Top Hit: Campylobacter gracilis 530/695 (77%) | | |  |
|  |  |  |  |  |  |
| NODE_23_fig|6666666.462.peg.660 | hypothetical protein | K.IGLVFNQR.W | 41 | 139-146 | 113 |
|  |  | K.FISQNEAIFEEGESK.I | 85 | 196-210 |  |
|  |  |  |  |  |  |
|  |  |  |  |  |  |
|  |  |  |  |  |  |
|  |  | Top Hit: Campylobacter hominis 25/108 (24%) | | |  |
|  |  |  |  |  |  |
| NODE_23_fig|6666666.462.peg.697 | hypothetical protein | K.ILEIIIPTNAGK.Y | 27 | 134-145 | 140 |
|  |  | K.VELENDHAAQDAFGVANGVK.M | 43 | 25-44 |  |
|  |  | K.VELENDHAAQDAFGVANGVK.M | 95 | 25-44 |  |
|  |  |  |  |  |  |
|  |  |  |  |  |  |
|  |  |  |  |  |  |
|  |  | Top Hit: Campylobacter rectus 25/86 (30%) | | |  |
|  |  |  |  |  |  |
| NODE_23_fig|6666666.462.peg.698 | hypothetical protein | R.IIDLCQYASEPLLSR.L | 56 | 107-121 | 64 |
|  |  |  |  |  |  |
|  |  |  |  |  |  |
|  |  |  |  |  |  |
|  |  | Top Hit: Campylobacter rectus 87/145 (60%) | | |  |
|  |  |  |  |  |  |
| NODE_23_fig|6666666.462.peg.699 | hypothetical protein | R.FIEDILSK.E | 24 | 291-298 | 41 |
|  |  | R.FIEDILSKEGSK.Y | 30 | 291-302 |  |
|  |  |  |  |  |  |
|  |  |  |  |  |  |
|  |  |  |  |  |  |
|  |  | Top Hit: Campylobacter jejuni 13/25 (52%) | | |  |
|  |  |  |  |  |  |
| NODE_23_fig|6666666.462.peg.703 | hypothetical protein | K.SMNVLIIK.R | 45 | 226-233 | 207 |
|  |  | K.SLNLFIDEK.R | 40 | 404-412 |  |
|  |  | K.SLKDENIFVLSK.F | 32 | 329-340 |  |
|  |  | R.SLDDSLLLDPELIYK.S | 50 | 314-328 |  |
|  |  |  |  |  |  |
|  |  |  |  |  |  |
|  |  |  |  |  |  |
|  |  | Top Hit: Campylobacter lari 16/38 (43%) | | |  |
|  |  |  |  |  |  |
| NODE_23_fig|6666666.462.peg.708 | hypothetical protein | K.NQAIEQTSNQK.N | 65 | 33-43 | 133 |
|  |  | R.SIAGVMANKVLPVK.T | 48 | 143-156 |  |
|  |  |  |  |  |  |
|  |  |  |  |  |  |
|  |  |  |  |  |  |
|  |  | Top Hit: Campylobacter lari 19/71 (27%) | | |  |
|  |  |  |  |  |  |
| NODE_23_fig|6666666.462.peg.710 | hypothetical protein | K.KVNGCIK.K | 21 | 413-419 | 21 |
|  |  |  |  |  |  |
|  |  |  |  |  |  |
|  |  |  |  |  |  |
|  |  | Top Hit: Campylobacter rectus 191/536 (36%) | | |  |
|  |  |  |  |  |  |
| NODE_26_fig|6666666.462.peg.748 | hypothetical protein | K.TIETEKTK.Q | 23 | 194-201 | 78 |
|  |  | K.GSLTEALSSLVIK.G | 69 | 79-91 |  |
|  |  |  |  |  |  |
|  |  |  |  |  |  |
|  |  |  |  |  |  |
|  |  | Top Hit: Campylobacter rectus 38/98 (39%) | | |  |
|  |  |  |  |  |  |
| NODE_26_fig|6666666.462.peg.757 | ABC-type nitrate/sulfonate/bicarbonate transportsystems2C periplasmic components | K.VTMSPSNVAANLR.N | 38 | 79-91 | 38 |
|  |  |  |  |  |  |
|  |  |  |  |  |  |
|  |  |  |  |  |  |
|  |  | Top Hit: Campylobacter showae 253/321 (79%) | | | |
|  |  |  |  |  |  |
| NODE_34_fig|6666666.462.peg.1049 | Putative ACR protein | K.VCGSCQTR.C | 20 | 95-102 | 173 |
|  |  | K.ELYASGAMLK.V | 41 | 85-94 |  |
|  |  | R.IFLMNDAVDLAR.N | 63 | 55-66 |  |
|  |  | R.IFLMNDAVDLAR.N | 53 | 55-66 |  |
|  |  |  |  |  |  |
|  |  |  |  |  |  |
|  |  |  |  |  |  |
|  |  | Top Hit: Campylobacter rectus 46/80 (58%) | | |  |
|  |  |  |  |  |  |
| NODE_35_fig|6666666.462.peg.1084 | hypothetical protein | M.SYDLQEIILER.T | 52 | 2-12 | 52 |
|  |  |  |  |  |  |
|  |  |  |  |  |  |
|  |  |  |  |  |  |
|  |  | Top Hit: Campylobacter rectus 97/292 (34%) | | |  |
|  |  |  |  |  |  |
| NODE_37_fig|6666666.462.peg.1104 | R.Ecl18kI | R.VLTFEK.L | 27 | 245-250 | 330 |
|  |  | R.NTVLISAK.T | 26 | 175-182 |  |
|  |  | M.ANISLDEYK.N | 47 | 2-10 |  |
|  |  | K.ETYYSDNER.V | 27 | 236-244 |  |
|  |  | K.FVEEYYDER.L | 31 | 291-299 |  |
|  |  | K.EFFLSNASEVVEK.L | 75 | 43-55 |  |
|  |  | M.ANISLDEYKNLVK.E | 52 | 2-14 |  |
|  |  | K.NIKETYYSDNER.V | 59 | 233-244 |  |
|  |  |  |  |  |  |
|  |  |  |  |  |  |
|  |  |  |  |  |  |
|  |  | Top Hit: Campylobacter hominis 17/46 (37%) | | |  |
|  |  |  |  |  |  |
| NODE_40_fig|6666666.462.peg.1157 | hypothetical protein | R.LDEISAYKDK.N | 30 | 83-92 | 535 |
|  |  | K.GADLAAIQADNK.K | 61 | 36-47 |  |
|  |  | K.LAQILSDKGYK.V | 53 | 208-218 |  |
|  |  | K.LAQILSDKGYK.V | 26 | 208-218 |  |
|  |  | K.FGSINAEEFKK.I | 63 | 135-145 |  |
|  |  | K.TIVTHCYSGNR.S | 42 | 194-204 |  |
|  |  | K.IKGADLAAIQADNK.K | 24 | 34-47 |  |
|  |  | K.IANDPNVLIIDVR.E | 24 | 146-158 |  |
|  |  | K.IANDPNVLIIDVR.E | 45 | 146-158 |  |
|  |  | K.HAISIPLEEIEAR.L | 28 | 70-82 |  |
|  |  | K.HAISIPLEEIEAR.L | 44 | 70-82 |  |
|  |  | K.GAISIPDGEPVDNYK.D | 56 | 170-184 |  |
|  |  | K.IANDPNVLIIDVREK.K | 42 | 146-160 |  |
|  |  | K.LSDAEGVKEFSYDLVK.F | 70 | 119-134 |  |
|  |  | K.LSDAEGVKEFSYDLVK.F | 37 | 119-134 |  |
|  |  |  |  |  |  |
|  |  |  |  |  |  |
|  |  |  |  |  |  |
|  |  | Top Hit: Campylobacter gracilis 161/235 (69%) | | |  |
|  |  |  |  |  |  |
| NODE_40_fig|6666666.462.peg.1161 | Radical SAM | K.DIFELAK.S | 23 | 275-281 | 89 |
|  |  | R.LNALGYGK.G | 34 | 153-160 |  |
|  |  | K.RTETMSK.E | 20 | 43-49 |  |
|  |  | R.WFIDEAAK.I | 31 | 82-89 |  |
|  |  |  |  |  |  |
|  |  |  |  |  |  |
|  |  |  |  |  |  |
|  |  | Top Hit: Campylobacter showae 44/151 (30%) | | |  |
|  |  |  |  |  |  |
| NODE_40_fig|6666666.462.peg.1162 | Fe-S oxidoreductase | K.SPYLYANNSPK.K | 27 | 87-97 | 27 |
|  |  |  |  |  |  |
|  |  |  |  |  |  |
|  |  |  |  |  |  |
|  |  | Top Hit: Campylobacter jejuni 42/169 (25%) | | |  |
|  |  |  |  |  |  |
| NODE_40_fig|6666666.462.peg.1163 | hypothetical protein | R.ASNYALVK.L | 35 | 45-52 | 334 |
|  |  | K.SIDYIVLVK.R | 38 | 28-36 |  |
|  |  | R.SDFNSDKTR.L | 49 | 155-163 |  |
|  |  | K.SIDYIVLVKR.F | 28 | 28-37 |  |
|  |  | R.TDEEILYNFSK.N | 61 | 192-202 |  |
|  |  | R.DIPLCIPENLTNIER.Q | 48 | 108-122 |  |
|  |  | R.SLSISDSENPDKIYCK.G | 60 | 128-143 |  |
|  |  |  |  |  |  |
|  |  |  |  |  |  |
|  |  |  |  |  |  |
|  |  | Top Hit: Campylobacter lari 50/194 (26%) | | |  |
|  |  |  |  |  |  |
| NODE_40_fig|6666666.462.peg.1164 | glycosyl transferase2C family 2 | R.REGVIK.T | 24 | 204-209 | 54 |
|  |  | K.NFDLALNLSK.I | 44 | 48-57 |  |
|  |  |  |  |  |  |
|  |  |  |  |  |  |
|  |  |  |  |  |  |
|  |  | Top Hit: Campylobacter showae 18/50 (36%) | | |  |
|  |  |  |  |  |  |
| NODE_40_fig|6666666.462.peg.1166 |  | K.KAQLEANK.A | 34 | 227-234 | 231 |
|  |  | R.ILGRDVVEK.L | 25 | 112-120 |  |
|  |  | K.QITDSVVMLSK.F | 33 | 28-38 |  |
|  |  | K.QITDSVVMLSK.F | 49 | 28-38 |  |
|  |  | K.ALAATDEFFTR.F | 55 | 126-136 |  |
|  |  |  |  |  |  |
|  |  |  |  |  |  |
|  |  |  |  |  |  |
|  |  | Top Hit: Campylobacter fetus 40/145 (28%) | | |  |
|  |  |  |  |  |  |
| NODE_40_fig|6666666.462.peg.1167 | Choline kinase | K.ALILFTR.A | 21 | 4-10 | 21 |
|  |  |  |  |  |  |
|  |  |  |  |  |  |
|  |  |  |  |  |  |
|  |  | Top Hit: Campylobacter gracilis 19/40 (48%) | | |  |
|  |  |  |  |  |  |
| NODE_40_fig|6666666.462.peg.1169 | hypothetical protein | R.HAVVFK.D | 29 | 61-66 | 782 |
|  |  | R.FGGNEATAK.S | 45 | 146-154 |  |
|  |  | K.YFTENTR.H | 34 | 54-60 |  |
|  |  | K.IEVTWNGAPK.S | 44 | 117-126 |  |
|  |  | K.NGPETHVEGDK.I | 61 | 104-114 |  |
|  |  | K.IGAKPGNNMTLK.N | 49 | 92-103 |  |
|  |  | K.IGAKPGNNMTLK.N | 27 | 92-103 |  |
|  |  | K.VNGKYFTENTR.H | 29 | 50-60 |  |
|  |  | K.VNGKYFTENTR.H | 30 | 50-60 |  |
|  |  | K.NGPETHVEGDKIK.I | 33 | 104-116 |  |
|  |  | K.NGPETHVEGDKIK.I | 28 | 104-116 |  |
|  |  | K.SYDINEVITDSNGK.Q | 87 | 127-140 |  |
|  |  | K.DVLPPDGTFVAISFR.V | 27 | 195-209 |  |
|  |  | K.SYDINEVITDSNGKQIDMR.F | 93 | 127-145 |  |
|  |  | K.SYDINEVITDSNGKQIDMR.F | 35 | 127-145 |  |
|  |  | K.SYDINEVITDSNGKQIDMR.F | 35 | 127-145 |  |
|  |  | K.SYDINEVITDSNGKQIDMR.F | 45 | 127-145 |  |
|  |  |  |  |  |  |
|  |  |  |  |  |  |
|  |  |  |  |  |  |
|  |  | Top Hit: Campylobacter jejuni 16/39 (42%) | | |  |
|  |  |  |  |  |  |
| NODE_42_fig|6666666.462.peg.1216 | hypothetical protein | K.KAEIVSQK.N | 35 | 390-397 | 180 |
|  |  | K.NNLNLVIER.N | 54 | 398-406 |  |
|  |  | K.LQEEITNLR.I | 41 | 362-370 |  |
|  |  | K.ELVDRIETDISTLNK.K | 65 | 240-254 |  |
|  |  |  |  |  |  |
|  |  |  |  |  |  |
|  |  |  |  |  |  |
|  |  | Top Hit: Campylobacter curvus 40/147 (28%) | | |  |
|  |  |  |  |  |  |
| NODE_51_fig|6666666.462.peg.1360 | hypothetical protein | K.ITQQLAK.S | 30 | 144-150 | 660 |
|  |  | -.MVNAVESK.I | 25 | 1-8 |  |
|  |  | K.KADFIFSK.Q | 26 | 179-186 |  |
|  |  | R.NQMTMVADIAK.A | 59 | 79-89 |  |
|  |  | K.QIEYETTSDDLAK.I | 67 | 187-199 |  |
|  |  | K.SAYQDLTQGTSIIK.T | 68 | 216-229 |  |
|  |  | K.WLPVAGAAAMAAWSK.I | 40 | 156-170 |  |
|  |  | K.ISDGVAQMQLAADDLK.S | 29 | 200-215 |  |
|  |  | K.ISDGVAQMQLAADDLK.S | 68 | 200-215 |  |
|  |  | K.ITEGINSALTSVINSR.E | 94 | 9-24 |  |
|  |  | K.ISDGVAQMQLAADDLK.S | 61 | 200-215 |  |
|  |  |  |  |  |  |
|  |  |  |  |  |  |
|  |  |  |  |  |  |
|  |  | Top Hit: Campylobacter fetus 31/103 (31%) | | |  |
|  |  |  |  |  |  |
| NODE_60_fig|6666666.462.peg.1490 | heat shock protein G homolog | K.IDNLIR.N | 41 | 168-173 | 1150 |
|  |  | K.LPNFIR.D | 29 | 374-379 |  |
|  |  | K.YTVYTK.T | 22 | 111-116 |  |
|  |  | K.NTPTGHAR.V | 36 | 570-577 |  |
|  |  | -.MNDNKIK.F | 30 | 1-7 |  |
|  |  | K.AANIEILK.Y | 39 | 639-646 |  |
|  |  | -.MNDNKIK.F | 22 | 1-7 |  |
|  |  | K.LLALLVNK.S | 53 | 402-409 |  |
|  |  | K.YAIIGIEK.Q | 35 | 72-79 |  |
|  |  | K.NNFEIFK.G | 28 | 526-532 |  |
|  |  | K.LAAFSLGEK.Y | 27 | 102-110 |  |
|  |  | K.IADENIFK.N | 41 | 294-301 |  |
|  |  | R.VVAIYELEK.L | 37 | 483-491 |  |
|  |  | K.KIADENIFK.N | 22 | 293-301 |  |
|  |  | K.VYIKIDNNK.I | 21 | 45-53 |  |
|  |  | K.LMSGNNILEK.F | 37 | 492-501 |  |
|  |  | K.GWFGSVITPR.Q | 31 | 265-274 |  |
|  |  | K.GMSIDEINHK.Y | 39 | 62-71 |  |
|  |  | K.FIDDLPNDLK.E | 24 | 146-155 |  |
|  |  | K.AIDQDDEFKK.N | 42 | 538-547 |  |
|  |  | R.AETLNVFELVK.E | 32 | 470-480 |  |
|  |  | R.NVYSTLSTQLSR.R | 61 | 177-188 |  |
|  |  | K.TVDNMEFSEIQK.I | 70 | 433-444 |  |
|  |  | K.TVDNMEFSEIQK.I | 74 | 433-444 |  |
|  |  | R.SYADFINIINAEK.K | 78 | 657-669 |  |
|  |  | K.IDNNKIYIIDNGK.G | 26 | 49-61 |  |
|  |  | K.IDNNKIYIIDNGK.G | 57 | 49-61 |  |
|  |  | K.IADENIFKNSGSAR.I | 73 | 294-307 |  |
|  |  | R.AETLNVFELVKER.V | 59 | 470-482 |  |
|  |  | K.QSSTEYENINDTDIK.V | 65 | 596-610 |  |
|  |  | K.ILSTIETVDDNHIELINK.L | 82 | 445-462 |  |
|  |  |  |  |  |  |
|  |  |  |  |  |  |
|  |  |  |  |  |  |
|  |  | Top Hit: Campylobacter showae 68/205 (34%) | | |  |
|  |  |  |  |  |  |
| NODE_6_fig|6666666.462.peg.1543 | hypothetical protein | K.LLNNQFK.Y | 22 | 72-78 | 199 |
|  |  | K.IADMTAAGKK.C | 38 | 15-24 |  |
|  |  | K.GKPAVIKVEK.G | 48 | 42-51 |  |
|  |  | K.GANQNDSLCLK.I | 44 | 52-62 |  |
|  |  | K.FTLVDFVTDTGDTK.G | 75 | 28-41 |  |
|  |  | K.ESETGEYPVSGLSVAF.- | 37 | 88-103 |  |
|  |  |  |  |  |  |
|  |  |  |  |  |  |
|  |  |  |  |  |  |
|  |  | Top Hit: Campylobacter hominis 34/99 (35%) | | |  |
|  |  |  |  |  |  |
| NODE_75_fig|6666666.462.peg.1661 | hypothetical protein | K.NIDTHIEK.Q | 26 | 336-343 | 283 |
|  |  | K.DKEGSEIENNTNLMK.Y | 80 | 130-144 |  |
|  |  | R.IAQVKPSSIILGTSRPK.H | 30 | 58-74 |  |
|  |  | K.TILGIGESTVLDNGNAIPLAK.V | 85 | 158-178 |  |
|  |  |  |  |  |  |
|  |  |  |  |  |  |
|  |  |  |  |  |  |
|  |  | Top Hit: Campylobacter curvus 162/358 (46%) | | |  |
|  |  |  |  |  |  |
| NODE_7_fig|6666666.462.peg.1727 |  | K.SNLGIGQK.V | 33 | 156-163 | 766 |
|  |  | K.EIDTSLIK.L | 33 | 205-212 |  |
|  |  | K.HNTLVASIK.M | 33 | 530-538 |  |
|  |  | K.MPTDLFVGK.S | 25 | 539-547 |  |
|  |  | K.GSNFDEFVK.S | 21 | 175-183 |  |
|  |  | K.IGLTTDFTGK.L | 47 | 330-339 |  |
|  |  | K.ATINSPQELK.Q | 24 | 405-414 |  |
|  |  | K.VTNLFNYSR.G | 38 | 700-708 |  |
|  |  | K.ISDGQTQLKR.V | 41 | 306-315 |  |
|  |  | K.LNNDIYNNEK.G | 46 | 213-222 |  |
|  |  | K.VSDFSDLSFFK.G | 60 | 164-174 |  |
|  |  | K.ATINSPQELKQK.E | 44 | 405-416 |  |
|  |  | R.TLSNTILSENINK.I | 90 | 293-305 |  |
|  |  | K.SSPEQGNTDGEILMR.K | 77 | 257-271 |  |
|  |  | K.IGDLFLVVSNPQLNK.E | 44 | 884-898 |  |
|  |  |  |  |  |  |
|  |  |  |  |  |  |
|  |  |  |  |  |  |
|  |  | Top Hit: Campylobacter upsaliensis 497/1103 (46%) | | | |
|  |  |  |  |  |  |
| NODE_15_fig|6666666.462.peg.347 | HAD-superfamily subfamily IB hydrolase2C TIGR01490 | K.EYLLSSFK.N | 34 | 62-69 | 217 |
|  |  | K.IVYNFIADSK.L | 64 | 49-58 |  |
|  |  | R.SDLPIFNLVGNK.I | 60 | 185-196 |  |
|  |  |  |  |  |  |
|  |  |  |  |  |  |
|  |  |  |  |  |  |
|  |  | Top Hit: Campylobacter gracilis 98/217 (46%) | | |  |
|  |  |  |  |  |  |
| NODE_12_fig|6666666.462.peg.132 | O-antigen ligase | K.ISQGISSNETR.W | 49 | 245-255 | 482 |
|  |  | K.ITSNNPMALFELK.S | 64 | 126-138 |  |
|  |  | K.NIDITTFFPGNSEVR.V | 63 | 281-295 |  |
|  |  |  |  |  |  |
|  |  |  |  |  |  |
|  |  |  |  |  |  |
|  |  | Top Hit: Campylobacter gracilis 29/114 (26%) | | |  |
