# Supplementary material for: Sequencing and Validation of the Genome of a Campylobacter concisus Reveals Intra-Species Diversity
Source: PLoS One. 2011 Jul 29;6(7):e22170. doi: 10.1371/journal.pone.0022170 (PMC3146479; doi:10.1371/journal.pone.0022170)
Supplement: Table S4 — Primer sequences used for PCR confirmation of missing clusters. (DOC) [file pone.0022170.s007.doc]

| Primer name | Primer Sequence | Reference |
| --- | --- | --- |
| F27  R1494 | AGAGTTTGATCCTGGCTCAG  TACGGCTACCTTGTTACGAC | [27] |
| 1420F  1420R | gcatcaacgaatacactatcg  TTTGCGATGTCGTGATAGAGC | This study |
| 2173F  2173R | gaggatatgaagccaagcgat  GCAAAAGTCAAAGCTCTGGC | This study |
| 2301F  2301R | acggcataaatcctatgagg  CAACGATGGCTATTTGAAGC | This study |
| ZotF  ZotR | GATAGCTAGTATCAAGCCCAT  GTTAGCGATTCTTTTGGTGGC | This study |
