# Supplementary material for: Sequencing and Validation of the Genome of a Campylobacter concisus Reveals Intra-Species Diversity
Source: PLoS One. 2011 Jul 29;6(7):e22170. doi: 10.1371/journal.pone.0022170 (PMC3146479; doi:10.1371/journal.pone.0022170)
Supplement: Table S5 — Ortholog IDs across UNSWCD and BAA-1457. (DOC) [file pone.0022170.s008.doc]

| **BAA-1457** | **UNSWCD** |
| --- | --- |
| YP_001467853.1| Holliday junction resolvase | fig|6666666.462.peg.498;Name=Crossover junction endodeoxyribonucleaseRuvC (EC 3.1.22.4);Ontology_term=KEGG_ENZYME:3.1.22.4 |
| YP_001467852.1| mosc domain-containing protein | fig|6666666.462.peg.497;Name=Uncharacterized protein conserved inbacteria |
| YP_001467851.1| acyl-CoA hydrolase | fig|6666666.462.peg.496;Name=hypothetical protein |
| YP_001467850.1| S-ribosylhomocysteinase | fig|6666666.462.peg.495;Name=S-ribosylhomocysteine lyase (EC 4.4.1.21) /Autoinducer-2 production proteinLuxS;Ontology_term=KEGG_ENZYME:4.4.1.21 |
| YP_001467849.1| D12 class N6 adenine-specific | fig|6666666.462.peg.494;Name=DNA modification methylase(Adenine-specific methyltransferase) (EC2.1.1.72);Ontology_term=KEGG_ENZYME:2.1.1.72 |
| YP_001467846.1| FAD-dependent thymidylate | fig|6666666.462.peg.493;Name=Thymidylate synthase thyX (EC2.1.1.-);Ontology_term=KEGG_ENZYME:2.1.1.- |
| YP_001467842.1| citrate transporter | fig|6666666.462.peg.491;Name=Histidine permease YuiF |
| YP_001467841.1| orotate | fig|6666666.462.peg.490;Name=Orotate phosphoribosyltransferase (EC2.4.2.10);Ontology_term=KEGG_ENZYME:2.4.2.10 |
| YP_001467840.1| NAD(P)H-flavin oxidoreductase | fig|6666666.462.peg.488;Name=Putative two-component response regulator |
| YP_001467839.1| hypothetical protein | fig|6666666.462.peg.487;Name=PUTATIVE TWO-COMPONENT SENSOR |
| YP_001467838.1| tRNA synthetase, class II | fig|6666666.462.peg.486;Name=cytochrome c2C putative |
| YP_001467837.1| adenylosuccinate synthetase | fig|6666666.462.peg.485;Name=FKBP-type peptidyl-prolyl cis-transisomerase FkpA precursor (EC5.2.1.8);Ontology_term=KEGG_ENZYME:5.2.1.8 |
| YP_001467836.1| 4Fe-4S ferredoxin iron-sulfur | fig|6666666.462.peg.484;Name=NrfC protein |
| YP_001467835.1| polysulphide reductase, NrfD | fig|6666666.462.peg.483;Name=NrfD protein |
| YP_001467834.1| NAD(FAD)-utilizing | fig|6666666.462.peg.482;Name=NRFI PROTEIN |
| YP_001467833.1| carbamoyl-phosphate synthase | fig|6666666.462.peg.481;Name=hypothetical protein |
| YP_001467832.1| hypothetical protein | fig|6666666.462.peg.480;Name=hypothetical protein |
| YP_001467831.1| NosL protein [Campylobacter | fig|6666666.462.peg.479;Name=Nitrous oxide reductase maturationprotein2C outer-membrane lipoprotein NosL |
| YP_001467830.1| hypothetical protein | fig|6666666.462.peg.478;Name=hypothetical protein |
| YP_001467829.1| | fig|6666666.462.peg.477;Name=ABC transporter2C permease protein |
| YP_001467828.1| | fig|6666666.462.peg.476;Name=ABC-TYPE TRANSPORTER2C ATPASE COMPONENT |
| YP_001467827.1| hypothetical protein | fig|6666666.462.peg.475;Name=membrane protein2C putative |
| YP_001467826.1| hypothetical protein | fig|6666666.462.peg.474;Name=hypothetical protein |
| YP_001467825.1| putative peptide ABC | fig|6666666.462.peg.472;Name=Putative ribosomal pseudouridine synthase(EC 4.2.1.70);Ontology_term=KEGG_ENZYME:4.2.1.70 |
| YP_001467824.1| arabinose 5-phosphate | fig|6666666.462.peg.471;Name=Arabinose 5-phosphate isomerase (EC5.3.1.13);Ontology_term=KEGG_ENZYME:5.3.1.13 |
| YP_001467823.1| peptide ABC transporter, | fig|6666666.462.peg.470;Name=Zn-dependent hydrolase2C RNA-metabolising |
| YP_001467822.1| dimethyladenosine transferase | fig|6666666.462.peg.469;Name=Dimethyladenosine transferase (EC2.1.1.-);Ontology_term=KEGG_ENZYME:2.1.1.- |
| YP_001467821.1| hypothetical protein | fig|6666666.462.pe g.468;Name=hypothetical protein |
| YP_001467820.1| imidazoleglycerol phosphate | fig|6666666.462.peg.467;Name=Imidazole glycerol phosphate synthasecyclase subunit (EC4.1.3.-);Ontology_term=KEGG_ENZYME:4.1.3.- |
| YP_001467819.1| bacterioferritin comigratory | fig|6666666.462.peg.466;Name=purine nucleoside phosphorylase (punB) |
| YP_001467818.1| radical SAM protein | fig|6666666.462.peg.465;Name=Ribosomal RNA large subunitmethyltransferase N (EC2.1.1.-);Ontology_term=KEGG_ENZYME:2.1.1.- |
| YP_001467817.1| disulfide isomerase | fig|6666666.462.peg.462;Name=Putative ribosomal pseudouridine synthase(EC 4.2.1.70);Ontology_term=KEGG_ENZYME:4.2.1.70 |
| YP_001467816.1| adenylosuccinate lyase | fig|6666666.462.peg.461;Name=Adenylosuccinate lyase (EC4.3.2.2);Ontology_term=KEGG_ENZYME:4.3.2.2 |
| YP_001467815.1| ribonucleotide-diphosphate | fig|6666666.462.peg.459;Name=Ribonucleotide reductase of class Ia(aerobic)2C alpha subunit (EC1.17.4.1);Ontology_term=KEGG_ENZYME:1.17.4.1 |
| YP_001467813.1| anaerobic ribonucleoside | fig|6666666.462.peg.455;Name=Ribonucleotide reductase of class III(anaerobic)2C large subunit (EC1.17.4.2);Ontology_term=KEGG_ENZYME:1.17.4.2 |
| YP_001467812.1| anaerobic | fig|6666666.462.peg.453;Name=Ribonucleotide reductase of class III(anaerobic)2C activating protein (EC1.97.1.4);Ontology_term=KEGG_ENZYME:1.97.1.4 |
| YP_001467811.1| biotin carboxyl carrier | fig|6666666.462.peg.452;Name=NADH-dependent butanol dehydrogenase A (EC1.1.1.-);Ontology_term=KEGG_ENZYME:1.1.1.- |
| YP_001467810.1| oxidoreductase htatip2 | fig|6666666.462.peg.451;Name=Oxidoreductase |
| YP_001467809.1| putative molybdenum ABC | fig|6666666.462.peg.450;Name=Oxidoreductase |
| YP_001467808.1| nicotinate | fig|6666666.462.peg.449;Name=Esterase/lipase |
| YP_001467806.1| antibiotic biosynthesis | fig|6666666.462.peg.448;Name=hypothetical protein |
| YP_001467805.1| flagellar biosynthetic protein | fig|6666666.462.peg.447;Name=Chlorogenate esterase |
| YP_001467804.1| general stress protein 14 | fig|6666666.462.peg.446;Name=NAD(P)H oxidoreductase YRKL (EC 1.6.99.-) @Putative NADPH-quinone reductase (modulator of drugactivity B) @ Flavodoxin2;Ontology_term=KEGG_ENZYME:1.6.99.- |
| YP_001467803.1| hypothetical protein | fig|6666666.462.peg.445;Name=Nitrite transporter from formate/nitritefamily |
| YP_001467802.1| hypothetical protein | fig|6666666.462.peg.444;Name=hypothetical protein |
| YP_001467801.1| enolase (2-phosphoglycerate | fig|6666666.462.peg.443;Name=Rubrerythrin |
| YP_001467798.1| integrase/recombinase | fig|6666666.462.peg.442;Name=Periplasmic thiol:disulfide interchangeprotein2C DsbA-like |
| YP_001467797.1| putative disulfide | fig|6666666.462.peg.441;Name=Inner membrane thiol:disulfideoxidoreductase2C DsbB-like |
| YP_001467796.1| His kinase A [Campylobacter | fig|6666666.462.peg.440;Name=hypothetical protein |
| YP_001467795.1| response regulator receiver | fig|6666666.462.peg.439;Name=Putative two-component response regulator |
| YP_001467790.1| phosphomethylpyrimidine kinase | fig|6666666.462.peg.437;Name=Phosphomethylpyrimidine kinase (EC2.7.4.7);Ontology_term=KEGG_ENZYME:2.7.4.7 |
| YP_001467789.1| dihydroxy-acid dehydratase | fig|6666666.462.peg.436;Name=Dihydroxy-acid dehydratase (EC4.2.1.9);Ontology_term=KEGG_ENZYME:4.2.1.9 |
| YP_001467783.1| hypothetical protein | fig|6666666.462.peg.435;Name=hypothetical protein |
| YP_001467778.1| cytochrome D ubiquinol | fig|6666666.462.peg.434;Name=Cytochrome d ubiquinol oxidase subunit II(EC 1.10.3.-);Ontology_term=KEGG_ENZYME:1.10.3.- |
| YP_001467777.1| ubiquinol oxidase | fig|6666666.462.peg.433;Name=Cytochrome d ubiquinol oxidase subunit I(EC 1.10.3.-);Ontology_term=KEGG_ENZYME:1.10.3.- |
| YP_001467776.1| transglutaminase family | fig|6666666.462.peg.432;Name=hypothetical protein |
| YP_001467775.1| CTP synthetase [Campylobacter | fig|6666666.462.peg.431;Name=CTP synthase (EC6.3.4.2);Ontology_term=KEGG_ENZYME:6.3.4.2 |
| YP_001467774.1| hypothetical protein | fig|6666666.462.peg.429;Name=hypothetical protein |
| YP_001467773.1| single-stranded-DNA-specific | fig|6666666.462.peg.428;Name=Single-stranded-DNA-specific exonucleaseRecJ (EC 3.1.-.-);Ontology_term=KEGG_ENZYME:3.1.-.- |
| YP_001467768.1| hypothetical protein | fig|6666666.462.peg.427;Name=Possible outer membrane protein |
| YP_001467767.1| hypothetical protein | fig|6666666.462.peg.426;Name=hypothetical protein |
| YP_001467766.1| protein-L-isoaspartate | fig|6666666.462.peg.425;Name=Protein-L-isoaspartate O-methyltransferase(EC 2.1.1.77);Ontology_term=KEGG_ENZYME:2.1.1.77 |
| YP_001467765.1| nitrilase/cyanide hydratase | fig|6666666.462.peg.424;Name=Aliphatic amidase amiE (EC3.5.1.4);Ontology_term=KEGG_ENZYME:3.5.1.4 |
| YP_001467763.1| ribonucleotide-diphosphate | fig|6666666.462.peg.422;Name=Ribonucleotide reductase of class Ia(aerobic)2C beta subunit (EC1.17.4.1);Ontology_term=KEGG_ENZYME:1.17.4.1 |
| YP_001467762.1| PepSY-associated TM helix | fig|6666666.462.peg.421;Name=hypothetical protein |
| YP_001467761.1| spore coat polysaccharide | fig|6666666.462.peg.420;Name=hypothetical protein |
| YP_001467758.1| chaperone and heat shock | fig|6666666.462.peg.417;Name=hypothetical protein |
| YP_001467757.1| co-chaperone protein GrpE | fig|6666666.462.peg.416;Name=hypothetical protein |
| YP_001467755.1| hypothetical protein | fig|6666666.462.peg.414;Name=hypothetical protein |
| YP_001467751.1| aspartate carbamoyltransferase | fig|6666666.462.peg.410;Name=hypothetical protein |
| YP_001467747.1| flagellar biosynthesis protein | fig|6666666.462.peg.406;Name=hypothetical protein |
| YP_001467745.1| 30S ribosomal protein S15 | fig|6666666.462.peg.404;Name=Integrase |
| YP_001467744.1| hypothetical protein | fig|6666666.462.peg.1764;Name=hypothetical protein |
| YP_001467742.1| Cys/Met metabolism | fig|6666666.462.peg.861;Name=Cystathionine gamma-lyase (EC4.4.1.1);Ontology_term=KEGG_ENZYME:4.4.1.1 |
| YP_001467740.1| septicolysin [Campylobacter | fig|6666666.462.peg.860;Name=hypothetical protein |
| YP_001467739.1| aminotransferase, class II | fig|6666666.462.peg.859;Name=Putative aminotransferase |
| YP_001467738.1| superoxide dismutase (Fe) | fig|6666666.462.peg.856;Name=Superoxide dismutase [Fe] (EC1.15.1.1);Ontology_term=KEGG_ENZYME:1.15.1.1 |
| YP_001467737.1| C4-dicarboxylate-binding | fig|6666666.462.peg.855;Name=TRAP-type C4-dicarboxylate transportsystem2C periplasmic component |
| YP_001467736.1| TRAP transporter, DctQ-like | fig|6666666.462.peg.854;Name=TRAP-type transport system2C small permeasecomponent2C predicted N-acetylneuraminate transporter |
| YP_001467735.1| trap dicarboxylate | fig|6666666.462.peg.853;Name=TRAP-type C4-dicarboxylate transportsystem2C large permease component |
| YP_001467732.1| aspartokinase (aspartate | fig|6666666.462.peg.850;Name=Ribonuclease E (EC3.1.26.12);Ontology_term=KEGG_ENZYME:3.1.26.12 |
| YP_001467731.1| carbonate dehydratase | fig|6666666.462.peg.848;Name=Carbonic anhydrase (EC4.2.1.1);Ontology_term=KEGG_ENZYME:4.2.1.1 |
| YP_001467730.1| putative ferric receptor CfrA | fig|6666666.462.peg.847;Name=membrane protein |
| YP_001467729.1| thiamine-phosphate | fig|6666666.462.peg.846;Name=Thiamin-phosphate pyrophosphorylase (EC2.5.1.3);Ontology_term=KEGG_ENZYME:2.5.1.3 |
| YP_001467728.1| AraC family transcriptional | fig|6666666.462.peg.845;Name=Preprotein translocase subunit SecG (TC3.A.5.1.1) |
| YP_001467727.1| polysaccharide deacetylase | fig|6666666.462.peg.844;Name=Polysaccharide deacetylase family protein |
| YP_001467726.1| ribosome recycling factor | fig|6666666.462.peg.843;Name=Ribosome recycling factor |
| YP_001467725.1| hypothetical protein | fig|6666666.462.peg.842;Name=hypothetical protein |
| YP_001467724.1| ribonuclease HII | fig|6666666.462.peg.841;Name=Ribonuclease HII (EC3.1.26.4);Ontology_term=KEGG_ENZYME:3.1.26.4 |
| YP_001467723.1| S-adenosylmethionine:tRNA | fig|6666666.462.peg.840;Name=hypothetical protein |
| YP_001467722.1| putative helix-turn-helix | fig|6666666.462.peg.839;Name=Putative helix-turn-helix containsingprotein |
| YP_001467721.1| 30S ribosomal protein S10 | fig|6666666.462.peg.838;Name=SSU ribosomal protein S10p (S20e) |
| YP_001467720.1| 50S ribosomal protein L3 | fig|6666666.462.peg.837;Name=LSU ribosomal protein L3p (L3e) |
| YP_001467719.1| 50S ribosomal protein L4 | fig|6666666.462.peg.836;Name=LSU ribosomal protein L4p (L1e) |
| YP_001467718.1| 50S ribosomal protein L23 | fig|6666666.462.peg.835;Name=LSU ribosomal protein L23p (L23Ae) |
| YP_001467717.1| 50S ribosomal protein L2 | fig|6666666.462.peg.834;Name=LSU ribosomal protein L2p (L8e) |
| YP_001467716.1| 30S ribosomal protein S19 | fig|6666666.462.peg.833;Name=SSU ribosomal protein S19p (S15e) |
| YP_001467715.1| ribosomal protein L22 | fig|6666666.462.peg.832;Name=LSU ribosomal protein L22p (L17e) |
| YP_001467714.1| 30S ribosomal protein S3 | fig|6666666.462.peg.831;Name=SSU ribosomal protein S3p (S3e) |
| YP_001467713.1| 50S ribosomal protein L16 | fig|6666666.462.peg.830;Name=LSU ribosomal protein L16p (L10e) |
| YP_001467712.1| 50S ribosomal protein L29 | fig|6666666.462.peg.829;Name=LSU ribosomal protein L29p (L35e) |
| YP_001467711.1| 30S ribosomal protein S17 | fig|6666666.462.peg.828;Name=SSU ribosomal protein S17p (S11e) |
| YP_001467710.1| 50S ribosomal protein L14 | fig|6666666.462.peg.827;Name=LSU ribosomal protein L14p (L23e) |
| YP_001467709.1| 50S ribosomal protein L24 | fig|6666666.462.peg.826;Name=LSU ribosomal protein L24p (L26e) |
| YP_001467708.1| 50S ribosomal protein L5 | fig|6666666.462.peg.825;Name=LSU ribosomal protein L5p (L11e) |
| YP_001467707.1| 30S ribosomal protein S14 | fig|6666666.462.peg.824;Name=SSU ribosomal protein S14p (S29e) |
| YP_001467706.1| 30S ribosomal protein S8 | fig|6666666.462.peg.823;Name=SSU ribosomal protein S8p (S15Ae) |
| YP_001467705.1| 50S ribosomal protein L6 | fig|6666666.462.peg.822;Name=LSU ribosomal protein L6p (L9e) |
| YP_001467704.1| 50S ribosomal protein L18 | fig|6666666.462.peg.821;Name=LSU ribosomal protein L18p (L5e) |
| YP_001467703.1| 30S ribosomal protein S5 | fig|6666666.462.peg.820;Name=SSU ribosomal protein S5p (S2e) |
| YP_001467702.1| 50S ribosomal protein L15 | fig|6666666.462.peg.819;Name=LSU ribosomal protein L15p (L27Ae) |
| YP_001467701.1| preprotein translocase subunit | fig|6666666.462.peg.818;Name=Preprotein translocase secY subunit (TC3.A.5.1.1) |
| YP_001467700.1| methionine aminopeptidase | fig|6666666.462.peg.817;Name=Methionine aminopeptidase (EC3.4.11.18);Ontology_term=KEGG_ENZYME:3.4.11.18 |
| YP_001467699.1| translation initiation factor | fig|6666666.462.peg.816;Name=Translation initiation factor 1 |
| YP_001467698.1| 30S ribosomal protein S13 | fig|6666666.462.peg.814;Name=SSU ribosomal protein S13p (S18e) |
| YP_001467697.1| 30S ribosomal protein S11 | fig|6666666.462.peg.813;Name=SSU ribosomal protein S11p (S14e) |
| YP_001467696.1| 30S ribosomal protein S4 | fig|6666666.462.peg.812;Name=SSU ribosomal protein S4p (S9e) |
| YP_001467695.1| DNA-directed RNA polymerase | fig|6666666.462.peg.811;Name=DNA-directed RNA polymerase alpha subunit(EC 2.7.7.6);Ontology_term=KEGG_ENZYME:2.7.7.6 |
| YP_001467694.1| ribosomal protein L17 | fig|6666666.462.peg.810;Name=LSU ribosomal protein L17p |
| YP_001467688.1| nitrogenase cofactor | fig|6666666.462.peg.809;Name=Alkanesulfonates-binding protein |
| YP_001467687.1| hypothetical protein | fig|6666666.462.peg.808;Name=NifU protein homolog |
| YP_001467686.1| two-component sensor kinase | fig|6666666.462.peg.807;Name=hypothetical protein |
| YP_001467685.1| | fig|6666666.462.peg.806;Name=UDP-N-acetylmuramoylalanyl-D-glutamate--22C6-diaminopimelate ligase (EC6.3.2.13);Ontology_term=KEGG_ENZYME:6.3.2.13 |
| YP_001467684.1| aspartate alpha-decarboxylase | fig|6666666.462.peg.805;Name=Aspartate 1-decarboxylase (EC4.1.1.11);Ontology_term=KEGG_ENZYME:4.1.1.11 |
| YP_001467683.1| hypothetical protein | fig|6666666.462.peg.804;Name=Transcriptional regulatory protein |
| YP_001467682.1| sigma 54 modulation | fig|6666666.462.peg.803;Name=Putative periplasmic protein |
| YP_001467681.1| geranyltranstransferase | fig|6666666.462.peg.802;Name=Octaprenyl-diphosphate synthase (EC2.5.1.-) / Dimethylallyltransferase (EC 2.5.1.1) /Geranyltranstransferase (farnesyldiphosphate synthase)(EC 2.5.1.10) / Geranylgeranyl pyrophosphate synthetase(EC2.5.1.29);Ontology_term=KEGG_ENZYME:2.5.1.-,KEGG_ENZYME:2.5.1.1,KEGG_ENZYME:2.5.1.10,KEGG_ENZYME:2.5.1.29 |
| YP_001467680.1| transketolase [Campylobacter | fig|6666666.462.peg.801;Name=Transketolase (EC2.2.1.1);Ontology_term=KEGG_ENZYME:2.2.1.1 |
| YP_001467679.1| putative undecaprenol kinase | fig|6666666.462.peg.800;Name=Undecaprenyl-diphosphatase (EC3.6.1.27);Ontology_term=KEGG_ENZYME:3.6.1.27 |
| YP_001467678.1| hypothetical protein | fig|6666666.462.peg.798;Name=hypothetical protein |
| YP_001467677.1| hypothetical protein | fig|6666666.462.peg.797;Name=hypothetical protein |
| YP_001467676.1| molybdenum cofactor | fig|6666666.462.peg.796;Name=Molybdenum cofactor biosynthesis proteinMoaC |
| YP_001467675.1| ATP-dependent protease La 1 | fig|6666666.462.peg.795;Name=hypothetical protein |
| YP_001467674.1| methyl-accepting chemotaxis | fig|6666666.462.peg.794;Name=Nitrous-oxide reductase (EC1.7.99.6);Ontology_term=KEGG_ENZYME:1.7.99.6 |
| YP_001467673.1| hypothetical protein | fig|6666666.462.peg.793;Name=hypothetical protein |
| YP_001467672.1| nitrous oxidase accessory | fig|6666666.462.peg.792;Name=Nitrous oxide reductase maturation proteinNosD |
| YP_001467671.1| hypothetical protein | fig|6666666.462.peg.790;Name=Cytochrome c family protein |
| YP_001467670.1| hypothetical protein | fig|6666666.462.peg.789;Name=Cytochrome c family protein |
| YP_001467669.1| NapH/MauN family | fig|6666666.462.peg.788;Name=Polyferredoxin NapH (periplasmic nitratereductase) |
| YP_001467668.1| methionine import ATP-binding | fig|6666666.462.peg.787;Name=Nitrous oxide reductase maturation proteinNosF (ATPase) |
| YP_001467667.1| ABC transporter [Campylobacter | fig|6666666.462.peg.786;Name=Nitrous oxide reductase maturationtransmembrane protein NosY |
| YP_001467666.1| Sel1 repeat-containing protein | fig|6666666.462.peg.785;Name=Putative periplasmic protein |
| YP_001467665.1| hypothetical protein | fig|6666666.462.peg.784;Name=hypothetical protein |
| YP_001467664.1| PepSY-associated TM helix | fig|6666666.462.peg.783;Name=hypothetical protein |
| YP_001467663.1| hypothetical protein | fig|6666666.462.peg.780;Name=hypothetical protein |
| YP_001467662.1| Na+/H+ antiporter | fig|6666666.462.peg.779;Name=Na+/H+ antiporter |
| YP_001467661.1| acetyltransferase | fig|6666666.462.peg.778;Name=Ribosomal-protein-S18p-alanineacetyltransferase (EC2.3.1.-);Ontology_term=KEGG_ENZYME:2.3.1.- |
| YP_001467660.1| M48 family peptidase | fig|6666666.462.peg.777;Name=Zn-dependent protease with chaperone function |
| YP_001467658.1| putative transporter | fig|6666666.462.peg.1627;Name=Transmembrane transport protein |
| YP_001467657.1| hypothetical protein | fig|6666666.462.peg.1628;Name=CopG protein |
| YP_001467656.1| sodium/neurotransmitter | fig|6666666.462.peg.1629;Name=Sodium-dependent tyrosine transporter |
| YP_001467655.1| pyruvate-ferredoxin | fig|6666666.462.peg.1630;Name=Hemolysins and related proteins containingCBS domains |
| YP_001467654.1| sugar efflux transporter B | fig|6666666.462.peg.1631;Name=hypothetical protein |
| YP_001467653.1| hypothetical protein | fig|6666666.462.peg.1632;Name=hypothetical protein |
| YP_001467652.1| LemA protein [Campylobacter | fig|6666666.462.peg.1633;Name=LemA protein |
| YP_001467651.1| fumarate hydratase | fig|6666666.462.peg.1634;Name=Fumarate hydratase class I2C aerobic (EC4.2.1.2)%3B L(+)-tartrate dehydratase alpha subunit (EC4.2.1.32);Ontology_term=KEGG_ENZYME:4.2.1.2,KEGG_ENZYME:4.2.1.32 |
| YP_001467650.1| L-cystine import ATP-binding | fig|6666666.462.peg.1635;Name=Fumarate hydratase class I2C aerobic (EC4.2.1.2)%3B L(+)-tartrate dehydratase beta subunit (EC4.2.1.32);Ontology_term=KEGG_ENZYME:4.2.1.2,KEGG_ENZYME:4.2.1.32 |
| YP_001467649.1| L-cystine-binding protein TcyA | fig|6666666.462.peg.1636;Name=hypothetical protein |
| YP_001467648.1| L-cystine permease protein | fig|6666666.462.peg.1637;Name=hypothetical protein |
| YP_001467647.1| putative endoribonuclease | fig|6666666.462.peg.1638;Name=Endoribonuclease L-PSP |
| YP_001467637.1| hydrogenase-4 component B | fig|6666666.462.peg.159;Name=hypothetical protein |
| YP_001467636.1| hydrogenase-4 component C | fig|6666666.462.peg.161;Name=hypothetical protein |
| YP_001467635.1| deoxycytidine triphosphate | fig|6666666.462.peg.162;Name=Deoxycytidine triphosphate deaminase (EC3.5.4.13);Ontology_term=KEGG_ENZYME:3.5.4.13 |
| YP_001467625.1| DNA topoisomerase I | fig|6666666.462.peg.1833;Name=DNA topoisomerase I (EC5.99.1.2);Ontology_term=KEGG_ENZYME:5.99.1.2 |
| YP_001467624.1| hypothetical protein | fig|6666666.462.peg.1834;Name=internalin2C putative (LPXTG motif) |
| YP_001467623.1| oxygen-sensitive | fig|6666666.462.peg.263;Name=hypothetical protein |
| YP_001467622.1| biotin synthase [Campylobacter | fig|6666666.462.peg.1835;Name=Biotin synthase (EC2.8.1.6);Ontology_term=KEGG_ENZYME:2.8.1.6 |
| YP_001467621.1| CrcB protein [Campylobacter | fig|6666666.462.peg.1836;Name=Protein crcB homolog |
| YP_001467619.1| hypothetical protein | fig|6666666.462.peg.502;Name=hypothetical protein |
| YP_001467617.1| radical SAM protein | fig|6666666.462.peg.503;Name=NA+/H+ antiporter (napA)2C putative |
| YP_001467616.1| citrate (Si)-synthase | fig|6666666.462.peg.504;Name=Citrate synthase (si) (EC2.3.3.1);Ontology_term=KEGG_ENZYME:2.3.3.1 |
| YP_001467615.1| protein CysQ [Campylobacter | fig|6666666.462.peg.505;Name=3'(2')2C5'-bisphosphate nucleotidase (EC3.1.3.7);Ontology_term=KEGG_ENZYME:3.1.3.7 |
| YP_001467614.1| DNA helicase related protein | fig|6666666.462.peg.506;Name=hypothetical protein |
| YP_001467613.1| dimethyladenosine transferase | fig|6666666.462.peg.507;Name=hypothetical protein |
| YP_001467612.1| putative BcpB [Campylobacter | fig|6666666.462.peg.508;Name=Alkyl hydroperoxide reductase subunit C-likeprotein |
| YP_001467611.1| molybdate ABC transporter, | fig|6666666.462.peg.509;Name=Molybdenum transport system permease proteinModB (TC 3.A.1.8.1) |
| YP_001467610.1| RNA pseudouridine synthase | fig|6666666.462.peg.510;Name=Molybdenum transport ATP-binding protein ModC(TC 3.A.1.8.1) |
| YP_001467609.1| beta-lactamase HcpA | fig|6666666.462.peg.511;Name=hypothetical protein |
| YP_001467608.1| molybdate ABC transporter, | fig|6666666.462.peg.512;Name=Molybdenum ABC transporter2C periplasmicmolybdenum-binding protein ModA (TC 3.A.1.8.1) |
| YP_001467607.1| molybdenum-pterin binding | fig|6666666.462.peg.513;Name=DNA-binding domain of ModE /Molybdate-binding domain of ModE |
| YP_001467606.1| ABC transporter ATPase | fig|6666666.462.peg.514;Name=putative periplasmic protein (vacJ homolog) |
| YP_001467605.1| toluene tolerance protein | fig|6666666.462.peg.515;Name=Putative periplasmic protein |
| YP_001467604.1| hypothetical protein | fig|6666666.462.peg.516;Name=membrane protein |
| YP_001467603.1| putative transport-associated | fig|6666666.462.peg.517;Name=Osmotically inducible protein Y precursor |
| YP_001467602.1| hypothetical protein | fig|6666666.462.peg.180;Name=hypothetical protein |
| YP_001467601.1| Hsp12 variant C [Campylobacter | fig|6666666.462.peg.179;Name=Fibronectin/fibrinogen-binding protein |
| YP_001467598.1| 3-isopropylmalate dehydratase | fig|6666666.462.peg.176;Name=3-isopropylmalate dehydratase large subunit(EC 4.2.1.33);Ontology_term=KEGG_ENZYME:4.2.1.33 |
| YP_001467597.1| hypothetical protein | fig|6666666.462.peg.175;Name=hypothetical protein |
| YP_001467596.1| hypothetical protein | fig|6666666.462.peg.174;Name=Molybdopterin-guanine dinucleotidebiosynthesis protein MobA |
| YP_001467595.1| putative two-component sensor | fig|6666666.462.peg.173;Name=Phospholipase A1 precursor (EC 3.1.1.322CEC 3.1.1.4)%3B Outer membrane phospholipaseA;Ontology_term=KEGG_ENZYME:3.1.1.32,KEGG_ENZYME:3.1.1.4 |
| YP_001467592.1| transport protein | fig|6666666.462.peg.598;Name=Integrase-recombinase protein XERCD family |
| YP_001467591.1| putative periplasmic protein | fig|6666666.462.peg.529;Name=Putative periplasmic protein |
| YP_001467590.1| hypothetical protein | fig|6666666.462.peg.528;Name=hypothetical protein |
| YP_001467589.1| phosphopyruvate hydratase | fig|6666666.462.peg.527;Name=Enolase (EC4.2.1.11);Ontology_term=KEGG_ENZYME:4.2.1.11 |
| YP_001467588.1| recombinase A [Campylobacter | fig|6666666.462.peg.526;Name=RecA protein |
| YP_001467587.1| addiction module antitoxin | fig|6666666.462.peg.525;Name=hypothetical protein |
| YP_001467586.1| | fig|6666666.462.peg.524;Name=UDP-N-acetylenolpyruvoylglucosaminereductase (EC1.1.1.158);Ontology_term=KEGG_ENZYME:1.1.1.158 |
| YP_001467585.1| flagellar biosynthesis protein | fig|6666666.462.peg.523;Name=Flagellar biosynthesis protein FliQ |
| YP_001467584.1| S-ribosylhomocysteine lyase | fig|6666666.462.peg.522;Name=Menaquinone via futalosine step 4 |
| YP_001467583.1| methyl-accepting chemotaxis | fig|6666666.462.peg.521;Name=hypothetical protein |
| YP_001467582.1| nicotinate | fig|6666666.462.peg.520;Name=Nicotinate phosphoribosyltransferase (EC2.4.2.11);Ontology_term=KEGG_ENZYME:2.4.2.11 |
| YP_001467581.1| molybdate ABC transporter, | fig|6666666.462.peg.519;Name=Molybdenum ABC transporter2C periplasmicmolybdenum-binding protein ModA (TC 3.A.1.8.1) |
| YP_001467579.1| acetyl-CoA carboxylase, biotin | fig|6666666.462.peg.182;Name=Biotin carboxyl carrier protein ofacetyl-CoA carboxylase |
| YP_001467578.1| biotin carboxylase | fig|6666666.462.peg.183;Name=Biotin carboxylase of acetyl-CoAcarboxylase (EC6.3.4.14);Ontology_term=KEGG_ENZYME:6.3.4.14 |
| YP_001467577.1| DNA gyrase subunit B | fig|6666666.462.peg.184;Name=membrane protein |
| YP_001467576.1| putative integral membrane | fig|6666666.462.peg.185;Name=membrane protein |
| YP_001467575.1| hydrolase [Campylobacter | fig|6666666.462.peg.186;Name=Phosphoribosyl-AMP cyclohydrolase (EC3.5.4.19) / Phosphoribosyl-ATP pyrophosphatase (EC3.6.1.31);Ontology_term=KEGG_ENZYME:3.5.4.19,KEGG_ENZYME:3.6.1.31 |
| YP_001467574.1| SPFH domain-containing protein | fig|6666666.462.peg.187;Name=Membrane protease subunits2Cstomatin/prohibitin homologs |
| YP_001467573.1| branched-chain amino acid | fig|6666666.462.peg.188;Name=Branched-chain amino acid aminotransferase(EC 2.6.1.42);Ontology_term=KEGG_ENZYME:2.6.1.42 |
| YP_001467572.1| hypothetical protein | fig|6666666.462.peg.189;Name=Periplasmic thiol:disulfide interchangeprotein2C DsbA-like |
| YP_001467570.1| anti-oxidant AhpCTSA family | fig|6666666.462.peg.190;Name=Thiol peroxidase2C Bcp-type (EC1.11.1.15);Ontology_term=KEGG_ENZYME:1.11.1.15 |
| YP_001467569.1| hypothetical protein | fig|6666666.462.peg.191;Name=4-oxalocrotonate tautomerase (EC5.3.2.-);Ontology_term=KEGG_ENZYME:5.3.2.- |
| YP_001467568.1| 50S ribosomal protein L20 | fig|6666666.462.peg.192;Name=hypothetical protein |
| YP_001467567.1| diaminopimelate epimerase (DAP | fig|6666666.462.pe g.193;Name=putative exported protein |
| YP_001467566.1| hypothetical protein | fig|6666666.462.peg.194;Name=Putative peptide ABC-transport systemperiplasmic peptide-binding protein |
| YP_001467565.1| dipeptidase [Campylobacter | fig|6666666.462.peg.195;Name=Putative peptide ABC-transport systempermease protein |
| YP_001467564.1| peptide ABC transporter, | fig|6666666.462.peg.196;Name=Putative peptide ABC-transport systempermease protein |
| YP_001467563.1| | fig|6666666.462.peg.197;Name=Putative peptide ABC-transport systemATP-binding protein |
| YP_001467562.1| putative ABC transporter, | fig|6666666.462.peg.198;Name=Putative peptide ABC-transport systemATP-binding protein |
| YP_001467561.1| putative orphan protein | fig|6666666.462.peg.199;Name=Protein of unknown function DUF208 |
| YP_001467560.1| (3R)-hydroxymyristoyl-ACP | fig|6666666.462.peg.200;Name=(3R)-hydroxymyristoyl-[acyl carrierprotein] dehydratase (EC4.2.1.-);Ontology_term=KEGG_ENZYME:4.2.1.- |
| YP_001467559.1| ATP-dependent protease | fig|6666666.462.peg.202;Name=ATP-dependent Clp protease ATP-bindingsubunit ClpX |
| YP_001467558.1| cell shape determining protein | fig|6666666.462.peg.203;Name=Rod shape-determining protein MreB |
| YP_001467557.1| putative | fig|6666666.462.peg.204;Name=Rod shape-determining protein MreC |
| YP_001467556.1| carbamoyl phosphate synthase | fig|6666666.462.pe g.205;Name=Carbamoyl-phosphate synthase large chain (EC 6.3.5.5);Ontology_term=KEGG_ENZYME:6.3.5.5 |
| YP_001467555.1| hypothetical protein | fig|6666666.462.peg.654;Name=NAD(FAD)-utilizing dehydrogenases |
| YP_001467554.1| ATPase, AAA family protein | fig|6666666.462.peg.775;Name=Flagellar protein FlbB |
| YP_001467553.1| hypothetical protein | fig|6666666.462.peg.774;Name=hypothetical protein |
| YP_001467552.1| adenylosuccinate synthetase | fig|6666666.462.peg.773;Name=Adenylosuccinate synthetase (EC6.3.4.4);Ontology_term=KEGG_ENZYME:6.3.4.4 |
| YP_001467551.1| ATP phosphoribosyltransferase | fig|6666666.462.peg.772;Name=ATP phosphoribosyltransferase regulatorysubunit2C divergent variant (EC2.4.2.17);Ontology_term=KEGG_ENZYME:2.4.2.17 |
| YP_001467550.1| YbaK [Campylobacter concisus | fig|6666666.462.pe g.771;Name=diguanylate cyclase/phosphodiesterase (GGDEF & EAL domains) with PAS/PAC sensor(s) |
| YP_001467549.1| nitroreductase family protein | fig|6666666.462.peg.770;Name=Oxygen-insensitive NAD(P)H nitroreductase(EC 1.-.-.-) / Dihydropteridine reductase (EC1.5.1.34);Ontology_term=KEGG_ENZYME:1.-.-.-,KEGG_ENZYME:1.5.1.34 |
| YP_001467548.1| selenide, water dikinase | fig|6666666.462.peg.769;Name=Selenide2Cwater dikinase (EC 2.7.9.3) @selenocysteine-containing;Ontology_term=KEGG_ENZYME:2.7.9.3 |
| YP_001467547.1| translation initiation factor | fig|6666666.462.peg.768;Name=Hypothetical protein Cj1505c |
| YP_001467546.1| formate dehydrogenase, | fig|6666666.462.peg.767;Name=Putative formate dehydrogenase2C cytochromeB subunit (EC 1.2.1.2);Ontology_term=KEGG_ENZYME:1.2.1.2 |
| YP_001467545.1| DJ-1/PfpI family protein | fig|6666666.462.peg.766;Name=ThiJ/PfpI family protein |
| YP_001467543.1| carboxynorspermidine | fig|6666666.462.peg.763;Name=Carboxynorspermidine decarboxylase2Cputative (EC 4.1.1.-);Ontology_term=KEGG_ENZYME:4.1.1.- |
| YP_001467542.1| molybdopterin converting | fig|6666666.462.peg.761;Name=Possible molybdopterin converting factor2Csubunit 2 |
| YP_001467541.1| hypothetical protein | fig|6666666.462.peg.760;Name=hypothetical protein |
| YP_001467540.1| hypothetical protein | fig|6666666.462.peg.753;Name=hypothetical protein |
| YP_001467539.1| thioesterase family protein | fig|6666666.462.peg.752;Name=Putative acyl-CoA thioester hydrolase (EC3.1.2.-);Ontology_term=KEGG_ENZYME:3.1.2.- |
| YP_001467538.1| lipoprotein signal peptidase | fig|6666666.462.peg.751;Name=Campylobacter invasion antigen B (CiaB) |
| YP_001467537.1| phosphoglucosamine mutase | fig|6666666.462.peg.749;Name=diguanylate cyclase/phosphodiesterase (GGDEF& EAL domains) with PAS/PAC sensor(s) |
| YP_001467536.1| 30S ribosomal protein S20 | fig|6666666.462.peg.540;Name=hypothetical protein |
| YP_001467534.1| hypothetical protein | fig|6666666.462.peg.538;Name=hypothetical protein |
| YP_001467533.1| dUTPase [Campylobacter | fig|6666666.462.peg.537;Name=Dimeric dUTPase (EC3.6.1.23);Ontology_term=KEGG_ENZYME:3.6.1.23 |
| YP_001467532.1| putative integral membrane | fig|6666666.462.peg.536;Name=Probable integral membrane protein Cj1452 |
| YP_001467531.1| diacylglycerol kinase | fig|6666666.462.peg.535;Name=Putative ATP/GTP-binding protein |
| YP_001467530.1| putative L-arabinose ABC | fig|6666666.462.peg.534;Name=hypothetical protein |
| YP_001467529.1| protein YhbP [Campylobacter | fig|6666666.462.peg.533;Name=hypothetical protein |
| YP_001467523.1| periplasmic solute binding | fig|6666666.462.peg.1832;Name=hypothetical protein |
| YP_001467506.1| hypothetical protein | fig|6666666.462.peg.1831;Name=hypothetical protein |
| YP_001467505.1| AAA ATPase family protein | fig|6666666.462.peg.1831;Name=hypothetical protein |
| YP_001467504.1| long-chain-fatty-acid--CoA | fig|6666666.462.peg.1830;Name=Competence/damage-inducible protein CinAfamily |
| YP_001467503.1| inositol-5-monophosphate | fig|6666666.462.peg.1827;Name=Inosine-5'-monophosphate dehydrogenase (EC1.1.1.205);Ontology_term=KEGG_ENZYME:1.1.1.205 |
| YP_001467502.1| homoserine O-acetyltransferase | fig|6666666.462.peg.1826;Name=Homoserine O-acetyltransferase (EC2.3.1.31);Ontology_term=KEGG_ENZYME:2.3.1.31 |
| YP_001467501.1| 30S ribosomal protein S17 | fig|6666666.462.peg.1825;Name=Putative coiled-coil protein |
| YP_001467500.1| 50S ribosomal protein L24 | fig|6666666.462.peg.1823;Name=DNA-3-methyladenine glycosylase (EC3.2.2.20);Ontology_term=KEGG_ENZYME:3.2.2.20 |
| YP_001467497.1| 30S ribosomal protein S8 | fig|6666666.462.peg.1822;Name=Protein of unknown function DUF419 |
| YP_001467482.1| hypothetical protein | fig|6666666.462.peg.1819;Name=membrane protein |
| YP_001467481.1| recombination and DNA strand | fig|6666666.462.peg.1818;Name=Recombination inhibitory protein MutS2 |
| YP_001467479.1| succinyl-diaminopimelate | fig|6666666.462.peg.1816;Name=N-succinyl-L2CL-diaminopimelatedesuccinylase (EC3.5.1.18);Ontology_term=KEGG_ENZYME:3.5.1.18 |
| YP_001467478.1| putative periplasmic protein | fig|6666666.462.peg.1813;Name=Possible periplasmic protein |
| YP_001467477.1| glutamyl-tRNA synthetase | fig|6666666.462.peg.1812;Name=Glutamyl-tRNA synthetase (EC6.1.1.17);Ontology_term=KEGG_ENZYME:6.1.1.17 |
| YP_001467476.1| transketolase A [Campylobacter | fig|6666666.462.peg.1811;Name=NADP-dependent malic enzyme (EC1.1.1.40);Ontology_term=KEGG_ENZYME:1.1.1.40 |
| YP_001467475.1| uracil | fig|6666666.462.peg.1810;Name=Uracil phosphoribosyltransferase (EC2.4.2.9);Ontology_term=KEGG_ENZYME:2.4.2.9 |
| YP_001467474.1| S-layer-like domain-containing | fig|6666666.462.peg.1809;Name=Putative ribonuclease |
| YP_001467473.1| hypothetical protein | fig|6666666.462.peg.1808;Name=hypothetical protein |
| YP_001467472.1| hypothetical protein | fig|6666666.462.peg.1807;Name=Menaquinone via futalosine step 1 |
| YP_001467467.1| arylsulfotransferase | fig|6666666.462.peg.663;Name=Putative arylsulfate sulfotransferase (EC2.8.2.22);Ontology_term=KEGG_ENZYME:2.8.2.22 |
| YP_001467465.1| shikimate 5-dehydrogenase | fig|6666666.462.peg.665;Name=Shikimate 5-dehydrogenase I alpha (EC1.1.1.25);Ontology_term=KEGG_ENZYME:1.1.1.25 |
| YP_001467464.1| ABC transporter ATP-binding | fig|6666666.462.peg.666;Name=membrane protein |
| YP_001467463.1| ABC-type transport system | fig|6666666.462.peg.667;Name=hypothetical protein |
| YP_001467462.1| serine | fig|6666666.462.peg.668;Name=Serine hydroxymethyltransferase (EC2.1.2.1);Ontology_term=KEGG_ENZYME:2.1.2.1 |
| YP_001467461.1| lysyl-tRNA synthetase | fig|6666666.462.peg.669;Name=Lysyl-tRNA synthetase (class II) (EC6.1.1.6);Ontology_term=KEGG_ENZYME:6.1.1.6 |
| YP_001467460.1| putative iron-regulated | fig|6666666.462.peg.670;Name=Ferric uptake regulation protein FUR |
| YP_001467459.1| CvpA family protein | fig|6666666.462.peg.671;Name=Putative integral membrane protein |
| YP_001467458.1| aspartyl/glutamyl-tRNA | fig|6666666.462.peg.673;Name=Aspartyl-tRNA(Asn) amidotransferasesubunit C (EC 6.3.5.6) @ Glutamyl-tRNA(Gln)amidotransferase subunit C (EC6.3.5.7);Ontology_term=KEGG_ENZYME:6.3.5.6,KEGG_ENZYME:6.3.5.7 |
| YP_001467457.1| hypothetical protein | fig|6666666.462.peg.674;Name=hypothetical protein |
| YP_001467456.1| plasminogen-binding protein | fig|6666666.462.peg.675;Name=Putative lipoprotein |
| YP_001467455.1| hypothetical protein | fig|6666666.462.peg.676;Name=hypothetical protein |
| YP_001467454.1| pantothenate kinase | fig|6666666.462.peg.677;Name=Pantothenate kinase type III2C CoaX-like(EC 2.7.1.33);Ontology_term=KEGG_ENZYME:2.7.1.33 |
| YP_001467453.1| ATP phosphoribosyltransferase | fig|6666666.462.peg.678;Name=ATP phosphoribosyltransferase (EC2.4.2.17);Ontology_term=KEGG_ENZYME:2.4.2.17 |
| YP_001467451.1| putative aminotransferase | fig|6666666.462.peg.679;Name=Putative aminotransferase |
| YP_001467450.1| sodium-dependent tyrosine | fig|6666666.462.peg.680;Name=hypothetical protein |
| YP_001467449.1| CBS domain-containing protein | fig|6666666.462.peg.681;Name=N-terminal HTH domain of molybdenum-bindingprotein family |
| YP_001467448.1| formate dehydrogenase | fig|6666666.462.peg.682;Name=Formate dehydrogenase chain D (EC1.2.1.2);Ontology_term=KEGG_ENZYME:1.2.1.2 |
| YP_001467447.1| hypothetical protein | fig|6666666.462.peg.683;Name=hypothetical protein |
| YP_001467446.1| cytoplasmic membrane protein | fig|6666666.462.peg.684;Name=Formate dehydrogenase-O2C iron-sulfursubunit (EC 1.2.1.2)%3B Putative formate dehydrogenaseiron-sulfur subunit (EC1.2.1.2);Ontology_term=KEGG_ENZYME:1.2.1.2,KEGG_ENZYME:1.2.1.2 |
| YP_001467445.1| fumarate hydratase | fig|6666666.462.peg.685;Name=Formate dehydrogenase-O2C major subunit (EC1.2.1.2) @selenocysteine-containing;Ontology_term=KEGG_ENZYME:1.2.1.2 |
| YP_001467444.1| Tat pathway signal sequence | fig|6666666.462.peg.686;Name=Formate dehydrogenase subunit oraccessory protein |
| YP_001467443.1| hypothetical protein | fig|6666666.462.peg.687;Name=Putative formate dehydrogenase-specificchaperone |
| YP_001467442.1| protein YabJ [Campylobacter | fig|6666666.462.peg.688;Name=ABC-type tungstate transport system2CATP-binding protein |
| YP_001467441.1| ABC transporter, permease | fig|6666666.462.peg.689;Name=ABC-type tungstate transport system2Cpermease protein |
| YP_001467439.1| molybdopterin oxidoreductase | fig|6666666.462.peg.1771;Name=NAD-dependent formate dehydrogenase alphasubunit @ selenocysteine-containing |
| YP_001467399.1| | fig|6666666.462.peg.713;Name=UDP-N-acetylmuramoylalanine--D-glutamateligase (EC 6.3.2.9);Ontology_term=KEGG_ENZYME:6.3.2.9 |
| YP_001467398.1| hypothetical protein | fig|6666666.462.peg.714;Name=hypothetical protein |
| YP_001467397.1| phosphoglyceromutase | fig|6666666.462.peg.716;Name=22C3-bisphosphoglycerate-independentphosphoglycerate mutase (EC5.4.2.1);Ontology_term=KEGG_ENZYME:5.4.2.1 |
| YP_001467396.1| | fig|6666666.462.peg.717;Name=3-oxoacyl-[acyl-carrier protein] reductase(EC 1.1.1.100);Ontology_term=KEGG_ENZYME:1.1.1.100 |
| YP_001467395.1| acyl carrier protein | fig|6666666.462.peg.718;Name=Acyl carrier protein |
| YP_001467394.1| 3-oxoacyl-(acyl carrier | fig|6666666.462.peg.719;Name=3-oxoacyl-[acyl-carrier-protein] synthase2CKASII (EC 2.3.1.41);Ontology_term=KEGG_ENZYME:2.3.1.41 |
| YP_001467393.1| acetyl-CoA carboxylase | fig|6666666.462.peg.720;Name=Acetyl-coenzyme A carboxyl transferasealpha chain (EC6.4.1.2);Ontology_term=KEGG_ENZYME:6.4.1.2 |
| YP_001467392.1| peptide methionine sulfoxide | fig|6666666.462.peg.722;Name=hypothetical protein |
| YP_001467390.1| riboflavin synthase subunit | fig|6666666.462.peg.723;Name=Riboflavin synthase alpha chain (EC2.5.1.9);Ontology_term=KEGG_ENZYME:2.5.1.9 |
| YP_001467389.1| hypothetical protein | fig|6666666.462.peg.724;Name=COG1496: Uncharacterized conserved protein |
| YP_001467388.1| hypothetical protein | fig|6666666.462.peg.725;Name=hypothetical protein |
| YP_001467387.1| isoaspartyl dipeptidase | fig|6666666.462.peg.726;Name=Isoaspartyl dipeptidase (EC 3.4.19.5) @Asp-X dipeptidase;Ontology_term=KEGG_ENZYME:3.4.19.5 |
| YP_001467386.1| 30S ribosomal protein S2 | fig|6666666.462.peg.727;Name=SSU ribosomal protein S2p (SAe) |
| YP_001467385.1| elongation factor Ts | fig|6666666.462.peg.728;Name=Translation elongation factor Ts |
| YP_001467384.1| modulator of drug activity | fig|6666666.462.peg.729;Name=Putative ABC transporter ATP bindingprotein |
| YP_001467381.1| guanylate kinase | fig|6666666.462.peg.732;Name=Guanylate kinase (EC2.7.4.8);Ontology_term=KEGG_ENZYME:2.7.4.8 |
| YP_001467380.1| twin arginine-targeting | fig|6666666.462.peg.733;Name=Twin-arginine translocation protein TatA |
| YP_001467379.1| arginyl-tRNA synthetase | fig|6666666.462.peg.734;Name=Arginyl-tRNA synthetase (EC6.1.1.19);Ontology_term=KEGG_ENZYME:6.1.1.19 |
| YP_001467356.1| hypothetical protein | fig|6666666.462.peg.736;Name=Putative membrane protein YeiH |
| YP_001467355.1| gerC2 protein [Campylobacter | fig|6666666.462.peg.737;Name=Nicotinate-nucleotide adenylyltransferase(EC 2.7.7.18) / FIGfam135315: Iojap-relatedprotein;Ontology_term=KEGG_ENZYME:2.7.7.18 |
| YP_001467354.1| glyceraldehyde-3-phosphate | fig|6666666.462.peg.738;Name=NAD-dependent glyceraldehyde-3-phosphatedehydrogenase (EC1.2.1.12);Ontology_term=KEGG_ENZYME:1.2.1.12 |
| YP_001467353.1| phosphoglycerate kinase | fig|6666666.462.peg.739;Name=Phosphoglycerate kinase (EC2.7.2.3);Ontology_term=KEGG_ENZYME:2.7.2.3 |
| YP_001467352.1| triosephosphate isomerase | fig|6666666.462.peg.740;Name=Triosephosphate isomerase (EC5.3.1.1);Ontology_term=KEGG_ENZYME:5.3.1.1 |
| YP_001467351.1| enoyl-(acyl carrier protein) | fig|6666666.462.peg.742;Name=Enoyl-[acyl-carrier-protein] reductase[NADH] (EC 1.3.1.9);Ontology_term=KEGG_ENZYME:1.3.1.9 |
| YP_001467350.1| hypothetical protein | fig|6666666.462.peg.743;Name=ABC-type sugar transport system2Cperiplasmic component |
| YP_001467349.1| histidinol-phosphate | fig|6666666.462.peg.1439;Name=Rod shape-determining protein RodA |
| YP_001467348.1| P-protein [Campylobacter | fig|6666666.462.peg.1438;Name=Ribosomal large subunit pseudouridinesynthase D (EC4.2.1.70);Ontology_term=KEGG_ENZYME:4.2.1.70 |
| YP_001467347.1| diaminopimelate decarboxylase | fig|6666666.462.peg.1437;Name=Putative fibronectin domain-containinglipoprotein |
| YP_001467346.1| tRNA | fig|6666666.462.peg.1435;Name=tRNA (guanine46-N7-)-methyltransferase (EC2.1.1.33);Ontology_term=KEGG_ENZYME:2.1.1.33 |
| YP_001467345.1| 50S ribosomal protein L25 | fig|6666666.462.peg.1434;Name=Cell division transporter2C ATP-bindingprotein FtsE (TC 3.A.5.1.1) |
| YP_001467344.1| cell division protein FtsX | fig|6666666.462.peg.1433;Name=Cell division protein FtsX |
| YP_001467343.1| transaldolase [Campylobacter | fig|6666666.462.peg.1432;Name=Putative periplasmic protein |
| YP_001467342.1| uridylate kinase | fig|6666666.462.peg.1431;Name=Uridylate kinase |
| YP_001467341.1| DNA-directed RNA polymerase | fig|6666666.462.peg.1430;Name=DNA-directed RNA polymerase omega subunit(EC 2.7.7.6);Ontology_term=KEGG_ENZYME:2.7.7.6 |
| YP_001467340.1| GTP pyrophosphokinase | fig|6666666.462.peg.1429;Name=GTP pyrophosphokinase (EC 2.7.6.5)2C (p)ppGppsynthetase II / Guanosine-3'2C5'-bis(diphosphate)3'-pyrophosphohydrolase (EC3.1.7.2);Ontology_term=KEGG_ENZYME:2.7.6.5,KEGG_ENZYME:3.1.7.2 |
| YP_001467339.1| tyrosyl-tRNA synthetase | fig|6666666.462.peg.1428;Name=Tyrosyl-tRNA synthetase (EC6.1.1.1);Ontology_term=KEGG_ENZYME:6.1.1.1 |
| YP_001467338.1| 2-nitropropane dioxygenase | fig|6666666.462.peg.1427;Name=Putative dioxygenase related to 2-nitropropanedioxygenase |
| YP_001467337.1| transcription elongation | fig|6666666.462.peg.932;Name=N-acetylmuramoyl-L-alanine amidase (EC3.5.1.28);Ontology_term=KEGG_ENZYME:3.5.1.28 |
| YP_001467336.1| | fig|6666666.462.peg.862;Name=Predicted glycine/D-amino acid oxidase(deaminating) |
| YP_001467335.1| 5'-nucleotidase SurE | fig|6666666.462.peg.863;Name=hypothetical protein |
| YP_001467334.1| molybdopterin biosynthesis | fig|6666666.462.peg.864;Name=Multidrug resistance transporter2C Bcr/CflAfamily |
| YP_001467333.1| uroporphyrinogen-III synthase | fig|6666666.462.peg.865;Name=Uroporphyrinogen-III synthase (EC4.2.1.75);Ontology_term=KEGG_ENZYME:4.2.1.75 |
| YP_001467332.1| phosphoribosylamine--glycine | fig|6666666.462.peg.866;Name=Phosphoribosylamine--glycine ligase (EC6.3.4.13);Ontology_term=KEGG_ENZYME:6.3.4.13 |
| YP_001467331.1| RDD family protein | fig|6666666.462.peg.867;Name=hypothetical protein |
| YP_001467330.1| elongation factor P (EF-P) | fig|6666666.462.peg.868;Name=Outer membrane protein Imp2C required forenvelope biogenesis / Organic solvent tolerance proteinprecursor |
| YP_001467329.1| phosphoglycerate dehydrogenase | fig|6666666.462.peg.869;Name=hypothetical protein |
| YP_001467328.1| polynucleotide | fig|6666666.462.peg.870;Name=Polyribonucleotide nucleotidyltransferase (EC2.7.7.8);Ontology_term=KEGG_ENZYME:2.7.7.8 |
| YP_001467327.1| ribosomal protein S1 | fig|6666666.462.peg.871;Name=Universal stress protein UspA and relatednucleotide-binding proteins |
| YP_001467326.1| 4-hydroxy-3-methylbut-2-enyl | fig|6666666.462.peg.137;Name=hypothetical protein |
| YP_001467325.1| DNA polymerase III subunit | fig|6666666.462 .peg.136;Name=DNA polymerase III alpha subunit (EC 2.7.7.7);Ontology_term=KEGG_ENZYME:2.7.7.7 |
| YP_001467324.1| phenylalanyl-tRNA synthetase | fig|6666666.462.peg.135;Name=hypothetical protein |
| YP_001467323.1| phenylalanyl-tRNA synthetase | fig|6666666.462.peg.139;Name=glutamyl-Q-tRNA synthetase |
| YP_001467322.1| HIT family protein | fig|6666666.462.peg.140;Name=RNA-binding protein |
| YP_001467321.1| DJ-1 family protein | fig|6666666.462.peg.141;Name=4-methyl-5(B-hydroxyethyl)-thiazolemonophosphate biosynthesis enzyme |
| YP_001467320.1| lipopolysaccharide | fig|6666666.462.peg.134;Name=ADP-heptose--lipooligosaccharideheptosyltransferase II (EC2.4.1.-);Ontology_term=KEGG_ENZYME:2.4.1.- |
| YP_001467319.1| glycosyl transferase, group 1 | fig|6666666.462.peg.133;Name=UDP-glucose:(heptosyl) LPSalpha12C3-glucosyltransferase WaaG (EC2.4.1.-);Ontology_term=KEGG_ENZYME:2.4.1.- |
| YP_001467318.1| glycosyl transferase, group 1 | fig|6666666.462.peg.130;Name=Glycosyl transferase2C group 1 |
| YP_001467317.1| NhaD [Campylobacter concisus | fig|6666666.462.peg.129;Name=Polysaccharide deacetylase |
| YP_001467316.1| GMP synthase | fig|6666666.462.peg.126;Name=Polysaccharide deacetylase |
| YP_001467315.1| phosphatidylserine | fig|6666666.462.peg.125;Name=hypothetical protein |
| YP_001467314.1| putative lipopolysaccharide | fig|6666666.462.peg.124;Name=Lipopolysaccharide heptosyltransferase III(EC 2.4.1.-);Ontology_term=KEGG_ENZYME:2.4.1.- |
| YP_001467313.1| glycosyl transferase, group 2 | fig|6666666.462.peg.123;Name=Putative two-domain glycosyltransferase |
| YP_001467312.1| lipid A biosynthesis lauroyl | fig|6666666.462.peg.122;Name=Lipid A biosynthesis lauroylacyltransferase (EC2.3.1.-);Ontology_term=KEGG_ENZYME:2.3.1.- |
| YP_001467311.1| lipopolysaccharide | fig|6666666.462.peg.121;Name=Lipopolysaccharide heptosyltransferase I(EC 2.4.1.-);Ontology_term=KEGG_ENZYME:2.4.1.- |
| YP_001467310.1| large-conductance | fig|6666666.462.peg.120;Name=Polysaccharide biosynthesis protein WlaX |
| YP_001467309.1| glutamyl-tRNA synthetase | fig|6666666.462.peg.119;Name=dTDP-glucose 42C6-dehydratase (EC4.2.1.46);Ontology_term=KEGG_ENZYME:4.2.1.46 |
| YP_001467308.1| yggt family protein | fig|6666666.462.peg.118;Name=DNA-3-methyladenine glycosylase (EC3.2.2.20);Ontology_term=KEGG_ENZYME:3.2.2.20 |
| YP_001467307.1| DNA ligase [Campylobacter | fig|6666666.462.peg.117;Name=DNA ligase (EC6.5.1.1);Ontology_term=KEGG_ENZYME:6.5.1.1 |
| YP_001467306.1| putative soluble lytic murein | fig|6666666.462.peg.116;Name=Flavin-utilizing monoxygenase |
| YP_001467305.1| UDP-glucose 4-epimerase | fig|6666666.462.peg.114;Name=UDP-glucose 4-epimerase (EC5.1.3.2);Ontology_term=KEGG_ENZYME:5.1.3.2 |
| YP_001467304.1| nucleotide sugar dehydrogenase | fig|6666666.462.peg.113;Name=UDP-glucose dehydrogenase (EC1.1.1.22);Ontology_term=KEGG_ENZYME:1.1.1.22 |
| YP_001467303.1| hypothetical protein | fig|6666666.462.peg.112;Name=hypothetical protein |
| YP_001467302.1| nucleotide sugar dehydrogenase | fig|6666666.462.peg.111;Name=UDP-glucose dehydrogenase (EC1.1.1.22);Ontology_term=KEGG_ENZYME:1.1.1.22 |
| YP_001467300.1| polysaccharide biosynthesis | fig|6666666.462.peg.110;Name=unknown |
| YP_001467299.1| ExsB [Campylobacter concisus | fig|6666666.462.peg.109;Name=Beta-12C4-galactosyltransferase |
| YP_001467298.1| glycosyl transferase, group 2 | fig|6666666.462.peg.108;Name=Glycosyltransferase PglI (EC2.4.1.-);Ontology_term=KEGG_ENZYME:2.4.1.- |
| YP_001467297.1| glycosyl transferase, group 1 | fig|6666666.462.peg.107;Name=Alpha-12C4-N-acetylgalactosaminetransferase PglH (EC2.4.1.-);Ontology_term=KEGG_ENZYME:2.4.1.- |
| YP_001467296.1| nitric oxide reductase large | fig|6666666.462.peg.106;Name=Alpha-12C4-N-acetylgalactosaminetransferase PglJ (EC2.4.1.-);Ontology_term=KEGG_ENZYME:2.4.1.- |
| YP_001467295.1| iron chelatin ABC transporter, | fig|6666666.462.peg.105;Name=Alpha-12C4-N-acetylgalactosaminetransferase PglJ (EC2.4.1.-);Ontology_term=KEGG_ENZYME:2.4.1.- |
| YP_001467294.1| iron chelatin ABC transporter, | fig|6666666.462.peg.104;Name=Oligosaccharyltransferase PglB (EC2.4.1.119);Ontology_term=KEGG_ENZYME:2.4.1.119 |
| YP_001467293.1| iron compounds ABC | fig|6666666.462.peg.103;Name=Alpha-12C3-N-acetylgalactosaminetransferase PglA (EC2.4.1.-);Ontology_term=KEGG_ENZYME:2.4.1.- |
| YP_001467292.1| Cpp29 [Campylobacter concisus | fig|6666666.462.peg.102;Name=Lipid carrier :UDP-N-acetylgalactosaminyltransferase (EC2.4.1.-);Ontology_term=KEGG_ENZYME:2.4.1.- |
| YP_001467291.1| general glycosylation pathway | fig|6666666.462.peg.101;Name=4-amino-6-deoxy-N-Acetyl-D-hexosaminyl-(Lipid carrier) acetyltrasferase |
| YP_001467290.1| processing protease | fig|6666666.462.peg.100;Name=4-keto-6-deoxy-N-Acetyl-D-hexosaminyl-(Lipid carrier) aminotransferase |
| YP_001467289.1| FO synthase subunit 2 | fig|6666666.462.peg.99;Name=UDP-N-acetylglucosamine 42C6-dehydratase(EC 4.2.1.-);Ontology_term=KEGG_ENZYME:4.2.1.- |
| YP_001467288.1| hypothetical protein | fig|6666666.462.peg.98;Name=N-linked glycosylation glycosyltransferasePglG |
| YP_001467287.1| imidazole glycerol phosphate | fig|6666666.462.peg.97;Name=Imidazole glycerol phosphate synthaseamidotransferase subunit (EC2.4.2.-);Ontology_term=KEGG_ENZYME:2.4.2.- |
| YP_001467286.1| | fig|6666666.462.peg.96;Name=Phosphoribosylformimino-5-aminoimidazolecarboxamide ribotide isomerase (EC5.3.1.16);Ontology_term=KEGG_ENZYME:5.3.1.16 |
| YP_001467285.1| lipoprotein [Campylobacter | fig|6666666.462.peg.95;Name=Chemotaxis regulator - transmitschemoreceptor signals to flagelllar motor componentsCheY |
| YP_001467284.1| ribosomal protein L11 | fig|6666666.462.peg.94;Name=Ribosomal protein L11 methyltransferase (EC2.1.1.-);Ontology_term=KEGG_ENZYME:2.1.1.- |
| YP_001467283.1| putative cell division | fig|6666666.462.peg.93;Name=Cell division protein FtsH (EC3.4.24.-);Ontology_term=KEGG_ENZYME:3.4.24.- |
| YP_001467282.1| phosphatidylserine | fig|6666666.462.peg.92;Name=Phosphatidylserine decarboxylase-relatedprotein |
| YP_001467281.1| argininosuccinate lyase | fig|6666666.462.peg.91;Name=CDP-diacylglycerol--serineO-phosphatidyltransferase (EC2.7.8.8);Ontology_term=KEGG_ENZYME:2.7.8.8 |
| YP_001467280.1| 2-isopropylmalate synthase | fig|6666666.462.peg.90;Name=2-isopropylmalate synthase (EC2.3.3.13);Ontology_term=KEGG_ENZYME:2.3.3.13 |
| YP_001467279.1| heavy metal | fig|6666666.462.peg.156;Name=Enterobactin receptor VctA |
| YP_001467278.1| acetyltransferase | fig|6666666.462.peg.155;Name=Malate:quinone oxidoreductase (EC1.1.99.16);Ontology_term=KEGG_ENZYME:1.1.99.16 |
| YP_001467277.1| hypothetical protein | fig|6666666.462.peg.152;Name=hypothetical protein |
| YP_001467276.1| peptidyl-prolyl cis-trans | fig|6666666.462.peg.1689;Name=ABC transporter ATP-binding protein uup |
| YP_001467275.1| aerobic C4-dicarboxylate | fig|6666666.462.peg.1688;Name=hypothetical protein |
| YP_001467274.1| glucose-6-phosphate isomerase | fig|6666666.462.peg.1687;Name=Glucose-6-phosphate isomerase (EC5.3.1.9);Ontology_term=KEGG_ENZYME:5.3.1.9 |
| YP_001467273.1| UTP-glucose-1-phosphate | fig|6666666.462.peg.1686;Name=UTP--glucose-1-phosphateuridylyltransferase (EC2.7.7.9);Ontology_term=KEGG_ENZYME:2.7.7.9 |
| YP_001467272.1| phosphoenolpyruvate | fig|6666666.462.peg.1685;Name=FIG000605: protein co-occurring withtransport systems (COG1739) |
| YP_001467271.1| prolipoprotein diacylglyceryl | fig|6666666.462.peg.1684;Name=Prolipoprotein diacylglyceryl transferase(EC 2.4.99.-);Ontology_term=KEGG_ENZYME:2.4.99.- |
| YP_001467270.1| fumarate reductase respiratory | fig|6666666.462.peg.1683;Name=Fumarate reductase cytochrome b subunit |
| YP_001467269.1| fumarate reductase | fig|6666666.462.peg.1682;Name=Succinate dehydrogenase flavoproteinsubunit (EC 1.3.99.1);Ontology_term=KEGG_ENZYME:1.3.99.1 |
| YP_001467268.1| fumarate reductase iron-sulfur | fig|6666666.462.peg.1681;Name=Succinate dehydrogenase iron-sulfur protein(EC 1.3.99.1);Ontology_term=KEGG_ENZYME:1.3.99.1 |
| YP_001467266.1| sodium- and chloride-dependent | fig|6666666.462.peg.1679;Name=Mrr restriction system protein |
| YP_001467265.1| phosphatidate | fig|6666666.462.peg.1672;Name=Phosphatidate cytidylyltransferase (EC2.7.7.41);Ontology_term=KEGG_ENZYME:2.7.7.41 |
| YP_001467264.1| 1-deoxy-D-xylulose 5-phosphate | fig|6666666.462.peg.1673;Name=1-deoxy-D-xylulose 5-phosphatereductoisomerase (EC1.1.1.267);Ontology_term=KEGG_ENZYME:1.1.1.267 |
| YP_001467263.1| permease subfamily protein | fig|6666666.462.peg.1674;Name=Uracil permease |
| YP_001467262.1| hypothetical protein | fig|6666666.462.peg.1675;Name=Putative periplasmic protein |
| YP_001467261.1| O-sialoglycoprotein | fig|6666666.462.peg.1676;Name=FIG134348: essential endopeptidase |
| YP_001467260.1| sodium/proline symporter | fig|6666666.462.peg.1301;Name=Pantothenate:Na+ symporter (TC 2.A.21.1.1) |
| YP_001467259.1| His/Glu/Gln/Arg/opine amino | fig|6666666.462.peg.1300;Name=Putative amino acid ABC tansporter permeaseprotein |
| YP_001467258.1| putative ABC transporter, | fig|6666666.462.peg.1299;Name=Putative glutamine transport ATP-bindingprotein |
| YP_001467257.1| homoserine kinase (HSK; HK) | fig|6666666.462.peg.1298;Name=Histidine-binding protein precursor |
| YP_001467256.1| UDP-3-O-[3-hydroxymyristoyl] | fig|6666666.462.peg.1516;Name=Histidine-binding protein precursor |
| YP_001467255.1| N- methylation [Campylobacter | fig|6666666.462.peg.1515;Name=hypothetical protein |
| YP_001467254.1| hypothetical protein | fig|6666666.462.peg.653;Name=hypothetical protein |
| YP_001467253.1| putative nucleotide | fig|6666666.462.peg.652;Name=hypothetical protein |
| YP_001467252.1| antibiotic biosynthesis | fig|6666666.462.peg.651;Name=hypothetical protein |
| YP_001467251.1| radical SAM domain-containing | fig|6666666.462.peg.650;Name=Biotin sulfoxide reductase (EC1.-.-.-);Ontology_term=KEGG_ENZYME:1.-.-.- |
| YP_001467250.1| putative GlnD family protein | fig|6666666.462.peg.649;Name=low-specificity D-threonine aldolase |
| YP_001467249.1| glucosamine | fig|6666666.462.peg.1678;Name=Putative ATP /GTP binding protein |
| YP_001467248.1| dynamin family protein | fig|6666666.462.peg.1677;Name=Putative ATP /GTP binding protein |
| YP_001467245.1| thiamine-phosphate | fig|6666666.462.peg.143;Name=Thiamin-phosphate pyrophosphorylase (EC2.5.1.3);Ontology_term=KEGG_ENZYME:2.5.1.3 |
| YP_001467244.1| phosphomethylpyrimidine kinase | fig|6666666.462.peg.144;Name=Phosphomethylpyrimidine kinase (EC2.7.4.7);Ontology_term=KEGG_ENZYME:2.7.4.7 |
| YP_001467243.1| tetrahydrodipicolinate | fig|6666666.462.peg.145;Name=Putative aminotransferase |
| YP_001467242.1| ATPase [Campylobacter concisus | fig|6666666.462.peg.146;Name=hypothetical protein |
| YP_001467241.1| sensor histidine kinase | fig|6666666.462.peg.147;Name=Flagellar sensory histidine kinase FlgS |
| YP_001467240.1| hypothetical protein | fig|6666666.462.peg.148;Name=Nicotinamidase (EC3.5.1.19);Ontology_term=KEGG_ENZYME:3.5.1.19 |
| YP_001467239.1| TonB-system energizer ExbB | fig|6666666.462.peg.149;Name=Ferric siderophore transport system2Cbiopolymer transport protein ExbB |
| YP_001467238.1| TonB system transport protein | fig|6666666.462.peg.150;Name=Biopolymer transport protein ExbD/TolR |
| YP_001467237.1| ATP-binding protein | fig|6666666.462.peg.151;Name=Ferric siderophore transport system2Cperiplasmic binding protein TonB |
| YP_001467236.1| thiamine biosynthesis protein | fig|6666666.462.peg.1690;Name=Aspartate ammonia-lyase (EC4.3.1.1);Ontology_term=KEGG_ENZYME:4.3.1.1 |
| YP_001467235.1| IspD/IspF bifunctional enzyme | fig|6666666.462.peg.1691;Name=C4-dicarboxylate transporter DcuA |
| YP_001467234.1| response regulator receiver | fig|6666666.462.peg.1692;Name=polysulfide reductase2C subunit A |
| YP_001467233.1| anaerobic dimethyl sulfoxide | fig|6666666.462.peg.1693;Name=polysulfide reductase2C subunit B |
| YP_001467232.1| phosphatidylglycerophosphatase | fig|6666666.462.peg.1694;Name=polysulfide reductase2C subunit C |
| YP_001467231.1| two component transcriptional | fig|6666666.462.peg.1695;Name=hypothetical protein |
| YP_001467230.1| putative two-component sensor | fig|6666666.462.peg.1696;Name=hypothetical protein |
| YP_001467229.1| Holliday junction DNA helicase | fig|6666666.462.peg.1697;Name=Holliday junction DNA helicase RuvB |
| YP_001467228.1| putative cytochrome c family | fig|6666666.462.peg.1698;Name=hypothetical protein |
| YP_001467227.1| major outer membrane protein | fig|6666666.462.peg.1699;Name=hypothetical protein |
| YP_001467226.1| hypothetical protein | fig|6666666.462.peg.1700;Name=Acid membrane antigen A |
| YP_001467225.1| HIT family protein | fig|6666666.462.peg.1701;Name=Aminopeptidase YpdF (MP-2C MA-2C MS-2CAP-2C NP- specific) |
| YP_001467224.1| 3-methyl-2-oxobutanoate | fig|6666666.462.peg.1703;Name=3-methyl-2-oxobutanoatehydroxymethyltransferase (EC2.1.2.11);Ontology_term=KEGG_ENZYME:2.1.2.11 |
| YP_001467223.1| hypothetical protein | fig|6666666.462.peg.1704;Name=hypothetical protein |
| YP_001467221.1| signal peptide peptidase SppA, | fig|6666666.462.peg.1707;Name=protease IV (PspA) |
| YP_001467220.1| chlorohydrolase [Campylobacter | fig|6666666.462.peg.1708;Name=Chlorohydrolase/deaminase family protein |
| YP_001467219.1| 3-dehydroquinate dehydratase | fig|6666666.462.peg.1709;Name=3-dehydroquinate dehydratase II (EC4.2.1.10);Ontology_term=KEGG_ENZYME:4.2.1.10 |
| YP_001467218.1| DNA polymerase III gamma and | fig|6666666.462.peg.1710;Name=Aminopeptidase YpdF (MP-2C MA-2C MS-2CAP-2C NP- specific) |
| YP_001467217.1| | fig|6666666.462.peg.1711;Name=2-amino-4-hydroxy-6-hydroxymethyldihydropteridine pyrophosphokinase (EC2.7.6.3);Ontology_term=KEGG_ENZYME:2.7.6.3 |
| YP_001467216.1| flagellar biosynthesis | fig|6666666.462.peg.1712;Name=Flagellar biosynthesis protein FlhF |
| YP_001467215.1| histidinol phosphatase | fig|6666666.462.peg.1713;Name=Flagellar synthesis regulator FleN |
| YP_001467214.1| hypothetical protein | fig|6666666.462.peg.1714;Name=Motility integral membrane protein |
| YP_001467213.1| flagellar biosynthesis sigma | fig|6666666.462.peg.1715;Name=RNA polymerase sigma factor for flagellaroperon |
| YP_001467212.1| flagellar motor switch protein | fig|6666666.462.peg.1716;Name=Flagellar motor switch protein FliM |
| YP_001467211.1| flagellar motor switch protein | fig|6666666.462.peg.1717;Name=Flagellar motor switch protein FliN |
| YP_001467210.1| hypothetical protein | fig|6666666.462.peg.1718;Name=Predicted Rossmann fold nucleotide-bindingprotein2C possible lysine decarboxylase |
| YP_001467209.1| tRNA | fig|6666666.462.peg.1719;Name=tRNA(5-methylaminomethyl-2-thiouridylate)-methyltransferase(EC 2.1.1.61);Ontology_term=KEGG_ENZYME:2.1.1.61 |
| YP_001467208.1| glycyl-tRNA synthetase alpha | fig|6666666.462.peg.1720;Name=hypothetical protein |
| YP_001467207.1| ribose-phosphate | fig|6666666.462.peg.1721;Name=Ribose-phosphate pyrophosphokinase (EC2.7.6.1);Ontology_term=KEGG_ENZYME:2.7.6.1 |
| YP_001467206.1| hypothetical protein | fig|6666666.462.peg.33;Name=membrane protein |
| YP_001467205.1| uroporphyrinogen decarboxylase | fig|6666666.462.peg.32;Name=Uroporphyrinogen III decarboxylase (EC4.1.1.37);Ontology_term=KEGG_ENZYME:4.1.1.37 |
| YP_001467204.1| signal recognition particle | fig|6666666.462.peg.31;Name=Putative Fe-S oxidoreductase |
| YP_001467203.1| phosphomethylpyrimidine kinase | fig|6666666.462.peg.30;Name=Pyridoxal kinase (EC2.7.1.35);Ontology_term=KEGG_ENZYME:2.7.1.35 |
| YP_001467201.1| ATP-dependent protease La | fig|6666666.462.peg.1837;Name=ATP-dependent protease La (EC 3.4.21.53) TypeI;Ontology_term=KEGG_ENZYME:3.4.21.53 |
| YP_001467200.1| tRNA | fig|6666666.462.peg.1838;Name=Putative lipoprotein |
| YP_001467199.1| flagellar assembly protein | fig|6666666.462.peg.1839;Name=Flagellar assembly factor FliW |
| YP_001467198.1| hypothetical protein | fig|6666666.462.peg.1 840;Name=internalin2C putative |
| YP_001467197.1| prepilin-type N- terminal | fig|6666666.462.peg.1841;Name=hypothetical protein |
| YP_001467196.1| prepilin-type N-terminal | fig|6666666.462.peg.1842;Name=hypothetical protein |
| YP_001467195.1| hypothetical protein | fig|6666666.462.peg.1843;Name=hypothetical protein |
| YP_001467194.1| CRISPR-associated protein Cas2 | fig|6666666.462.peg.1844;Name=Ribosomal subunit interface protein |
| YP_001467193.1| peptidase propeptide/YPEB | fig|6666666.462.peg.1734;Name=Putative protease ydgD (EC3.4.21.-);Ontology_term=KEGG_ENZYME:3.4.21.- |
| YP_001467191.1| response regulator receiver | fig|6666666.462.peg.1737;Name=Two-component response regulator czcR |
| YP_001467190.1| hypothetical protein | fig|6666666.462.peg.1738;Name=Transcription-repair coupling factor |
| YP_001467188.1| radical SAM domain-containing | fig|6666666.462.peg.1740;Name=Nitrogenase FeMo-cofactor synthesis FeScore scaffold and assembly protein NifB |
| YP_001467187.1| riboflavin biosynthesis | fig|6666666.462.peg.1741;Name=hypothetical protein |
| YP_001467186.1| type I | fig|6666666.462.peg.1742;Name=Molybdenum ABC transporter2C periplasmicmolybdenum-binding protein ModA (TC 3.A.1.8.1) |
| YP_001467185.1| anti-codon nuclease masking | fig|6666666.462.peg .1743;Name=hypothetical protein |
| YP_001467184.1| inorganic diphosphatase | fig|6666666.462.peg.1745;Name=Inorganic pyrophosphatase (EC3.6.1.1);Ontology_term=KEGG_ENZYME:3.6.1.1 |
| YP_001467183.1| protein YgiW [Campylobacter | fig|6666666.462.peg.1746;Name=hypothetical protein |
| YP_001467182.1| adenylate kinase | fig|6666666.462.peg.1747;Name=Adenylate kinase (EC2.7.4.3);Ontology_term=KEGG_ENZYME:2.7.4.3 |
| YP_001467181.1| aspartyl-tRNA synthetase | fig|6666666.462.peg.1748;Name=Aspartyl-tRNA synthetase (EC 6.1.1.12) @Aspartyl-tRNA(Asn) synthetase (EC6.1.1.23);Ontology_term=KEGG_ENZYME:6.1.1.12,KEGG_ENZYME:6.1.1.23 |
| YP_001467180.1| Na+/H+ antiporter family | fig|6666666.462.peg.1749;Name=NAD kinase (EC2.7.1.23);Ontology_term=KEGG_ENZYME:2.7.1.23 |
| YP_001467179.1| tRNA | fig|6666666.462.peg.1750;Name=DNA repair protein RecN |
| YP_001467178.1| threonine dehydratase | fig|6666666.462.peg.1751;Name=Putative two-component response regulator |
| YP_001467177.1| glyoxalase II [Campylobacter | fig|6666666.462.peg.1752;Name=Putative deoxyribonuclease YcfH |
| YP_001467176.1| hypothetical protein | fig|6666666.462.peg.1753;Name=hypothetical protein |
| YP_001467175.1| Dyp-type peroxidase | fig|6666666.462.peg.1754;Name=Membrane-bound lytic mureintransglycosylase D precursor (EC3.2.1.-);Ontology_term=KEGG_ENZYME:3.2.1.- |
| YP_001467174.1| drug/metabolite exporter (DME) | fig|6666666.462.peg.1755;Name=RlpA-like lipoprotein precursor |
| YP_001467173.1| imidazoleglycerol-phosphate | fig|6666666.462.peg.1756;Name=Imidazoleglycerol-phosphate dehydratase (EC4.2.1.19);Ontology_term=KEGG_ENZYME:4.2.1.19 |
| YP_001467172.1| 3-deoxy-D-manno-octulosonate | fig|6666666.462.peg.1757;Name=3-deoxy-D-manno-octulosonate 8-phosphatephosphatase (EC3.1.3.45);Ontology_term=KEGG_ENZYME:3.1.3.45 |
| YP_001467171.1| hypothetical protein | fig|6666666.462.peg.1758;Name=hypothetical protein |
| YP_001467170.1| cell envelope biogenesis | fig|6666666.462.peg.1759;Name=OstA family organic solvent toleranceprotein |
| YP_001467169.1| GTPase EngB [Campylobacter | fig|6666666.462.peg.1760;Name=GTP-binding protein EngB |
| YP_001467168.1| hypothetical protein | fig|6666666.462.peg.1761;Name=membrane protein |
| YP_001467167.1| penicillin-binding protein 2 | fig|6666666.462.peg.1762;Name=Cell division protein FtsI [Peptidoglycansynthetase] (EC2.4.1.129);Ontology_term=KEGG_ENZYME:2.4.1.129 |
| YP_001467166.1| 30S ribosomal protein S18 | fig|6666666.462.peg.1845;Name=SSU ribosomal protein S18p |
| YP_001467165.1| amino acid carrier protein | fig|6666666.462.peg.1846;Name=Single-stranded DNA-binding protein |
| YP_001467164.1| 30S ribosomal protein S6 | fig|6666666.462.peg.1847;Name=SSU ribosomal protein S6p |
| YP_001467163.1| DNA polymerase III subunit | fig|6666666.462.peg.1848;Name=hypothetical protein |
| YP_001467162.1| hypothetical protein | fig|6666666.462.peg.1849;Name=3'-to-5' exoribonuclease RNase R |
| YP_001467161.1| HDOD domain-contain protein | fig|6666666.462.peg.1850;Name=Predicted signal transduction protein |
| YP_001467159.1| ketol-acid reductoisomerase | fig|6666666.462.peg.1851;Name=Ketol-acid reductoisomerase (EC1.1.1.86);Ontology_term=KEGG_ENZYME:1.1.1.86 |
| YP_001467158.1| ferric-uptake regulator | fig|6666666.462.peg.1852;Name=Putative periplasmic protein |
| YP_001467157.1| DNA protecting protein DprA | fig|6666666.462.peg.1853;Name=SMF family protein2C DNA processing chain A(dprA) |
| YP_001467155.1| sec-independent translocase | fig|6666666.462.peg.1858;Name=Twin-arginine translocation protein TatB |
| YP_001467153.1| PepSY-associated TM helix | fig|6666666.462.peg.1863;Name=hypothetical protein |
| YP_001467152.1| dinucleoside polyphosphate | fig|6666666.462.peg.1864;Name=Adenosine (5')-pentaphospho-(5'')-adenosinepyrophosphohydrolase (EC3.6.1.-);Ontology_term=KEGG_ENZYME:3.6.1.- |
| YP_001467151.1| aspartate kinase | fig|6666666.462.peg.1865;Name=Aspartokinase (EC2.7.2.4);Ontology_term=KEGG_ENZYME:2.7.2.4 |
| YP_001467150.1| hypothetical protein | fig|6666666.462.peg.1866;Name=hypothetical protein |
| YP_001467149.1| DNA polymerase III subunit | fig|6666666.462.peg.1867;Name=DNA poymerase III subunit delta' (EC2.7.7.7);Ontology_term=KEGG_ENZYME:2.7.7.7 |
| YP_001467148.1| putative dihydropteroate | fig|6666666.462.peg.1868;Name=Alternative dihydrofolate reductase 2 /Dihydropteroate synthase (EC2.5.1.15);Ontology_term=KEGG_ENZYME:2.5.1.15 |
| YP_001467147.1| hypothetical protein | fig|6666666.462.peg.1869;Name=hypothetical protein |
| YP_001467146.1| NAD-dependent DNA ligase LigA | fig|6666666.462.peg.1870;Name=DNA ligase (EC6.5.1.2);Ontology_term=KEGG_ENZYME:6.5.1.2 |
| YP_001467145.1| ribosomal RNA large subunit | fig|6666666.462.peg.1871;Name=RNA binding methyltransferase FtsJ like |
| YP_001467144.1| bifunctional riboflavin | fig|6666666.462.peg.1872;Name=Riboflavin kinase (EC 2.7.1.26) / FMNadenylyltransferase (EC2.7.7.2);Ontology_term=KEGG_ENZYME:2.7.1.26,KEGG_ENZYME:2.7.7.2 |
| YP_001467143.1| putative methyltransferase | fig|6666666.462.peg.1873;Name=tRNA (uridine-5-oxyacetic acid methylester) 34 synthase |
| YP_001467138.1| putative Myb2 protein | fig|6666666.462.peg.1875;Name=NAD-dependent formate dehydrogenase alphasubunit @ selenocysteine-containing |
| YP_001467136.1| 30S ribosomal protein S15 | fig|6666666.462.peg.1876;Name=SSU ribosomal protein S15p (S13e) |
| YP_001467135.1| Rrf2 family protein (putative | fig|6666666.462.peg.1877;Name=Rrf2 family transcriptional regulator |
| YP_001467134.1| flagellar biosynthesis protein | fig|6666666.462.peg.1394;Name=Flagellar biosynthesis protein FlhA |
| YP_001467133.1| hypothetical protein | fig|6666666.462.peg.1393;Name=3'-to-5' oligoribonuclease B2C Bacillustype |
| YP_001467132.1| cell division protein | fig|6666666.462.peg.1392;Name=hypothetical protein |
| YP_001467131.1| molybdopterin biosynthesis | fig|6666666.462.peg.1391;Name=hypothetical protein |
| YP_001467130.1| aspartate carbamoyltransferase | fig|6666666.462.peg.1390;Name=Aspartate carbamoyltransferase (EC2.1.3.2);Ontology_term=KEGG_ENZYME:2.1.3.2 |
| YP_001467129.1| chaperone protein HtpG (heat | fig|6666666.462.peg.1389;Name=Dihydroorotase (EC3.5.2.3);Ontology_term=KEGG_ENZYME:3.5.2.3 |
| YP_001467128.1| aminoacyl-histidine | fig|6666666.462.peg.1388;Name=Aminoacyl-histidine dipeptidase (PeptidaseD) (EC 3.4.13.3);Ontology_term=KEGG_ENZYME:3.4.13.3 |
| YP_001467127.1| hypothetical protein | fig|6666666.462.peg.1373;Name=hypothetical protein |
| YP_001467126.1| hypothetical protein | fig|6666666.462.peg.1372;Name=hypothetical protein |
| YP_001467125.1| heat-inducible transcription | fig|6666666.462.peg.1371;Name=Heat-inducible transcription repressor HrcA |
| YP_001467124.1| co-chaperone GrpE | fig|6666666.462.peg.1370;Name=Heat shock protein GrpE |
| YP_001467123.1| molecular chaperone DnaK | fig|6666666.462.peg.1369;Name=Chaperone protein DnaK |
| YP_001467122.1| DNA polymerase III epsilon | fig|6666666.462.peg.1380;Name=hypothetical |
| YP_001467121.1| DegT/DnrJ/EryC1/StrS | fig|6666666.462.peg.1378;Name=UDP-4-amino-4-deoxy-L-arabinose--oxoglutarate aminotransferase (EC2.6.1.-);Ontology_term=KEGG_ENZYME:2.6.1.- |
| YP_001467120.1| threonine synthase | fig|6666666.462.peg.1375;Name=Threonine synthase (EC4.2.3.1);Ontology_term=KEGG_ENZYME:4.2.3.1 |
| YP_001467119.1| 3-deoxy-manno-octulosonate | fig|6666666.462.peg.1374;Name=3-deoxy-manno-octulosonatecytidylyltransferase (EC2.7.7.38);Ontology_term=KEGG_ENZYME:2.7.7.38 |
| YP_001467116.1| divalent cation transporter | fig|6666666.462.peg.1366;Name=Cytochrome c-type biogenesis protein DsbD2Cprotein-disulfide reductase (EC1.8.1.8);Ontology_term=KEGG_ENZYME:1.8.1.8 |
| YP_001467115.1| polyphosphate kinase 2 | fig|6666666.462.peg.1367;Name=UDP-galactose-lipid carrier transferase (EC2.-.-.-);Ontology_term=KEGG_ENZYME:2.-.-.- |
| YP_001467113.1| tRNA-processing ribonuclease | fig|6666666.462.peg.1368;Name=hypothetical protein |
| YP_001467111.1| thioesterase superfamily | fig|6666666.462.peg.1382;Name=Hot dog fold protein HP0420 |
| YP_001467110.1| hypothetical protein | fig|6666666.462.peg.1383;Name=tRNA (5-methoxyuridine) 34 synthase |
| YP_001467109.1| hypothetical protein | fig|6666666.462.peg.1384;Name=Putative lipoprotein |
| YP_001467108.1| DNA-binding response regulator | fig|6666666.462.peg.1385;Name=Transglutaminase-like enzymes2C putativecysteine proteases |
| YP_001467107.1| hypothetical protein | fig|6666666.462.peg.1386;Name=hypothetical protein |
| YP_001467106.1| mercuric transport protein | fig|6666666.462.peg.1387;Name=Periplasmic mercury(+2) binding protein |
| YP_001467105.1| holo-[acyl-carrier-protein] | fig|6666666.462.peg.1364;Name=Peptidyl-prolyl cis-trans isomerase ppiD (EC5.2.1.8);Ontology_term=KEGG_ENZYME:5.2.1.8 |
| YP_001467104.1| fructose-bisphosphate aldolase | fig|6666666.462.peg.1363;Name=Fructose-bisphosphate aldolase class II (EC4.1.2.13);Ontology_term=KEGG_ENZYME:4.1.2.13 |
| YP_001467103.1| hypothetical protein | fig|6666666.462.peg.1067;Name=membrane protein |
| YP_001467102.1| putative chemotaxis protein | fig|6666666.462.peg.1066;Name=Putative periplasmic protein |
| YP_001467101.1| | fig|6666666.462.peg.1065;Name=pyridoxal phosphate-dependentdeaminase2C putative |
| YP_001467100.1| histidinol dehydrogenase | fig|6666666.462.peg.1064;Name=Histidinol dehydrogenase (EC1.1.1.23);Ontology_term=KEGG_ENZYME:1.1.1.23 |
| YP_001467099.1| hypothetical protein | fig|6666666.462.peg.1063;Name=Flagellin C |
| YP_001467098.1| RNA polymerase sigma factor | fig|6666666.462.peg.1062;Name=RNA polymerase sigma factor RpoD |
| YP_001467097.1| flagellar basal-body rod | fig|6666666.462.peg.1061;Name=Flagellar basal-body rod protein FlgF |
| YP_001467096.1| flagellar basal body rod | fig|6666666.462.peg.1060;Name=Flagellar basal-body rod protein FlgG |
| YP_001467095.1| HD/HDIG/KH domain-containing | fig|6666666.462.peg.1059;Name=hypothetical protein |
| YP_001467094.1| integral membrane protein | fig|6666666.462.peg.1057;Name=hypothetical protein |
| YP_001467093.1| Sel1 repeat-containing protein | fig|6666666.462.peg.1056;Name=hypothetical protein |
| YP_001467092.1| branched-chain amino acid | fig|6666666.462.peg.1054;Name=branched chain amino acid transportprotein AzlD |
| YP_001467091.1| L-asparaginase [Campylobacter | fig|6666666.462.peg.1053;Name=L-asparaginase (EC3.5.1.1);Ontology_term=KEGG_ENZYME:3.5.1.1 |
| YP_001467088.1| LysE family translocator | fig|6666666.462.peg.1050;Name=Putative threonine efflux protein |
| YP_001467087.1| hypothetical protein | fig|6666666.462.peg.1048;Name=hypothetical protein |
| YP_001467085.1| 3-isopropylmalate dehydratase | fig|6666666.462.peg.1046;Name=3-isopropylmalate dehydratase smallsubunit (EC 4.2.1.33);Ontology_term=KEGG_ENZYME:4.2.1.33 |
| YP_001467084.1| 3-isopropylmalate | fig|6666666.462.peg.1045;Name=3-isopropylmalate dehydrogenase (EC1.1.1.85);Ontology_term=KEGG_ENZYME:1.1.1.85 |
| YP_001467083.1| hypothetical protein | fig|6666666.462.peg.1044;Name=hypothetical protein |
| YP_001467082.1| OorC subunit of | fig|6666666.462.peg.1043;Name=hypothetical protein |
| YP_001467081.1| tRNA | fig|6666666.462.peg.1042;Name=tRNA nucleotidyltransferase (EC 2.7.7.21)(EC2.7.7.25);Ontology_term=KEGG_ENZYME:2.7.7.21,KEGG_ENZYME:2.7.7.25 |
| YP_001467080.1| RNA methyltransferase | fig|6666666.462.peg.1041;Name=Putative RNA methyltransferase (EC2.1.1.-);Ontology_term=KEGG_ENZYME:2.1.1.- |
| YP_001467079.1| putative | fig|6666666.462.peg.1040;Name=hypothetical protein |
| YP_001467078.1| putative ribosomal RNA adenine | fig|6666666.462.peg.1039;Name=hypothetical protein |
| YP_001467077.1| glycyl-tRNA synthetase subunit | fig|6666666.462.peg.1038;Name=Glycyl-tRNA synthetase beta chain (EC6.1.1.14);Ontology_term=KEGG_ENZYME:6.1.1.14 |
| YP_001467076.1| iron(III) dicitrate-binding | fig|6666666.462.peg.1037;Name=membrane protein |
| YP_001467075.1| hypothetical protein | fig|6666666.462.peg.1037;Name=membrane protein |
| YP_001467074.1| hypothetical protein | fig|6666666.462.peg.1036;Name=hypothetical protein |
| YP_001467073.1| succinate dehydrogenase, | fig|6666666.462.peg.1035;Name=Multimodulartranspeptidase-transglycosylase (EC 2.4.1.129) (EC3.4.-.-);Ontology_term=KEGG_ENZYME:2.4.1.129,KEGG_ENZYME:3.4.-.- |
| YP_001467072.1| Maf-like protein | fig|6666666.462.peg.1034;Name=Maf-like protein Cj0507 |
| YP_001467071.1| hypothetical protein | fig|6666666.462.peg.1033;Name=hypothetical protein |
| YP_001467070.1| alanyl-tRNA synthetase | fig|6666666.462.peg.1032;Name=Alanyl-tRNA synthetase (EC6.1.1.7);Ontology_term=KEGG_ENZYME:6.1.1.7 |
| YP_001467069.1| thiazole biosynthesis protein | fig|6666666.462.peg.1031;Name=Predicted dehydrogenases and relatedproteins |
| YP_001467068.1| ferrochelatase [Campylobacter | fig|6666666.462.peg.1030;Name=Ferrochelatase2C protoheme ferro-lyase(EC 4.99.1.1);Ontology_term=KEGG_ENZYME:4.99.1.1 |
| YP_001467067.1| hypothetical protein | fig|6666666.462.peg.1029;Name=hypothetical protein |
| YP_001467066.1| hypothetical protein | fig|6666666.462.peg.1028;Name=Cell division protein FtsI [Peptidoglycansynthetase] (EC2.4.1.129);Ontology_term=KEGG_ENZYME:2.4.1.129 |
| YP_001467065.1| flagellar hook-basal body | fig|6666666.462.peg.1027;Name=Flagellar hook-basal body complexprotein FliE |
| YP_001467064.1| flagellar basal body rod | fig|6666666.462.peg.1026;Name=Flagellar basal-body rod protein FlgC |
| YP_001467063.1| flagellar basal body rod | fig|6666666.462.peg.1025;Name=Flagellar basal-body rod protein FlgB |
| YP_001467062.1| thioredoxin domain-containing | fig|6666666.462.peg.1024;Name=Possible periplasmic thioredoxin |
| YP_001467061.1| SsrA-binding protein | fig|6666666.462.peg.1023;Name=tmRNA-binding protein SmpB |
| YP_001467060.1| | fig|6666666.462.peg.1022;Name=4-diphosphocytidyl-2-C-methyl-D-erythritol kinase (EC2.7.1.148);Ontology_term=KEGG_ENZYME:2.7.1.148 |
| YP_001467059.1| carbon storage regulator | fig|6666666.462.peg.1021;Name=Carbon storage regulator |
| YP_001467058.1| tRNA pseudouridine synthase B | fig|6666666.462.peg.1020;Name=tRNA pseudouridine synthase B (EC4.2.1.70);Ontology_term=KEGG_ENZYME:4.2.1.70 |
| YP_001467057.1| UDP-N-acetylglucosamine | fig|6666666.462.peg.1019;Name=ATP-dependent DNA helicaseUvrD/PcrA/Rep2C epsilon proteobacterial type 2 |
| YP_001467056.1| hypothetical protein | fig|6666666.462.peg.1018;Name=hypothetical protein |
| YP_001467055.1| rhomboid family protein | fig|6666666.462.peg.1017;Name=Oligoendopeptidase F (EC3.4.24.-);Ontology_term=KEGG_ENZYME:3.4.24.- |
| YP_001467054.1| serine/threonine transporter | fig|6666666.462.peg.1016;Name=Sodium/dicarboxylate symporter |
| YP_001467053.1| S-adenosylmethionine | fig|6666666.462.peg.1015;Name=S-adenosylmethionine synthetase (EC2.5.1.6);Ontology_term=KEGG_ENZYME:2.5.1.6 |
| YP_001467052.1| apolipoprotein | fig|6666666.462.peg.1014;Name=Apolipoprotein N-acyltransferase (EC2.3.1.-);Ontology_term=KEGG_ENZYME:2.3.1.- |
| YP_001467051.1| preprotein translocase subunit | fig|6666666.462.peg.1013;Name=Preprotein translocase subunit YajC(TC 3.A.5.1.1) |
| YP_001467050.1| preprotein translocase subunit | fig|6666666.462.peg.1012;Name=Protein-export membrane protein SecD (TC3.A.5.1.1) |
| YP_001467049.1| preprotein translocase subunit | fig|6666666.462.peg.1011;Name=Protein-export membrane protein SecF (TC3.A.5.1.1) |
| YP_001467048.1| MacA [Campylobacter concisus | fig|6666666.462.peg.1010;Name=conserved hypothetical integral membraneprotein |
| YP_001467047.1| leucyl-tRNA synthetase | fig|6666666.462.peg.1009;Name=Leucyl-tRNA synthetase (EC6.1.1.4);Ontology_term=KEGG_ENZYME:6.1.1.4 |
| YP_001467046.1| penicillin-binding protein | fig|6666666.462.peg.1008;Name=Probable lipoprotein Cj1090c |
| YP_001467045.1| GGDEF domain-containing | fig|6666666.462.peg.1007;Name=hypothetical protein |
| YP_001467044.1| valyl-tRNA synthetase | fig|6666666.462.peg.1006;Name=Dihydrofolate synthase (EC 6.3.2.12) /Folylpolyglutamate synthase (EC6.3.2.17);Ontology_term=KEGG_ENZYME:6.3.2.12,KEGG_ENZYME:6.3.2.17 |
| YP_001467043.1| transcription-repair coupling | fig|6666666.462.peg.1005;Name=Transcription-repair coupling factor |
| YP_001467042.1| D-methionine ABC transporter, | fig|6666666.462.peg.1004;Name=Putative ATP/GTP-binding protein |
| YP_001467041.1| endonuclease III | fig|6666666.462.peg.1003;Name=Endonuclease III (EC4.2.99.18);Ontology_term=KEGG_ENZYME:4.2.99.18 |
| YP_001467040.1| cysteine synthase A | fig|6666666.462.peg.1002;Name=Cysteine synthase (EC2.5.1.47);Ontology_term=KEGG_ENZYME:2.5.1.47 |
| YP_001467039.1| serine O-acetyltransferase | fig|6666666.462.peg.1001;Name=Serine acetyltransferase (EC2.3.1.30);Ontology_term=KEGG_ENZYME:2.3.1.30 |
| YP_001467037.1| glutamate racemase 2 | fig|6666666.462.peg.998;Name=Cytochrome C553 (soluble cytochrome f) |
| YP_001467036.1| cell division [Campylobacter | fig|6666666.462.peg.997;Name=D-glycero-D-manno-heptose12C7-bisphosphate phosphatase (EC3.1.1.-);Ontology_term=KEGG_ENZYME:3.1.1.- |
| YP_001467035.1| ADP-glyceromanno-heptose | fig|6666666.462.peg.996;Name=ADP-L-glycero-D-manno-heptose-6-epimerase(EC 5.1.3.20);Ontology_term=KEGG_ENZYME:5.1.3.20 |
| YP_001467034.1| | fig|6666666.462.peg.995;Name=ADP-heptose synthase (EC 2.7.-.-) /D-glycero-beta-D-manno-heptose 7-phosphatekinase;Ontology_term=KEGG_ENZYME:2.7.-.- |
| YP_001467033.1| phosphoheptose isomerase | fig|6666666.462.peg.994;Name=Phosphoheptose isomerase 1 (EC5.3.1.-);Ontology_term=KEGG_ENZYME:5.3.1.- |
| YP_001467032.1| hypothetical protein | fig|6666666.462.peg.993;Name=hypothetical protein |
| YP_001467030.1| ATP/GTP hydrolase | fig|6666666.462.peg.991;Name=tRNA(Ile)-lysidine synthetase |
| YP_001467029.1| hypothetical protein | fig|6666666.462.peg.990;Name=Ribosomal protein S12p Asp88 (E. coli)methylthiotransferase |
| YP_001467028.1| pantoate--beta-alanine ligase | fig|6666666.462.peg.989;Name=Pantoate--beta-alanine ligase (EC6.3.2.1);Ontology_term=KEGG_ENZYME:6.3.2.1 |
| YP_001467027.1| peptide chain release factor 2 | fig|6666666.462.peg.988;Name=Peptide chain release factor 2 |
| YP_001467026.1| methyl-accepting chemotaxis | fig|6666666.462.peg.987;Name=hypothetical protein |
| YP_001467025.1| signal-transduction sensor | fig|6666666.462.peg.986;Name=hypothetical protein |
| YP_001467024.1| type II secretion system | fig|6666666.462.peg.985;Name=hypothetical protein |
| YP_001467023.1| type II secretion system | fig|6666666.462.peg.984;Name=Putative Dihydrolipoamide dehydrogenase(EC 1.8.1.4)%3B Mercuric ion reductase (EC 1.16.1.1)%3BPF00070 family2C FAD-dependent NAD(P)-disulphideoxidoreductase;Ontology_term=KEGG_ENZYME:1.8.1.4,KEGG_ENZYME:1.16.1.1 |
| YP_001467022.1| Sel1 repeat-containing protein | fig|6666666.462.peg.983;Name=hypothetical protein |
| YP_001467021.1| transcriptional regulatory | fig|6666666.462.peg.981;Name=two component transcriptional regulator2Cwinged helix family |
| YP_001467020.1| hypothetical protein | fig|6666666.462.peg.979;Name=membrane protein |
| YP_001467019.1| hypothetical protein | fig|6666666.462.peg.978;Name=Putative membrane protein |
| YP_001467018.1| hypothetical protein | fig|6666666.462.peg.977;Name=hypothetical protein |
| YP_001467017.1| hypothetical protein | fig|6666666.462.peg.976;Name=hypothetical protein |
| YP_001467016.1| GTP-binding protein | fig|6666666.462.peg.975;Name=Putative lipoprotein |
| YP_001467015.1| tRNA-I(6)A37 thiotransferase | fig|6666666.462.peg.974;Name=tRNA-i(6)A37 methylthiotransferase |
| YP_001467014.1| hypothetical protein | fig|6666666.462.peg.973;Name=hypothetical protein |
| YP_001467013.1| transcription elongation | fig|6666666.462.peg.972;Name=Transcription termination protein NusA |
| YP_001467012.1| argininosuccinate synthase | fig|6666666.462.peg.971;Name=acetyltransferase2C gnat family |
| YP_001467010.1| Ppx/GppA family phosphatase | fig|6666666.462.peg.969;Name=Putative H-T-H containing protein |
| YP_001467008.1| transcription termination | fig|6666666.462.peg.968;Name=Transcription termination factor Rho |
| YP_001467006.1| threonine synthase | fig|6666666.462.peg.967;Name=Threonine synthase (EC4.2.3.1);Ontology_term=KEGG_ENZYME:4.2.3.1 |
| YP_001467005.1| L-PSP family endoribonuclease | fig|6666666.462.peg.966;Name=hypothetical protein |
| YP_001467004.1| hypothetical protein | fig|6666666.462.peg.965;Name=YheO-like PAS domain |
| YP_001467003.1| heavy metal translocating | fig|6666666.462.peg.964;Name=Lead2C cadmium2C zinc and mercurytransporting ATPase (EC 3.6.3.3) (EC 3.6.3.5)%3BCopper-translocating P-type ATPase (EC3.6.3.4);Ontology_term=KEGG_ENZYME:3.6.3.3,KEGG_ENZYME:3.6.3.5,KEGG_ENZYME:3.6.3.4 |
| YP_001467001.1| DNA polymerase III subunits | fig|6666666.462.peg.961;Name=DNA polymerase III subunits gamma and tau(EC 2.7.7.7);Ontology_term=KEGG_ENZYME:2.7.7.7 |
| YP_001466999.1| glutamine synthetase, type I | fig|6666666.462.peg.959;Name=Glutamine synthetase type I (EC6.3.1.2);Ontology_term=KEGG_ENZYME:6.3.1.2 |
| YP_001466998.1| PHP domain-containing protein | fig|6666666.462.peg.958;Name=Histidinol-phosphatase (EC3.1.3.15);Ontology_term=KEGG_ENZYME:3.1.3.15 |
| YP_001466997.1| hypothetical protein | fig|6666666.462.peg.957;Name=hypothetical protein |
| YP_001466996.1| hypothetical protein | fig|6666666.462.peg.954;Name=hypothetical protein |
| YP_001466995.1| transcription activator | fig|6666666.462.peg.953;Name=hypothetical protein |
| YP_001466994.1| glycyl-tRNA synthetase subunit | fig|6666666.462.peg.952;Name=Glycyl-tRNA synthetase alpha chain (EC6.1.1.14);Ontology_term=KEGG_ENZYME:6.1.1.14 |
| YP_001466993.1| hypothetical protein | fig|6666666.462.peg.951;Name=FIG137478: Hypothetical protein |
| YP_001466992.1| hypothetical protein | fig|6666666.462.peg.950;Name=FIG008220: hypothetical protein |
| YP_001466991.1| | fig|6666666.462.peg.949;Name=3-deoxy-D-manno-octulosonic-acid transferase(EC 2.-.-.-);Ontology_term=KEGG_ENZYME:2.-.-.- |
| YP_001466990.1| RNA pseudouridine synthase | fig|6666666.462.peg.948;Name=FIG000124: Ribosomal large subunitpseudouridine synthase D (EC4.2.1.70);Ontology_term=KEGG_ENZYME:4.2.1.70 |
| YP_001466989.1| signal recognition particle | fig|6666666.462.peg.947;Name=Signal recognition particle2C subunit FfhSRP54 (TC 3.A.5.1.1) |
| YP_001466988.1| ribosomal protein S16 | fig|6666666.462.peg.946;Name=SSU ribosomal protein S16p |
| YP_001466987.1| hypothetical protein | fig|6666666.462.peg.945;Name=KH domain RNA binding protein YlqC |
| YP_001466986.1| 16S rRNA processing protein | fig|6666666.462.peg.944;Name=16S rRNA processing protein RimM |
| YP_001466985.1| tRNA | fig|6666666.462.peg.943;Name=tRNA (Guanine37-N1) -methyltransferase (EC2.1.1.31);Ontology_term=KEGG_ENZYME:2.1.1.31 |
| YP_001466984.1| 50S ribosomal protein L19 | fig|6666666.462.peg.942;Name=LSU ribosomal protein L19p |
| YP_001466983.1| hypothetical protein | fig|6666666.462.peg.941;Name=Late competence protein ComEA2C DNA receptor |
| YP_001466970.1| bifunctional | fig|6666666.462.peg.1487;Name=32C4-dihydroxy-2-butanone 4-phosphatesynthase / GTP cyclohydrolase II (EC3.5.4.25);Ontology_term=KEGG_ENZYME:3.5.4.25 |
| YP_001466969.1| type I | fig|6666666.462.peg.1489;Name=Type I restriction-modification system2CDNA-methyltransferase subunit M (EC2.1.1.72);Ontology_term=KEGG_ENZYME:2.1.1.72 |
| YP_001466967.1| type I restriction enzyme | fig|6666666.462.peg. 1493;Name=Type I restriction-modification system2C restriction subunit R (EC 3.1.21.3);Ontology_term=KEGG_ENZYME:3.1.21.3 |
| YP_001466966.1| | fig|6666666.462.peg.1494;Name=Methylated-DNA--protein-cysteinemethyltransferase (EC2.1.1.63);Ontology_term=KEGG_ENZYME:2.1.1.63 |
| YP_001466965.1| bifunctional aconitate | fig|6666666.462.peg.1496;Name=Aconitate hydratase 2 (EC4.2.1.3);Ontology_term=KEGG_ENZYME:4.2.1.3 |
| YP_001466964.1| hypothetical protein | fig|6666666.462.peg.1497;Name=Ankyrin repeat-containing possibleperiplasmic protein |
| YP_001466963.1| NAD-dependent | fig|6666666.462.peg.1498;Name=Oxidoreductase |
| YP_001466962.1| Na(+)/H(+) antiporter family | fig|6666666.462.peg.1499;Name=NhaC2C Na+/H+ antiporter |
| YP_001466961.1| tRNA | fig|6666666.462.peg.1500;Name=tRNA (uracil-5-)-methyltransferase (EC2.1.1.35);Ontology_term=KEGG_ENZYME:2.1.1.35 |
| YP_001466960.1| threonine dehydratase | fig|6666666.462.peg.1501;Name=Threonine dehydratase (EC4.3.1.19);Ontology_term=KEGG_ENZYME:4.3.1.19 |
| YP_001466959.1| glyoxalase II [Campylobacter | fig|6666666.462.peg.1502;Name=metallo-beta-lactamase domain protein |
| YP_001466958.1| hypothetical protein | fig|6666666.462.peg.1503;Name=membrane protein |
| YP_001466957.1| Dyp-type peroxidase | fig|6666666.462.peg.1504;Name=TyrA protein |
| YP_001466956.1| 4-hydroxybenzoate | fig|6666666.462.peg.1505;Name=hypothetical protein |
| YP_001466955.1| putative cytochrome C-type | fig|6666666.462.peg.1506;Name=putative cytochrome C-type haem-bindingperiplasmic protein |
| YP_001466952.1| radical SAM domain-containing | fig|6666666.462.peg.1507;Name=Biotin sulfoxide reductase (EC1.-.-.-);Ontology_term=KEGG_ENZYME:1.-.-.- |
| YP_001466949.1| ModD protein [Campylobacter | fig|6666666.462.peg.1125;Name=Quinolinate phosphoribosyltransferase[decarboxylating] (EC2.4.2.19);Ontology_term=KEGG_ENZYME:2.4.2.19 |
| YP_001466948.1| hypothetical protein | fig|6666666.462.peg.1124;Name=Helicase2C C-terminal |
| YP_001466947.1| hypothetical protein | fig|6666666.462.peg.1123;Name=Putative amino-acid transport protein |
| YP_001466946.1| tetrapyrrole methylase family | fig|6666666.462.peg.1122;Name=METAL TRANSPORTING ATPASE MTA72 |
| YP_001466945.1| RNA methyltransferase | fig|6666666.462.peg.1121;Name=hypothetical protein |
| YP_001466944.1| hypothetical protein | fig|6666666.462.peg.1120;Name=hypothetical protein |
| YP_001466943.1| hypothetical protein | fig|6666666.462.peg.1119;Name=hypothetical protein |
| YP_001466942.1| aminotransferase | fig|6666666.462.peg.1118;Name=hypothetical protein |
| YP_001466941.1| hypothetical protein | fig|6666666.462.peg.1117;Name=hypothetical protein |
| YP_001466940.1| FUR family transcriptional | fig|6666666.462.peg.1115;Name=Ferric uptake regulation protein FUR |
| YP_001466939.1| hypothetical protein | fig|6666666.462.peg.1114;Name=hypothetical protein |
| YP_001466938.1| hypothetical protein | fig|6666666.462.peg.1113;Name=hypothetical protein |
| YP_001466937.1| thioredoxin reductase (trxr) | fig|6666666.462.peg.1112;Name=putative membrane protein |
| YP_001466936.1| dihydrodipicolinate reductase | fig|6666666.462.peg.1111;Name=COG1565: Uncharacterized conserved protein |
| YP_001466935.1| YbaK/ebsC protein | fig|6666666.462.peg.1110;Name=Transcriptional regulator |
| YP_001466934.1| flagellar basal | fig|6666666.462.peg.1109;Name=Flagellar biosynthesis protein FliL |
| YP_001466933.1| holo-(acyl-carrier-protein) | fig|6666666.462.peg.1108;Name=Holo-[acyl-carrier protein] synthase (EC2.7.8.7);Ontology_term=KEGG_ENZYME:2.7.8.7 |
| YP_001466928.1| DNA repair protein RadA | fig|6666666.462.peg.1403;Name=DNA repair protein RadA |
| YP_001466927.1| TPR repeat-containing protein | fig|6666666.462.peg.1402;Name=hypothetical protein |
| YP_001466926.1| signal recognition | fig|6666666.462.peg.1401;Name=Signal recognition particle receptor proteinFtsY (alpha subunit) (TC 3.A.5.1.1) |
| YP_001466925.1| transporter [Campylobacter | fig|6666666.462.peg.1400;Name=Putative lipoprotein thiredoxin |
| YP_001466924.1| 5,10-methenyltetrahydrofolate | fig|6666666.462.peg.1399;Name=5-formyltetrahydrofolate cyclo-ligase (EC6.3.3.2);Ontology_term=KEGG_ENZYME:6.3.3.2 |
| YP_001466923.1| phosphodiesterase | fig|6666666.462.peg.1398;Name=Hydrolase (HAD superfamily) |
| YP_001466922.1| GTP-binding protein | fig|6666666.462.peg.1396;Name=membrane protein |
| YP_001466921.1| hypothetical protein | fig|6666666.462.peg.1395;Name=Putative periplasmic protein |
| YP_001466920.1| flagellar basal-body rod | fig|6666666.462.peg.1089;Name=hypothetical protein |
| YP_001466918.1| hypothetical protein | fig|6666666.462.peg.1086;Name=FIG003955: protein of unknown function |
| YP_001466917.1| RIP metalloprotease RseP | fig|6666666.462.peg.1085;Name=Membrane-associated zinc metalloprotease |
| YP_001466915.1| | fig|6666666.462.peg.1083;Name=CDP-diacylglycerol--glycerol-3-phosphate3-phosphatidyltransferase (EC2.7.8.5);Ontology_term=KEGG_ENZYME:2.7.8.5 |
| YP_001466914.1| 7-alpha-hydroxysteroid | fig|6666666.462.peg.1082;Name=3-oxoacyl-[acyl-carrier protein] reductase(EC 1.1.1.100);Ontology_term=KEGG_ENZYME:1.1.1.100 |
| YP_001466913.1| dihydrodipicolinate synthase | fig|6666666.462.peg.1081;Name=Dihydrodipicolinate synthase (EC4.2.1.52);Ontology_term=KEGG_ENZYME:4.2.1.52 |
| YP_001466912.1| cytochrome c551 peroxidase | fig|6666666.462.peg.1080;Name=Putative zinc protease |
| YP_001466911.1| dihydroorotate dehydrogenase 2 | fig|6666666.462.peg.1079;Name=Dihydroorotate dehydrogenase (EC1.3.3.1);Ontology_term=KEGG_ENZYME:1.3.3.1 |
| YP_001466910.1| lipid A export | fig|6666666.462.peg.1078;Name=Phospholipid-lipopolysaccharide ABCtransporter |
| YP_001466909.1| cysteinyl-tRNA synthetase | fig|6666666.462.peg.1077;Name=Cysteinyl-tRNA synthetase (EC6.1.1.16);Ontology_term=KEGG_ENZYME:6.1.1.16 |
| YP_001466908.1| holliday junction DNA helicase | fig|6666666.462.peg.1074;Name=Holliday junction DNA helicase RuvA |
| YP_001466907.1| hypothetical protein | fig|6666666.462.peg.1072;Name=hypothetical protein |
| YP_001466906.1| Mur ligase middle | fig|6666666.462.peg.1070;Name=UDP-N-acetylmuramoylalanyl-D-glutamyl-22C6-diaminopimelate--D-alanyl-D-alanine ligase (EC6.3.2.10);Ontology_term=KEGG_ENZYME:6.3.2.10 |
| YP_001466905.1| hypothetical protein | fig|6666666.462.peg.1069;Name=hypothetical protein |
| YP_001466903.1| hydrogenase | fig|6666666.462.peg.598;Name=Integrase-recombinase protein XERCD family |
| YP_001466902.1| acetate kinase [Campylobacter | fig|6666666.462.peg.597;Name=Acetate kinase (EC2.7.2.1);Ontology_term=KEGG_ENZYME:2.7.2.1 |
| YP_001466901.1| hydrogenase | fig|6666666.462.peg.596;Name=Phosphate acetyltransferase (EC2.3.1.8);Ontology_term=KEGG_ENZYME:2.3.1.8 |
| YP_001466900.1| flagellar basal body L-ring | fig|6666666.462.peg.595;Name=Flagellar L-ring protein FlgH |
| YP_001466899.1| putative periplasmic protein | fig|6666666.462.peg.594;Name=Quinone-reactive Ni/Fe-hydrogenase smallchain precursor (EC1.12.5.1);Ontology_term=KEGG_ENZYME:1.12.5.1 |
| YP_001466898.1| aminodeoxychorismate lyase | fig|6666666.462.peg.593;Name=Quinone-reactive Ni/Fe-hydrogenase largechain (EC 1.12.5.1);Ontology_term=KEGG_ENZYME:1.12.5.1 |
| YP_001466897.1| hydrogenase maturation | fig|6666666.462.peg.591;Name=Hydrogenase maturation protease (EC3.4.24.-);Ontology_term=KEGG_ENZYME:3.4.24.- |
| YP_001466896.1| hypothetical protein | fig|6666666.462.peg.590;Name=hypothetical protein |
| YP_001466895.1| [NiFe] hydrogenase maturation | fig|6666666.462.peg.589;Name=[NiFe] hydrogenase metallocenter assemblyprotein HypF |
| YP_001466894.1| nickel responsive regulator | fig|6666666.462.peg.588;Name=Nickel responsive regulator NikR |
| YP_001466892.1| hypothetical protein | fig|6666666.462.peg.586;Name=hypothetical protein |
| YP_001466891.1| hypothetical protein | fig|6666666.462.peg.585;Name=hypothetical protein |
| YP_001466890.1| hydrogenase accessory protein | fig|6666666.462.peg.584;Name=[NiFe] hydrogenase nickelincorporation-associated protein HypB |
| YP_001466889.1| hydrogenase assembly chaperone | fig|6666666.462.peg.583;Name=[NiFe] hydrogenase metallocenterassembly protein HypC |
| YP_001466888.1| hydrogenase | fig|6666666.462.peg.582;Name=[NiFe] hydrogenase metallocenter assemblyprotein HypD |
| YP_001466887.1| hydrogenase | fig|6666666.462.peg.581;Name=[NiFe] hydrogenase metallocenter assemblyprotein HypE |
| YP_001466886.1| hydrogenase nickel insertion | fig|6666666.462.peg.580;Name=[NiFe] hydrogenase nickel incorporationprotein HypA |
| YP_001466884.1| hypothetical protein | fig|6666666.462.peg.579;Name=Putative periplasmic protein |
| YP_001466883.1| hypothetical protein | fig|6666666.462.peg.578;Name=FIG004453: protein YceG like |
| YP_001466882.1| monomeric isocitrate | fig|6666666.462.peg.577;Name=Isocitrate dehydrogenase [NADP] (EC1.1.1.42)%3B Monomeric isocitrate dehydrogenase [NADP](EC1.1.1.42);Ontology_term=KEGG_ENZYME:1.1.1.42,KEGG_ENZYME:1.1.1.42 |
| YP_001466881.1| malate dehydrogenase | fig|6666666.462.peg.576;Name=Malate dehydrogenase (EC1.1.1.37);Ontology_term=KEGG_ENZYME:1.1.1.37 |
| YP_001466880.1| 2-oxoglutarate-acceptor | fig|6666666.462.peg.575;Name=2-oxoglutarate oxidoreductase2C deltasubunit2C putative (EC1.2.7.3);Ontology_term=KEGG_ENZYME:1.2.7.3 |
| YP_001466879.1| 2-oxoglutarate-acceptor | fig|6666666.462.peg.574;Name=2-oxoglutarate oxidoreductase2C alpha subunit(EC 1.2.7.3);Ontology_term=KEGG_ENZYME:1.2.7.3 |
| YP_001466878.1| 2-oxoglutarate-acceptor | fig|6666666.462.peg.573;Name=2-oxoglutarate oxidoreductase2C beta subunit(EC 1.2.7.3);Ontology_term=KEGG_ENZYME:1.2.7.3 |
| YP_001466877.1| 2-oxoglutarate-acceptor | fig|6666666.462.peg.572;Name=2-oxoglutarate oxidoreductase2C gamma subunit(EC 1.2.7.3);Ontology_term=KEGG_ENZYME:1.2.7.3 |
| YP_001466876.1| putative heat shock protein | fig|6666666.462.peg.571;Name=hypothetical protein |
| YP_001466869.1| succinate dehydrogenase | fig|6666666.462.peg.569;Name=Succinate dehydrogenase flavoprotein subunit(EC 1.3.99.1);Ontology_term=KEGG_ENZYME:1.3.99.1 |
| YP_001466868.1| O-acetylhomoserine | fig|6666666.462.peg.568;Name=Succinate dehydrogenase iron-sulfur protein(EC 1.3.99.1);Ontology_term=KEGG_ENZYME:1.3.99.1 |
| YP_001466867.1| hypothetical protein | fig|6666666.462.peg.567;Name=Putative succinate dehydrogenase cytochrome bsubunit |
| YP_001466865.1| thiamine monophosphate | fig|6666666.462.peg.552;Name=Thiamin-phosphate pyrophosphorylase (EC2.5.1.3);Ontology_term=KEGG_ENZYME:2.5.1.3 |
| YP_001466864.1| thiamine biosynthesis protein | fig|6666666.462.peg.551;Name=Thiazole biosynthesis protein ThiH |
| YP_001466863.1| thiamine biosynthesis protein | fig|6666666.462.peg.549;Name=Sulfur carrier protein adenylyltransferaseThiF |
| YP_001466862.1| thiamine biosynthesis protein | fig|6666666.462.peg.548;Name=Sulfur carrier protein ThiS |
| YP_001466861.1| aspartate aminotransferase | fig|6666666.462.peg.547;Name=Aspartate aminotransferase (EC2.6.1.1);Ontology_term=KEGG_ENZYME:2.6.1.1 |
| YP_001466860.1| arginine decarboxylase | fig|6666666.462.peg.546;Name=Arginine decarboxylase (EC4.1.1.19);Ontology_term=KEGG_ENZYME:4.1.1.19 |
| YP_001466859.1| histidyl-tRNA synthetase | fig|6666666.462.peg.545;Name=Histidyl-tRNA synthetase (EC6.1.1.21);Ontology_term=KEGG_ENZYME:6.1.1.21 |
| YP_001466858.1| thymidylate kinase | fig|6666666.462.peg.544;Name=Thymidylate kinase (EC2.7.4.9);Ontology_term=KEGG_ENZYME:2.7.4.9 |
| YP_001466857.1| phosphopantetheine | fig|6666666.462.peg.543;Name=Phosphopantetheine adenylyltransferase (EC2.7.7.3);Ontology_term=KEGG_ENZYME:2.7.7.3 |
| YP_001466856.1| 3-octaprenyl-4-hydroxybenzoate | fig|6666666.462.peg.542;Name=3-polyprenyl-4-hydroxybenzoate carboxy-lyaseUbiX (EC 4.1.1.-);Ontology_term=KEGG_ENZYME:4.1.1.- |
| YP_001466854.1| MoeA domain-containing protein | fig|6666666.462.peg.1317;Name=Molybdopterin biosynthesis MoeA protein |
| YP_001466853.1| UDP-N-acetylglucosamine | fig|6666666.462.peg.1316;Name=UDP-N-acetylglucosamine1-carboxyvinyltransferase (EC2.5.1.7);Ontology_term=KEGG_ENZYME:2.5.1.7 |
| YP_001466852.1| hypothetical protein | fig|6666666.462.peg.1315;Name=hypothetical protein |
| YP_001466851.1| peptidase E [Campylobacter | fig|6666666.462.peg.1313;Name=Putative integral membrane protein |
| YP_001466850.1| hypothetical protein | fig|6666666.462.peg.1312;Name=COG2833: uncharacterized protein |
| YP_001466849.1| phosphoglycerate mutase family | fig|6666666.462.peg.1311;Name=phosphohistidine phosphatase SixA2C putative |
| YP_001466846.1| putative flagellar | fig|6666666.462.peg.1308;Name=Outer membrane efflux protein |
| YP_001466845.1| macrolide export | fig|6666666.462.peg.1307;Name=Macrolide export ATP-binding/permease proteinMacB (EC 3.6.3.-);Ontology_term=KEGG_ENZYME:3.6.3.- |
| YP_001466844.1| membrane fusion protein (MFP) | fig|6666666.462.peg.1306;Name=Macrolide-specific efflux protein MacA |
| YP_001466843.1| putative site-specific DNA | fig|6666666.462.peg.364;Name=ATP-dependent DNA helicase UvrD/PcrA/Rep2Cepsilon proteobacterial type 1 |
| YP_001466842.1| flagellar P-ring protein | fig|6666666.462.peg.365;Name=Penicillin-insensitive transglycosylase (EC2.4.2.-) & transpeptidasePBP-1C;Ontology_term=KEGG_ENZYME:2.4.2.- |
| YP_001466841.1| alpha-2-macroglobulin family | fig|6666666.462.peg. 366;Name=Alpha-2-macroglobulin |
| YP_001466839.1| valyl-tRNA synthetase | fig|6666666.462.peg.367;Name=Valyl-tRNA synthetase (EC6.1.1.9);Ontology_term=KEGG_ENZYME:6.1.1.9 |
| YP_001466837.1| methionine import ATP-binding | fig|6666666.462.peg.368;Name=Methionine ABC transporter ATP-bindingprotein |
| YP_001466836.1| putative ABC transporter, | fig|6666666.462.peg.369;Name=Methionine ABC transporter permease protein |
| YP_001466835.1| nlpa lipoprotein | fig|6666666.462.peg.371;Name=Methionine ABC transportersubstrate-binding protein |
| YP_001466834.1| glutamate racemase | fig|6666666.462.peg.373;Name=Glutamate racemase (EC5.1.1.3);Ontology_term=KEGG_ENZYME:5.1.1.3 |
| YP_001466833.1| cell cycle protein FtsW | fig|6666666.462.peg.374;Name=Cell division protein FtsW |
| YP_001466832.1| N-acetylglucosaminyl | fig|6666666.462.peg.375;Name=UDP-N-acetylglucosamine--N-acetylmuramyl-(pentapeptide) pyrophosphoryl-undecaprenolN-acetylglucosamine transferase (EC2.4.1.227);Ontology_term=KEGG_ENZYME:2.4.1.227 |
| YP_001466831.1| hypothetical protein | fig|6666666.462.peg.376;Name=hypothetical protein |
| YP_001466830.1| hypothetical protein | fig|6666666.462.peg.377;Name=hypothetical protein |
| YP_001466829.1| RNA polymerase factor sigma-54 | fig|6666666.462.peg.378;Name=RNA polymerase sigma-54 factor RpoN |
| YP_001466828.1| ABC-transporter ATP-binding | fig|6666666.462.peg.379;Name=ABC-transporter ATP-binding protein |
| YP_001466827.1| hypothetical protein | fig|6666666.462.peg.380;Name=ATPase YjeE2C predicted to have essentialrole in cell wall biosynthesis |
| YP_001466826.1| uroporphyrinogen decarboxylase | fig|6666666.462.peg.381;Name=Ribosome-associated heat shock proteinimplicated in the recycling of the 50S subunit |
| YP_001466825.1| methyl-accepting chemotaxis | fig|6666666.462.peg.398;Name=Signal transduction protein CetA2C mediatesan energy taxis response |
| YP_001466824.1| methyl-accepting chemotaxis | fig|6666666.462.peg.397;Name=Signal transduction protein CetB2C mediatesan energy taxis response |
| YP_001466823.1| transcription regulator AsnC | fig|6666666.462.peg.396;Name=Type II secretion system protein |
| YP_001466822.1| L(+)-tartrate dehydratase | fig|6666666.462.peg.395;Name=Type II secretion cytoplasmic ATP bindingprotein (PulE2C ATPase) |
| YP_001466821.1| hypothetical protein | fig|6666666.462.peg.394;Name=hypothetical protein |
| YP_001466820.1| hypothetical protein | fig|6666666.462.peg.393;Name=Transformation system protein |
| YP_001466819.1| phosphoribosylaminoimidazole | fig|6666666.462.peg.392;Name=MSHA biogenesis protein MshM |
| YP_001466818.1| PP family ATPase | fig|6666666.462.peg.391;Name=Type II secretion outermembrane poreforming protein (PulD) |
| YP_001466817.1| hypothetical protein | fig|6666666.462.peg.390;Name=hypothetical protein |
| YP_001466816.1| hypothetical protein | fig|6666666.462.peg.389;Name=hypothetical protein |
| YP_001466814.1| GTP-binding protein Era | fig|6666666.462.peg.387;Name=GTP-binding protein Era |
| YP_001466813.1| ATP-dependent protease | fig|6666666.462.peg.386;Name=ATP-dependent hsl protease ATP-bindingsubunit HslU |
| YP_001466812.1| ATP-dependent protease | fig|6666666.462.peg.385;Name=ATP-dependent protease HslV (EC3.4.25.-);Ontology_term=KEGG_ENZYME:3.4.25.- |
| YP_001466811.1| ribosomal protein L9 | fig|6666666.462.peg.384;Name=LSU ribosomal protein L9p |
| YP_001466810.1| argininosuccinate synthase | fig|6666666.462.peg.383;Name=Argininosuccinate synthase (EC6.3.4.5);Ontology_term=KEGG_ENZYME:6.3.4.5 |
| YP_001466809.1| phosphate transporter family | fig|6666666.462.peg.401;Name=Probable low-affinity inorganic phosphatetransporter |
| YP_001466808.1| WalN protein [Campylobacter | fig|6666666.462.peg.400;Name=Possible phosphatase |
| YP_001466807.1| excinuclease ABC subunit A | fig|6666666.462.peg.399;Name=Transamidase GatB domain protein |
| YP_001466792.1| hypothetical protein | fig|6666666.462.peg.1096;Name=conserved hypothetical protein |
| YP_001466791.1| hypothetical protein | fig|6666666.462.peg.1094;Name=hypothetical protein |
| YP_001466790.1| FtsK/SpoIIIE family protein | fig|6666666.462.peg.1093;Name=Cell division protein FtsK |
| YP_001466788.1| DNA-binding protein HU 1 | fig|6666666.462.peg.1090;Name=DNA-binding protein HU-beta |
| YP_001466784.1| response regulator | fig|6666666.462.peg.1087;Name=hypothetical protein |
| YP_001466783.1| auxin efflux carrier (AEC) | fig|6666666.462.peg.1088;Name=hypothetical protein |
| YP_001466781.1| extracellular solute-binding | fig|6666666.462.peg.402;Name=Two-component system response regulator DccR |
| YP_001466780.1| hypothetical protein | fig|6666666.462.peg.403;Name=Two-component system histidine kinase DccS |
| YP_001466779.1| elongation factor P (EF-P) | fig|6666666.462.peg.1408;Name=Aldehyde dehydrogenase B (EC1.2.1.22);Ontology_term=KEGG_ENZYME:1.2.1.22 |
| YP_001466778.1| Ser/Thr protein phosphatase | fig|6666666.462.peg.1409;Name=serine/threonine protein phosphatase |
| YP_001466777.1| hypothetical protein | fig|6666666.462.peg.1410;Name=hypothetical protein |
| YP_001466776.1| putative molybdopterin | fig|6666666.462.peg.1411;Name=Ribonuclease BN (EC3.1.-.-);Ontology_term=KEGG_ENZYME:3.1.-.- |
| YP_001466775.1| hypothetical protein | fig|6666666.462.peg.1412;Name=hypothetical protein |
| YP_001466774.1| M23 peptidase | fig|6666666.462.peg.1413;Name=Membrane proteins related tometalloendopeptidases |
| YP_001466773.1| magnesium transporter | fig|6666666.462.peg.1414;Name=Magnesium transporter |
| YP_001466772.1| NUDIX family hydrolase | fig|6666666.462.peg.1415;Name=Uridine diphosphate glucose pyrophosphatase(EC 3.6.1.45);Ontology_term=KEGG_ENZYME:3.6.1.45 |
| YP_001466771.1| polyphosphate kinase | fig|6666666.462.peg.1416;Name=Polyphosphate kinase (EC2.7.4.1);Ontology_term=KEGG_ENZYME:2.7.4.1 |
| YP_001466770.1| putative endonuclease | fig|6666666.462.peg.1417;Name=hypothetical protein |
| YP_001466769.1| hypothetical protein | fig|6666666.462.peg.1418;Name=probable membrane protein STY4869 |
| YP_001466768.1| carboxypeptidase G2 | fig|6666666.462.peg.1419;Name=Acetylornithinedeacetylase/Succinyl-diaminopimelate desuccinylase andrelated deacylases |
| YP_001466767.1| hypothetical protein | fig|6666666.462.peg.251;Name=3-polyprenyl-4-hydroxybenzoate carboxy-lyase(EC 4.1.1.-);Ontology_term=KEGG_ENZYME:4.1.1.- |
| YP_001466766.1| hypothetical protein | fig|6666666.462.peg.1421;Name=3-polyprenyl-4-hydroxybenzoatecarboxy-lyase (EC4.1.1.-);Ontology_term=KEGG_ENZYME:4.1.1.- |
| YP_001466765.1| DNA polymerase III subunit | fig|6666666.462.peg.1423;Name=Exonuclease2C possibly dna polymerase IIIepsilon subunit (EC2.7.7.7);Ontology_term=KEGG_ENZYME:2.7.7.7 |
| YP_001466762.1| 50S ribosomal protein L28 | fig|6666666.462.peg.1099;Name=LSU ribosomal protein L28p |
| YP_001466761.1| TrkA domain-containing protein | fig|6666666.462.peg.1098;Name=Potassium channel protein |
| YP_001466760.1| hypothetical protein | fig|6666666.462.peg.1097;Name=hypothetical protein |
| YP_001466759.1| heat shock protein 90 | fig|6666666.462.peg.566;Name=Chaperone protein HtpG |
| YP_001466758.1| nitrogen regulation protein | fig|6666666.462.peg.553;Name=hypothetical protein |
| YP_001466757.1| molybdenum cofactor synthesis | fig|6666666.462.peg.554;Name=Molybdopterin biosynthesis Mog protein2Cmolybdochelatase |
| YP_001466756.1| M41 family peptidase | fig|6666666.462.peg.555;Name=Cell division protein FtsH (EC3.4.24.-);Ontology_term=KEGG_ENZYME:3.4.24.- |
| YP_001466755.1| hypothetical protein | fig|6666666.462.peg.556;Name=MiaB family protein2C possibly involved intRNA or rRNA modification |
| YP_001466754.1| small conductance | fig|6666666.462.peg.557;Name=Putative mechanosensitive ion channel |
| YP_001466753.1| 3-dehydroquinate synthase | fig|6666666.462.peg.558;Name=3-dehydroquinate synthase (EC4.2.3.4);Ontology_term=KEGG_ENZYME:4.2.3.4 |
| YP_001466752.1| TrkA domain-containing protein | fig|6666666.462.peg.559;Name=TrkA domain protein |
| YP_001466751.1| hypothetical protein | fig|6666666.462.peg.560;Name=hypothetical protein |
| YP_001466750.1| mce related protein | fig|6666666.462.peg.561;Name=Possible ABC transport system periplasmicsubstrate-binding protein |
| YP_001466749.1| PP-loop family protein | fig|6666666.462.peg.562;Name=Methionine ABC transporter ATP-bindingprotein |
| YP_001466748.1| MTA/SAH nucleosidase | fig|6666666.462.peg.563;Name=Putative ABC transport system permeaseprotein |
| YP_001466747.1| queuine | fig|6666666.462.peg.564;Name=tRNA-guanine transglycosylase (EC2.4.2.29);Ontology_term=KEGG_ENZYME:2.4.2.29 |
| YP_001466746.1| fkbp-type peptidyl-prolyl | fig|6666666.462.peg.565;Name=probable membrane protein Cj1011 |
| YP_001466745.1| chaperone ClpB 1 | fig|6666666.462.peg.933;Name=ClpB protein |
| YP_001466744.1| peptidoglycan associated | fig|6666666.462.peg.934;Name=Putative carboxyl-terminal protease (EC3.4.21.-);Ontology_term=KEGG_ENZYME:3.4.21.- |
| YP_001466743.1| | fig|6666666.462.peg.935;Name=Phosphoribosylaminoimidazole-succinocarboxamide synthase (EC6.3.2.6);Ontology_term=KEGG_ENZYME:6.3.2.6 |
| YP_001466742.1| | fig|6666666.462.peg.936;Name=Phosphoribosylformylglycinamidinesynthase2C PurS subunit (EC6.3.5.3);Ontology_term=KEGG_ENZYME:6.3.5.3 |
| YP_001466741.1| | fig|6666666.462.peg.937;Name=Phosphoribosylformylglycinamidine synthase2Cglutamine amidotransferase subunit (EC6.3.5.3);Ontology_term=KEGG_ENZYME:6.3.5.3 |
| YP_001466740.1| putative periplasmic protein | fig|6666666.462.peg.938;Name=Putative periplasmic protein |
| YP_001466739.1| ATP synthase F1, subunit | fig|6666666.462.peg.939;Name=1-acyl-sn-glycerol-3-phosphate acyltransferase(EC 2.3.1.51);Ontology_term=KEGG_ENZYME:2.3.1.51 |
| YP_001466737.1| UDP-3-O-[3-hydroxymyristoyl] | fig|6666666.462.peg.21;Name=UDP-3-O-[3-hydroxymyristoyl] glucosamineN-acyltransferase (EC2.3.1.-);Ontology_term=KEGG_ENZYME:2.3.1.- |
| YP_001466736.1| acetolactate synthase 3 | fig|6666666.462.peg.20;Name=Acetolactate synthase small subunit (EC2.2.1.6);Ontology_term=KEGG_ENZYME:2.2.1.6 |
| YP_001466735.1| acetolactate synthase 3 | fig|6666666.462.peg.19;Name=Acetolactate synthase large subunit (EC2.2.1.6);Ontology_term=KEGG_ENZYME:2.2.1.6 |
| YP_001466733.1| tRNA 2-selenouridine synthase | fig|6666666.462.peg.1171;Name=Selenophosphate-dependent tRNA2-selenouridine synthase |
| YP_001466732.1| histidine triad | fig|6666666.462.peg.1172;Name=HIT family protein |
| YP_001466731.1| TPR repeat-containing protein | fig|6666666.462.peg.1174;Name=Putative lipoprotein |
| YP_001466730.1| hypothetical protein | fig|6666666.462.peg.1175;Name=hypothetical protein |
| YP_001466729.1| methyltransferase small | fig|6666666.462.peg.1176;Name=COG4123: Predicted O-methyltransferase |
| YP_001466728.1| hypothetical protein | fig|6666666.462.peg.1177;Name=Cell division initiation protein DivIVA |
| YP_001466727.1| pyridine nucleotide-disulphide | fig|6666666.462.peg.1178;Name=Thioredoxin reductase (EC1.8.1.9);Ontology_term=KEGG_ENZYME:1.8.1.9 |
| YP_001466726.1| 3-deoxy-7-phosphoheptulonate | fig|6666666.462.peg.1179;Name=2-keto-3-deoxy-D-arabino-heptulosonate-7-phosphate synthase II (EC2.5.1.54);Ontology_term=KEGG_ENZYME:2.5.1.54 |
| YP_001466725.1| RarD protein [Campylobacter | fig|6666666.462.peg.1180;Name=Protein rarD |
| YP_001466723.1| arylsulfate sulfotransferase | fig|6666666.462.peg.1182;Name=membrane protein |
| YP_001466722.1| glycoside hydrolase family 3 | fig|6666666.462.peg.1183;Name=Beta-hexosaminidase (EC3.2.1.52);Ontology_term=KEGG_ENZYME:3.2.1.52 |
| YP_001466720.1| F0F1 ATP synthase subunit A | fig|6666666.462.peg.1186;Name=ATP synthase A chain (EC3.6.3.14);Ontology_term=KEGG_ENZYME:3.6.3.14 |
| YP_001466719.1| hypothetical protein | fig|6666666.462.peg.1187;Name=Putative membrane protein YfcA |
| YP_001466718.1| copper/zinc superoxide | fig|6666666.462.peg.1188;Name=Superoxide dismutase [Cu-Zn] precursor (EC1.15.1.1);Ontology_term=KEGG_ENZYME:1.15.1.1 |
| YP_001466717.1| hypothetical protein | fig|6666666.462.peg.1189;Name=hypothetical protein |
| YP_001466716.1| flagellar hook-associated | fig|6666666.462.peg.1190;Name=Flagellar hook-associated protein FlgK |
| YP_001466715.1| hypothetical protein | fig|6666666.462.peg.1191;Name=hypothetical protein |
| YP_001466714.1| hypothetical protein | fig|6666666.462.peg.1192;Name=hypothetical protein |
| YP_001466713.1| hypothetical protein | fig|6666666.462.peg.1193;Name=hypothetical protein |
| YP_001466712.1| flagellar basal body P-ring | fig|6666666.462.peg.1194;Name=Flagellar P-ring protein FlgI |
| YP_001466711.1| putative methyltransferase | fig|6666666.462.peg.1195;Name=Site-specific DNA methyltransferase (EC2.1.1.-);Ontology_term=KEGG_ENZYME:2.1.1.- |
| YP_001466710.1| ornithine carbamoyltransferase | fig|6666666.462.peg.1196;Name=JHP0747 family |
| YP_001466709.1| flagellar protein FlaG | fig|6666666.462.peg.1197;Name=Possible flagellar protein |
| YP_001466708.1| flagellar capping protein | fig|6666666.462.peg.1198;Name=Flagellar hook-associated protein FliD |
| YP_001466707.1| flagellar protein FliS | fig|6666666.462.peg.1199;Name=Flagellar biosynthesis protein FliS |
| YP_001466706.1| hypothetical protein | fig|6666666.462.peg.1200;Name=hypothetical protein |
| YP_001466702.1| tRNA pseudouridine synthase D | fig|6666666.462.peg.1766;Name=tRNA pseudouridine 13 synthase (EC4.2.1.-);Ontology_term=KEGG_ENZYME:4.2.1.- |
| YP_001466701.1| thiamine monophosphate kinase | fig|6666666.462.peg.1767;Name=Thiamine-monophosphate kinase (EC2.7.4.16);Ontology_term=KEGG_ENZYME:2.7.4.16 |
| YP_001466700.1| hypothetical protein | fig|6666666.462.peg.1768;Name=hypothetical protein |
| YP_001466698.1| putative periplasmic protein | fig|6666666.462.peg.251;Name=3-polyprenyl-4-hydroxybenzoate carboxy-lyase(EC 4.1.1.-);Ontology_term=KEGG_ENZYME:4.1.1.- |
| YP_001466695.1| porphobilinogen deaminase | fig|6666666.462.peg.253;Name=Porphobilinogen deaminase (EC2.5.1.61);Ontology_term=KEGG_ENZYME:2.5.1.61 |
| YP_001466694.1| hypothetical protein | fig|6666666.462.peg.254;Name=Putative integral memnbrane protein |
| YP_001466693.1| prolyl-tRNA synthetase | fig|6666666.462.peg.255;Name=Prolyl-tRNA synthetase (EC6.1.1.15);Ontology_term=KEGG_ENZYME:6.1.1.15 |
| YP_001466692.1| glutamyl-tRNA reductase | fig|6666666.462.peg.256;Name=Glutamyl-tRNA reductase (EC1.2.1.70);Ontology_term=KEGG_ENZYME:1.2.1.70 |
| YP_001466691.1| hypothetical protein | fig|6666666.462.peg.258;Name=hypothetical protein |
| YP_001466690.1| hypothetical protein | fig|6666666.462.peg.259;Name=hypothetical protein |
| YP_001466689.1| putative periplasmic protein | fig|6666666.462.peg.260;Name=Predicted phosphatase |
| YP_001466688.1| hypothetical protein | fig|6666666.462.peg.261;Name=hypothetical protein |
| YP_001466687.1| methyl-accepting chemotaxis | fig|6666666.462.peg.262;Name=O-acetylhomoserine sulfhydrylase (EC2.5.1.49) / O-succinylhomoserine sulfhydrylase (EC2.5.1.48);Ontology_term=KEGG_ENZYME:2.5.1.49,KEGG_ENZYME:2.5.1.48 |
| YP_001466684.1| hypothetical protein | fig|6666666.462.peg.265;Name=hypothetical protein |
| YP_001466683.1| sensor histidine kinase | fig|6666666.462.peg.266;Name=Putative two-component sensor |
| YP_001466682.1| response regulator receiver | fig|6666666.462.peg.267;Name=Putative two-component regulator |
| YP_001466681.1| protease DO [Campylobacter | fig|6666666.462.peg.268;Name=HtrA protease/chaperone protein / Serineprotease (Protease DO) (EC3.4.21.-);Ontology_term=KEGG_ENZYME:3.4.21.- |
| YP_001466680.1| DnaJ domain-containing protein | fig|6666666.462.peg.269;Name=DnaJ-class molecular chaperone CbpA |
| YP_001466679.1| cation-efflux system membrane | fig|6666666.462.peg.270;Name=HspR2C transcriptional repressor of DnaKoperon |
| YP_001466678.1| monovalent cation:proton | fig|6666666.462.peg.271;Name=Glutathione-regulated potassium-effluxsystem protein KefB |
| YP_001466675.1| Rrf2 family protein | fig|6666666.462.peg.272;Name=Lipoprotein releasing system transmembraneprotein LolC |
| YP_001466674.1| preprotein translocase subunit | fig|6666666.462.peg.273;Name=Protein export cytoplasm protein SecAATPase RNA helicase (TC 3.A.5.1.1) |
| YP_001466673.1| outer-membrane lipoprotein | fig|6666666.462.peg.274;Name=Outer-membrane lipoprotein carrier proteinprecursor |
| YP_001466672.1| putative periplasmic protein | fig|6666666.462.peg.275;Name=Putative periplasmic protein |
| YP_001466671.1| hypothetical protein | fig|6666666.462.peg.276;Name=Putative helicase |
| YP_001466670.1| DNA gyrase subunit A | fig|6666666.462.peg.277;Name=Putative lipoprotein |
| YP_001466669.1| chemotaxis protein | fig|6666666.462.peg.278;Name=hypothetical protein |
| YP_001466668.1| purine nucleoside | fig|6666666.462.peg.279;Name=hypothetical protein |
| YP_001466667.1| methyl-accepting chemotaxis | fig|6666666.462.peg.280;Name=D-alanine aminotransferase (EC2.6.1.21);Ontology_term=KEGG_ENZYME:2.6.1.21 |
| YP_001466666.1| hypothetical protein | fig|6666666.462.peg.281;Name=membrane protein |
| YP_001466665.1| FlhB domain-containing protein | fig|6666666.462.peg.282;Name=putative membrane protein |
| YP_001466664.1| hypothetical protein | fig|6666666.462.peg.283;Name=membrane protein |
| YP_001466663.1| S-adenosyl-methyltransferase | fig|6666666.462.peg.284;Name=rRNA small subunit methyltransferase H |
| YP_001466662.1| hypothetical protein | fig|6666666.462.peg.285;Name=hypothetical protein |
| YP_001466661.1| glutamate-1-semialdehyde | fig|6666666.462.peg.286;Name=Peptidyl-prolyl cis-trans isomerase ppiD(EC 5.2.1.8);Ontology_term=KEGG_ENZYME:5.2.1.8 |
| YP_001466660.1| cell division protein FtsA | fig|6666666.462.peg.287;Name=Cell division protein FtsA |
| YP_001466659.1| cell division protein FtsZ | fig|6666666.462.peg.288;Name=Cell division protein FtsZ (EC3.4.24.-);Ontology_term=KEGG_ENZYME:3.4.24.- |
| YP_001466658.1| YitW [Campylobacter concisus | fig|6666666.462.peg.289;Name=PaaD-like protein (DUF59) involved inFe-S cluster assembly |
| YP_001466657.1| peptidase M50 [Campylobacter | fig|6666666.462.peg.290;Name=hypothetical protein |
| YP_001466656.1| peptide methionine sulfoxide | fig|6666666.462.peg.292;Name=Peptide methionine sulfoxide reductaseMsrA (EC 1.8.4.11) / Peptide methionine sulfoxidereductase MsrB (EC1.8.4.12);Ontology_term=KEGG_ENZYME:1.8.4.11,KEGG_ENZYME:1.8.4.12 |
| YP_001466655.1| bifunctional | fig|6666666.462.peg.293;Name=IMP cyclohydrolase (EC 3.5.4.10) /Phosphoribosylaminoimidazolecarboxamideformyltransferase (EC2.1.2.3);Ontology_term=KEGG_ENZYME:3.5.4.10,KEGG_ENZYME:2.1.2.3 |
| YP_001466654.1| hypothetical protein | fig|6666666.462.peg.294;Name=hypothetical protein |
| YP_001466653.1| silent information regulator | fig|6666666.462.peg.295;Name=DnaJ-like protein DjlA |
| YP_001466652.1| R2.LlaJI [Campylobacter | fig|6666666.462.peg.296;Name=Similar to phosphoglycolate phosphatase2Cclustered with ribosomal large subunit pseudouridinesynthase C |
| YP_001466650.1| modulator of drug activity B | fig|6666666.462.peg.299;Name=Modulator of drug activity B |
| YP_001466649.1| tRNA modification GTPase TrmE | fig|6666666.462.peg.300;Name=GTPase and tRNA-U34 5-formylation enzymeTrmE |
| YP_001466648.1| hypothetical protein | fig|6666666.462.peg.301;Name=RNA-binding protein Jag |
| YP_001466647.1| putative inner membrane | fig|6666666.462.peg.302;Name=Inner membrane protein translocasecomponent YidC2C long form |
| YP_001466646.1| hypothetical protein | fig|6666666.462.peg.303;Name=Protein YidD |
| YP_001466645.1| hypothetical protein | fig|6666666.462.peg.304;Name=hypothetical protein |
| YP_001466644.1| 50S ribosomal protein L34 | fig|6666666.462.peg.305;Name=LSU ribosomal protein L34p |
| YP_001466643.1| uracil-DNA glycosylase | fig|6666666.462.peg.306;Name=DNA polymerase2C bacteriophage-type (EC2.7.7.7);Ontology_term=KEGG_ENZYME:2.7.7.7 |
| YP_001466642.1| hypothetical protein | fig|6666666.462.peg.307;Name=Putative periplasmic protein |
| YP_001466641.1| thioesterase family protein | fig|6666666.462.peg.308;Name=4-hydroxybenzoyl-CoA thioesterase familyactive site |
| YP_001466640.1| hypothetical protein | fig|6666666.462.peg.309;Name=membrane protein |
| YP_001466639.1| hypothetical protein | fig|6666666.462.peg.310;Name=membrane protein |
| YP_001466638.1| XRE family transcriptional | fig|6666666.462.peg.313;Name=Putative integral membranezinc-metalloprotease |
| YP_001466636.1| sodium/neurotransmitter | fig|6666666.462.peg.315;Name=sodium-dependent transporter |
| YP_001466635.1| hypothetical protein | fig|6666666.462.peg.317;Name=High-affinity choline uptake protein BetT |
| YP_001466634.1| hypothetical protein | fig|6666666.462.peg.318;Name=Fusobacterium membrane protein |
| YP_001466633.1| hypothetical protein | fig|6666666.462.peg.319;Name=hypothetical protein |
| YP_001466632.1| hypothetical protein | fig|6666666.462.peg.320;Name=hypothetical protein |
| YP_001466631.1| peptidase T (tripeptide | fig|6666666.462.peg.321;Name=ABC transport system ATP-binding protein |
| YP_001466630.1| DcuC protein [Campylobacter | fig|6666666.462.peg.322;Name=Na+ driven multidrug efflux pump |
| YP_001466629.1| hypothetical protein | fig|6666666.462.peg.323;Name=Predicted metal-dependent hydrolase |
| YP_001466628.1| Hsp12 variant C [Campylobacter | fig|6666666.462.peg.324;Name=conserved hypothetical secreted protein |
| YP_001466627.1| N-carbamoyl-L-amino acid | fig|6666666.462.peg.325;Name=TRAP-type uncharacterized transportsystem2C fused permease component |
| YP_001466625.1| DNA primase [Campylobacter | fig|6666666.462.peg.327;Name=TRAP transporter solute receptor2C TAXIfamily precursor |
| YP_001466619.1| carbon-nitrogen family | fig|6666666.462.peg.328;Name=N-carbamoylputrescine amidase (3.5.1.53) |
| YP_001466618.1| orotidine 5'-phosphate | fig|6666666.462.peg.330;Name=Agmatine deiminase (EC3.5.3.12);Ontology_term=KEGG_ENZYME:3.5.3.12 |
| YP_001466617.1| putative permease | fig|6666666.462.peg.331;Name=hypothetical protein |
| YP_001466616.1| 6,7-dimethyl-8-ribityllumazine | fig|6666666.462.peg.332;Name=Polar amino acid ABC uptake transportermembrane-spanning protein |
| YP_001466615.1| SugE protein [Campylobacter | fig|6666666.462.peg.333;Name=Amino-acid ABC transporter integralmembrane protein |
| YP_001466614.1| SMR family multidrug efflux | fig|6666666.462.peg.334;Name=Amino-acid ABC transporter ATP-bindingprotein |
| YP_001466613.1| surface antigen, CjaA | fig|6666666.462.peg.335;Name=Putative amino-acid transporterperiplasmic solute-binding protein |
| YP_001466611.1| integral membrane protein | fig|6666666.462.peg.352;Name=Phosphomannomutase (EC 5.4.2.8) /Phosphoglucomutase (EC 5.4.2.2) / Phosphoglucosaminemutase (EC5.4.2.10);Ontology_term=KEGG_ENZYME:5.4.2.8,KEGG_ENZYME:5.4.2.2,KEGG_ENZYME:5.4.2.10 |
| YP_001466610.1| chaperonin GroEL | fig|6666666.462.peg.353;Name=Heat shock protein 60 family chaperoneGroEL |
| YP_001466609.1| co-chaperonin GroES | fig|6666666.462.peg.354;Name=Heat shock protein 60 familyco-chaperone GroES |
| YP_001466608.1| tryptophanyl-tRNA synthetase | fig|6666666.462.peg.355;Name=Putative periplasmic protein |
| YP_001466607.1| ProP protein [Campylobacter | fig|6666666.462.peg.356;Name=Fosmidomycin resistance protein |
| YP_001466603.1| excinuclease ABC subunit B | fig|6666666.462.peg.359;Name=Excinuclease ABC subunit B |
| YP_001466602.1| ABC transporter [Campylobacter | fig|6666666.462.peg.360;Name=hypothetical protein |
| YP_001466601.1| hypothetical protein | fig|6666666.462.peg.361;Name=hypothetical protein |
| YP_001466599.1| primosome assembly protein | fig|6666666.462.peg.363;Name=Helicase PriA essential fororiC/DnaA-independent DNA replication |
| YP_001466597.1| | fig|6666666.462.peg.1660;Name=1-hydroxy-2-methyl-2-(E)-butenyl4-diphosphate synthase (EC1.17.7.1);Ontology_term=KEGG_ENZYME:1.17.7.1 |
| YP_001466596.1| replicative DNA helicase | fig|6666666.462.peg.1659;Name=Replicative DNA helicase (EC3.6.1.-);Ontology_term=KEGG_ENZYME:3.6.1.- |
| YP_001466595.1| hypothetical protein | fig|6666666.462.peg.1657;Name=hypothetical protein |
| YP_001466594.1| hypothetical protein | fig|6666666.462.peg.1656;Name=Putative acetate efflux pump2C MadN |
| YP_001466593.1| putative periplasmic protein | fig|6666666.462.peg.1655;Name=Probable outer membrane component ofmultidrug efflux pump |
| YP_001466592.1| RND transporter, | fig|6666666.462.pe g.1654;Name=Acriflavin resistance protein / Multidrug efflux system CmeDEF |
| YP_001466591.1| selenocysteine-specific | fig|6666666.462.peg.1653;Name=Membrane fusion protein of RND familymultidrug efflux pump |
| YP_001466590.1| L-seryl-tRNA(Sec) selenium | fig|6666666.462.peg.1652;Name=Probable outer membrane component ofmultidrug efflux pump |
| YP_001466589.1| flagellar biosynthesis protein | fig|6666666.462.peg.1651;Name=Flagellar biosynthesis protein FliP |
| YP_001466588.1| OmpA family protein | fig|6666666.462.peg.1650;Name=Flagellar motor rotation protein MotB |
| YP_001466587.1| permease [Campylobacter | fig|6666666.462.peg.1649;Name=Flagellar motor rotation protein MotA |
| YP_001466586.1| UDP-N-acetylglucosamine | fig|6666666.462.peg.1648;Name=N-acetylglucosamine-1-phosphateuridyltransferase (EC 2.7.7.23) /Glucosamine-1-phosphate N-acetyltransferase (EC2.3.1.157);Ontology_term=KEGG_ENZYME:2.7.7.23,KEGG_ENZYME:2.3.1.157 |
| YP_001466585.1| | fig|6666666.462.peg.1645;Name=Undecaprenyl pyrophosphate synthetase (EC2.5.1.31);Ontology_term=KEGG_ENZYME:2.5.1.31 |
| YP_001466584.1| saccharopine dehydrogenase | fig|6666666.462.peg.1644;Name=Leader peptidase (Prepilin peptidase) (EC3.4.23.43);Ontology_term=KEGG_ENZYME:3.4.23.43 |
| YP_001466583.1| ribonuclease PH [Campylobacter | fig|6666666.462.peg.1643;Name=Predicted permease YjgP/YjgQ family |
| YP_001466582.1| tRNA pseudouridine synthase A | fig|6666666.462.peg.1642;Name=tRNA pseudouridine synthase A (EC4.2.1.70);Ontology_term=KEGG_ENZYME:4.2.1.70 |
| YP_001466581.1| hypothetical protein | fig|6666666.462.peg.1641;Name=hypothetical protein |
| YP_001466580.1| adenylosuccinate lyase | fig|6666666.462.peg.1640;Name=Possible dnaJ-like protein |
| YP_001466577.1| putative integral membrane | fig|6666666.462.peg.16;Name=membrane protein |
| YP_001466576.1| outer membrane | fig|6666666.462.peg.15;Name=Putative nucleotidephosphoribosyltransferase (EC2.4.2.22);Ontology_term=KEGG_ENZYME:2.4.2.22 |
| YP_001466575.1| inner membrane protein YicO | fig|6666666.462.peg.14;Name=Putative transmembrane transport protein |
| YP_001466574.1| hypothetical protein | fig|6666666.462.peg.13;Name=Gene SCO44942C often clustered with othergenes in menaquinone via futalosine pathway |
| YP_001466573.1| ATP-dependent DNA helicase | fig|6666666.462.peg.12;Name=Possible nucleotidyltransferase |
| YP_001466572.1| D-fructose-6-phosphate | fig|6666666.462.peg.11;Name=Glucosamine--fructose-6-phosphateaminotransferase [isomerizing] (EC2.6.1.16);Ontology_term=KEGG_ENZYME:2.6.1.16 |
| YP_001466569.1| putative lipoprotein | fig|6666666.462.peg.7;Name=hypothetical protein |
| YP_001466568.1| hypothetical protein | fig|6666666.462.peg.6;Name=22C32C42C5-tetrahydropyridine-22C6-dicarboxylate N-succinyltransferase (EC2.3.1.117);Ontology_term=KEGG_ENZYME:2.3.1.117 |
| YP_001466567.1| hypothetical protein | fig|6666666.462.peg.5;Name=hypothetical protein |
| YP_001466566.1| hypothetical protein | fig|6666666.462.peg.4;Name=hypothetical protein |
| YP_001466565.1| cytochrome c oxidase, diheme | fig|6666666.462.peg.3;Name=hypothetical protein |
| YP_001466564.1| hypothetical protein | fig|6666666.462.peg.2;Name=hypothetical protein |
| YP_001466563.1| hypothetical protein | fig|6666666.462.peg.1;Name=ACT domain containing transcriptionalregulators2C related to gcvR of E.coli |
| YP_001466562.1| cytochrome c oxidase, heme b | fig|6666666.462.peg.600;Name=putative ATP/GTP-binding protein (mrp proteinhomolog) |
| YP_001466561.1| thiamine biosynthesis protein | fig|6666666.462.peg.601;Name=Thiamin biosynthesis protein ThiC |
| YP_001466560.1| bifunctional | fig|6666666.462.peg.602;Name=2-C-methyl-D-erythritol 4-phosphatecytidylyltransferase (EC 2.7.7.60) /2-C-methyl-D-erythritol 22C4-cyclodiphosphate synthase(EC4.6.1.12);Ontology_term=KEGG_ENZYME:2.7.7.60,KEGG_ENZYME:4.6.1.12 |
| YP_001466559.1| putative integral membrane | fig|6666666.462.peg.603;Name=Possible two-component regulator |
| YP_001466558.1| ATP-sulfurylase family protein | fig|6666666.462.peg.604;Name=Possible sulfate adenylyltransferase (EC2.7.7.4);Ontology_term=KEGG_ENZYME:2.7.7.4 |
| YP_001466557.1| phosphatidylglycerophosphatase | fig|6666666.462.peg.605;Name=Phosphatidylglycerophosphatase A (EC3.1.3.27);Ontology_term=KEGG_ENZYME:3.1.3.27 |
| YP_001466554.1| cytochrome C assembly protein | fig|6666666.462.peg. 608;Name=Putative cytochrome C-type biogenesis protein |
| YP_001466553.1| hypothetical protein | fig|6666666.462.peg.609;Name=Putative cytochrome C |
| YP_001466551.1| hypothetical protein | fig|6666666.462.peg.611;Name=hypothetical protein |
| YP_001466550.1| hydrogenase-4 component G | fig|6666666.462.peg.612;Name=hypothetical protein |
| YP_001466549.1| 30S ribosomal protein S7 | fig|6666666.462.peg.616;Name=SSU ribosomal protein S7p (S5e) |
| YP_001466548.1| isoaspartyl dipeptidase | fig|6666666.462.peg.618;Name=Isoaspartyl dipeptidase (EC 3.4.19.5) @Asp-X dipeptidase;Ontology_term=KEGG_ENZYME:3.4.19.5 |
| YP_001466547.1| Na+/H+ antiporter NhaC | fig|6666666.462.peg.619;Name=Na+/H+ antiporter NhaC |
| YP_001466546.1| SRC kinase associated | fig|6666666.462.peg.620;Name=hypothetical protein |
| YP_001466545.1| DNA-directed RNA polymerase, | fig|6666666.462.pe g.621;Name=DNA-directed RNA polymerase beta' subunit (EC 2.7.7.6);Ontology_term=KEGG_ENZYME:2.7.7.6 |
| YP_001466544.1| 50S ribosomal protein L7/L12 | fig|6666666.462.peg.623;Name=LSU ribosomal protein L7/L12 (P1/P2) |
| YP_001466543.1| 50S ribosomal protein L10 | fig|6666666.462.peg.624;Name=LSU ribosomal protein L10p (P0) |
| YP_001466542.1| 50S ribosomal protein L1 | fig|6666666.462.peg.625;Name=LSU ribosomal protein L1p (L10Ae) |
| YP_001466541.1| 50S ribosomal protein L11 | fig|6666666.462.peg.626;Name=LSU ribosomal protein L11p (L12e) |
| YP_001466540.1| transcription antitermination | fig|6666666.462.peg.627;Name=Transcription antitermination protein NusG |
| YP_001466538.1| elongation factor Tu | fig|6666666.462.peg.630;Name=Translation elongation factor Tu |
| YP_001466537.1| flagellar biosynthesis protein | fig|6666666.462.peg.631;Name=hypothetical protein |
| YP_001466536.1| OmpA/MotB [Campylobacter | fig|6666666.462.peg.632;Name=hypothetical protein |
| YP_001466535.1| putative outer membrane | fig|6666666.462.peg.633;Name=Regulatory protein2C LuxR |
| YP_001466534.1| membrane fusion protein | fig|6666666.462.peg.634;Name=hypothetical protein |
| YP_001466533.1| acriflavine resistance protein | fig|6666666.462.peg.635;Name=HlyD family secretion protein |
| YP_001466532.1| hypothetical protein | fig|6666666.462.peg.636;Name=ABC transporter2C transmembrane region:ABCtransporter:Peptidase C392C bacteriocin processing |
| YP_001466531.1| MadN protein [Campylobacter | fig|6666666.462.peg.637;Name=GGDEF and EAL domain proteins |
| YP_001466530.1| periplasmic protein | fig|6666666.462.peg.638;Name=FIGfam010717 |
| YP_001466529.1| ComEC/Rec2 family protein | fig|6666666.462.peg.639;Name=Agglutination protein |
| YP_001466528.1| coproporphyrinogen III oxidase | fig|6666666.462.peg.640;Name=Coproporphyrinogen III oxidase2Coxygen-independent (EC1.3.99.22);Ontology_term=KEGG_ENZYME:1.3.99.22 |
| YP_001466527.1| | fig|6666666.462.peg.641;Name=hypothetical protein |
| YP_001466526.1| ornithine carbamoyltransferase | fig|6666666.462.peg.642;Name=Ornithine carbamoyltransferase (EC2.1.3.3);Ontology_term=KEGG_ENZYME:2.1.3.3 |
| YP_001466525.1| delta-aminolevulinic acid | fig|6666666.462.peg.643;Name=Porphobilinogen synthase (EC4.2.1.24);Ontology_term=KEGG_ENZYME:4.2.1.24 |
| YP_001466524.1| GTP cyclohydrolase II | fig|6666666.462.peg.644;Name=GTP cyclohydrolase II (EC3.5.4.25);Ontology_term=KEGG_ENZYME:3.5.4.25 |
| YP_001466523.1| methyltransferase GidB | fig|6666666.462.peg.645;Name=rRNA small subunit methyltransferase2Cglucose inhibited division protein GidB |
| YP_001466522.1| hypothetical protein | fig|6666666.462.peg.646;Name=hypothetical protein |
| YP_001466521.1| hypothetical protein | fig|6666666.462.peg.647;Name=hypothetical protein |
| YP_001466520.1| M48 family peptidase | fig|6666666.462.peg.648;Name=Heat shock protein HtpX |
| YP_001466519.1| alanine racemase | fig|6666666.462.peg.1669;Name=Alanine racemase (EC5.1.1.1);Ontology_term=KEGG_ENZYME:5.1.1.1 |
| YP_001466518.1| hypothetical protein | fig|6666666.462.peg.1668;Name=hypothetical protein |
| YP_001466517.1| 60 kDa chaperonin (protein | fig|6666666.462.peg.1667;Name=Putative periplasmic protein |
| YP_001466516.1| phosphomannomutase | fig|6666666.462.peg.1666;Name=Copper metallochaperone2C bacterial analog ofCox17 protein |
| YP_001466515.1| hypothetical protein | fig|6666666.462.peg.1665;Name=Putative periplasmic protein |
| YP_001466514.1| SCO1/SenC family protein | fig|6666666.462.peg.1664;Name=Cytochrome oxidase biogenesis proteinSco1/SenC/PrrC2C putative copper metallochaperone |
| YP_001466512.1| nitrate reductase | fig|6666666.462.peg.23;Name=Periplasmic nitrate reductase precursor (EC1.7.99.4);Ontology_term=KEGG_ENZYME:1.7.99.4 |
| YP_001466511.1| quinol dehydrogenase | fig|6666666.462.peg.24;Name=Ferredoxin-type protein NapG (periplasmicnitrate reductase) |
| YP_001466510.1| putative permease | fig|6666666.462.peg.25;Name=Polyferredoxin NapH (periplasmic nitratereductase) |
| YP_001466509.1| periplasmic nitrate reductase, | fig|6666666.462.peg.26;Name=Nitrate reductase cytochrome c550-typesubunit |
| YP_001466508.1| 4Fe-4S ferredoxin, iron-sulfur | fig|6666666.462.peg.27;Name=Ferredoxin-type protein NapF (periplasmicnitrate reductase) |
| YP_001466507.1| carbon-nitrogen family | fig|6666666.462.peg.28;Name=Periplasmic nitrate reductase component NapL |
| YP_001466506.1| hypothetical protein | fig|6666666.462.peg.1733;Name=Possible purine/pyrimidinephosphoribosyltransferase |
| YP_001466505.1| hypothetical protein | fig|6666666.462.peg.1732;Name=hypothetical protein |
| YP_001466504.1| outer membrane lipoprotein | fig|6666666.462.peg.1731;Name=Outer membrane liproprotein mapA precursor |
| YP_001466503.1| hypothetical protein | fig|6666666.462.peg.1730;Name=Putative isomerase |
| YP_001466502.1| hypothetical protein | fig|6666666.462.peg.1729;Name=hypothetical protein |
| YP_001466501.1| GTP-binding protein LepA | fig|6666666.462.peg.1728;Name=Translation elongation factor LepA |
| YP_001466499.1| Hsp12 variant C [Campylobacter | fig|6666666.462.peg.1726;Name=Zn(II) and Co(II) transmembrane diffusionfacilitator |
| YP_001466498.1| hypothetical protein | fig|6666666.462.peg.1725;Name=hypothetical protein |
| YP_001466497.1| mate efflux family protein | fig|6666666.462.peg.1724;Name=hypothetical protein |
| YP_001466495.1| hypothetical protein | fig|6666666.462.peg.1723;Name=Rrf2-linked NADH-flavin reductase |
| YP_001466494.1| transcriptional regulator | fig|6666666.462.peg.1722;Name=hypothetical protein |
| YP_001466493.1| acetoacetate metabolism | fig|6666666.462.peg.35;Name=Signal-transduction regulatory protein FlgR |
| YP_001466492.1| hypothetical protein | fig|6666666.462.peg.36;Name=hypothetical protein |
| YP_001466491.1| DNA gyrase subunit A | fig|6666666.462.peg.38;Name=DNA gyrase subunit A (EC5.99.1.3);Ontology_term=KEGG_ENZYME:5.99.1.3 |
| YP_001466490.1| protein-glutamate | fig|6666666.462.peg.40;Name=Chemotaxis response regulatorprotein-glutamate methylesterase CheB (EC3.1.1.61);Ontology_term=KEGG_ENZYME:3.1.1.61 |
| YP_001466488.1| FlhB domain-containing protein | fig|6666666.462.peg.41;Name=Flagellar biosynthesis protein FlhB |
| YP_001466487.1| hypothetical protein | fig|6666666.462.peg.42;Name=hypothetical protein |
| YP_001466486.1| hypothetical protein | fig|6666666.462.peg.43;Name=Transmembrane transport protein |
| YP_001466485.1| hypothetical protein | fig|6666666.462.peg.44;Name=Probable integral membrane protein Cj0851c |
| YP_001466484.1| hypothetical protein | fig|6666666.462.peg.45;Name=membrane protein |
| YP_001466483.1| glutamate-1-semialdehyde | fig|6666666.462.peg.46;Name=Glutamate-1-semialdehyde aminotransferase(EC 5.4.3.8);Ontology_term=KEGG_ENZYME:5.4.3.8 |
| YP_001466482.1| hypothetical protein | fig|6666666.462.peg.47;Name=Putative periplasmic protein |
| YP_001466481.1| tetrahydrofolate | fig|6666666.462.peg.48;Name=Methylenetetrahydrofolate dehydrogenase(NADP+) (EC 1.5.1.5) / Methenyltetrahydrofolatecyclohydrolase (EC3.5.4.9);Ontology_term=KEGG_ENZYME:1.5.1.5,KEGG_ENZYME:3.5.4.9 |
| YP_001466479.1| M50 family peptidase | fig|6666666.462.peg.50;Name=membrane protein2C putative |
| YP_001466478.1| hypothetical protein | fig|6666666.462.peg.51;Name=hypothetical protein |
| YP_001466477.1| ribose 5-phosphate isomerase B | fig|6666666.462.peg.52;Name=Ribose 5-phosphate isomerase B (EC5.3.1.6);Ontology_term=KEGG_ENZYME:5.3.1.6 |
| YP_001466476.1| hypothetical protein | fig|6666666.462.peg.53;Name=membrane protein |
| YP_001466475.1| adenine | fig|6666666.462.peg.54;Name=Adenine phosphoribosyltransferase (EC2.4.2.7);Ontology_term=KEGG_ENZYME:2.4.2.7 |
| YP_001466474.1| phage integrase family site | fig|6666666.462.peg.55;Name=membrane protein |
| YP_001466473.1| leucyl aminopeptidase | fig|6666666.462.peg.56;Name=Cytosol aminopeptidase PepA (EC3.4.11.1);Ontology_term=KEGG_ENZYME:3.4.11.1 |
| YP_001466472.1| translation-associated GTPase | fig|6666666.462.peg.57;Name=GTP-binding and nucleic acid-bindingprotein YchF |
| YP_001466471.1| hypothetical protein | fig|6666666.462.peg.214;Name=Phosphatidylserine decarboxylase (EC4.1.1.65);Ontology_term=KEGG_ENZYME:4.1.1.65 |
| YP_001466469.1| anthranilate synthase | fig|6666666.462.peg.60;Name=Anthranilate synthase2C aminase component(EC 4.1.3.27);Ontology_term=KEGG_ENZYME:4.1.3.27 |
| YP_001466468.1| Cpp22 [Campylobacter concisus | fig|6666666.462.peg.61;Name=hypothetical protein |
| YP_001466467.1| para protein [Campylobacter | fig|6666666.462.peg.62;Name=hypothetical protein |
| YP_001466466.1| phosphoserine aminotransferase | fig|6666666.462.peg.63;Name=Phosphoserine aminotransferase (EC2.6.1.52);Ontology_term=KEGG_ENZYME:2.6.1.52 |
| YP_001466465.1| 1-deoxy-D-xylulose-5-phosphate | fig|6666666.462.peg.69;Name=1-deoxy-D-xylulose 5-phosphate synthase (EC2.2.1.7);Ontology_term=KEGG_ENZYME:2.2.1.7 |
| YP_001466464.1| flagellar assembly protein H | fig|6666666.462.peg.70;Name=Flagellar assembly protein FliH |
| YP_001466463.1| flagellar motor switch protein | fig|6666666.462.peg.71;Name=Flagellar motor switch protein FliG |
| YP_001466462.1| flagellar MS-ring protein | fig|6666666.462.peg.72;Name=Flagellar M-ring protein FliF |
| YP_001466461.1| histidinol-phosphate | fig|6666666.462.peg.73;Name=Biosynthetic Aromatic amino acidaminotransferase beta (EC 2.6.1.57) @Histidinol-phosphate aminotransferase (EC2.6.1.9);Ontology_term=KEGG_ENZYME:2.6.1.57,KEGG_ENZYME:2.6.1.9 |
| YP_001466460.1| transcription termination | fig|6666666.462.peg.74;Name=Chorismate mutase I (EC 5.4.99.5) /Prephenate dehydratase (EC4.2.1.51);Ontology_term=KEGG_ENZYME:5.4.99.5,KEGG_ENZYME:4.2.1.51 |
| YP_001466459.1| diaminopimelate decarboxylase | fig|6666666.462.peg.75;Name=Diaminopimelate decarboxylase (EC4.1.1.20);Ontology_term=KEGG_ENZYME:4.1.1.20 |
| YP_001466458.1| tRNA-I(6)A37 thiotransferase | fig|6666666.462.peg.76;Name=Permease YjgP/YjgQ |
| YP_001466457.1| lipoprotein [Campylobacter | fig|6666666.462.peg.77;Name=Peptidyl-tRNA hydrolase (EC3.1.1.29);Ontology_term=KEGG_ENZYME:3.1.1.29 |
| YP_001466456.1| 50S ribosomal protein | fig|6666666.462.peg.78;Name=LSU ribosomal protein L25p |
| YP_001466455.1| twitching motility protein | fig|6666666.462.peg.79;Name=Twitching motility protein PilT |
| YP_001466454.1| transaldolase [Campylobacter | fig|6666666.462.peg.80;Name=Transaldolase (EC2.2.1.2);Ontology_term=KEGG_ENZYME:2.2.1.2 |
| YP_001466453.1| phosphoserine phosphatase SerB | fig|6666666.462.peg.81;Name=Phosphoserine phosphatase (EC3.1.3.3);Ontology_term=KEGG_ENZYME:3.1.3.3 |
| YP_001466452.1| putative response regulator of | fig|6666666.462.peg.83;Name=Signal transduction histidine kinase CheA(EC 2.7.3.-);Ontology_term=KEGG_ENZYME:2.7.3.- |
| YP_001466451.1| chemotaxis protein CheV | fig|6666666.462.peg.84;Name=Chemotaxis protein CheV (EC2.7.3.-);Ontology_term=KEGG_ENZYME:2.7.3.- |
| YP_001466450.1| pyridine nucleotide-disulfide | fig|6666666.462.peg.85;Name=hypothetical protein |
| YP_001466449.1| transcription elongation | fig|6666666.462.peg.86;Name=Transcription elongation factor GreA |
| YP_001466448.1| lipid-A-disaccharide synthase | fig|6666666.462.peg.87;Name=Lipid-A-disaccharide synthase (EC2.4.1.182);Ontology_term=KEGG_ENZYME:2.4.1.182 |
| YP_001466447.1| stationary phase survival | fig|6666666.462.peg.88;Name=5-nucleotidase SurE (EC3.1.3.5);Ontology_term=KEGG_ENZYME:3.1.3.5 |
| YP_001466446.1| peptide chain release factor 2 | fig|6666666.462.peg.89;Name=MoeB/thiF family protein |
| YP_001466443.1| tRNA(Ile)-lysidine synthase | fig|6666666.462.peg.1440;Name=Dihydroorotase (EC3.5.2.3);Ontology_term=KEGG_ENZYME:3.5.2.3 |
| YP_001466442.1| elongation factor P | fig|6666666.462.peg.1441;Name=Translation elongation factor P |
| YP_001466441.1| D-3-phosphoglycerate | fig|6666666.462.peg.1442;Name=D-3-phosphoglycerate dehydrogenase (EC1.1.1.95);Ontology_term=KEGG_ENZYME:1.1.1.95 |
| YP_001466440.1| hypothetical protein | fig|6666666.462.peg.1443;Name=Putative periplasmic protein |
| YP_001466439.1| 30S ribosomal protein S1 | fig|6666666.462.peg.1444;Name=SSU ribosomal protein S1p |
| YP_001466438.1| 4-hydroxy-3-methylbut-2-enyl | fig|6666666.462.peg.1445;Name=4-hydroxy-3-methylbut-2-enyl diphosphatereductase (EC1.17.1.2);Ontology_term=KEGG_ENZYME:1.17.1.2 |
| YP_001466437.1| phenylalanyl-tRNA synthetase | fig|6666666.462.peg.1448;Name=Phenylalanyl-tRNA synthetase alpha chain(EC 6.1.1.20);Ontology_term=KEGG_ENZYME:6.1.1.20 |
| YP_001466436.1| hypothetical protein | fig|6666666.462.peg.212;Name=hypothetical protein |
| YP_001466435.1| excinuclease ABC subunit C | fig|6666666.462.peg.211;Name=Excinuclease ABC subunit C |
| YP_001466434.1| hypothetical protein | fig|6666666.462.peg.210;Name=hypothetical protein |
| YP_001466433.1| hypothetical protein | fig|6666666.462.peg.209;Name=hypothetical protein |
| YP_001466432.1| hypothetical protein | fig|6666666.462.peg.208;Name=Na+/H+ antiporter NhaD type |
| YP_001466431.1| bifunctional GMP | fig|6666666.462.peg.207;Name=GMP synthase [glutamine-hydrolyzing] (EC6.3.5.2);Ontology_term=KEGG_ENZYME:6.3.5.2 |
| YP_001466430.1| phosphatidylserine | fig|6666666.462.peg.214;Name=Phosphatidylserine decarboxylase (EC4.1.1.65);Ontology_term=KEGG_ENZYME:4.1.1.65 |
| YP_001466429.1| acyl carrier protein | fig|6666666.462.peg.215;Name=Predicted phosphohydrolase |
| YP_001466428.1| beta ketoacyl-acyl carrier | fig|6666666.462.peg.216;Name=Hydroxylamine oxidoreductase (Fragment) |
| YP_001466427.1| acetyl-coenzyme A carboxylase | fig|6666666.462.peg.217;Name=Functional role page for Cytochrome c-typeprotein TorY |
| YP_001466426.1| protein YieJ [Campylobacter | fig|6666666.462.peg.218;Name=transcriptional regulator2C Crp/Fnr family |
| YP_001466425.1| large conductance | fig|6666666.462.peg.219;Name=Large-conductance mechanosensitive channel |
| YP_001466424.1| glutamyl-tRNA synthetase | fig|6666666.462.peg.220;Name=Glutamyl-tRNA(Gln) synthetase (EC6.1.1.24);Ontology_term=KEGG_ENZYME:6.1.1.24 |
| YP_001466423.1| riboflavin synthase alpha | fig|6666666.462.peg.221;Name=membrane protein |
| YP_001466422.1| transglycosylase SLT | fig|6666666.462.peg.222;Name=Soluble lytic murein transglycosylaseprecursor (EC 3.2.1.-);Ontology_term=KEGG_ENZYME:3.2.1.- |
| YP_001466421.1| molybdopterin-guanine | fig|6666666.462.peg.223;Name=Putative ATP/GTP binding protein |
| YP_001466420.1| fructose-1,6-bisphosphatase | fig|6666666.462.peg.224;Name=Fructose-12C6-bisphosphatase2C type I (EC3.1.3.11);Ontology_term=KEGG_ENZYME:3.1.3.11 |
| YP_001466419.1| hypothetical protein | fig|6666666.462.peg.225;Name=hypothetical protein |
| YP_001466418.1| hydroxylamine reductase | fig|6666666.462.peg.228;Name=Hydroxylamine reductase (EC1.7.-.-);Ontology_term=KEGG_ENZYME:1.7.-.- |
| YP_001466416.1| ExsB protein [Campylobacter | fig|6666666.462.peg.230;Name=Queuosine Biosynthesis QueC ATPase |
| YP_001466415.1| putative metalloprotease | fig|6666666.462.peg.231;Name=FIG000233: metal-dependent hydrolase |
| YP_001466414.1| twin-arginine translocation | fig|6666666.462.peg.232;Name=hypothetical protein |
| YP_001466413.1| arginyl-tRNA synthetase | fig|6666666.462.peg.233;Name=Nitric-oxide reductase (EC 1.7.99.7)2Cquinol-dependent;Ontology_term=KEGG_ENZYME:1.7.99.7 |
| YP_001466411.1| hexapaptide repeat-containing | fig|6666666.462.peg.235;Name=Vitamin B12 ABC transporter2C permeasecomponent BtuC |
| YP_001466410.1| iron compounds ABC | fig|6666666.462.peg.236;Name=iron compounds ABC transporter2CATP-binding protein |
| YP_001466408.1| res subunit family type III | fig|6666666.462.peg.239;Name=ATP-dependent DNA helicase RecG (EC3.6.1.-);Ontology_term=KEGG_ENZYME:3.6.1.- |
| YP_001466407.1| processing protease | fig|6666666.462.peg.240;Name=Zinc protease-like protein |
| YP_001466406.1| hypothetical protein | fig|6666666.462.peg.241;Name=Menaquinone via futalosine step 3 |
| YP_001466405.1| hypothetical protein | fig|6666666.462.peg.243;Name=hypothetical protein |
| YP_001466404.1| nodulation efficiency protein | fig|6666666.462.peg.244;Name=hypothetical protein |
| YP_001466403.1| band 7/Mec-2 family protein | fig|6666666.462.peg.245;Name=Putative stomatin/prohibitin-familymembrane protease subunit YbbK |
| YP_001466402.1| hypothetical protein | fig|6666666.462.peg.247;Name=Lipoprotein |
| YP_001466401.1| hypothetical protein | fig|6666666.462.peg.248;Name=hypothetical protein |
| YP_001466400.1| putative methionyl-tRNA | fig|6666666.462.peg.249;Name=Putative integral membrane protein |
| YP_001466399.1| methyl-accepting chemotaxis | fig|6666666.462.peg.1450;Name=PUTATIVE MCP-TYPE SIGNAL TRANSDUCTIONPROTEIN |
| YP_001466398.1| argininosuccinate lyase | fig|6666666.462.peg.1451;Name=Argininosuccinate lyase (EC4.3.2.1);Ontology_term=KEGG_ENZYME:4.3.2.1 |
| YP_001466396.1| hypothetical protein | fig|6666666.462.peg.1452;Name=Putative oxidoreductase component ofanaerobic dehydrogenases%3B Functional role page forChaperone protein TorD |
| YP_001466395.1| asparagine synthase, | fig|6666666.462.peg.1454;Name=hypothetical protein |
| YP_001466394.1| acetyltransferase | fig|6666666.462.peg.1455;Name=Histone acetyltransferase HPA2 and relatedacetyltransferases |
| YP_001466393.1| hypothetical protein | fig|6666666.462.peg.1456;Name=FIG000859: hypothetical protein |
| YP_001466392.1| peptidyl-prolyl cis-trans | fig|6666666.462.peg.1457;Name=Peptidyl-prolyl cis-trans isomerase (EC5.2.1.8);Ontology_term=KEGG_ENZYME:5.2.1.8 |
| YP_001466391.1| glycosyl transferase, group 1 | fig|6666666.462.peg.1458;Name=Putative C4-dicarboxylate transport protein |
| YP_001466390.1| aspartate racemase | fig|6666666.462.peg.1459;Name=aspartate racemase |
| YP_001466388.1| phosphoenolpyruvate | fig|6666666.462.peg.1460;Name=Phosphoenolpyruvate carboxykinase [ATP] (EC4.1.1.49);Ontology_term=KEGG_ENZYME:4.1.1.49 |
| YP_001466387.1| glyceraldehyde-3-phosphate | fig|6666666.462.peg.1461;Name=Pyruvate carboxyl transferase subunit B (EC6.4.1.1);Ontology_term=KEGG_ENZYME:6.4.1.1 |
| YP_001466386.1| trap dicarboxylate | fig|6666666.462.peg.1462;Name=TRAP dicarboxylate transporter2C DctMsubunit2C unknown substrate 6 |
| YP_001466385.1| triosephosphate isomerase | fig|6666666.462.peg.1463;Name=TRAP dicarboxylate transporter2C DctQsubunit2C unknown substrate 6 |
| YP_001466384.1| sodium:neurotransmitter | fig|6666666.462.peg.1464;Name=Putative transmembrane transport protein |
| YP_001466383.1| sodium/neurotransmitter | fig|6666666.462.peg.1465;Name=Putative transmembrane transport protein |
| YP_001466382.1| cell division membrane protein | fig|6666666.462.peg.1466;Name=Putative transmembrane transport protein |
| YP_001466381.1| F0F1 ATP synthase subunit C | fig|6666666.462.peg.1467;Name=ATP synthase C chain (EC3.6.3.14);Ontology_term=KEGG_ENZYME:3.6.3.14 |
| YP_001466380.1| riboflavin biosynthesis | fig|6666666.462.peg.1469;Name=Diaminohydroxyphosphoribosylaminopyrimidinedeaminase (EC 3.5.4.26) /5-amino-6-(5-phosphoribosylamino)uracil reductase (EC1.1.1.193);Ontology_term=KEGG_ENZYME:3.5.4.26,KEGG_ENZYME:1.1.1.193 |
| YP_001466379.1| hypothetical protein | fig|6666666.462.peg.1471;Name=COG0779: clustered with transcriptiontermination protein NusA |
| YP_001466378.1| ribosome-binding factor A | fig|6666666.462.peg.1472;Name=Ribosome-binding factor A |
| YP_001466377.1| translation initiation factor | fig|6666666.462.peg.1473;Name=Translation initiation factor 2 |
| YP_001466376.1| hypothetical protein | fig|6666666.462.peg.1474;Name=hypothetical protein |
| YP_001466375.1| homoserine kinase | fig|6666666.462.peg.1475;Name=Homoserine kinase (EC2.7.1.39);Ontology_term=KEGG_ENZYME:2.7.1.39 |
| YP_001466374.1| glycoprotease family protein | fig|6666666.462.peg.1476;Name=Inactive homolog of metal-dependentproteases2C putative molecular chaperone |
| YP_001466373.1| UDP-3-O-[3-hydroxymyristoyl] | fig|6666666.462.peg.1477;Name=UDP-3-O-[3-hydroxymyristoyl]N-acetylglucosamine deacetylase (EC3.5.1.-);Ontology_term=KEGG_ENZYME:3.5.1.- |
| YP_001466372.1| tyrosyl-tRNA synthetase | fig|6666666.462.peg.1478;Name=Putative periplasmic protein |
| YP_001466371.1| prephenate dehydrogenase | fig|6666666.462.peg.1479;Name=Prephenate and/or arogenate dehydrogenase(unknown specificity) (EC 1.3.1.12)(EC1.3.1.43);Ontology_term=KEGG_ENZYME:1.3.1.12,KEGG_ENZYME:1.3.1.43 |
| YP_001466370.1| outer membrane protein | fig|6666666.462.peg.1480;Name=Outer membrane protein assembly factor YaeTprecursor |
| YP_001466369.1| acetyl-CoA carboxylase subunit | fig|6666666.462.peg.1481;Name=Acetyl-coenzyme A carboxyl transferase betachain (EC 6.4.1.2);Ontology_term=KEGG_ENZYME:6.4.1.2 |
| YP_001466368.1| hypothetical protein | fig|6666666.462.peg.1482;Name=LSU m3Psi1915 methyltransferase RlmH |
| YP_001466367.1| TraR/DksA family | fig|6666666.462.peg.1483;Name=C4-type zinc finger protein2C DksA/TraRfamily |
| YP_001466366.1| uroporphyrinogen III synthase | fig|6666666.462.peg.1484;Name=Probable membrane protein Cj0124c |
| YP_001466365.1| phosphoribosylamine--glycine | fig|6666666.462.peg.1485;Name=tRNA dihydrouridine synthase B (EC1.-.-.-);Ontology_term=KEGG_ENZYME:1.-.-.- |
| YP_001466364.1| hypothetical protein | fig|6666666.462.peg.1486;Name=hypothetical protein |
| YP_001466361.1| polyribonucleotide | fig|6666666.462.peg.1508;Name=hypothetical protein |
| YP_001466357.1| glutamyl-tRNA synthetase | fig|6666666.462.peg.1152;Name=hypothetical protein |
| YP_001466356.1| PP-loop family protein | fig|6666666.462.peg.1151;Name=tRNA(Cytosine32)-2-thiocytidine synthetase |
| YP_001466355.1| | fig|6666666.462.peg.1150;Name=Menaquinone via futalosine step 2 |
| YP_001466354.1| malonyl CoA-acyl carrier | fig|6666666.462.peg.1149;Name=Malonyl CoA-acyl carrier proteintransacylase (EC2.3.1.39);Ontology_term=KEGG_ENZYME:2.3.1.39 |
| YP_001466353.1| putative peptidyl-prolyl | fig|6666666.462.peg.1148;Name=FKBP-type peptidyl-prolyl cis-transisomerase SlyD (EC5.2.1.8);Ontology_term=KEGG_ENZYME:5.2.1.8 |
| YP_001466352.1| ADP-heptose-LPS | fig|6666666.462.peg.1147;Name=TPR repeat containing exported protein%3BPutative periplasmic protein contains a proteinprenylyltransferase domain |
| YP_001466351.1| Omp18 [Campylobacter concisus | fig|6666666.462.peg.1146;Name=Outer membrane lipoprotein omp16 precursor |
| YP_001466350.1| translocation protein TolB | fig|6666666.462.peg.1145;Name=tolB protein precursor2C periplasmicprotein involved in the tonb-independent uptake of groupA colicins |
| YP_001466349.1| putative periplasmic protein | fig|6666666.462.peg.1144;Name=TonB-like%3B putative TolA function |
| YP_001466348.1| putative polysaccharide | fig|6666666.462.peg.1143;Name=Biopolymer transport protein ExbD/TolR |
| YP_001466347.1| ExbB\TolQ family transport | fig|6666666.462.peg.1142;Name=MotA/TolQ/ExbB proton channel familyprotein |
| YP_001466346.1| F0F1 ATP synthase subunit | fig|6666666.462.peg.1141;Name=ATP synthase epsilon chain (EC3.6.3.14);Ontology_term=KEGG_ENZYME:3.6.3.14 |
| YP_001466345.1| F0F1 ATP synthase subunit beta | fig|6666666.462.peg.1140;Name=ATP synthase beta chain (EC3.6.3.14);Ontology_term=KEGG_ENZYME:3.6.3.14 |
| YP_001466344.1| F0F1 ATP synthase subunit | fig|6666666.462.peg.1139;Name=ATP synthase gamma chain (EC3.6.3.14);Ontology_term=KEGG_ENZYME:3.6.3.14 |
| YP_001466343.1| F0F1 ATP synthase subunit | fig|6666666.462.peg.1138;Name=ATP synthase alpha chain (EC3.6.3.14);Ontology_term=KEGG_ENZYME:3.6.3.14 |
| YP_001466342.1| F0F1 ATP synthase subunit | fig|6666666.462.peg.1137;Name=ATP synthase delta chain (EC3.6.3.14);Ontology_term=KEGG_ENZYME:3.6.3.14 |
| YP_001466341.1| F0F1 ATP synthase subunit B | fig|6666666.462.peg.1136;Name=ATP synthase B chain (EC3.6.3.14);Ontology_term=KEGG_ENZYME:3.6.3.14 |
| YP_001466340.1| F0F1 ATP synthase subunit B' | fig|6666666.462.peg.1135;Name=ATP synthase B' chain (EC3.6.3.14);Ontology_term=KEGG_ENZYME:3.6.3.14 |
| YP_001466339.1| stage 0 sporulation protein J | fig|6666666.462.peg.1134;Name=Chromosome (plasmid) partitioning proteinParB / Stage 0 sporulation protein J |
| YP_001466338.1| sporulation initiation | fig|6666666.462.peg.1133;Name=Chromosome (plasmid) partitioning proteinParA |
| YP_001466337.1| biotin--protein ligase | fig|6666666.462.peg.1132;Name=Biotin-protein ligase (EC6.3.4.15);Ontology_term=KEGG_ENZYME:6.3.4.15 |
| YP_001466336.1| phosphomethylpyrimidine kinase | fig|6666666.462.peg.1131;Name=Phosphomethylpyrimidine kinase (EC2.7.4.7);Ontology_term=KEGG_ENZYME:2.7.4.7 |
| YP_001466335.1| methionyl-tRNA | fig|6666666.462.peg.1130;Name=Methionyl-tRNA formyltransferase (EC2.1.2.9);Ontology_term=KEGG_ENZYME:2.1.2.9 |
| YP_001466334.1| UDP-glucose/GDP-mannose | fig|6666666.462.peg.1129;Name=hypothetical protein |
| YP_001466330.1| L(+)-tartrate dehydratase | fig|6666666.462.peg.1359;Name=L(+)-tartrate dehydratase beta subunit (EC4.2.1.32);Ontology_term=KEGG_ENZYME:4.2.1.32 |
| YP_001466329.1| tartrate dehydratase subunit | fig|6666666.462.peg.1358;Name=L(+)-tartrate dehydratase alpha subunit(EC 4.2.1.32);Ontology_term=KEGG_ENZYME:4.2.1.32 |
| YP_001466328.1| hypothetical protein | fig|6666666.462.peg.1357;Name=hypothetical protein |
| YP_001466327.1| ncair mutase [Campylobacter | fig|6666666.462.peg.1356;Name=Circadian phase modifier @ NCAIR mutase(PurE)-related protein |
| YP_001466326.1| hypothetical protein | fig|6666666.462.peg.1355;Name=ATP-utilizing enzyme of the PP-loopsuperfamily |
| YP_001466325.1| hypothetical protein | fig|6666666.462.peg.1354;Name=FIG099352: hypothetical protein |
| YP_001466324.1| general glycosylation pathway | fig|6666666.462.peg.1353;Name=Transcriptional regulator |
| YP_001466323.1| pyridoxal phosphate-dependent | fig|6666666.462.peg.1352;Name=Putative anaerobic C4-dicarboxylatetransporter |
| YP_001466322.1| flagellar biosynthesis protein | fig|6666666.462.peg.1351;Name=Flagellar biosynthesis protein FlhB |
| YP_001466321.1| hypothetical protein | fig|6666666.462.peg.1349;Name=hypothetical protein |
| YP_001466320.1| 50S ribosomal protein L21 | fig|6666666.462.peg.1348;Name=LSU ribosomal protein L21p |
| YP_001466319.1| 50S ribosomal protein L27 | fig|6666666.462.peg.1347;Name=LSU ribosomal protein L27p |
| YP_001466315.1| excinuclease ABC subunit A | fig|6666666.462.peg.1346;Name=Excinuclease ABC subunit A |
| YP_001466314.1| CDP-diacylglycerol--serine | fig|6666666.462.peg.1345;Name=Ferredoxin |
| YP_001466313.1| nucleoside diphosphate kinase | fig|6666666.462.peg.1344;Name=Nucleoside diphosphate kinase (EC2.7.4.6);Ontology_term=KEGG_ENZYME:2.7.4.6 |
| YP_001466312.1| hypothetical protein | fig|6666666.462.peg.1343;Name=hypothetical protein |
| YP_001466311.1| 50S ribosomal protein L32 | fig|6666666.462.peg.1342;Name=LSU ribosomal protein L32p |
| YP_001466310.1| putative glycerol-3-phosphate | fig|6666666.462.peg.1341;Name=Phosphate:acyl-ACP acyltransferase PlsX |
| YP_001466309.1| 3-oxoacyl-(acyl carrier | fig|6666666.462.peg.1340;Name=3-oxoacyl-[acyl-carrier-protein]synthase2C KASIII (EC2.3.1.41);Ontology_term=KEGG_ENZYME:2.3.1.41 |
| YP_001466308.1| 2-cys peroxiredoxin BAS1, | fig|6666666.462.peg.1339;Name=Alkyl hydroperoxide reductase subunitC-like protein |
| YP_001466307.1| type IV pilus assembly protein | fig|6666666.462.peg.1338;Name=LSU ribosomal protein L12a (P1/P2) |
| YP_001466306.1| hypothetical protein | fig|6666666.462.peg.1337;Name=conserved hypothetical protein-putativeATP-binding protein |
| YP_001466305.1| putative glycerol-3-phosphate | fig|6666666.462.peg.1336;Name=Acyl-phosphate:glycerol-3-phosphateO-acyltransferase PlsY |
| YP_001466304.1| dihydroneopterin aldolase | fig|6666666.462.peg.1335;Name=Dihydroneopterin aldolase (EC4.1.2.25);Ontology_term=KEGG_ENZYME:4.1.2.25 |
| YP_001466303.1| response regulator receiver | fig|6666666.462.peg.1334;Name=Two-component regulator |
| YP_001466302.1| prolipoprotein diacylglyceryl | fig|6666666.462.peg.1333;Name=hypothetical protein |
| YP_001466301.1| fumarate reductase | fig|6666666.462.peg.1332;Name=Exopolyphosphatase (EC3.6.1.11);Ontology_term=KEGG_ENZYME:3.6.1.11 |
| YP_001466299.1| hypothetical protein | fig|6666666.462.peg.1331;Name=Probable transmembrane protein Cj0352 |
| YP_001466298.1| flagellar motor switch protein | fig|6666666.462.peg.1330;Name=Flagellar motor switch protein FliN |
| YP_001466297.1| Mrr restriction system protein | fig|6666666.462.peg.1329;Name=Putative uncharacterized protein FIG019238 |
| YP_001466296.1| tryptophan synthase subunit | fig|6666666.462.peg.1328;Name=Tryptophan synthase alpha chain (EC4.2.1.20);Ontology_term=KEGG_ENZYME:4.2.1.20 |
| YP_001466295.1| tryptophan synthase subunit | fig|6666666.462.peg.1327;Name=Tryptophan synthase beta chain (EC4.2.1.20);Ontology_term=KEGG_ENZYME:4.2.1.20 |
| YP_001466294.1| | fig|6666666.462.peg.1326;Name=Phosphoribosylanthranilate isomerase (EC5.3.1.24);Ontology_term=KEGG_ENZYME:5.3.1.24 |
| YP_001466293.1| anthranilate | fig|6666666.462.peg.1325;Name=Anthranilate synthase2C amidotransferasecomponent (EC 4.1.3.27) / Anthranilatephosphoribosyltransferase (EC2.4.2.18);Ontology_term=KEGG_ENZYME:4.1.3.27,KEGG_ENZYME:2.4.2.18 |
| YP_001466292.1| recombination protein RecR | fig|6666666.462.peg.1324;Name=Recombination protein RecR |
| YP_001466291.1| proline permease | fig|6666666.462.peg.1323;Name=Two-component system histidine kinase RacS |
| YP_001466290.1| His/Glu/Gln/Arg/opine amino | fig|6666666.462.peg.1322;Name=Two-component system response regulator RacR |
| YP_001466288.1| chaperone protein DnaJ | fig|6666666.462.peg.1321;Name=Chaperone protein DnaJ |
| YP_001466287.1| pyruvate kinase [Campylobacter | fig|6666666.462.peg.1510;Name=Pyruvate kinase (EC2.7.1.40);Ontology_term=KEGG_ENZYME:2.7.1.40 |
| YP_001466286.1| CjaC [Campylobacter concisus | fig|6666666.462.peg.1511;Name=TRAP transporter solute receptor2C unknownsubstrate 6 |
| YP_001466282.1| trimethylamine-n-oxide | fig|6666666.462.peg.1512;Name=Putative molybdopterin biosynthesis protein |
| YP_001466281.1| metal-activated pyridoxal | fig|6666666.462.peg.1513;Name=Conserved domain protein |
| YP_001466244.1| periplasmic binding protein | fig|6666666.462.peg.1528;Name=hypothetical protein |
| YP_001466243.1| ATP-binding protein | fig|6666666.462.peg.1527;Name=tRNA(5-methylaminomethyl-2-thiouridylate)-methyltransferase(EC 2.1.1.61);Ontology_term=KEGG_ENZYME:2.1.1.61 |
| YP_001466242.1| peptidase T [Campylobacter | fig|6666666.462.peg.1526;Name=Tripeptide aminopeptidase (EC3.4.11.4);Ontology_term=KEGG_ENZYME:3.4.11.4 |
| YP_001466241.1| peptidase E [Campylobacter | fig|6666666.462.peg.1524;Name=Alpha-aspartyl dipeptidase Peptidase E (EC3.4.13.21);Ontology_term=KEGG_ENZYME:3.4.13.21 |
| YP_001466240.1| aminobenzoyl-glutamate | fig|6666666.462.peg.1523;Name=Catalyzes the cleavage ofp-aminobenzoyl-glutamate to p-aminobenzoate andglutamate2C subunit A |
| YP_001466239.1| DNA primase [Campylobacter | fig|6666666.462.peg.1521;Name=DNA primase (EC2.7.7.-);Ontology_term=KEGG_ENZYME:2.7.7.- |
| YP_001466238.1| SpoOJ regulator [Campylobacter | fig|6666666.462.peg.1520;Name=Putative periplasmic protein |
| YP_001466235.1| transcription antitermination | fig|6666666.462.peg.1775;Name=Transcription termination protein NusB |
| YP_001466234.1| riboflavin synthase subunit | fig|6666666.462.peg.1776;Name=62C7-dimethyl-8-ribityllumazine synthase (EC2.5.1.9);Ontology_term=KEGG_ENZYME:2.5.1.9 |
| YP_001466233.1| methionyl-tRNA | fig|6666666.462.peg.1777;Name=Quaternary ammonium compound-resistanceprotein sugE |
| YP_001466232.1| hypothetical protein | fig|6666666.462.peg.1779;Name=hypothetical protein |
| YP_001466231.1| | fig|6666666.462.peg.1780;Name=2-Keto-3-deoxy-D-manno-octulosonate-8-phosphate synthase (EC2.5.1.55);Ontology_term=KEGG_ENZYME:2.5.1.55 |
| YP_001466230.1| hypothetical protein | fig|6666666.462.peg.1781;Name=Putative integral membrane protein |
| YP_001466229.1| GTP-binding protein EngA | fig|6666666.462.peg.1782;Name=GTP-binding protein EngA |
| YP_001466228.1| shikimate kinase | fig|6666666.462.peg.1783;Name=Shikimate kinase I (EC2.7.1.71);Ontology_term=KEGG_ENZYME:2.7.1.71 |
| YP_001466227.1| tryptophanyl-tRNA synthetase | fig|6666666.462.peg.1785;Name=Tryptophanyl-tRNA synthetase (EC6.1.1.2);Ontology_term=KEGG_ENZYME:6.1.1.2 |
| YP_001466226.1| protein translocation protein, | fig|6666666.462.peg.1786;Name=hypothetical protein |
| YP_001466225.1| seryl-tRNA synthetase | fig|6666666.462.peg.1787;Name=Seryl-tRNA synthetase (EC6.1.1.11);Ontology_term=KEGG_ENZYME:6.1.1.11 |
| YP_001466224.1| integral membrane protein | fig|6666666.462.peg.1788;Name=membrane protein |
| YP_001466222.1| hypothetical protein | fig|6666666.462.peg.1790;Name=hypothetical protein |
| YP_001466221.1| thioredoxin family protein | fig|6666666.462.peg.1791;Name=Possible lipoprotein thiredoxin |
| YP_001466220.1| response regulatory protein | fig|6666666.462.peg.1792;Name=Putative ABC transport system ATP-bindingprotein |
| YP_001466219.1| integral membrane | fig|6666666.462.peg.1793;Name=Cell division protein FtsX |
| YP_001466218.1| ABC transport permease | fig|6666666.462.peg.1794;Name=Cell division protein FtsX |
| YP_001466217.1| hypothetical protein | fig|6666666.462.peg.1795;Name=membrane protein |
| YP_001466216.1| C4-dicarboxylate-binding | fig|6666666.462.peg.1796;Name=Periplasmic protein p19 involved inhigh-affinity Fe2+ transport |
| YP_001466215.1| superoxide dismutase | fig|6666666.462.peg.1797;Name=Putative high-affinity iron permease |
| YP_001466214.1| formate dehydrogenase | fig|6666666.462.peg.1798;Name=Formate dehydrogenase-O2C iron-sulfursubunit (EC 1.2.1.2)%3B Putative formate dehydrogenaseiron-sulfur subunit (EC1.2.1.2);Ontology_term=KEGG_ENZYME:1.2.1.2,KEGG_ENZYME:1.2.1.2 |
| YP_001466213.1| hypothetical protein | fig|6666666.462.peg.1799;Name=Formate dehydrogenase-O2C major subunit (EC1.2.1.2) @selenocysteine-containing;Ontology_term=KEGG_ENZYME:1.2.1.2 |
| YP_001466212.1| hypothetical protein | fig|6666666.462.peg.1800;Name=Formate dehydrogenase subunit oraccessory protein |
| YP_001466211.1| thiol peroxidase | fig|6666666.462.peg.1801;Name=Putative periplasmic protein |
| YP_001466209.1| selenocysteine-specific | fig|6666666.462.peg.1802;Name=Selenocysteine-specific translationelongation factor |
| YP_001466208.1| selenocysteine synthase | fig|6666666.462.peg.1803;Name=L-seryl-tRNA(Sec) selenium transferase (EC2.9.1.1);Ontology_term=KEGG_ENZYME:2.9.1.1 |
| YP_001466207.1| iron-sulfur cluster-binding | fig|6666666.462.peg.1804;Name=Iron-sulfur cluster-binding protein |
| YP_001466206.1| hypothetical protein | fig|6666666.462.peg.1805;Name=Putative formate dehydrogenase-specificchaperone |
| YP_001466205.1| permease [Campylobacter | fig|6666666.462.peg.1806;Name=Cytochrome oxidase biogenesis proteinSurf12C facilitates heme A insertion |
| YP_001466204.1| RmuC domain-containing protein | fig|6666666.462.peg.662;Name=DNA recombination protein RmuC |
| YP_001466203.1| saccharopine dehydrogenase | fig|6666666.462.peg.658;Name=Carboxynorspermidine dehydrogenase2C putative(EC 1.1.1.-);Ontology_term=KEGG_ENZYME:1.1.1.- |
| YP_001466201.1| Ham1 family protein | fig|6666666.462.peg.656;Name=Xanthosine/inosine triphosphatepyrophosphatase |
| YP_001466200.1| major facilitator transporter | fig|6666666.462.peg.655;Name=Putative efflux protein |
| YP_001466199.1| putative pyridine | fig|6666666.462.peg.654;Name=NAD(FAD)-utilizing dehydrogenases |
| YP_001466198.1| hypothetical protein | fig|6666666.462.peg.206;Name=hypothetical protein |
| YP_001466197.1| carbamoyl-phosphate synthase | fig|6666666.462.pe g.205;Name=Carbamoyl-phosphate synthase large chain (EC 6.3.5.5);Ontology_term=KEGG_ENZYME:6.3.5.5 |
| YP_001466196.1| hypothetical protein | fig|6666666.462.peg.875;Name=Phosphoglycolate phosphatase (EC3.1.3.18);Ontology_term=KEGG_ENZYME:3.1.3.18 |
| YP_001466195.1| outer membrane | fig|6666666.462.peg.876;Name=Outer membrane fibronectin-binding protein |
| YP_001466194.1| 30S ribosomal protein S9 | fig|6666666.462.peg.877;Name=SSU ribosomal protein S9p (S16e) |
| YP_001466193.1| 50S ribosomal protein L13 | fig|6666666.462.peg.878;Name=LSU ribosomal protein L13p (L13Ae) |
| YP_001466192.1| ribonucleoside-diphosphate | fig|6666666.462.peg.879;Name=Helicase |
| YP_001466191.1| hypothetical protein | fig|6666666.462.peg.880;Name=hypothetical protein |
| YP_001466188.1| Na+/H+ antiporter NhaA | fig|6666666.462.peg.882;Name=Na+/H+ antiporter NhaA type |
| YP_001466186.1| putative lipoprotein | fig|6666666.462.peg.884;Name=Putative lipoprotein |
| YP_001466185.1| hypothetical protein | fig|6666666.462.peg.885;Name=membrane protein |
| YP_001466184.1| hypothetical protein | fig|6666666.462.peg.886;Name=Putative periplasmic protein |
| YP_001466183.1| cytochrome c oxidase, | fig|6666666.462.peg.887;Name=Cytochrome c oxidase subunit CcoP (EC1.9.3.1);Ontology_term=KEGG_ENZYME:1.9.3.1 |
| YP_001466182.1| cytochrome c oxidase, | fig|6666666.462.peg.888;Name=Cytochrome c oxidase subunit CcoQ (EC1.9.3.1);Ontology_term=KEGG_ENZYME:1.9.3.1 |
| YP_001466180.1| hypothetical protein | fig|6666666.462.peg.892;Name=Putative two-component sensor histidinekinase |
| YP_001466179.1| hypothetical protein | fig|6666666.462.peg.893;Name=Heavy-metal-associated domain (N-terminus)and membrane-bounded cytochrome biogenesis cycZ-likedomain2C possible membrane copper tolerance protein |
| YP_001466178.1| competence/damage-inducible | fig|6666666.462.peg.895;Name=Hypothetical protein probably associatedwith Carbamoyl-phosphate synthase |
| YP_001466177.1| isoleucyl-tRNA synthetase | fig|6666666.462.peg.896;Name=hypothetical protein |
| YP_001466176.1| formate/nitrite transporter | fig|6666666.462.peg.897;Name=Formate efflux transporter (TC 2.A.44family) |
| YP_001466175.1| hydrogenase maturation | fig|6666666.462.peg.898;Name=Coenzyme F420 hydrogenase maturationprotease (EC3.4.24.-);Ontology_term=KEGG_ENZYME:3.4.24.- |
| YP_001466174.1| hypothetical protein | fig|6666666.462.peg.899;Name=hypothetical protein |
| YP_001466173.1| hydrogenase-4 component I | fig|6666666.462.peg.900;Name=Formate hydrogenlyase subunit 7 |
| YP_001466172.1| formate hydrogenlyase complex | fig|6666666.462.peg.901;Name=Formate hydrogenlyase complex 3 iron-sulfurprotein%3B Formate hydrogenlyase subunit 6%3BNi2CFe-hydrogenase III medium subunit |
| YP_001466171.1| DNA-3-methyladenine | fig|6666666.462.peg.902;Name=Hydrogenase-4 component G (EC1.-.-.-);Ontology_term=KEGG_ENZYME:1.-.-.- |
| YP_001466170.1| hypothetical protein | fig|6666666.462.peg.903;Name=Hydrogenase-4 component F (EC1.-.-.-);Ontology_term=KEGG_ENZYME:1.-.-.- |
| YP_001466169.1| hydrogenase 4 membrane subunit | fig|6666666.462.peg.904;Name=Hydrogenase-4 component E (EC1.-.-.-);Ontology_term=KEGG_ENZYME:1.-.-.- |
| YP_001466168.1| hypothetical protein | fig|6666666.462.peg.905;Name=Formate hydrogenlyase subunit 4 |
| YP_001466167.1| L-alanyl-D-glutamate peptidase | fig|6666666.462.peg.906;Name=Hydrogenase-4 component B (EC 1.-.-.-) /Formate hydrogenlyase subunit3;Ontology_term=KEGG_ENZYME:1.-.-.- |
| YP_001466166.1| 4Fe-4S ferredoxin iron-sulfur | fig|6666666.462.peg.907;Name=Hydrogenase-4 component A (EC1.-.-.-);Ontology_term=KEGG_ENZYME:1.-.-.- |
| YP_001466165.1| putative lipoprotein | fig|6666666.462.peg.908;Name=Outer membrane protein romA |
| YP_001466164.1| hypothetical protein | fig|6666666.462.peg.909;Name=hypothetical protein |
| YP_001466163.1| hypothetical protein | fig|6666666.462.peg.910;Name=hypothetical protein |
| YP_001466161.1| cytochrome c subfamily protein | fig|6666666.462.peg.913;Name=ubiquinol cytochrome C oxidoreductase2Ccytochrome C1 subunit |
| YP_001466160.1| cytochrome B(N-)/b6/PetB | fig|6666666.462.peg.914;Name=Ubiquinol--cytochrome c reductase2Ccytochrome B subunit (EC1.10.2.2);Ontology_term=KEGG_ENZYME:1.10.2.2 |
| YP_001466159.1| ubiquinol-cytochrome c | fig|6666666.462.peg.915;Name=Ubiquinol-cytochrome C reductase iron-sulfursubunit (EC 1.10.2.2);Ontology_term=KEGG_ENZYME:1.10.2.2 |
| YP_001466158.1| tRNA uridine | fig|6666666.462.peg.916;Name=tRNA uridine 5-carboxymethylaminomethylmodification enzyme GidA |
| YP_001466157.1| | fig|6666666.462.peg .917;Name=Putative 2-acylglycerophosphoethanolamine acyltransferase / acyl-acyl carrier protein synthetase (EC 6.2.1.20);Ontology_term=KEGG_ENZYME:6.2.1.20 |
| YP_001466156.1| glutamine-binding periplasmic | fig|6666666.462.peg.918;Name=hypothetical protein |
| YP_001466155.1| solute-binding family 1 | fig|6666666.462.peg.919;Name=amino acid ABC transporter2C periplasmicamino acid-binding protein |
| YP_001466154.1| solute-binding family 1 | fig|6666666.462.peg.920;Name=hypothetical protein |
| YP_001466153.1| glutamine-binding periplasmic | fig|6666666.462.peg.921;Name=amino acid ABC transporter2C permeaseprotein |
| YP_001466152.1| putative ABC transporter, | fig|6666666.462.peg.922;Name=Cell division transporter2C ATP-bindingprotein FtsE (TC 3.A.5.1.1) |
| YP_001466151.1| flavodoxin FldA [Campylobacter | fig|6666666.462.peg.923;Name=Flavodoxin 1 |
| YP_001466150.1| | fig|6666666.462.peg.924;Name=hypothetical protein |
| YP_001466149.1| hypothetical protein | fig|6666666.462.peg.925;Name=hypothetical protein |
| YP_001466148.1| sugar efflux transporter | fig|6666666.462.peg.926;Name=Probable sugar efflux transporter |
| YP_001466147.1| pyruvate:ferredoxin | fig|6666666.462.peg .927;Name=Pyruvate-flavodoxin oxidoreductase (EC 1.2.7.-);Ontology_term=KEGG_ENZYME:1.2.7.- |
| YP_001466145.1| Mug G:T/U mismatch-specific | fig|6666666.462.peg.929;Name=G:T/U mismatch-specific uracil/thymineDNA-glycosylase |
| YP_001466112.1| TPR repeat-containing protein | fig|6666666.462.peg.1296;Name=Paralysed flagella protein PflA |
| YP_001466111.1| NADH dehydrogenase subunit N | fig|6666666.462.peg.1295;Name=NADH-ubiquinone oxidoreductase chain N (EC1.6.5.3);Ontology_term=KEGG_ENZYME:1.6.5.3 |
| YP_001466110.1| NADH dehydrogenase subunit M | fig|6666666.462.peg.1294;Name=NADH-ubiquinone oxidoreductase chain M (EC1.6.5.3);Ontology_term=KEGG_ENZYME:1.6.5.3 |
| YP_001466109.1| NADH dehydrogenase subunit L | fig|6666666.462.peg.1293;Name=NADH-ubiquinone oxidoreductase chain L (EC1.6.5.3);Ontology_term=KEGG_ENZYME:1.6.5.3 |
| YP_001466108.1| NADH dehydrogenase subunit I | fig|6666666.462.peg.1290;Name=NADH-ubiquinone oxidoreductase chain I (EC1.6.5.3);Ontology_term=KEGG_ENZYME:1.6.5.3 |
| YP_001466107.1| NADH dehydrogenase subunit H | fig|6666666.462.peg.1289;Name=NADH-ubiquinone oxidoreductase chain H (EC1.6.5.3);Ontology_term=KEGG_ENZYME:1.6.5.3 |
| YP_001466106.1| NADH dehydrogenase subunit G | fig|6666666.462.peg.1288;Name=NADH-ubiquinone oxidoreductase chain G (EC1.6.5.3);Ontology_term=KEGG_ENZYME:1.6.5.3 |
| YP_001466105.1| hypothetical protein | fig|6666666.462.peg.1287;Name=hypothetical protein |
| YP_001466104.1| hypothetical protein | fig|6666666.462.peg.1286;Name=NADH-ubiquinone oxidoreductase chain E(EC 1.6.5.3);Ontology_term=KEGG_ENZYME:1.6.5.3 |
| YP_001466103.1| NADH dehydrogenase subunit B | fig|6666666.462.peg.1283;Name=NADH-ubiquinone oxidoreductase chain B (EC1.6.5.3);Ontology_term=KEGG_ENZYME:1.6.5.3 |
| YP_001466102.1| NADH dehydrogenase subunit A | fig|6666666.462.peg.1282;Name=NADH ubiquinone oxidoreductase chain A (EC1.6.5.3);Ontology_term=KEGG_ENZYME:1.6.5.3 |
| YP_001466101.1| ABC transporter ATP-binding | fig|6666666.462.peg.1281;Name=pyoverdine ABC export system2Cpermease/ATP-binding protein2C putative |
| YP_001466100.1| coproporphyrinogen III oxidase | fig|6666666.462.peg.1280;Name=Oxygen-independent coproporphyrinogen IIIoxidase2C putative |
| YP_001466099.1| hypothetical protein | fig|6666666.462.peg.1279;Name=hypothetical protein |
| YP_001466097.1| nucleotide-binding protein | fig|6666666.462.peg.1276;Name=UPF0234 protein YajQ |
| YP_001466096.1| D-isomer specific | fig|6666666.462.peg.1275;Name=Putative D-2-hydroxyacid dehydrogenase |
| YP_001466095.1| glutathionylspermidine | fig|6666666.462.peg.1274;Name=glutathionylspermidine synthetase/amidase |
| YP_001466094.1| hypothetical protein | fig|6666666.462.peg.1273;Name=Putative flagellar motility protein |
| YP_001466093.1| putative phage integrase | fig|6666666.462.peg.1272;Name=hypothetical protein |
| YP_001466091.1| FAD binding domain-containing | fig|6666666.462.peg.1270;Name=Flavocytochrome c flavin subunit |
| YP_001466090.1| cytochrome c oxidase accessory | fig|6666666.462.peg.1269;Name=Ferredoxin domain-containing integralmembrane protein |
| YP_001466089.1| transcriptional regulator, | fig|6666666.462.peg.1268;Name=Transcriptional repressor of CmeABCoperon2C CmeR |
| YP_001466088.1| CmeA [Campylobacter concisus | fig|6666666.462.peg.1267;Name=RND efflux system2C membrane fusionprotein CmeA |
| YP_001466087.1| multidrug resistance protein | fig|6666666.462.pe g.1266;Name=RND efflux system2C inner membrane transporter CmeB |
| YP_001466086.1| 30S ribosomal protein S12 | fig|6666666.462.peg.1265;Name=RND efflux system2C outer membranelipoprotein CmeC |
| YP_001466085.1| isoaspartyl dipeptidase | fig|6666666.462.peg.1264;Name=hypothetical protein |
| YP_001466084.1| Na+/H+ antiporter NhaC | fig|6666666.462.peg.1263;Name=Aquaporin Z |
| YP_001466052.1| NifS family cysteine | fig|6666666.462.peg.1262;Name=Cysteine desulfurase (EC2.8.1.7);Ontology_term=KEGG_ENZYME:2.8.1.7 |
| YP_001466051.1| acetolactate synthase small | fig|6666666.462.peg.1261;Name=Iron-sulfur cluster assembly scaffoldprotein IscU/NifU-like |
| YP_001466050.1| tRNA | fig|6666666.462.peg.1260;Name=tRNA delta(2)-isopentenylpyrophosphatetransferase (EC2.5.1.8);Ontology_term=KEGG_ENZYME:2.5.1.8 |
| YP_001466049.1| putative FAD dependent | fig|6666666.462.peg.1259;Name=D-amino acid dehydrogenase small subunit(EC 1.4.99.1);Ontology_term=KEGG_ENZYME:1.4.99.1 |
| YP_001466048.1| prenyltransferase | fig|6666666.462.peg.1258;Name=4-hydroxybenzoate polyprenyltransferase(EC 2.5.1.-);Ontology_term=KEGG_ENZYME:2.5.1.- |
| YP_001466047.1| hypothetical protein | fig|6666666.462.peg.1257;Name=hypothetical protein |
| YP_001466046.1| hypothetical protein | fig|6666666.462.peg.1256;Name=Putative periplasmic protein |
| YP_001466045.1| molybdenum cofactor | fig|6666666.462.peg.1255;Name=Molybdenum cofactor biosynthesis proteinMoaA |
| YP_001466044.1| | fig|6666666.462.peg.1254;Name=Queuosine Biosynthesis QueE Radical SAM |
| YP_001466043.1| putative 6-pyruvoyl | fig|6666666.462.peg.1253;Name=Queuosine biosynthesis QueD2C PTPS-I |
| YP_001466042.1| hypothetical protein | fig|6666666.462.peg.1251;Name=membrane protein |
| YP_001466041.1| hypothetical protein | fig|6666666.462.peg.1250;Name=Ribosomal RNA small subunitmethyltransferase E (EC2.1.1.-);Ontology_term=KEGG_ENZYME:2.1.1.- |
| YP_001466040.1| 50S ribosomal protein L31 | fig|6666666.462.peg.1249;Name=LSU ribosomal protein L31p |
| YP_001466039.1| hypothetical protein | fig|6666666.462.peg.1248;Name=rRNA small subunit methyltransferase I |
| YP_001466038.1| RNA methyltransferase | fig|6666666.462.peg.1247;Name=rRNA methylases |
| YP_001466037.1| phosphatidylglycerophosphate | fig|6666666.462.peg.1246;Name=membrane protein |
| YP_001466036.1| hypothetical protein | fig|6666666.462.peg.1245;Name=Putative periplasmic protein |
| YP_001466035.1| aspartate aminotransferase | fig|6666666.462.peg.1244;Name=Aspartate aminotransferase (EC2.6.1.1);Ontology_term=KEGG_ENZYME:2.6.1.1 |
| YP_001466034.1| homoserine dehydrogenase | fig|6666666.462.peg.1243;Name=Homoserine dehydrogenase (EC1.1.1.3);Ontology_term=KEGG_ENZYME:1.1.1.3 |
| YP_001466033.1| hypothetical protein | fig|6666666.462.peg.1242;Name=Predicted endonuclease distantly relatedto archaeal Holliday junction resolvase |
| YP_001466032.1| thioredoxin [Campylobacter | fig|6666666.462.peg.1241;Name=Thioredoxin |
| YP_001466031.1| hypothetical protein | fig|6666666.462.peg.1240;Name=hypothetical protein |
| YP_001466030.1| thioredoxin-disulfide | fig|6666666.462.peg.1239;Name=Thioredoxin reductase (EC1.8.1.9);Ontology_term=KEGG_ENZYME:1.8.1.9 |
| YP_001466028.1| dihydrodipicolinate reductase | fig|6666666.462.peg.1238;Name=Dihydrodipicolinate reductase (EC1.3.1.26);Ontology_term=KEGG_ENZYME:1.3.1.26 |
| YP_001466027.1| amidophosphoribosyltransferase | fig|6666666.462.peg.1237;Name=Amidophosphoribosyltransferase (EC2.4.2.14);Ontology_term=KEGG_ENZYME:2.4.2.14 |
| YP_001466026.1| hypothetical protein | fig|6666666.462.peg.1236;Name=hypothetical protein |
| YP_001466024.1| UDP-MurNac-pentapeptide | fig|6666666.462.peg.1234;Name=hypothetical protein |
| YP_001466023.1| putative | fig|6666666.462.peg.1233;Name=hypothetical protein |
| YP_001466022.1| mate efflux family protein | fig|6666666.462.peg.1232;Name=hypothetical protein |
| YP_001466021.1| curli production | fig|6666666.462.peg.1231;Name=Probable lipoprotein |
| YP_001466020.1| integrase/recombinase | fig|6666666.462.peg.1230;Name=hypothetical protein |
| YP_001466019.1| lipoprotein [Campylobacter | fig|6666666.462.peg.1229;Name=Putative lipoprotein |
| YP_001466018.1| phosphate acetyltransferase | fig|6666666.462.peg.1530;Name=Permease of the drug/metabolite transporter(DMT) superfamily |
| YP_001466017.1| hypothetical protein | fig|6666666.462.peg.1531;Name=Sua5 YciO YrdC YwlC family protein |
| YP_001466014.1| GTP-binding protein TypA/BipA | fig|6666666.462.peg.1533;Name=GTP-binding protein TypA/BipA |
| YP_001466013.1| quinone-reactive Ni/Fe | fig|6666666.462.peg.1534;Name=Flagellar hook-length control protein FliK |
| YP_001466012.1| flagellar basal body rod | fig|6666666.462.peg.1535;Name=Flagellar basal-body rod modification proteinFlgD |
| YP_001466011.1| putative cytochrome c-type | fig|6666666.462.peg.1537;Name=Functional role page for Cytochrome c-typeprotein TorY |
| YP_001466010.1| hypothetical protein | fig|6666666.462.peg.1538;Name=Trimethylamine-N-oxide reductase (EC1.6.6.9);Ontology_term=KEGG_ENZYME:1.6.6.9 |
| YP_001466001.1| hypothetical protein | fig|6666666.462.peg.1546;Name=Putative lipoprotein |
| YP_001466000.1| outer membrane protein | fig|6666666.462.peg.1548;Name=Outer membrane protein |
| YP_001465999.1| acyl-CoA synthetase | fig|6666666.462.peg.1549;Name=Long-chain-fatty-acid--CoA ligase (EC6.2.1.3);Ontology_term=KEGG_ENZYME:6.2.1.3 |
| YP_001465998.1| cysteine synthase A | fig|6666666.462.peg.1550;Name=Probable AAA family ATPase |
| YP_001465995.1| flagellum-specific ATP | fig|6666666.462.peg.1555;Name=Flagellum-specific ATP synthase FliI |
| YP_001465994.1| GTP cyclohydrolase I | fig|6666666.462.peg.1556;Name=GTP cyclohydrolase I (EC 3.5.4.16) type1;Ontology_term=KEGG_ENZYME:3.5.4.16 |
| YP_001465992.1| trigger factor [Campylobacter | fig|6666666.462.peg.1557;Name=Cell division trigger factor (EC5.2.1.8);Ontology_term=KEGG_ENZYME:5.2.1.8 |
| YP_001465991.1| ATP-dependent Clp protease | fig|6666666.462.peg.1558;Name=ATP-dependent Clp protease proteolyticsubunit (EC3.4.21.92);Ontology_term=KEGG_ENZYME:3.4.21.92 |
| YP_001465989.1| peptide deformylase | fig|6666666.462.peg.1560;Name=Peptide deformylase (EC3.5.1.88);Ontology_term=KEGG_ENZYME:3.5.1.88 |
| YP_001465988.1| putative Mg chelatase-like | fig|6666666.462.peg.1561;Name=MG(2+) CHELATASE FAMILY PROTEIN /ComM-related protein |
| YP_001465987.1| preprotein translocase, YajC | fig|6666666.462.peg.1562;Name=YjeF protein2C function unknown |
| YP_001465986.1| phosphoribosylglycinamide | fig|6666666.462.peg.1563;Name=Phosphoribosylglycinamide formyltransferase(EC 2.1.2.2);Ontology_term=KEGG_ENZYME:2.1.2.2 |
| YP_001465985.1| S-adenosylmethionine | fig|6666666.462.peg.1564;Name=Integral membrane protein TerC |
| YP_001465984.1| hypothetical protein | fig|6666666.462.peg.1566;Name=Multi antimicrobial extrusion protein(Na(+)/drug antiporter)2C MATE family of MDR effluxpumps |
| YP_001465983.1| oligoendopeptidase F | fig|6666666.462.peg.1567;Name=Zinc ABC transporter2C inner membranepermease protein ZnuB |
| YP_001465982.1| ATP-dependent DNA helicase | fig|6666666.462.peg.1568;Name=Zinc ABC transporter2C ATP-binding proteinZnuC |
| YP_001465981.1| tRNA pseudouridine synthase B | fig|6666666.462.peg.1569;Name=membrane protein2C putative |
| YP_001465980.1| putative carbon storage | fig|6666666.462.peg.1570;Name=Zinc ABC transporter2C periplasmic-bindingprotein ZnuA |
| YP_001465979.1| SsrA-binding protein | fig|6666666.462.peg.1571;Name=Peroxide stress regulator / Ferric uptakeregulation protein |
| YP_001465978.1| | fig|6666666.462.peg.1572;Name=hypothetical protein |
| YP_001465977.1| flagellar basal-body rod | fig|6666666.462.peg.1573;Name=Hypothetical protein Cj0261c |
| YP_001465976.1| permeases of the | fig|6666666.462.peg.1574;Name=Permease of the drug/metabolite transporter(DMT) superfamily |
| YP_001465975.1| hypothetical protein | fig|6666666.462.peg.1575;Name=hypothetical protein |
| YP_001465974.1| cell division protein | fig|6666666.462.peg.1576;Name=membrane protein |
| YP_001465973.1| exodeoxyribonuclease III | fig|6666666.462.peg.1577;Name=Exodeoxyribonuclease III (EC3.1.11.2);Ontology_term=KEGG_ENZYME:3.1.11.2 |
| YP_001465972.1| diacylglycerol kinase (dagk; | fig|6666666.462.peg.1578;Name=Diacylglycerol kinase (EC2.7.1.107);Ontology_term=KEGG_ENZYME:2.7.1.107 |
| YP_001465971.1| hypothetical protein | fig|6666666.462.peg.1579;Name=Putative helix-turn-helix motif protein |
| YP_001465970.1| FAD binding domain-containing | fig|6666666.462.peg.1580;Name=Flavocytochrome c flavin subunit |
| YP_001465969.1| ModE family transcriptional | fig|6666666.462.peg.1581;Name=hypothetical protein |
| YP_001465968.1| peptide chain release factor 1 | fig|6666666.462.peg.1582;Name=Peptide chain release factor 1 |
| YP_001465967.1| 30S ribosomal protein S20 | fig|6666666.462.peg.1583;Name=SSU ribosomal protein S20p |
| YP_001465966.1| phosphoglucosamine mutase | fig|6666666.462.peg.1584;Name=Phosphoglucosamine mutase (EC 5.4.2.10) /Phosphomannomutase (EC5.4.2.8);Ontology_term=KEGG_ENZYME:5.4.2.10,KEGG_ENZYME:5.4.2.8 |
| YP_001465965.1| lipoprotein signal peptidase | fig|6666666.462.peg.1585;Name=Lipoprotein signal peptidase (EC3.4.23.36);Ontology_term=KEGG_ENZYME:3.4.23.36 |
| YP_001465964.1| TM2 [Campylobacter concisus | fig|6666666.462.peg.1586;Name=hypothetical protein |
| YP_001465963.1| hypothetical protein | fig|6666666.462.peg.1588;Name=hypothetical protein |
| YP_001465962.1| acetyl-CoA carboxylase | fig|6666666.462.peg.1589;Name=Pyruvate carboxyl transferase subunit A (EC6.4.1.1);Ontology_term=KEGG_ENZYME:6.4.1.1 |
| YP_001465961.1| PQQ repeat-containing protein | fig|6666666.462.peg.1590;Name=hypothetical protein |
| YP_001465960.1| | fig|6666666.462.peg.1591;Name=hypothetical protein |
| YP_001465959.1| glutamate dehydrogenase | fig|6666666.462.peg.1592;Name=NADP-specific glutamate dehydrogenase (EC1.4.1.4);Ontology_term=KEGG_ENZYME:1.4.1.4 |
| YP_001465956.1| translation initiation factor | fig|6666666.462.peg.1594;Name=Translation initiation factor 3 |
| YP_001465955.1| threonyl-tRNA synthetase | fig|6666666.462.peg.1595;Name=Threonyl-tRNA synthetase (EC6.1.1.3);Ontology_term=KEGG_ENZYME:6.1.1.3 |
| YP_001465954.1| hypothetical protein | fig|6666666.462.peg.1596;Name=Multicopper oxidase |
| YP_001465953.1| YbaK/prolyl-tRNA synthetases | fig|6666666.462.peg.1597;Name=hypothetical protein |
| YP_001465952.1| uracil-DNA glycosylase | fig|6666666.462.peg.1598;Name=Uracil-DNA glycosylase2C family 1 |
| YP_001465951.1| hypothetical protein | fig|6666666.462.peg.1599;Name=hypothetical protein |
| YP_001465950.1| hypothetical protein | fig|6666666.462.peg.1600;Name=5-nitroimidazole antibiotic resistanceprotein |
| YP_001465949.1| recombination factor protein | fig|6666666.462.peg.1601;Name=Helicase-like protein |
| YP_001465948.1| hypothetical protein | fig|6666666.462.peg.1602;Name=Holliday junction DNA helicase RuvA |
| YP_001465947.1| branched-chain amino acid | fig|6666666.462.peg.1603;Name=Na(+)-linked D-alanine glycine permease |
| YP_001465946.1| flavocytochrome c heme subunit | fig|6666666.462.peg.1605;Name=Flavocytochrome c heme subunit |
| YP_001465945.1| NapC/NirT cytochrome c family, | fig|6666666.462.peg.1606;Name=Functional role page for Cytochrome c-typeprotein TorY |
| YP_001465944.1| putative DNA-binding protein | fig|6666666.462.peg.1609;Name=52C10-methylenetetrahydrofolate reductase(EC 1.5.1.20);Ontology_term=KEGG_ENZYME:1.5.1.20 |
| YP_001465940.1| S-layer-RTX protein | fig|6666666.462.peg.1613;Name=Probable dipeptidase (EC3.4.-.-);Ontology_term=KEGG_ENZYME:3.4.-.- |
| YP_001465939.1| 50S ribosomal protein L35 | fig|6666666.462.peg.1614;Name=LSU ribosomal protein L35p |
| YP_001465938.1| 50S ribosomal protein L20 | fig|6666666.462.peg.1615;Name=LSU ribosomal protein L20p |
| YP_001465937.1| diaminopimelate epimerase | fig|6666666.462.peg.1616;Name=Diaminopimelate epimerase (EC5.1.1.7);Ontology_term=KEGG_ENZYME:5.1.1.7 |
| YP_001465936.1| phosphomethylpyrimidine kinase | fig|6666666.462.peg.1617;Name=hypothetical protein |
| YP_001465935.1| dephospho-CoA kinase | fig|6666666.462.peg.1618;Name=Dephospho-CoA kinase (EC2.7.1.24);Ontology_term=KEGG_ENZYME:2.7.1.24 |
| YP_001465934.1| phosphoribosylaminoimidazole | fig|6666666.462.peg.1619;Name=Phosphoribosylformylglycinamidinecyclo-ligase (EC6.3.3.1);Ontology_term=KEGG_ENZYME:6.3.3.1 |
| YP_001465932.1| RDD family protein | fig|6666666.462.peg.1621;Name=Putative integral membrane protein |
| YP_001465931.1| hypothetical protein | fig|6666666.462.peg.1623;Name=hypothetical protein |
| YP_001465930.1| hypothetical protein | fig|6666666.462.peg.1624;Name=Hydrolase (HAD superfamily) |
| YP_001465929.1| response regulator | fig|6666666.462.peg.1625;Name=NADPH dependent preQ0 reductase |
| YP_001465928.1| DNA gyrase subunit B | fig|6666666.462.peg.1626;Name=DNA gyrase subunit B (EC5.99.1.3);Ontology_term=KEGG_ENZYME:5.99.1.3 |
| YP_001465927.1| DNA polymerase III subunit | fig|6666666.462.peg.500;Name=DNA polymerase III beta subunit (EC2.7.7.7);Ontology_term=KEGG_ENZYME:2.7.7.7 |
| YP_001465925.1| chromosomal replication | fig|6666666.462.peg.499;Name=Chromosomal replication initiator proteinDnaA |
| ABW74835.1| putative lipoprotein [Campylobacter | fig|6666666.462.peg.1765;Name=hypothetical protein |
| ABW74834.1| mechanosensitive ion channel family | fig|6666666.462.peg.849;Name=membrane protein |
| ABW74833.1| iron-sulfur protein [Campylobacter | fig|6666666.462.peg.791;Name=Ferredoxin-type protein NapG (periplasmicnitrate reductase) |
| ABW74830.1| GntT protein [Campylobacter | fig|6666666.462.peg.172;Name=D-glycerate transporter (predicted) |
| ABW74827.1| conserved domain protein | fig|6666666.462.peg.762;Name=Possible molybdopterin convertingfactor2C subunit 1 |
| ABW74823.1| hydrolase, carbon-nitrogen family | fig|6666666.462.peg.1824;Name=Predicted amidohydrolase |
| ABW74821.1| UDP-N-acetylmuramate--alanine | fig|6666666.462.peg.1821;Name=UDP-N-acetylmuramate--alanine ligase (EC6.3.2.8);Ontology_term=KEGG_ENZYME:6.3.2.8 |
| ABW74814.1| protein YieJ [Campylobacter | fig|6666666.462.peg.721;Name=hypothetical protein |
| ABW74812.1| oxidoreductase, FAD/FMN-binding | fig|6666666.462.peg.157;Name=hypothetical protein |
| ABW74810.1| hypothetical protein CCC13826_0379 | fig|6666666.462.peg.1702;Name=hypothetical protein |
| ABW74808.1| conserved hypothetical protein | fig|6666666.462.peg.1854;Name=Putative Holliday junction resolvase (EC3.1.-.-);Ontology_term=KEGG_ENZYME:3.1.-.- |
| ABW74806.1| twin arginine-targeting protein | fig|6666666.462.peg.1857;Name=Twin-arginine translocation protein TatC |
| ABW74805.1| putative oxygen-independent | fig|6666666.462.peg.1860;Name=Similar to coproporphyrinogen III oxidase2Coxygen-independent (EC1.3.99.22);Ontology_term=KEGG_ENZYME:1.3.99.22 |
| ABW74804.1| putative transcriptional regulator, | fig|6666666.462.peg.1861;Name=Transcriptional regulator |
| ABW74803.1| TonB-dependent receptor | fig|6666666.462.peg.1862;Name=TonB-dependent receptor%3B Outer membranereceptor for ferrienterochelin and colicins |
| ABW74802.1| NAD+ synthetase [Campylobacter | fig|6666666.462.peg.1379;Name=NAD synthetase (EC6.3.1.5);Ontology_term=KEGG_ENZYME:6.3.1.5 |
| ABW74798.1| branched-chain amino acid transport | fig|6666666.462.peg.1055;Name=Branched-chain amino acid transportprotein azlC |
| ABW74797.1| peptidase U32 [Campylobacter | fig|6666666.462.peg.956;Name=Predicted protease from collagenase family |
| ABW74796.1| phosphoribosylaminoimidazole | fig|6666666.462.peg.955;Name=Phosphoribosylaminoimidazole carboxylasecatalytic subunit (EC4.1.1.21);Ontology_term=KEGG_ENZYME:4.1.1.21 |
| ABW74795.1| integral membrane protein MviN | fig|6666666.462.peg.1076;Name=Proposed peptidoglycan lipid II flippaseMurJ |
| ABW74794.1| conserved hypothetical protein | fig|6666666.462.peg.1075;Name=hypothetical protein |
| ABW74792.1| alpha/beta hydrolase fold | fig|6666666.462.peg.1071;Name=2-hydroxy-6-oxohepta-22C4-dienoate hydrolase |
| ABW74791.1| Ni/Fe-hydrogenase, B-type | fig|6666666.462.peg.592;Name=Quinone-reactive Ni/Fe hydrogenase2Ccytochrome b subunit |
| ABW74788.1| DedA family protein [Campylobacter | fig|6666666.462.peg.372;Name=putative integral membrane protein (dedAhomolog) |
| ABW74784.1| flagellar hook-associated protein | fig|6666666.462.peg.1092;Name=Flagellar hook-associated protein FlgL |
| ABW74783.1| conserved hypothetical protein | fig|6666666.462.peg.1091;Name=UPF0246 protein YaaA |
| ABW74782.1| ribulose-phosphate 3-epimerase | fig|6666666.462.peg.1424;Name=Ribulose-phosphate 3-epimerase (EC5.1.3.1);Ontology_term=KEGG_ENZYME:5.1.3.1 |
| ABW74780.1| glycerol-3-phosphate dehydrogenase | fig|6666666.462.peg.1184;Name=Glycerol-3-phosphate dehydrogenase[NAD(P)+] (EC1.1.1.94);Ontology_term=KEGG_ENZYME:1.1.1.94 |
| ABW74779.1| aspartyl/glutamyl-tRNA(Asn/Gln) | fig|6666666.462.peg.1185;Name=Aspartyl-tRNA(Asn) amidotransferasesubunit B (EC 6.3.5.6) @ Glutamyl-tRNA(Gln)amidotransferase subunit B (EC6.3.5.7);Ontology_term=KEGG_ENZYME:6.3.5.6,KEGG_ENZYME:6.3.5.7 |
| ABW74777.1| bifunctional short chain isoprenyl | fig|6666666.462.peg.257;Name=Octaprenyl-diphosphate synthase (EC 2.5.1.-)/ Dimethylallyltransferase (EC 2.5.1.1) /Geranyltranstransferase (farnesyldiphosphate synthase)(EC 2.5.1.10) / Geranylgeranyl pyrophosphate synthetase(EC2.5.1.29);Ontology_term=KEGG_ENZYME:2.5.1.-,KEGG_ENZYME:2.5.1.1,KEGG_ENZYME:2.5.1.10,KEGG_ENZYME:2.5.1.29 |
| ABW74773.1| bifunctional methyltransferase | fig|6666666.462.peg.312;Name=HemK2C Methylase of polypeptide chainrelease factors |
| ABW74772.1| ferrous-iron efflux pump FieF | fig|6666666.462.peg.329;Name=Cobalt-zinc-cadmium resistance protein |
| ABW74771.1| ComEC/Rec2 family protein | fig|6666666.462.peg.1658;Name=Competence protein |
| ABW74769.1| putative flavoprotein | fig|6666666.462.peg.1646;Name=hypothetical protein |
| ABW74762.1| periplasmic nitrate reductase | fig|6666666.462.peg.29;Name=Periplasmic nitrate reductase component NapD |
| ABW74760.1| aspartate-semialdehyde | fig|6666666.462.peg.34;Name=Aspartate-semialdehyde dehydrogenase (EC1.2.1.11);Ontology_term=KEGG_ENZYME:1.2.1.11 |
| ABW74759.1| protein of unknown function | fig|6666666.462.peg.37;Name=Putative lipoprotein required for motility |
| ABW74758.1| chemotaxis protein | fig|6666666.462.peg.39;Name=Chemotaxis protein methyltransferase CheR(EC 2.1.1.80);Ontology_term=KEGG_ENZYME:2.1.1.80 |
| ABW74756.1| ubiquinone/menaquinone biosynthesis | fig|6666666.462.peg.65;Name=Ubiquinone/menaquinone biosynthesismethyltransferase UbiE (EC2.1.1.-);Ontology_term=KEGG_ENZYME:2.1.1.- |
| ABW74755.1| chad domain family [Campylobacter | fig|6666666.462.peg.66;Name=hypothetical protein |
| ABW74754.1| transcriptional regulator, Fur | fig|6666666.462.peg.68;Name=Peroxide stress regulator%3B Ferric uptakeregulation protein%3B Fe2+/Zn2+ uptake regulationproteins |
| ABW74753.1| purine-binding chemotaxis protein | fig|6666666.462.peg.82;Name=Positive regulator of CheA protein activity(CheW) |
| ABW74751.1| phenylalanyl-tRNA synthetase, beta | fig|6666666.462.peg.1447;Name=Phenylalanyl-tRNA synthetase beta chain (EC6.1.1.20);Ontology_term=KEGG_ENZYME:6.1.1.20 |
| ABW74750.1| histidine triad nucleotide-binding | fig|6666666.462.peg.1449;Name=HIT-family protein |
| ABW74749.1| putative lipoprotein [Campylobacter | fig|6666666.462.peg.213;Name=BarA sensory histidine kinase ( VarS GacS) |
| ABW74748.1| methionyl-tRNA synthetase | fig|6666666.462.peg.226;Name=Methionyl-tRNA synthetase (EC6.1.1.10);Ontology_term=KEGG_ENZYME:6.1.1.10 |
| ABW74747.1| conserved hypothetical protein | fig|6666666.462.peg.227;Name=hypothetical protein |
| ABW74746.1| protein of unknown function , | fig|6666666.462.peg.1468;Name=nodulin 21-related protein |
| ABW74743.1| DNA polymerase I (POL I) | fig|6666666.462.peg.1127;Name=DNA polymerase I (EC2.7.7.7);Ontology_term=KEGG_ENZYME:2.7.7.7 |
| ABW74741.1| DcuC protein [Campylobacter | fig|6666666.462.peg.1525;Name=C4-dicarboxylate anaerobic carrier2Cputative |
| ABW74740.1| N-carbamoyl-L-amino acid hydrolase | fig|6666666.462.peg.1522;Name=N-carbamoyl-L-amino acid hydrolase (EC3.5.1.87);Ontology_term=KEGG_ENZYME:3.5.1.87 |
| ABW74736.1| umuc domain protein dna-repair | fig|6666666.462.peg.1227;Name=ImpB/MucB/SamB family protein |
| ABW74735.1| hypothetical protein CCC13826_0787 | fig|6666666.462.peg.1228;Name=hypothetical protein |
| ABW74731.1| cytochrome c oxidase, Cbb3-type, | fig|6666666.462.peg.890;Name=Cytochrome c oxidase subunit CcoN (EC1.9.3.1);Ontology_term=KEGG_ENZYME:1.9.3.1 |
| ABW74730.1| carbamoyl-phosphate synthase, small | fig|6666666.462.peg.894;Name=Carbamoyl-phosphate synthase small chain (EC6.3.5.5);Ontology_term=KEGG_ENZYME:6.3.5.5 |
| ABW74727.1| NADH-ubiquinone/plastoquinone | fig|6666666.462.peg.1291;Name=NADH-ubiquinone oxidoreductase chain J (EC1.6.5.3);Ontology_term=KEGG_ENZYME:1.6.5.3 |
| ABW74722.1| putative lipoprotein [Campylobacter | fig|6666666.462.peg.1252;Name=hypothetical protein |
| ABW74721.1| flagellar hook protein | fig|6666666.462.peg.1536;Name=Flagellar hook protein FlgE |
| ABW74719.1| transmembrane protein | fig|6666666.462.peg.1587;Name=Hypothetical membrane protein2C possibleinvolvement in cytochrome functioning/assembly |
| ABA54534.1| RpoB [Campylobacter concisus] | fig|6666666.462.peg.622;Name=DNA-directed RNA polymerase beta subunit(EC 2.7.7.6);Ontology_term=KEGG_ENZYME:2.7.7.6 |
| A8Z6P9.1|LPXA_CAMC1 RecName: | fig|6666666.462.peg.201;Name=Acyl-[acyl-carrier-protein]--UDP-N-acetylglucosamine O-acyltransferase (EC2.3.1.129);Ontology_term=KEGG_ENZYME:2.3.1.129 |
| A8Z6P6.1|SYI_CAMC1 RecName: Full=Isoleucyl-tRNA | fig|6666666.462.peg.1829;Name=Isoleucyl-tRNA synthetase (EC6.1.1.5);Ontology_term=KEGG_ENZYME:6.1.1.5 |
| A8Z6P5.1|GATA_CAMC1 RecName: | fig|6666666.462.peg.1828;Name=Aspartyl-tRNA(Asn) amidotransferase subunitA (EC 6.3.5.6) @ Glutamyl-tRNA(Gln) amidotransferasesubunit A (EC6.3.5.7);Ontology_term=KEGG_ENZYME:6.3.5.6,KEGG_ENZYME:6.3.5.7 |
| A8Z6N6.1|MRAY_CAMC1 RecName: | fig|6666666.462.peg.715;Name=Phospho-N-acetylmuramoyl-pentapeptide-transferase (EC2.7.8.13);Ontology_term=KEGG_ENZYME:2.7.8.13 |
| A8Z6M6.1|QUEA_CAMC1 RecName: | fig|6666666.462.peg.1856;Name=S-adenosylmethionine:tRNAribosyltransferase-isomerase (EC5.-.-.-);Ontology_term=KEGG_ENZYME:5.-.-.- |
| A8Z6M1.1|LPXK_CAMC1 RecName: | fig|6666666.462.peg.1377;Name=Tetraacyldisaccharide 4'-kinase (EC2.7.1.130);Ontology_term=KEGG_ENZYME:2.7.1.130 |
| A8Z6L3.1|DDL_CAMC1 RecName: | fig|6666666.462.peg.1073;Name=D-alanine--D-alanine ligase (EC6.3.2.4);Ontology_term=KEGG_ENZYME:6.3.2.4 |
| A8Z6K1.1|TRPC_CAMC1 RecName: | fig|6666666.462.peg.1173;Name=Indole-3-glycerol phosphate synthase (EC4.1.1.48);Ontology_term=KEGG_ENZYME:4.1.1.48 |
| A8Z6J5.1|PURL_CAMC1 RecName: | fig|6666666.462.peg.297;Name=Phosphoribosylformylglycinamidinesynthase2C synthetase subunit (EC6.3.5.3);Ontology_term=KEGG_ENZYME:6.3.5.3 |
| A8Z6I6.1|EFG_CAMC1 RecName: Full=Elongation | fig|6666666.462.peg.615;Name=Translation elongation factor G |
| A8Z6I5.1|RS12_CAMC1 RecName: Full=30S ribosomal | fig|6666666.462.peg.617;Name=SSU ribosomal protein S12p (S23e) |
| A8Z6G1.1|FTHS_CAMC1 RecName: | fig|6666666.462.peg.1470;Name=Formate--tetrahydrofolate ligase (EC6.3.4.3);Ontology_term=KEGG_ENZYME:6.3.4.3 |
| A8Z6G0.1|OBG_CAMC1 RecName: Full=GTPase obg; | fig|6666666.462.peg.1128;Name=COG0536: GTP-binding protein Obg |
| A8Z6F6.1|RNC_CAMC1 RecName: Full=Ribonuclease | fig|6666666.462.peg.1518;Name=Ribonuclease III (EC3.1.26.3);Ontology_term=KEGG_ENZYME:3.1.26.3 |
| A8Z6F5.1|AROC_CAMC1 RecName: Full=Chorismate | fig|6666666.462.peg.1224;Name=Chorismate synthase (EC4.2.3.5);Ontology_term=KEGG_ENZYME:4.2.3.5 |
| A8Z6E7.1|NUOK_CAMC1 RecName: Full=NADH-quinone | fig|6666666.462.peg.1292;Name=NADH-ubiquinone oxidoreductase chain K (EC1.6.5.3);Ontology_term=KEGG_ENZYME:1.6.5.3 |
| A8Z6E5.1|NUOD_CAMC1 RecName: Full=NADH-quinone | fig|6666666.462.peg.1285;Name=NADH-ubiquinone oxidoreductase chain D (EC1.6.5.3);Ontology_term=KEGG_ENZYME:1.6.5.3 |
| A8Z6D9.1|END4_CAMC1 RecName: Full=Probable | fig|6666666.462.peg.1547;Name=Endonuclease IV (EC3.1.21.2);Ontology_term=KEGG_ENZYME:3.1.21.2 |
| A8Z6D5.1|AROA_CAMC1 RecName: | fig|6666666.462.peg.1446;Name=5-Enolpyruvylshikimate-3-phosphate synthase(EC 2.5.1.19);Ontology_term=KEGG_ENZYME:2.5.1.19 |
